# A Neutral “Masked” Diborene and Its Reactivity Toward Metal‐Free Pyridine Homocoupling

**DOI:** 10.1002/anie.2388449

**Published:** 2026-05-15

**Authors:** Ke Li, Arseni Kostenko, John A. Kelly, Tobias Weng, Shigeyoshi Inoue

**Affiliations:** ^1^ TUM School of Natural Sciences Department of Chemistry Catalysis Research Center and Institute of Silicon Chemistry Technische Universität München Garching bei München Germany

**Keywords:** masked diborene, free diborene, reversible interconversion, pyridine homocoupling

## Abstract

An unprecedented neutral “masked” non‐external‐donor‐stabilized diborene is generated via intramolecular arene dearomatization of an N‐heterocyclic imine (NHI)‐supported free diborene. The free diborene intermediate is photochemically accessible from the masked form, as supported experimentally through chalcogenation reactions. Computational studies elucidate an alkyne‐like bonding pattern and a triplet ground state with a Δ*E*_S‐T_ = +1.2 kcal mol^−1^ for the NHI‐supported free diborene. Remarkably, the “masked” diborene activates pyridines, promoting transition‐metal‐free C−C bond formation with high C2 regioselectivity to afford homocoupling products under mild conditions. Mechanistic investigations reveal that stepwise coordination and B−C bond cleavage produce a bis‐pyridine diborene intermediate, which undergoes reductive coupling via intrinsically strong double single‐π‐electron transfer from the B═B unit to the intramolecular pyridine ligands. Furthermore, oxidation with *p*‐benzoquinone allows the release of free 2,2′‐bipyridine derivatives. These findings provide access to insight into a hitherto elusive free diborene species, establish a previously unknown reactivity mode for diborenes, and demonstrate that this “masked” diborene functions as an exceptionally powerful double single‐electron donor with the potential to mimic transition‐metal behavior in the construction of organic molecules.

## Graphical abstract

An unprecedented neutral “masked” diborene, formed via intramolecular arene dearomatization of an N‐heterocyclic imine‐supported free diborene, can be photochemically released to regenerate the highly reactive triplet diborene intermediate. Computational analysis elucidates the bonding situation of the hitherto elusive free diborene species, and the system establishes a previously unknown reactivity mode for diborenes that promotes efficient C2‐selective homocoupling of N‐heteroarenes via intrinsically powerful double single‐electron transfer.

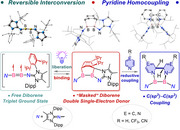

## 1 Introduction

**FIGURE 1:**
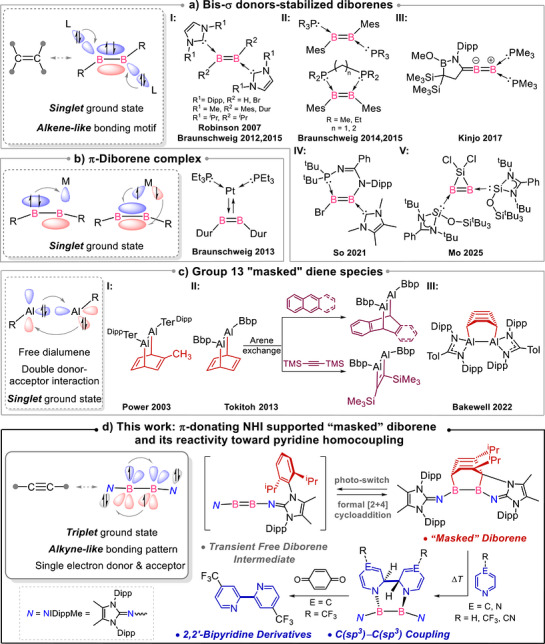
(a) Simplified schematic valence‐bond description of bis‐Lewis base‐stabilized diborenes (R = substituents, L = donor ligands) and selected examples of NHC‐, phosphine‐, and silylene‐ligand‐stabilized diborenes (Dipp = 2,6‐(*^i^* Pr)_2_C_6_H_3_; Mes = 2,4,6‐Me_3_C_6_H_2_; Dur = 2,3,5,6‐Me_4_C_6_H); (b) simplified schematic diagram of the major interactions in a transition‐metal π‐diborene complex (M = Pt^0^) and the corresponding example; (c) simplified schematic valence‐bond description of a free dialumene and selected examples of “masked” dialumenes as well as their reactivities (^Dipp^Ter = 2,6‐Dipp_2_C_6_H_3_, Bbp = 2,6‐[CH(SiMe_3_)_2_]_2_C_6_H_3_, Tol = 4‐MeC_6_H_5_); (d) this work.

The element boron preferentially adopts multicenter bonding to compensate for its electron deficiency, as it is reluctant to form electron‐precise homonuclear multiple bonds with itself [1]. Consequently, multiply bonded boron compounds are intrinsically unstable. However, they have garnered interest due to their distinctive structural and bonding characteristics. To access these compounds, sterically bulky ligands and/or Lewis basic donors have been introduced to provide kinetic as well as electronic stability [1, 2]. The simple linear, acetylene‐like diborene HB═BH molecule possessing a triplet ground state was detected using electron paramagnetic resonance (EPR) and infrared (IR) spectroscopy through matrix isolation at 4 K [3, 4]. To date, diborenes have been stabilized primarily through coordination by strong *σ*‐donor ligands or via π‐backdonation from transition metals. In 2007, Robinson and coworkers reported the first neutral bis‐base‐stabilized diborene, IDipp(H)B═B(H)IDipp (IDipp = 1,3‐bis(Dipp)imidazol‐2‐ylidene, Dipp = 2,6‐diisopropylphenyl), synthesized by reducing IDipp(BBr_3_) and subsequent abstraction of hydrogen atoms from the solvent (Figure 1a‐I) [5]. In 2012, the selective reduction of IDipp(Br)_2_BB(Br)_2_IDipp resulted in the formation of IDipp(Br)B═B(Br)IDipp (Figure 1a‐I) [6]. Subsequently, a series of other N‐heterocyclic carbene (NHCs)‐stabilized alkyl‐ or aryl‐substituted diborenes L(R)B═B(R)L (where L = IMe, I*^i^* Pr (IMe = 1,3‐bis(methyl)imidazol‐2‐ylidene, I*^i^* Pr = 1,3‐bis(isopropyl)imidazol‐2‐ylidene) and R = Mes, Dur, *^i^* Pr (Mes = 2,4,6‐trimethylphenyl, Dur = 2,3,5,6‐tetramethylphenyl)) were reported by Braunschweig and coworkers (Figure 1a‐I) [7, 8]. In addition to carbene stabilization, phosphine ligands were also used to form acyclic or cyclic, symmetric or asymmetric diborenes (Figure 1a‐II, III, IV) [9, 10, 11, 12]. Recently, Mo and coworkers utilized a bulky silylene to stabilize a diborene featuring a three‐membered −B−Si−B− ring (Figure 1a‐V) [13]. Apart from σ‐donors, Braunschweig in 2013 employed π‐backdonation from a Pt^0^ center into empty bonding π‐orbitals of a Dur‐substituted diborene, achieving a breakthrough in transition‐metal π‐diborene complexes (Figure 1b) [14]. Notably, the stable diborenes uniformly exhibit singlet ground states and a predominantly alkene‐like bonding motif (Figure 1a). While such external donor interactions confer stability, they simultaneously perturb the inherent electronic structure and obscure the genuine reactivity of the B═B π‐system. Consequently, despite significant fundamental interest, a donor‐ligand‐independent diborene RB═BR remains elusive and represents a longstanding challenge. As a result, its bonding nature and intrinsic reactivity have yet to be explored.

Heavier group 13 doubly bonded species (RE═ER, where E = Ga, In, Tl, R = terphenyl) were successfully isolated two decades ago [15, 16, 17]. Nonetheless, boron‐based doubly bonded species are inherently highly reactive owing to strong true covalent σ‐ and π‐bonding interactions and a substantially reduced HOMO‐LUMO gap relative to their heavier congeners. The latter typically exists as a weakly dimerized species, exhibiting diminished double‐bond character and lower reactivity. As comparatively stronger dimers formed through double donor‐acceptor interactions between two singlet aluminylenes (Figure 1c) [2], RAl═AlR species display stabilities between those of diborenes and the heavier analogs. As a result, isolable RE═ER (E = B, Al) species are rarely observed without substantial electronic or steric stabilization, except for an example being the dialuminene supported by a significantly bulky terphenyl substituent reported by Power and coworkers in 2024 [2]. Prior to the case above, compounds containing Al = Al bonds were usually trapped as their cycloaddition products [18, 19, 20, 21]. For example, in 2003, a “masked” dialuminene, formed through the reduction of a Dipp‐substituted 1,2‐diiodoalane, followed by the dearomatization of toluene, was reported (Figure 1c‐I) [18]. In 2013, Tokitoh and coworkers replaced the Dipp group with Bbp (Bbp = 2,6‐[CH(SiMe_3_)_2_]_2_C_6_H_3_) and trapped a dialumene‐benzene adduct. This “masked” dialumene exhibited reactivity toward arenes and alkynes, yielding the corresponding arene‐exchanged products (Figure 1c‐II) [19]. In 2022, the group of Bakewell reduced an Al(III) dihydride compound in benzene, yielding an intermolecular [2 + 4] cycloaddition product (Figure 1c‐III) [21]. Despite numerous advances in low‐valent group 13 chemistry, a “masked” diborene species has been unknown so far.

To stabilize a low‐valent multi‐bonded boron species, employing a sterically hindered and electron‐rich substituent is a promising strategy. The anionic N‐heterocyclic imidazolin‐2‐iminato (NHI) substituent is capable of donating up to 2σ and 4π electrons, as specifically illustrated by a canonical resonance form [22] in which the exocyclic nitrogen atom has substantial polarization and carries two formal negative charges. This is accompanied by effective delocalization of positive charge density into the imidazoline ring, markedly enhancing its π‐donating properties. Additionally, with sterically bulky substituents, it effectively provides both thermodynamic and kinetic stabilization to electron‐deficient species by shielding the reactive centers and preventing unwanted side reactions [22]. Several low‐valent main‐group element‐based compounds supported by NHIs [23, 24, 25, 26, 27] have been reported to date.

In this work, a monoanionic bulky NHI substituent was employed to enable the synthesis and isolation of a free diborene without additional donor stabilization. Unexpectedly, this approach led to the discovery of an unprecedented “masked” diborene and revealed an alkyne‐like bonding pattern of the free diborene intermediate generated photochemically from the masked form (Figure 1d). Further investigations into its reactivity toward N‐heteroarenes, including pyridines and pyrazine, demonstrated transition‐metal‐free C2‐selective homocoupling mediated by a main‐group diborene species.

## 2 Results and Discussion

**SCHEME 1:**
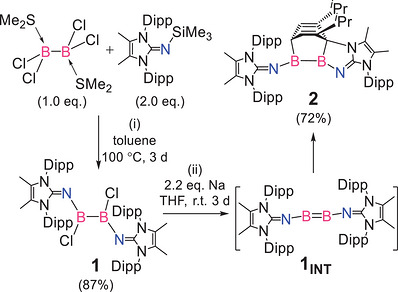
Synthesis and reduction of dichlorodiboron **1** to form compound **2** via intermediate **1_INT_**.

To access a neutral NHI‐supported diborene, the precursor dichlorodiboron **1** was synthesized by treating one equivalent of diboron tetrachloride bis(dimethylsulfide) adduct with two equivalents of ^DippMe^NHI(TMS) (^DippMe^NHI(TMS) = 1,3‐bis (2,6‐diisopropylphenyl)‐4,5‐dimethyl‐N‐(trimethylsilyl)‐1,3‐dihydro‐2H‐imidazol‐2‐imine) in toluene and stirred at 100°C for 3 days (Scheme 1). Compound **1** was reduced with a slight excess of sodium metal, yielding a green heterogeneous mixture that ultimately afforded compound **2** in 72% yield. In the ^11^B NMR spectrum, two broad resonances appear at 30.1 and 57.9 ppm, which are consistent with the computational predictions (33.5 and 56.1 ppm), confirming the presence of two three‐coordinate boron centers (calculations were carried out using ORCA software [28]).

**FIGURE 2:**
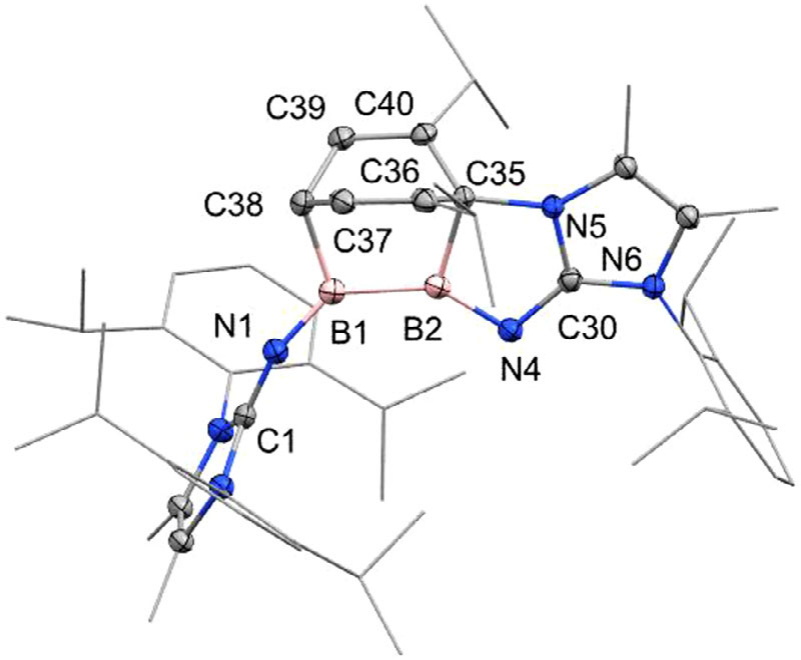
The thermal ellipsoid drawing of compound **2** at a 50% probability level. Hydrogen atoms are omitted, and parts of groups are represented as wireframes for the sake of clarity. Selected bond lengths (Å): B1−B2 = 1.7204(18), B1−C38 = 1.6463(17), B2−C35 = 1.6655(16), C35−C36 = 1.5195(16), C36−C37 = 1.3408(17), C37−C38 = 1.5091(17), C38−C39 = 1.5041(16), C39−C40 = 1.3402(16), C40−C35 = 1.5199(16), B1−N1 = 1.3652(16), B2−N4 = 1.4291(16), N5−C35 = 1.4611(14).

Compound **2** was crystallized at −30°C from a penta‐fluorobenzene/pentane solution. It appears pale yellow, with its structure shown in Figure 2. The B1−B2 bond length is 1.7204(18) Å, which falls within the typical range for B−B single bonds (1.682−1.762 Å) [29, 30] in three‐coordinate diboranes. The B1−C38 and B2−C35 bond lengths are 1.6463(17) Å and 1.6655(16) Å, respectively. They are noticeably longer than the typical B(sp^2^)−C(sp^2^) single bond (1.59 Å) [31] and are longer than B(sp^2^)−C(sp^3^) single bond (1.618 Å) [32] in a boron‐containing five‐membered ring. The Wiberg Bond Indexes (WBIs) of the two B−C bonds are 0.67 and 0.77 (Figure S65), respectively, suggesting σ‐bond characters weaker than that of a typical single bond. Additionally, the distorted C_6_ fragment features four single bonds (1.5041(16)–1.5199(16) Å) and two double bonds (1.3408(17) Å, 1.3402(16) Å). This indicates that the arene ring in **2** is de‐aromatized by the B═B double bond in intermediate **1_INT_**; thus, **2** is regarded as a formal intramolecular [2 + 4] cycloaddition “masked” diborene. While dearomatization of rigid arenes usually relies on photochemical or transition‐metal‐mediated conditions, recent advances have demonstrated that low‐valent main group compounds can also promote the activation of stable aromatic systems. Several examples of arene dearomatization mediated by low‐valent Si, Ge, Al, B, and P species have been reported, predominantly proceeding via cycloaddition or Büchner ring‐expansion pathways [33].

**FIGURE 3:**
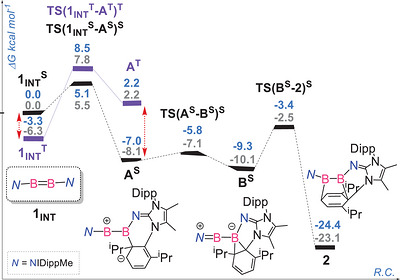
Free energy reaction coordinate diagram of the proposed mechanism for the formation of “masked” diborene **2** at the (SMD) PW6B95‐D4/def2‐QZVPP//r^2^SCAN‐3c level of theory. The grey and blue values represent solvation effects of THF and benzene, respectively.

Calculations of the proposed reaction mechanism for the formation of **2** from diborene **1_INT,_** generated via the reduction of the dichlorodiboron, are presented in Figure 3. The free energy of the triplet state **1_INT_^T^** is lower than that of the singlet state **1_INT_^S^** by 6.3 kcal mol^−1^ in THF. However, the singlet transition state **TS(1_INT_^S^‐A^S^)^S^** that leads to the formation of intermediate **A^S^** is 2.3 kcal mol^−1^ lower in energy than the corresponding triplet transition state **TS(1_INT_^T^‐A^T^)^T^**. Intermediate **A^S^** is a Meisenheimer‐type complex [34] that is commonly described as a reactive intermediate in the reactions of low‐valent main group complexes with C_6_ aromatic compounds [27, 35]. The next step is the formation of intermediate **B^S^** at −10.1 kcal mol^−1^ via a low barrier of 1.0 kcal mol^−1^. The resulting zwitterionic species **B^S^** features a three‐membered −B−C−C− ring. Similar dearomatization pathways are common in main‐group chemistry. For example, in the case of some acyclic silylenes, the process can eventually lead to the C−C bond cleavage and the C_6_ ring‐expansion, forming a seven‐membered silepin [27, 33, 36, 37, 38, 39]. In this case, the presence of an additional intramolecular electron‐deficient boron center in **B^S^** allows for the formation of the isolated compound **2** via a barrier of 7.6 kcal mol^−1^. **2** is a product of a formal [4 + 2] cycloaddition [18, 19, 21, 40, 41] and can also be considered as a “masked” diborene, although the thermal barrier for the reverse reaction is relatively high, that is, 28.6 and 29.5 kcal mol^−1^ in THF and benzene, respectively, the free diborene species can be accessed under ambient conditions photochemically (*vide infra*). Intermolecular reaction featuring dearomatization of benzene by **1_INT_** was also considered (Figure S71). This process is, however, kinetically unfavored by 10.1 kcal mol^−1^ in comparison to the intramolecular dearomatization, which yields **2**.

**FIGURE 4:**
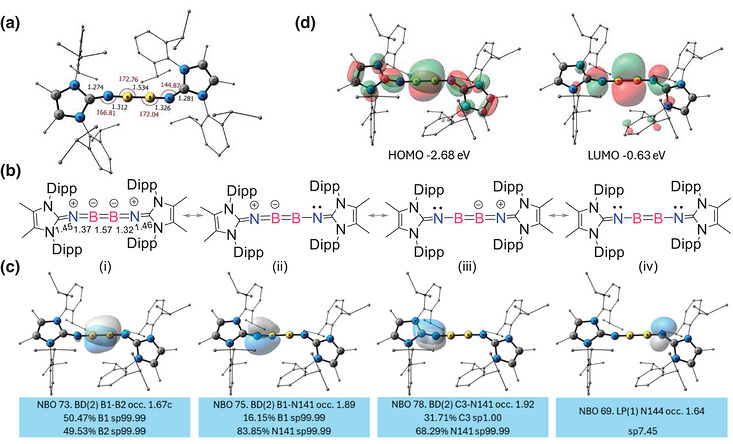
(a) Optimized structure of **1_INT_^S^**; hydrogens are omitted for clarity, and Me and Dipp substituents are represented by wireframes; (b) possible resonance structures of **1_INT_^S^** with the WBIs of the central CNBBNC moiety; (c) selected Natural Bond Orbitals (NBOs) of **1_INT_^S^** (iso = 0.03); (d) selected molecular orbitals of **1_INT_^S^** (iso = 0.03).

We carried out further computational analysis to elucidate the properties and electronic structure of **1_INT_**. The optimized structure of **1_INT_^S^** (Figure 4a) exhibits a B−B bond length of 1.534 Å, which is at the lower end of the reported B−B bonds in base‐stabilized diborenes and is similar to that of parent HB═BH (1.528 Å). The B−N bond lengths of 1.31 and 1.33 Å are in the range of B═N bonds (∼1.39 Å) [42, 43]. The N−C^NHC^ bonds are also short, 1.27 and 1.28 Å, indicating double bond character [42, 44, 45]. The NBO calculation converges to the electronic structure corresponding to the resonance forms (ii) and (iii) (Figure 4b). These feature B═B, N═B, and two C═N double bonds, as well as a single bond in the second B−N moiety, with a lone pair on the nitrogen atom (Figure 4c). The lone pair is highly delocalized to the lone vacancy p orbital of the adjacent boron atom (Figure S66). These geometrical and electronic features are consistent with a cumulenic bonding pattern −C═N═B═B═N═C−, allowing **1_INT_^S^** to be regarded as an analog of a C_4_ cumulene (resonance form (i)). Additionally, the π(B═N) orbital of the first B═N unit is strongly polarized toward N, consistent with a description in which a nitrogen lone pair donates into an empty boron p orbital. Thus, the interpretation of **1_INT_^S^** in resonance form (iv) also becomes relevant. Overall, the electronic structure of **1_INT_^S^** incorporates contributions from resonance forms (i)–(iv).

As mentioned above, the ground state of **1_INT_** is a triplet with a Δ*E*_S‐T_ = *E*_S_–*E*_T_ = +1.2 kcal mol^−1^. This is much smaller than the singlet‐triplet gap for the ground‐state triplet parent diborene HB = BH Δ*E*_S‐T_ = +22.5 kcal mol^−1^ (+20.6 [46] and +16.6 kcal mol^−1^ [46] in previous reports). The considerably smaller Δ*E*_S‐T_ in **1_INT_** compared to the parent system can be attributed to the enhanced stabilization of the singlet state in the NHI‐substituted diborene. This arises from the above‐mentioned ability of the π(B−B) orbital to be stabilized by delocalization into the adjacent π*(N−C) orbitals, as well as the stabilization of the empty p orbitals of the B by the lone pairs of the adjacent nitrogen atoms. These interactions lead to the N−B double bonding character, which results in a cumulenic structure. Such stabilization mechanisms are not available in the parent system.

NBO analysis of the frontier canonical molecular orbitals (Figure 4d and Figure S67) reveals that the HOMO is predominately a bonding orbital with major contribution from the π(B═B) localized at the B═B moiety, whereas the LUMO corresponds mainly to an antibonding interaction involving the B═N fragments. As discussed above, the bonding π(B−N) interactions are strongly polarized toward the N centers, and hence, the lobe of the antibonding LUMO is predominately located on the B atoms. The perpendicular orientation of the electron‐donor HOMO and the electron‐acceptor LUMO with a very small energy gap of only 2.05 eV endows the boron centers in **1_INT_^S^** with pronounced amphiphilic character and an incredible ability to dearomatize a C_6_ aromatic moiety with a barrier of merely 5.5 kcal mol^−1^, which is significantly lower than that of most processes mediated by other main‐group species [27, 33, 39, 40, 47, 48, 49, 50, 51, 52, 53, 54, 55].

**FIGURE 5:**
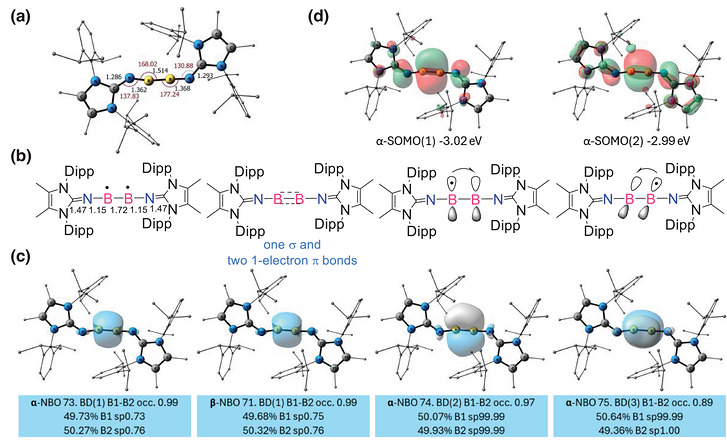
(a) Optimized structure of **1_INT_^T^**; hydrogens are omitted for clarity, and Me and Dipp substituents are represented by wireframes; (b) Lewis structures showing WBIs of the central CNBBNC moiety and displaying the unpaired‐electron interaction in **1_INT_^T^**; (c) selected NBOs of **1_INT_^T^** (iso = 0.03); (d) selected canonical orbitals of **1_INT_^T^** (iso = 0.03).

The optimized geometry of the triplet **1_INT_^T^** (Figure 5a) exhibits a slightly shorter B═B bond of 1.514 Å in comparison to **1_INT_^S^**, and it is nearly identical to the B−B distance in HB═BH (1.513 Å). Also, the B═N bonds are substantially longer (1.362 and 1.368 Å) than those in **1_INT_^S^**. The bond length trend is consistent with the WBIs (Figure 5b, left). According to the NBO analysis, the electronic structure can be described as featuring a delocalized π‐conjugation within the C−N−B moieties (Figures S68–S69). In terms of the B−B bonding, in addition to the σ‐bond represented by the corresponding α‐ and β‐spin NBOs (Figure 5c), there are two additional mutually perpendicular α‐spin bonding interactions. These can be interpreted as interactions between the unpaired electrons and the vacant p‐orbitals of the adjacent boron center (Figure 5b). Consequently, the triplet state can be described as having a B−B linkage composed of one σ‐bond and two orthogonal singly occupied π‐type orbitals, corresponding to two one‐electron π bonds [4, 14, 29]. This situation is reflected in the nearly degenerate singly occupied molecular orbitals (SOMOs; Figure 5d), which correspond to the π‐bonding interactions. The Mulliken atomic spin population analysis indicates that 0.77 and 0.76 of the spin density are localized on each of the boron atoms. An extended discussion of **1_INT_^S^** and **1_INT_^T^** is provided in the computational section of the Supporting Information.

**SCHEME 2:**
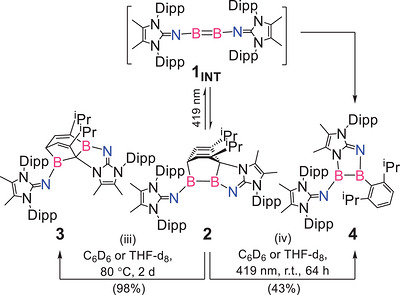
Transformations among isomers **2**, **3**, **1_INT_**, and **4**.

**FIGURE 6:**
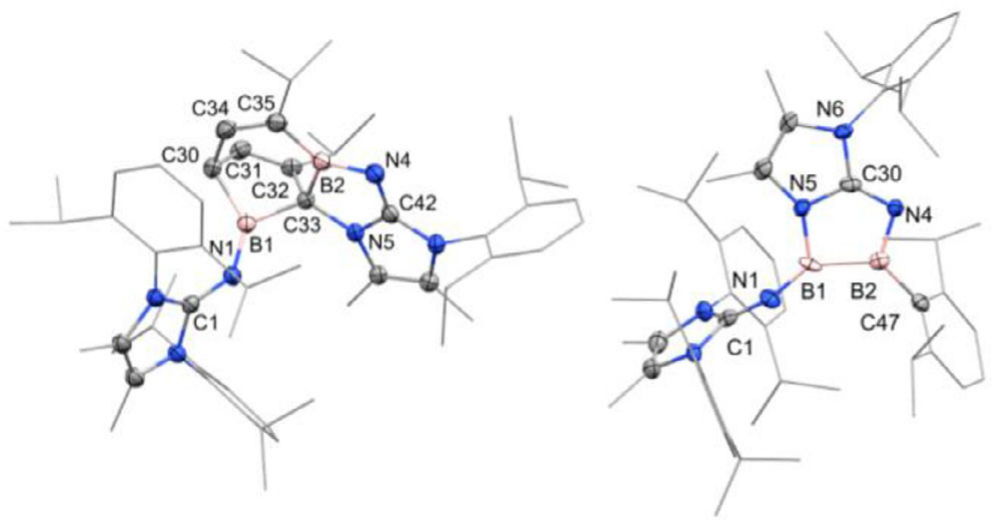
The thermal ellipsoid drawing of isomer **3** (left) and isomer **4** (right) at the 50% probability level. Hydrogen atoms are omitted, and parts of groups are represented as wireframes for the sake of clarity. Selected bond lengths (Å): **3**: B1−C30 = 1.621(3), B1−C33 = 1.610(2), B2−C33 = 1.610(3), B2−C35 = 1.550(3), B1−N1 = 1.365(2), B2−N4 = 1.433(3), N5−C33 = 1.455(2); **4**: B1−B2 = 1.768(4), B1−N5 = 1.523(4), B2−N4 = 1.428(4), N4−C30 = 1.324(3), N5−C30 = 1.382(3).

Next, the reactivity of the “masked” diborene **2** was studied. Compound **2** was nearly fully converted to isomer **3** in benzene or THF solution when the temperature was elevated to 80°C and maintained for 2 days (Scheme 2). Irradiation of **2** at 419 nm under ambient conditions for 64 h resulted in isomerization to **4** with a conversion of 43%, whereas irradiation at 300 nm for 2 days afforded **4** in nearly quantitative conversion (Figure S16). Compound **4** was also observed during the reduction process under ambient conditions. All products were successfully isolated and fully characterized. The crystal structures of isomers **3** and **4** are shown in Figure 6. The ^11^B NMR spectra reveals the broad ^11^B signals for the three‐coordinate B centers in **3** and **4** (44.8 and 49.9 ppm for **3**; 27.8 and 63.7 ppm for **4**), which are consistent with the calculated values (predicted: 42.3 and 48.4 ppm for **3**; 26.0 and 60.4 ppm for **4**).

**FIGURE 7:**
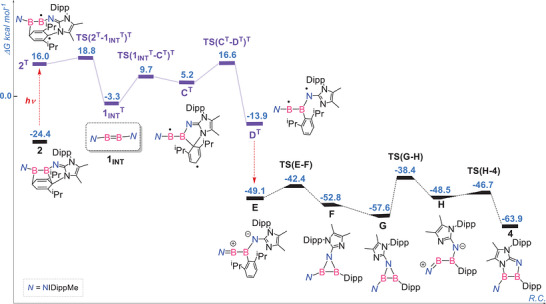
Free energy reaction coordinate diagram of the proposed mechanism for isomerization of **2** to **4** at the (SMD = benzene) PW6B95‐D4/def2‐QZVPP//r^2^SCAN‐3c level of theory.

The thermal transformation **2** → **4** involves a high activation barrier of 32.5 kcal mol^−1^ (Figure S70), which is not feasible under ambient conditions. The proposed isomerization mechanism is shown in Figure 7. Upon irradiation of **2** at 419 nm, homolytic cleavage of a B−C bond occurs, generating the first excited state **2ᵀ**. This is followed by the cleavage of the second B−C bond, resulting in the formation of **1_INT_^T^**. One of the B‐centered radicals then bonds with the *C*(N) atom in the arene ring, producing the diradical intermediate **C^T^**. Subsequently, the unpaired electron of the C‐centered radical is transferred to the exocyclic imino‐nitrogen atom, leading to the formation of **D^T^**. The triplet **D^T^** then relaxes to form the zwitterionic singlet intermediate **E^S^**. **E^S^** proceeds through a nucleophilic attack at the boron center, generating a three‐membered −B−N−B− ring. This is followed by geometric twist and ring opening via the transition state **TS(G‐H)**, ultimately triggering the B−N bond formation and affording **4**. Since the transformation from **1_INT_^T^** to **4** does not require particularly high activation energies (19.9 kcal mol^−1^), the process can occur spontaneously, thereby accounting for the experimental observation of **4** apart from **2** during the reduction under ambient conditions.

**SCHEME 3:**
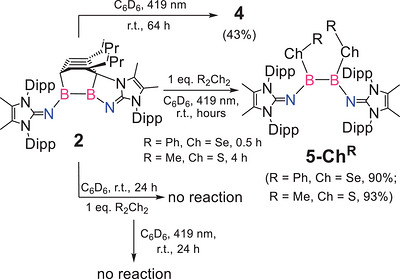
Trapping reactions for the triplet intermediate **1_INT_^T^**.

To verify the proposed mechanism, the highly reactive and short‐lived intermediate, **1_INT_^T^**, was trapped using radical probes. Upon irradiation at 419 nm, mixing one equivalent of dichalcogenides R_2_Ch_2_ (R = Ph, Me; Ch = Se, S) with **2** in C_6_D_6_ led to the selective conversion of **2** into **5‐Ch^R^** (Scheme 3). To exclude the possibility that this conversion was initiated by direct reaction of RCh• radicals with **2**, a series of comparative control experiments were also conducted (Figures S27–S31). When **2** was mixed with R_2_Ch_2_ at room temperature for 24 h, no detectable reaction was observed. Moreover, irradiation of Ph_2_Se_2_ or Me_2_S_2_ in C_6_D_6_ at 419 nm at room temperature for 24 h resulted in neither color change nor observable decomposition products, indicating that homolytic cleavage of the Ch−Ch bond to generate RCh• radicals does not occur under these conditions. These results demonstrate that the kinetically favored formation of **5‐Ch^R^** likely arises from the immediate trapping of the diradical species **1_INT_^T^**, generated via photochemical B−C bond homolysis of **2**.

**FIGURE 8:**
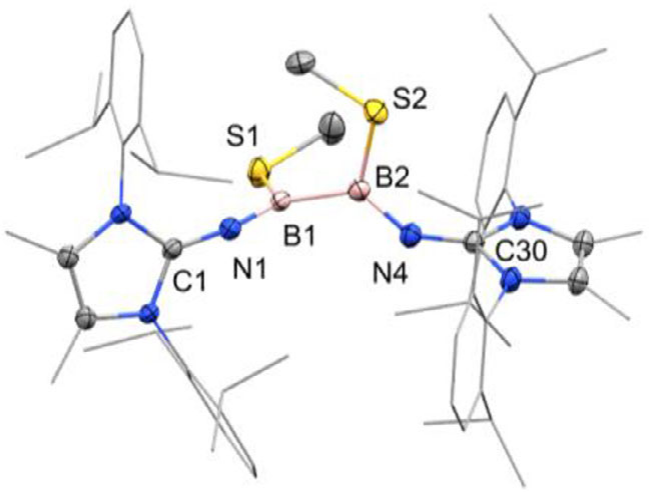
The thermal ellipsoid drawing of **5‐S^Me^** at a 50% probability level. Hydrogen atoms and solvent molecules are omitted, and parts of groups are represented as wireframes for the sake of clarity. Selected bond lengths (Å) and torsion angles (°): B1−B2 = 1.717(3), B1−S1 = 1.855(2), B2−S2 = 1.850(2), B1−N1 = 1.367(3), B2−N4 = 1.382(3), N1−C1 = 1.269(3), N4−C30 = 1.283(3); S1−B1−B2−S2 = 88.98(17).

For **5‐Se^Ph^**, a broad ^11^B NMR signal appears at 30.79 ppm. The ^77^Se NMR spectrum displays a sharp singlet peak at 290.5 ppm, significantly upfield shifted compared to Ph_2_Se_2_ (461.4 ppm), which is consistent with the formation of a diselenium diborane. The crystal structure of **5‐S^Me^** is shown in Figure 8. The bond length of B1−B2 is 1.717(3) Å, within the typical range of B−B single bonds. The average B−S bond distance is 1.853 Å, similar to the B−S bond lengths in the triplet diradical species [^Me^cAAC(RS)B−B(SR)cAAC^Me^]^2•^ (1.84–1.86 Å, R = *^n^* Bu, Ph) [56]. The torsion angle of S1−B1−B2−S2 is near 90° (88.98(17)°), showing two orthogonal NBS planes with the dihedral angle of 89.29° (Figure S62). In the ^11^B NMR spectrum, a broad signal at 39.5 ppm was observed, which is in good agreement with the computational predictions of 39.4 and 38.8 ppm. These reactions provide evidence that the transformation from **2** to **4** under photoirradiation proceeds via triplet **1_INT_^T^**. They also support that **2** features an intramolecular formal “masked” B═B double bond character.

We also attempted to directly observe the **1_INT_^T^** using EPR spectroscopy by continuous irradiation of a toluene solution of **2** at temperatures of 170 K and above (for details, see the EPR section in the Supporting Information). Upon irradiation, a persistent radical species is observed (Figures S72–S73). However, DFT simulations indicate that the observed spectrum could not be correlated with the reactive intermediates shown in Figure 7. Those are presumably short‐lived, and the observed signal probably belongs to a secondary paramagnetic species or decomposition products. It is possible that owing to its high reactivity and extremely short lifetime, **1_INT_^T^** decays on a timescale beyond the detection limit of EPR spectroscopy.

**SCHEME 4:**
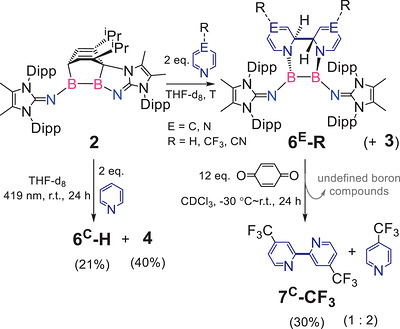
Homocoupling reactivity of **2** toward N‐heteroarenes.

When **2** was heated with pyridine in THF at 80°C for 2 days, the homocoupling product **6^C^‐H** along with compound **3** was obtained (Scheme 4 and Table 1). Bipyridines establish a class of chelating heterocyclic ligands that can account for the majority of applications in coordination chemistry [57, 58], catalysis [59], supra‐ [60], nano‐ [57], and macromolecular [61] chemistry, as well as photochemistry [62]. Due to their high applicability, synthetic methods for preparing these compounds have been extensively studied. Representative examples involve the homo‐ and hetero‐coupling of pyridine derivatives, typically in the presence of transition‐metal catalysts. Common coupling methods include Suzuki‐Miyaura, Negishi, Kumada, Hiyama, Stille, Wurtz, and Ullmann reactions [63, 64, 65]. Another notable advancement involving PCl_3_ or POCl_3_ reagents is phosphorus‐ligand coupling, in which the transition metal catalyst is replaced during the biaryl bond‐forming step [66, 67, 68, 69, 70]. To the best of our knowledge, aside from two known examples of main‐group diboron compounds that trigger the formation of 4,4′‐bipyridine analogs via pyridinyl radical intermediates [71, 72] and a homocoupling of isoquinolines promoted by chiral organo‐diboron reagents via a concerted [3,3]‐sigmatropic rearrangement [73], the C−C bond formation between α‐positions of unsubstituted pyridine directly mediated by a main‐group boron species has been rarely explored. Previous reports indicate that the catalytic cycles of Cu‐, Ni‐, Pd‐, or Ru‐catalyzed pyridine coupling typically proceed through oxidative addition, transmetallation, and reductive elimination steps [63, 74, 75]. For phosphorus‐ligand coupling reactions or coupling of substituted pyridines catalyzed by the organo‐diboron reagent B_2_pin_2_, radical mechanisms are generally employed [70, 76, 77]. To further investigate the mechanism of the coupling reaction promoted by our “masked” diborene, pyridine derivatives with C4‐position‐bearing electron‐donating groups (e.g., NMe_2_) or electron‐withdrawing groups (e.g., CF_3_ and CN) were employed as substrates (Scheme 4 and Table 1). Homocoupling was unsuccessful with 4‐dimethylaminopyridine (DMAP). In contrast, CF_3_‐ or CN‐substituted pyridines significantly accelerated the coupling process and markedly improved selectivity, affording **6^C^‐CF_3_** and **6^C^‐CN** at −30°C within 30 min in 95% and 92% yields, respectively. In addition, reaction of pyrazine with **2** at room temperature for 1 h selectively afforded the C−C coupled product **6^N^**. Notably, homocoupling of pyridine promoted by **2** also proceeds photolytically (Scheme 4 and Figures S36–S39). No reaction occurred under ambient conditions, whereas irradiation at 419 nm afforded **6^C^‐H** as a minor product, likely because conversion of **2** to **4** is kinetically favored. Collectively, these observations support a diborene‐mediated pathway. Remarkably, when an excess of *p*‐benzoquinone was added to **6^C^‐CF_3_** at −30°C and the reaction was allowed to proceed for 24 h as it gradually warmed to room temperature, the dianionic 2,2'‐bipyridine unit was released and further oxidized via single‐electron transfer (SET) and proton transfer processes, affording product **7^C^‐CF_3_** (Figures S54–S57).

**TABLE 1:** Substrate scope.

Entry	Substrate	T (°C)	Time (h)	Product		Yield (6^E^‐R)^a^	Ratio (6^E^‐R:3)^b^
1	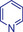	80	48	**6^C^‐H**	**3**	48%	1:1
2	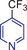	−30	0.5	**6^C^‐CF_3_**	**3** n.d.^c^	95%	—
3	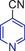	−30	0.5	**6^C^‐CN**	**3** n.d.	92%	—
4	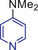	80	48	**6^C^‐NMe_2_**	**3** 98%	n.d.	—
5	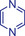	25	1	**6^N^**	**3** n.d.	92%	—

^a, b^ The yield and ratio were determined by crude ^1^H NMR analysis using mesitylene as an internal standard.

^c^ n.d. represents “not detected.”

**FIGURE 9:**
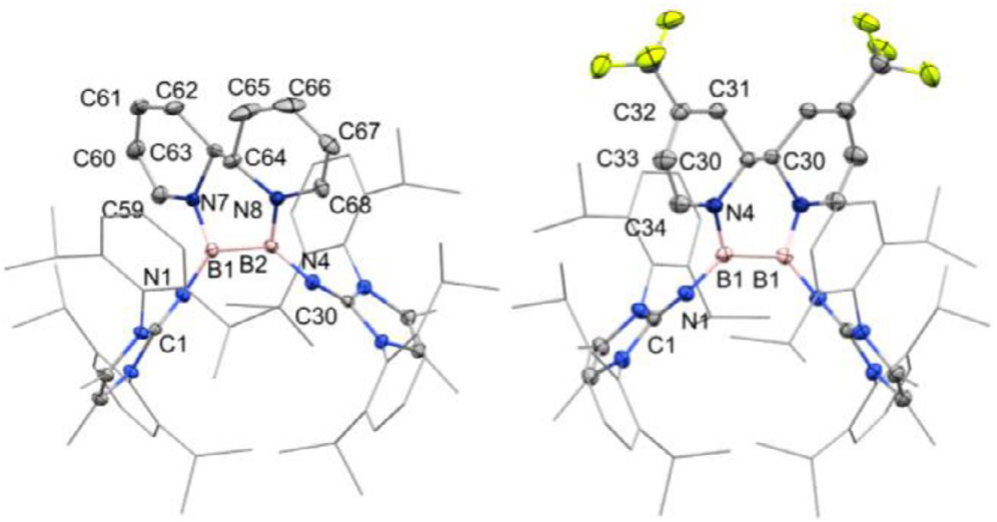
The thermal ellipsoid drawing of pyridine coupling product **6^C^‐H** (left) and 4‐(trifluoromethyl)‐pyridine coupling product **6^C^‐CF_3_** (right) at the 50% probability level. Hydrogen atoms are omitted, and parts of groups are represented as wireframes for the sake of clarity. Selected bond lengths (Å): **6^C^‐H**: B1−B2 = 1.7121(17), B1−N7 = 1.4634(15), B2−N8 = 1.4564(15), C63−C64 = 1.5447(16), **6^C^‐CF_3_**: B1−B1′ = 1.709(3), B1−N4 = 1.4688(16), C30−C30′ = 1.546(2).

All the C−C coupled products **6^E^‐R** were successfully isolated and characterized. The crystal structures of **6^C^‐ H** and **6^C^‐CF_3_** are shown in Figure 9. The B−B, C_α_−C_α_, and B−N bond lengths are approximately 1.71, 1.54, and 1.46 Å, respectively, all indicating a typical single‐bond character. For **6^C^‐H**, the ^1^H NMR signal at the C2‐position of the dearomatized pyridine appears as a singlet at 3.36 ppm, confirming that the carbon atoms connected to the protons are sp^3^ hybridized. In addition, a weak and broad ^11^B signal was detected at 33.0 ppm, which is in good agreement with computational predictions (35.3 and 35.2 ppm).

**FIGURE 10:**
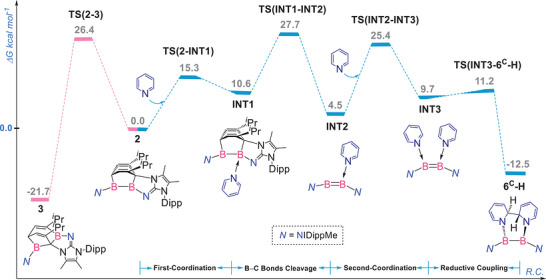
Free energy reaction coordinates diagram of the proposed mechanism for the pyridine homocoupling reaction mediated by **2** and the competitive isomerization of **2** to **3** at the (SMD = THF) PW6B95‐D4/def2‐QZVPP//r^2^SCAN‐3c level of theory.

To gain insight into the mechanism of the pyridine homocoupling reaction and the competitive production of **3**, DFT calculations were performed (Figure 10). When pyridine is mixed with **2**, a Lewis‐basic pyridine coordinates to the more electrophilic boron center (Figure S65), forming a mono‐pyridine‐coordinated “masked” diborene adduct **INT1**, driving the cleavage of the two B−C bonds and the restoration of the aromaticity of the Dipp group to form a mono‐pyridine‐diborene adduct **INT2** via the transition state **TS(INT1‐INT2)** with an activation barrier of 27.7 kcal mol^−1^. B−C bond cleavage represents the rate‐determining step during the coupling process. Subsequently, the second pyridine coordinates to the second boron center, forming the bis‐pyridine‐diborene intermediate **INT3**. This is followed by reductive C−C coupling between C2‐positions of pyridines, generating the homocoupling product facilitated by a low‐barrier transition state **TS(INT3‐6^C^‐H)**. The formation of a σ‐bond between two C(sp^3^) atoms leads to the thermally stable final product **6^C^‐H** with a free energy gain of −12.5 kcal mol^−1^. Throughout the process, the coordination of a single pyridine initially triggers the cleavage of two B−C bonds, reducing the B(II) centers to B(I). Subsequently, the B═B moiety donates two single π electrons to the pyridine ligands, leading to oxidation back to B(II). It was proposed that the driving force behind the coupled product formation is the reduction of pyridine moieties and the energy release from C(sp^3^)−C(sp^3^) bond construction. During the reaction process, the intermediates (**INT1**, **INT2**, and **INT3,** or their analogs) are not expected to be isolable or observable, as they are energetically well above the reactants on the free‐energy surface. Notably, the irreversible transformation from **2** to **3** proceeds with an activation barrier of 26.4 kcal mol^−1^ for **TS (2‐3)**, which is comparable to the rate‐determining step of the coupling process. This correlation aligns with the experimental observation of an approximately 1:1 ratio between **3** and the coupling product **6^C^‐H** when heated to 80°C (Figure S35 and Table S1). Benefiting from the electron‐withdrawing effect of the trifluoromethyl group, the activation barrier of the rate‐determining transition state for the coupling process **(TS(INT1‐INT2)_Py‐CF3_**) is lowered by 4.6 kcal mol^−1^, according to calculations at 298.15 K. The calculated barrier at 243.15 K (i.e., −30°C, the temperature of the experiment) is only 18.0 kcal mol^−1^. As a result, high temperatures are no longer a necessary condition for the coupling reaction to occur, thereby preventing the conversion from **2** to **3**. The reaction rate and selectivity of the coupling product were thus accelerated significantly as well.

## 3 Conclusion

In conclusion, we have successfully synthesized, isolated, and crystallographically characterized an unprecedented neutral “masked” diborene **2** via a free diborene intermediate featuring a nearly linear geometry and a triplet ground state with a +1.2 kcal mol^−1^ singlet‐triplet energy gap. The ground‐state triplet species **1_INT_^T^** exhibits two orthogonal single‐electron π interactions, whereas the excited singlet state **1_INT_^S^** adopts a cumulene‐like structure with pronounced amphiphilic character, leading to dearomatization of an intramolecular C_6_ aromatic moiety. The nature of the formal “masked” diborene was supported by chalcogenation reactions. Accordingly, **2** serves as a stable and isolable source of an NHI‐supported free diborene, enabling systematic investigation of the intrinsic electronic properties and reactivity of hitherto elusive free diborene species and expanding the conceptual boundaries of low‐valent p‐block chemistry. Compound **2** displays unique reactivity toward N‐heterocycles, invoking C2‐selective homocoupling that establishes a previously unknown reactivity mode for diborenes, which arises from the distinct bonding characteristics of the NHI‐supported free diborene compared to conventional Lewis base‐stabilized diborenes. This transformation efficiently promotes C−C bond formation with high regioselectivity and substrate generality under mild conditions. This alternative pathway provides a novel approach for designing and synthesizing bipyridine derivatives under mild, homogeneous conditions utilizing main‐group boron compounds and offers a promising avenue to address the challenges associated with traditional catalytic methods. Furthermore, these findings underscore the ability of **2** to function as an exceptionally powerful double single‐electron donor, highlighting its potential for reductive C−C coupling with other nitrogen‐containing substrates such as imines.

## Author Contributions

**Ke Li**: investigation, methodology, data curation, formal analysis, conceptualization, writing – original draft, writing – review and editing. **Arseni Kostenko**: data curation, formal analysis, writing – review and editing, investigation. **John A. Kelly**: data curation, formal analysis, methodology. **Tobias Weng**: methodology, data curation, formal analysis. **Shigeyoshi Inoue**: conceptualization, funding acquisition, resources, writing – review and editing, project administration, supervision, formal analysis, visualization.

## Conflicts of Interest

The authors declare no conflicts of interest.

## Supporting information




**Supporting File 1**: Experimental details include synthetic, spectroscopic, crystallographic, and computational data (PDF).

**Supporting File 2**: anie72613‐sup‐0002‐cif.zip.

## Data Availability

The data that supports the findings of this study are available in the supplementary material of this article.
