# Supplementary material for: A Neutral “Masked” Diborene and Its Reactivity Toward Metal‐Free Pyridine Homocoupling
Source: Angew Chem Int Ed Engl. 2026 May 15;65(27):e2388449. doi: 10.1002/anie.2388449 (PMC13327575; doi:10.1002/anie.2388449)
Supplement: Supplementary file 1 — Supporting File 1: Experimental details include synthetic, spectroscopic, crystallographic, and computational data (PDF). [file ANIE-65-e2388449-s002.pdf]

Supporting Information

A Neutral “Masked” Diborene and Its Reactivity toward Metal-Free Pyridine Homocoupling

Ke Li,[1] Arseni Kostenko,[1] John A. Kelly,[1] Tobias Weng,[1] and Shigeyoshi Inoue*[1]

[1]School of Natural Sciences, Department of Chemistry, Institute of Silicon Chemistry and Catalysis Research Center, Technische Universität München, Lichtenbergstraße 4, 85748, Garching bei München, Germany

Table of Contents

1. Experimental Section 3

1.1 General procedures 3

1.2 Synthesis and characterization 4

1.2.1 Synthesis of compound 1 4

1.2.2 Synthesis of compound 2 8

1.2.3 Synthesis of compound 3 11

1.2.4 Synthesis of compound 4 15

1.2.5 Synthesis of compound 5-S^{Me} 19

1.2.6 Synthesis of compound 5-Se^{Ph} 22

1.2.7 Comparative control reactions of 2 with Me_2S_2 and Ph_2Se_2 26

1.2.8 Synthesis of compound 6^C-H 31

1.2.9 Synthesis of compound 6^C-CF_3 39

1.2.10 Synthesis of compound 6^C-CN 43

1.2.11 Synthesis of compound 6^N 47

1.2.12 Synthesis of compound 7^C-CF_3 52

2. Crystallographic Section 55

2.1 General considerations 55

2.2 SC-XRD data and structure refinement 57

2.3 SC-XRD structures 59

3. Computational Details 62

4. EPR spectroscopy 119

5. References 120

1. Experimental Section

1.1 General procedures

All manipulations of air- and moisture-sensitive reactions were carried out under an atmosphere 4.6 (≥99.996%; Westfahlen AG) of argon using standard Schlenk techniques or in LAB star glovebox from M-Braun Inert Gas-System GmbH with H_2O and O_2 values below 0.5 ppm. All glassware was dried under high vacuum prior to use. PTFE-based grease (Triboflon III, Freudenberg & Co. KG) was used as the sealant. Drying of n-pentane was accomplished by a solvent purification system (SPS), degassed and stored under 4 Å molecular sieves. THF and Penta-fluorobenzene were dried with potassium mirror and CaH_2, respectively, and freshly distilled and degassed by three freeze-pump-thaw cycles prior to use. Deuterated solvents were stored under argon over activated 4 Å molecular sieves. Pyridine and 4-trifluoromethyl-pyridine were degassed and dried with 4 Å molecular sieves. Other chemicals were purchased from commercial suppliers and used as received. NMR samples were prepared under an argon atmosphere in J-Young NMR tubes equipped with PTFE valves. A quartz J-Young NMR tube and normal borosilicate J-Young NMR tubes were used for ^{11}B NMR measurements. NMR spectra were recorded on a Bruker Avance Neo 400 NMR spectrometer (^{1}H: 400 MHz, ^{13}C: 101 MHz, ^{11}B: 128 MHz, ^{19}F: 376 MHz, ^{77}Se: 76 MHz) at ambient temperature (298 K). Chemical shifts (δ) are provided in ppm. ^{1}H and ^{13}C NMR spectra are calibrated against the residual proton and natural abundance carbon resonances of the respective deuterated solvents as internal standard (C_6D_6: δ(^{1}H) = 7.16 ppm and δ(^{13}C) = 128.06 ppm; THF-d_8: δ(^{1}H) = 1.72, 3.58 ppm and δ(^{13}C) = 67.21, 25.31 ppm).[1] The following abbreviations are used to describe signal multiplicities: s = singlet, d = doublet, t = triplet, sept = septet, m = multiplet, br = broad and combinations thereof (e.g. dd = doublet of doublets). Some NMR spectra include resonances for silicone grease (C_6D_6: δ(^{1}H) = 0.29 ppm, δ(^{13}C) = 1.38 ppm; THF-d_8: δ(^{1}H) = 0.11 ppm and δ(^{13}C) = 1.20 ppm) derived from B. Braun Melsungen AG Sterican® cannulas. The asterisk (*) refers to residual peaks of solvents, silicone grease or H grease. The UV-Vis spectra were taken on an Agilent Cary 50

spectrophotometer with a Schlenk quartz cuvette at the Central Analytic Department at the TUM Catalysis Research Center. Liquid Injection Field Desorption Ionization Mass Spectroscopy (LIFDI-MS) was measured directly from an inert atmosphere glovebox with a Thermo Fisher Scientific Exactive Plus Orbitrap equipped with an ion source from Linden CMS.[2] Compounds ^{DippMe}NHI(TMS) (^{DippMe}NHI(TMS) = 1,3-bis (2,6-diisopropylphenyl)-4,5-dimethyl-N-(trimethylsilyl)-1,3-dihydro-2H-imidazol-2-imine) and $B_2Cl_4(SMe_2)_2$ were synthesized according to literature.[3-5]

1.2 Synthesis and characterization

1.2.1 Synthesis of compound 1

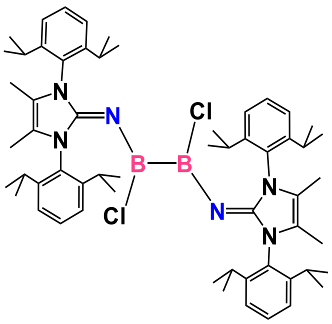

Dry ^{DippMe}NHI(TMS) (2.00 g, 3.969 mmol) was dissolved in 40 mL of toluene in a 100 mL Schlenk flask while $B_2Cl_4(SMe_2)_2$ (0.57 g, 1.985 mmol) was dissolved in 120 mL of toluene in a 200 mL pressure Schlenk flask. The former solution was added dropwise to the $B_2Cl_4(SMe_2)_2$ solution at room temperature. Then the mixture warmed up to 100 °C and stirred for 3 days. Afterwards, it cooled down to room temperature followed by filtration to separate the small amounts of insoluble substances. The solvent and volatile byproducts of the filtrate were removed in vacuo under 60 °C for 5 h. Then the pale-yellow solid was washed with 15 mL of pentane three times. The residual solvent was removed in vacuo. 1.45 g off-white solid was obtained, yield: 76.7%.

^{1}H NMR (400 MHz, C_6D_6, 298 K): δ [ppm] 7.21-7.17 (m, 4H, Ar^{Dipp}-*H*), 7.07 (d, *J* = 7.6 Hz, 8H, Ar^{Dipp}-*H*), 3.12 (sept, *J* = 6.7 Hz, 8H, C*H*(CH_3)$_2$), 1.48 (s, 12H, C*H*$_3$C=CC*H*$_3$), 1.34 (d, *J* = 6.8 Hz, 24H, CH(C*H*$_3$)$_2$), 1.12 (d, *J* = 7.0 Hz, 24H, CH(C*H*$_3$)$_2$).

^{13}C{^{1}H} NMR (101 MHz, C_6D_6, 298 K): δ [ppm] 148.11 (Ar^{Dipp}-*C*), 143.37 (N*C*N), 131.42 (Ar^{Dipp}-*C*), 129.64 (Ar^{Dipp}-*C*), 124.18 (Ar^{Dipp}-*C*), 117.36 (CH_3*C*=*C*CH_3), 28.82 (*C*H(CH_3)$_2$), 24.83 (CH(*C*H$_3$)$_2$), 24.21 (CH(*C*H$_3$)$_2$), 9.66 (*C*H$_3$C=C*C*H$_3$).

^{11}B NMR (128 MHz, C_6D_6, 298 K): δ [ppm] 28.11 (s, br).

LIFDI-MS: *m*/*z* for $C_{58}H_{80}B_2Cl_2N_6$, Calcd: 952.6008; Found: 952.6004.

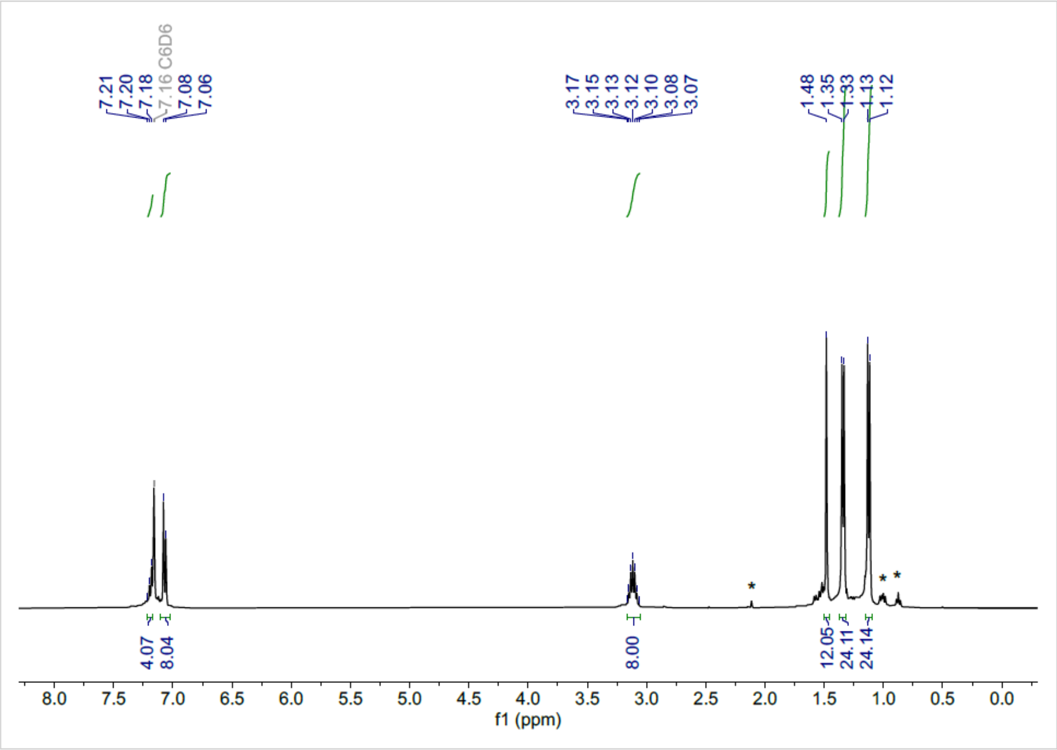

Figure S1. ^{1}H NMR spectrum of compound **1** (400 MHz, C_6D_6, 298 K). * Corresponds to residual peaks of pentane, toluene and H grease.

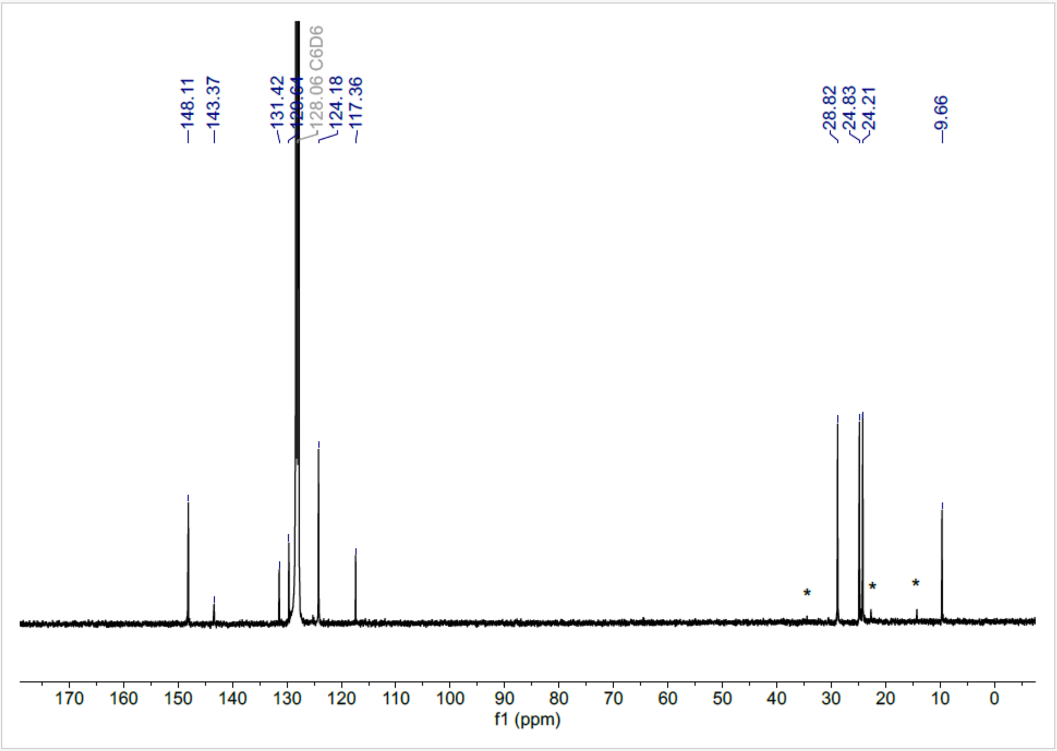

Figure S2. $^{13}C\{^{1}H\}$ NMR spectrum of compound **1** (101 MHz, C_6D_6, 298 K). * Corresponds to residual peaks of pentane.

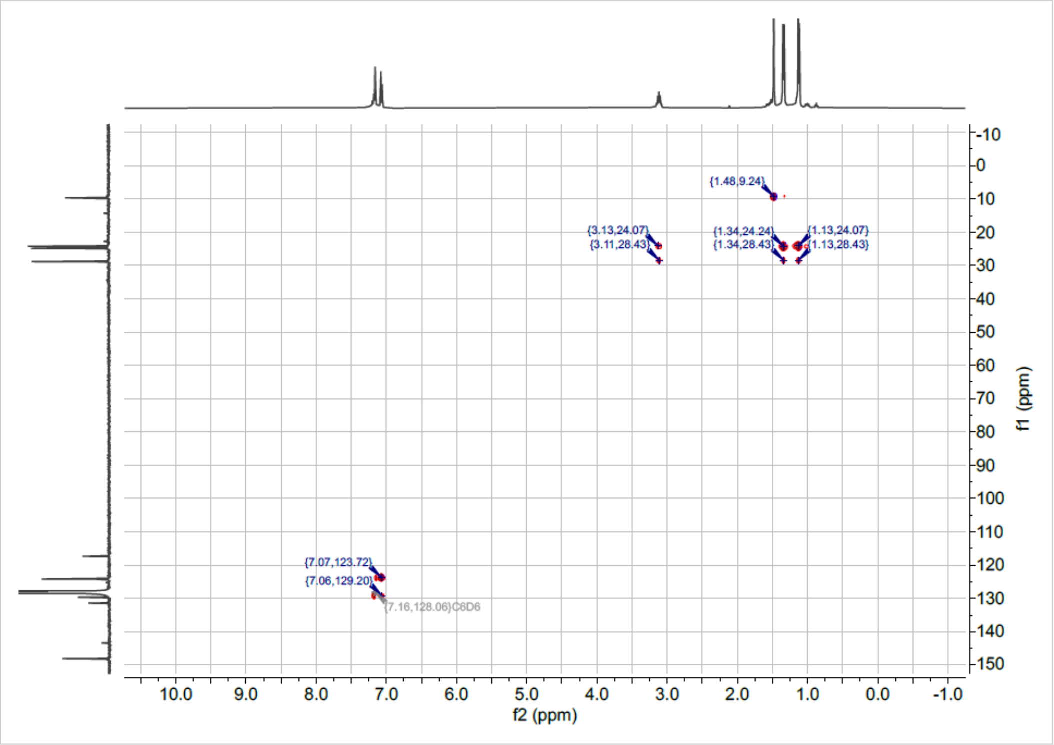

Figure S3. $^{1}H/^{13}C$ HSQC NMR spectrum of compound **1** (C_6D_6, 298 K).

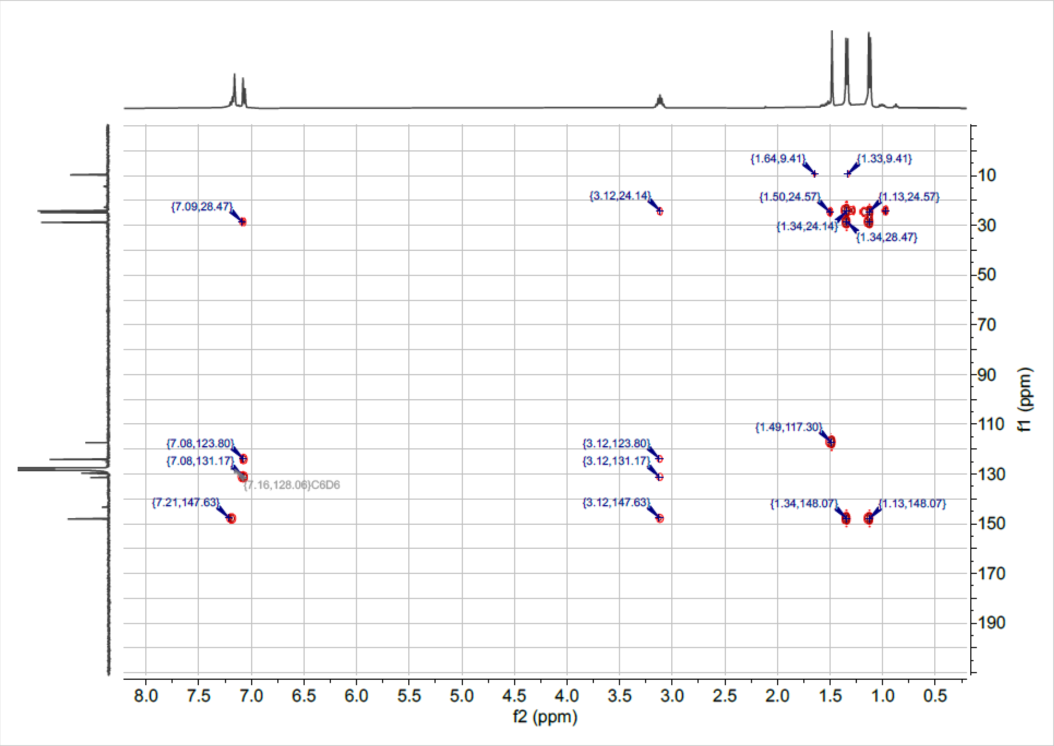

Figure S4. $^{1}H/^{13}C$ HMBC NMR spectrum of compound **1** (C_6D_6, 298 K).

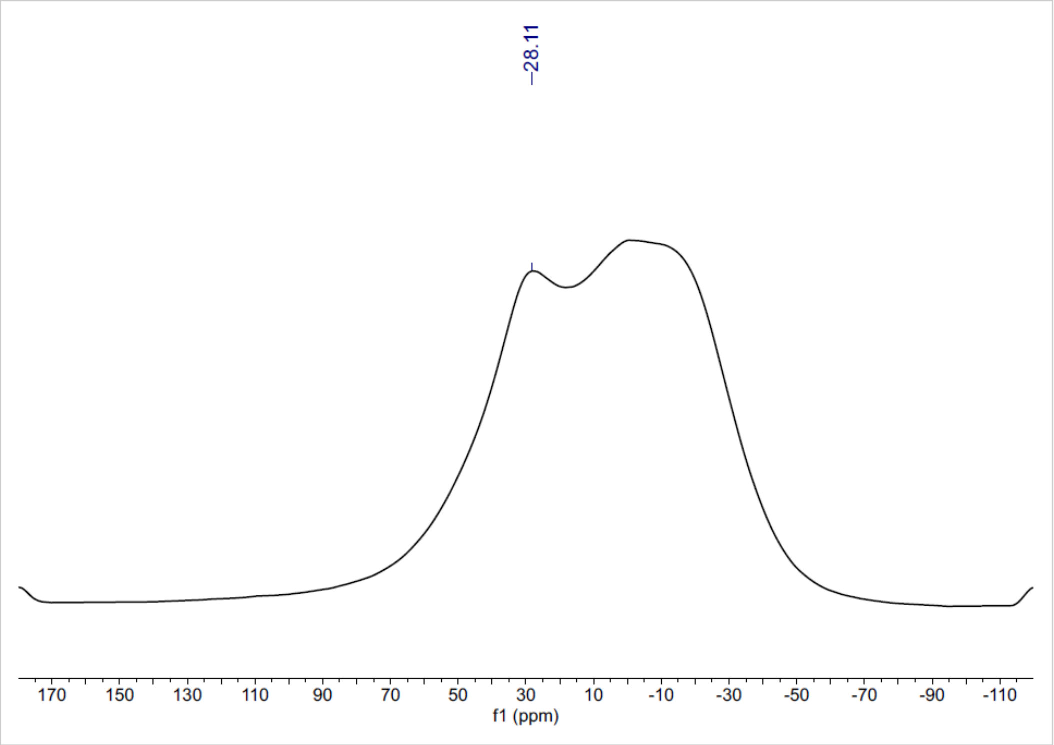

Figure S5. ^{11}B NMR spectrum of compound **1** (128 MHz, C_6D_6, 298 K).

1.2.2 Synthesis of compound 2

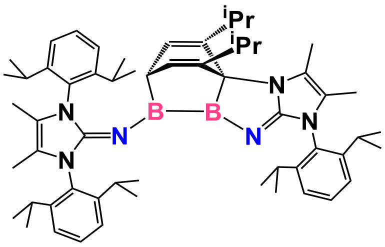

Compound **1** (400 mg, 0.4192 mmol) and sodium metal (21.2 mg, 0.9223 mmol) were put into a 100 mL pressure Schlenk flask, 40 mL of THF was transferred to the flask, after stirring vigorously at room temperature for 3 days, the mixture was filtered with a syringe filter to remove the sodium chloride and the residual sodium, obtaining an orange filtrate. Then the solvent was removed in vacuo, the oil was extracted with 40 mL of pentane, followed by removing the solvent from filtrate in vacuo, the extraction was repeated five times, obtaining 175.0 mg (47.3%) orange-yellow solid. Pale-yellow crystals suitable for X-ray diffraction analysis were obtained from a penta-fluorobenzene/pentane solution at –30 °C for a week.

^{1}H NMR (400 MHz, C_6D_6, 298 K): δ (ppm) 7.27-7.17 (m, 3H, Ar^{Dipp}-*H*), 7.13 (d, *J* = 8.4 Hz, 6H, Ar^{Dipp}-*H*), 5.62 (d, *J* = 6.7 Hz, 2H, BCHC*H*), 3.26 (sept, *J* = 6.6 Hz, 4H, C*H*(CH_3)$_2$), 2.94-2.84 (m, 2H, C*H*(CH_3)$_2$), 2.82 (t, *J* = 6.8 Hz, 1H, BC*H*CH), 2.27 (sept, *J* = 6.6 Hz, 2H, C*H*(CH_3)$_2$), 1.98 (s, 3H, C*H*$_3$C=CCH$_3$), 1.57 (s, 6H, C*H*$_3$C=CC*H*$_3$), 1.52 (s, 3H, CH$_3$C=CC*H*$_3$), 1.50 (d, *J* = 6.7 Hz, 12H, CH(C*H*$_3$)$_2$), 1.33 (d, *J* = 6.6 Hz, 6H, CH(C*H*$_3$)$_2$), 1.20 (d, *J* = 6.7 Hz, 12H, CH(C*H*$_3$)$_2$), 1.17 (d, *J* = 7.1 Hz, 6H, CH(C*H*$_3$)$_2$), 1.14 (d, *J* = 6.7 Hz, 6H, CH(C*H*$_3$)$_2$), 0.62 (d, *J* = 6.4 Hz, 6H, CH(C*H*$_3$)$_2$).

^{13}C{^{1}H} NMR (101 MHz, C_6D_6, 298 K): δ [ppm] 171.34 (N*C*N), 149.20 (Ar^{Dipp}-*C*), 147.79 (Ar^{Dipp}-*C*), 140.02 (N*C*N), 139.52 (CH*C*(^{i}Pr)), 132.34 (Ar^{Dipp}-*C*), 131.90 (Ar^{Dipp}-*C*), 129.68 (Ar^{Dipp}-*C*), 129.11 (Ar^{Dipp}-*C*), 123.63 (Ar^{Dipp}-*C*), 119.28 (CH$_3$*C*=CCH$_3$), 118.81 (CH*C*HC), 118.42 (CH$_3$C=*C*CH$_3$), 115.55 (CH$_3$*C*=*C*CH$_3$), 80.65 (B*C*N), 45.08 (B*C*HCH), 29.10 (*C*H(CH$_3$)$_2$), 28.99 (*C*H(CH$_3$)$_2$), 28.88 (*C*H(CH$_3$)$_2$), 25.36 (CH(*C*H$_3$)$_2$), 25.21 (CH(*C*H$_3$)$_2$), 23.97 (CH(*C*H$_3$)$_2$), 22.80 (CH(*C*H$_3$)$_2$), 10.82 (*C*H$_3$C=C*C*H$_3$), 9.80 (*C*H$_3$C=CCH$_3$), 9.43 (CH$_3$C=C*C*H$_3$).

^{11}B NMR (128 MHz, C_6D_6, 298 K): δ [ppm] 30.13 (s, br), 57.90 (s, br).

LIFDI-MS: *m*/*z* for $C_{58}H_{80}B_2N_6$, Calcd: 882.6631; Found: 882.6670.

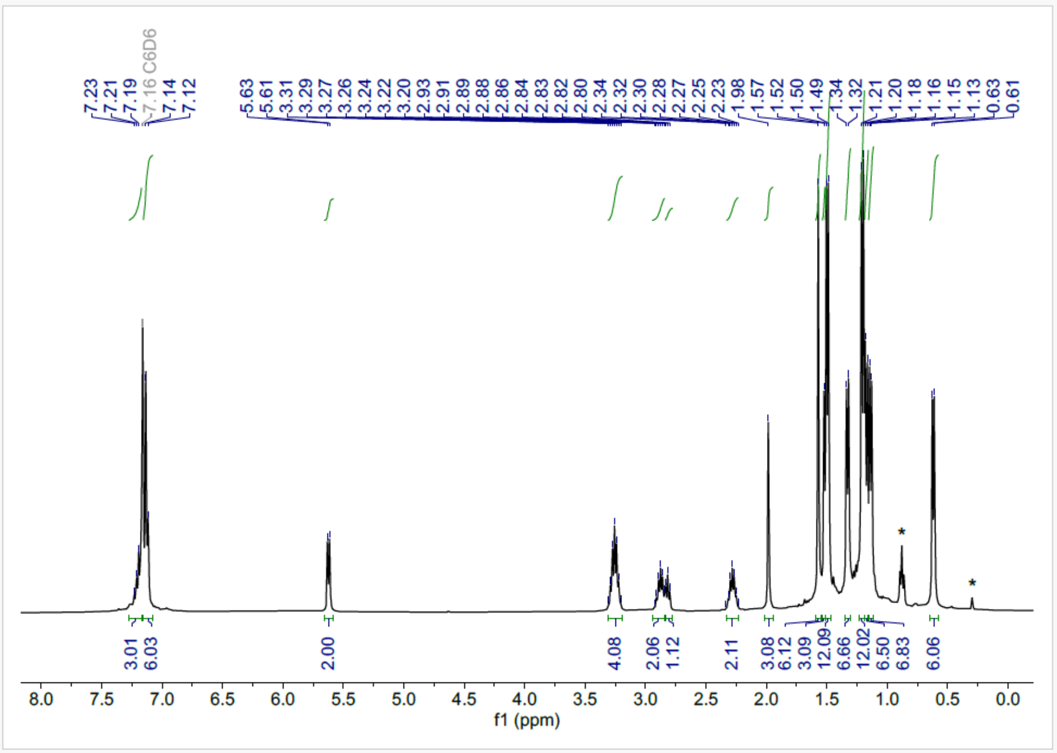

Figure S6. ^{1}H NMR spectrum of compound **2** (400 MHz, C_6D_6, 298 K). * Corresponds to residual peaks of pentane and silicone grease.

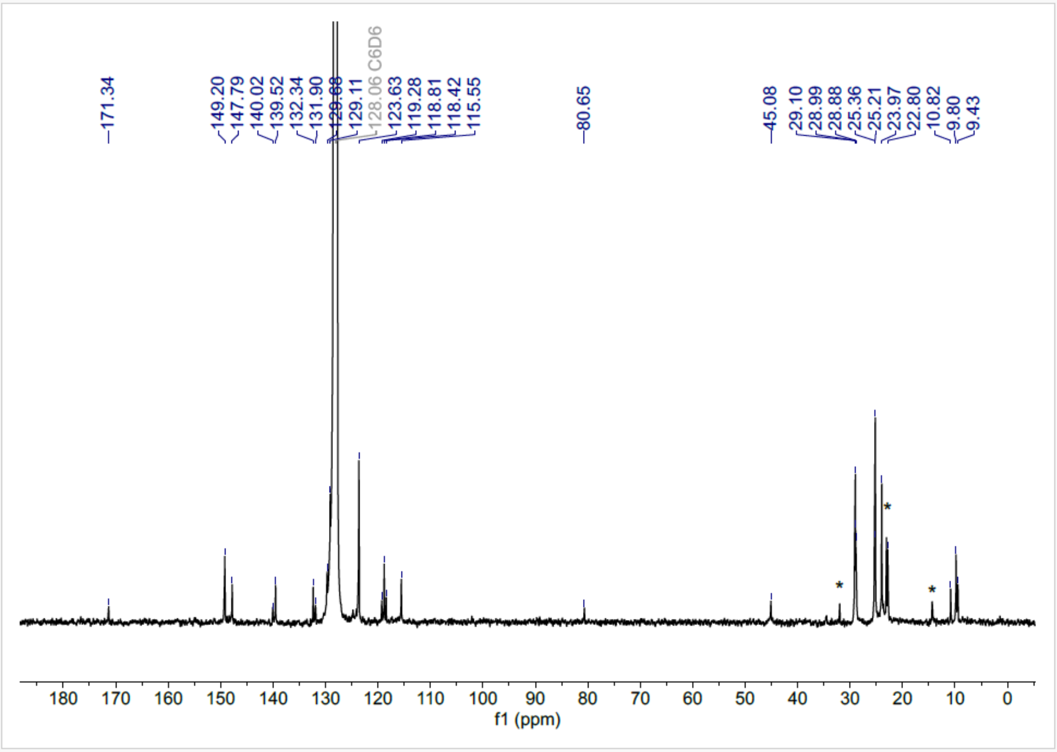

Figure S7. $^{13}C\{^{1}H\}$ NMR spectrum of compound **2** (101 MHz, C_6D_6, 298 K). * Corresponds to residual peaks of pentane.

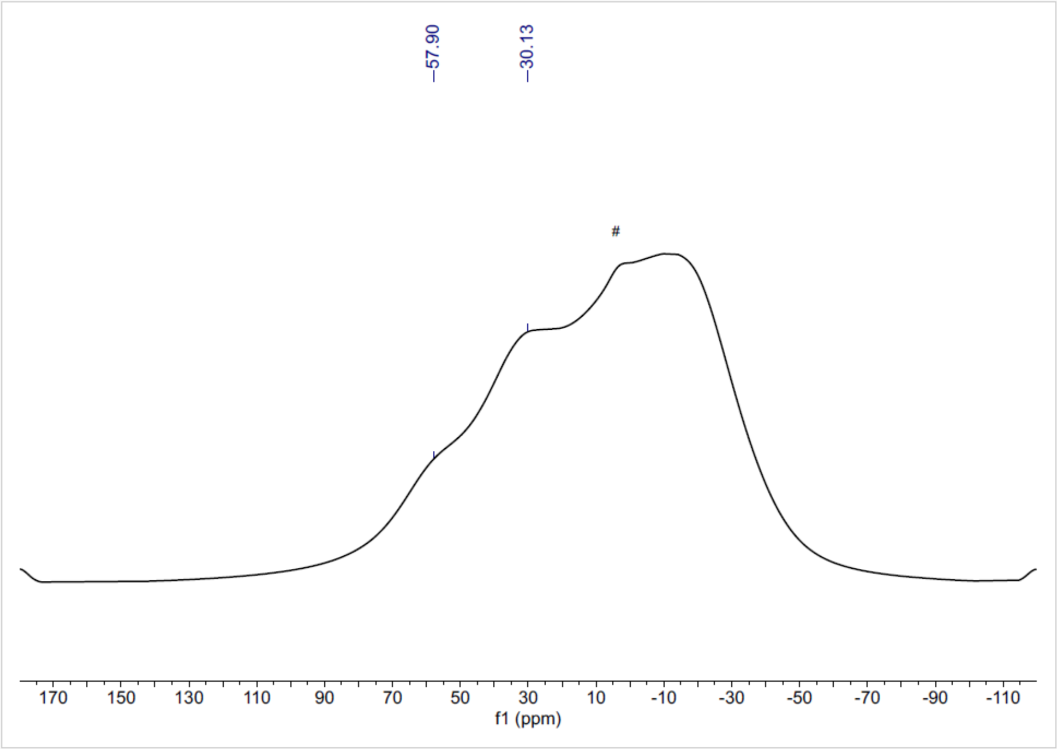

Figure S8. ^{11}B NMR spectrum of compound **2** (128 MHz, C_6D_6, 298 K). # Corresponds to trace amount of impurity.

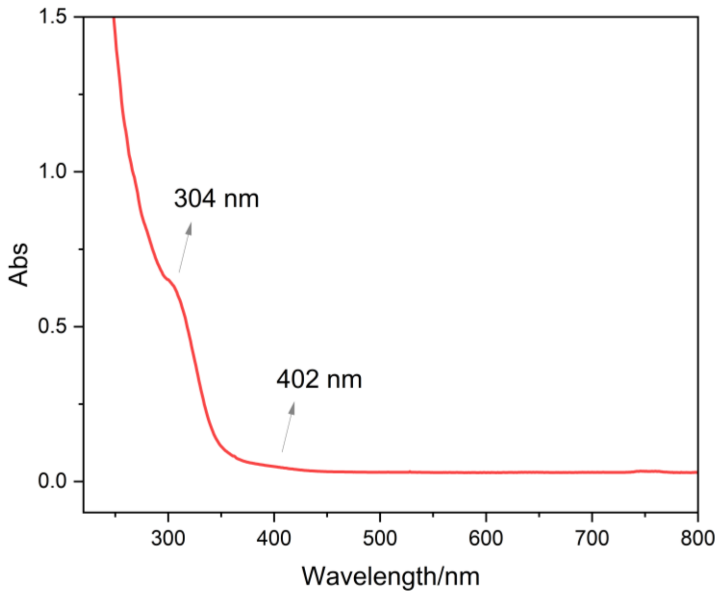

Figure S9. UV-Vis spectrum of compound **2** (hexane, 298 K).

1.2.3 Synthesis of compound 3

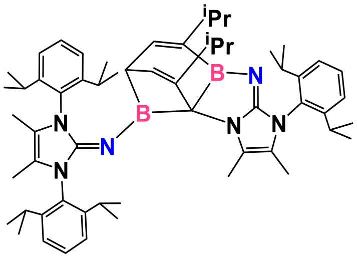

Compound **2** (40 mg, 0.0452 mmol) was dissolved in 2 mL of THF in a 10 mL pressure Schlenk flask at room temperature, then heated to 80 °C and stirred for 2 days. After removal of the solvent, the resulting solid was washed three times with 3 mL of pentane, and the residual solvent was evaporated to afford 3.4 mg of a white solid. The supernatant was subjected to the same drying and washing procedure three times, and all the resulting white precipitate was collected to give 10.2 mg of product (25.5%). (Note: compound **3**, compound **2** and a small amount of byproduct have quite similar solubility in common solvents). Colorless crystals suitable for X-ray diffraction analysis were obtained from a penta-fluorobenzene solution at –30 °C for a week.

^{1}H NMR (400 MHz, C_6D_6, 298 K): δ (ppm) 7.28-7.22 (m, 3H, Ar^{Dipp}-*H*), 7.22-7.18 (m, 2H, Ar^{Dipp}-*H*), 7.15-7.09 (m, 4H, Ar^{Dipp}-*H*), 6.25 (d, *J* = 7.9 Hz, 1H, BC(^{i}Pr)=C*H*), 5.63 (d, *J* = 4.3 Hz, 1H, C*H*CHB),

3.06 (sept, J = 6.8 Hz, 1H, C*H*(CH_3)$_2$), 2.98-2.82 (m, 4H, C*H*(CH_3)$_2$), 2.82-2.71 (m, 2H, C*H*(CH_3)$_2$), 2.29 (dd, J = 7.9, 4.3 Hz, 1H, BC*H*CH), 2.20 (sept, J = 6.9 Hz, 1H, C*H*(CH_3)$_2$), 1.68 (s, 3H, CH_3C=CCH$_3$), 1.45 (s, 3H, CH$_3$C=CCH_3), 1.44 (s, 6H, CH_3C=CCH_3), 1.43 (d, J = 2.1 Hz, 3H, CH(CH_3)$_2$), 1.36-1.29 (m, 24H, CH(CH_3)$_2$), 1.15 (dd, J = 7.0, 3.7 Hz, 12H, CH(CH_3)$_2$), 1.11 (d, J = 7.1 Hz, 6H, CH(CH_3)$_2$), 0.77 (d, J = 6.7 Hz, 3H, CH(CH_3)$_2$).

$^{13}C\{^1H\}$ NMR (101 MHz, C_6D_6, 298 K): δ [ppm] 166.82 (N*C*N), 153.60 (B*C*(^{i}Pr)), 148.64 (N*C*N), 148.36 (Ar^{Dipp}-*C*), 148.07 (C*C*(^{i}Pr)), 147.44 (Ar^{Dipp}-*C*), 146.09 (Ar^{Dipp}-*C*), 144.85 (BC(^{i}Pr)=*C*H), 132.57 (Ar^{Dipp}-*C*), 131.94 (Ar^{Dipp}-*C*), 129.90 (Ar^{Dipp}-*C*), 129.42 (Ar^{Dipp}-*C*), 124.77 (Ar^{Dipp}-*C*), 124.09 (Ar^{Dipp}-*C*), 123.87 (Ar^{Dipp}-*C*), 119.82 (CH$_3$*C*=*C*CH$_3$), 117.31 (CH$_3$*C*=*C*CH$_3$), 116.41 (CH$_3$*C*=*C*CH$_3$), 116.14 (CC(^{i}Pr)=*C*H), 39.78 (B*C*HCH), 29.16 (*C*H(CH$_3$)$_2$), 29.11 (*C*H(CH$_3$)$_2$), 28.74 (*C*H(CH$_3$)$_2$), 28.60 (*C*H(CH$_3$)$_2$), 25.85 (CH(*C*H$_3$)$_2$), 25.48 (CH(*C*H$_3$)$_2$), 24.83 (CH(*C*H$_3$)$_2$), 24.50 (CH(*C*H$_3$)$_2$), 24.26 (CH(*C*H$_3$)$_2$), 24.14 (CH(*C*H$_3$)$_2$), 23.74 (CH(*C*H$_3$)$_2$), 23.66 (CH(*C*H$_3$)$_2$), 23.46 (CH(*C*H$_3$)$_2$), 22.65 (CH(*C*H$_3$)$_2$), 20.53 (CH(*C*H$_3$)$_2$), 9.60 (*C*H$_3$C=C*C*H$_3$), 9.42 (*C*H$_3$C=CCH$_3$), 8.56 (CH$_3$C=C*C*H$_3$).

^{11}B NMR (128 MHz, C_6D_6, 298 K): δ [ppm] 44.76 (s, br), 49.87 (s, br).

LIFDI-MS: *m*/*z* for $C_{58}H_{80}B_2N_6$, Calcd: 882.6631; Found: 882.6692.

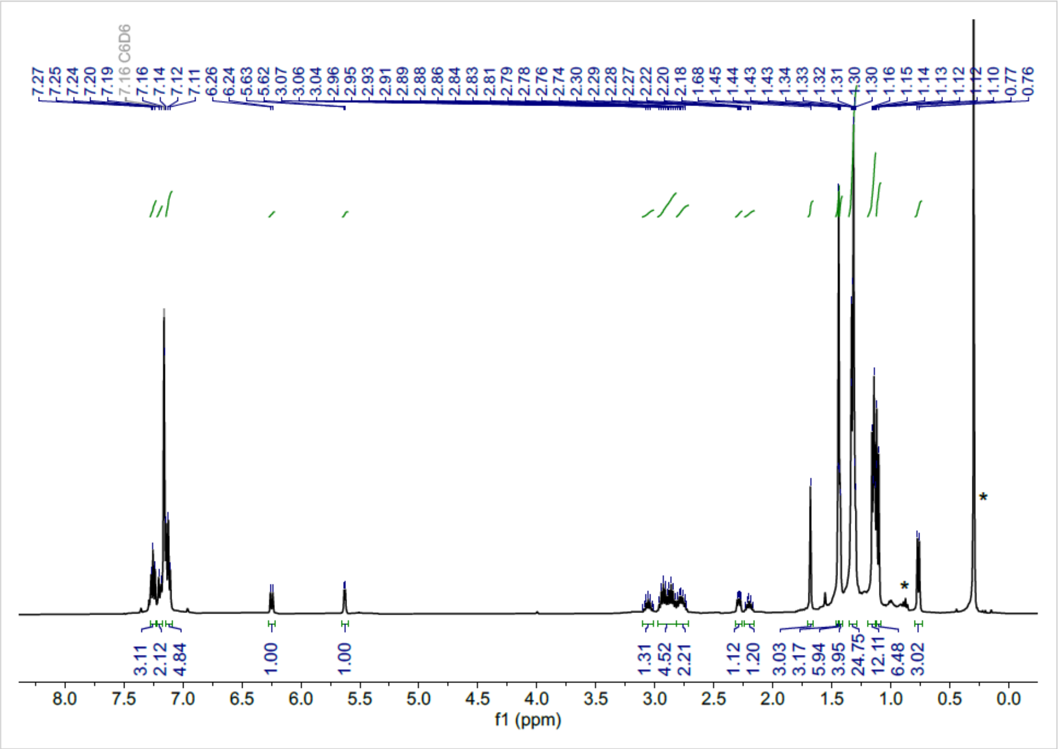

Figure S10. ^{1}H NMR spectrum of compound **3** (400 MHz, C_6D_6, 298 K). * Corresponds to residual peaks of pentane, hexane and silicone grease.

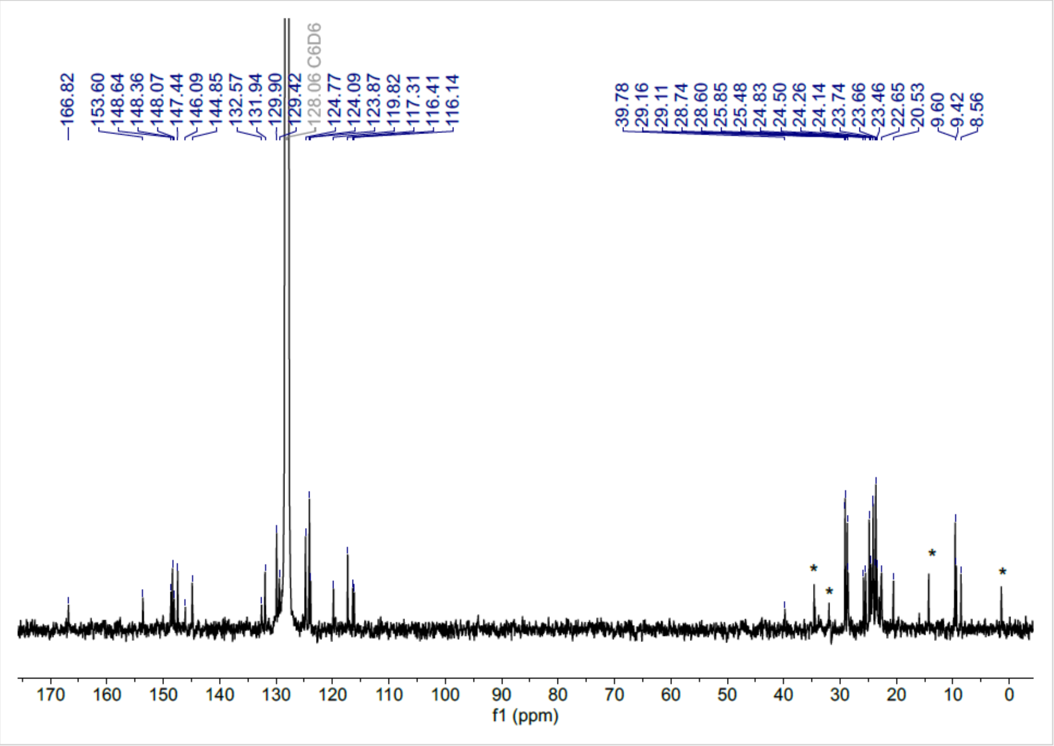

Figure S11. $^{13}C\{^{1}H\}$ NMR spectrum of compound **3** (101 MHz, C_6D_6, 298 K). * Corresponds to residual peaks of pentane, hexane and silicone grease.

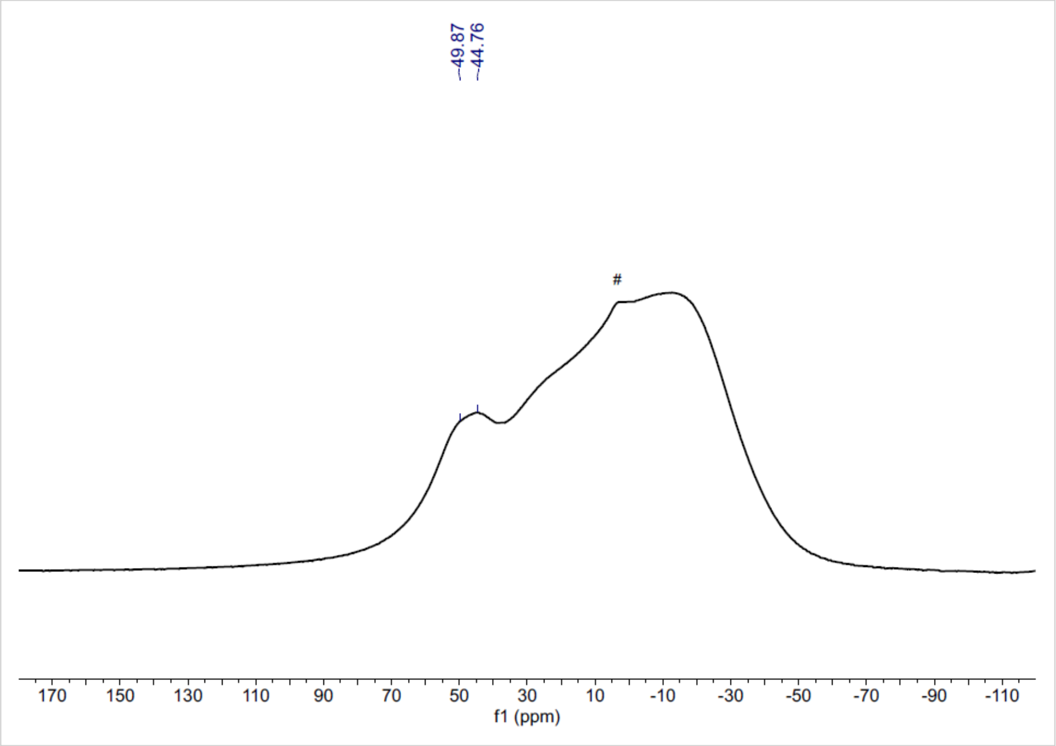

Figure S12. ^{11}B NMR spectrum of compound **3** (128 MHz, C_6D_6, 298 K). # Corresponds to trace amount of impurity.

1.2.4 Synthesis of compound 4

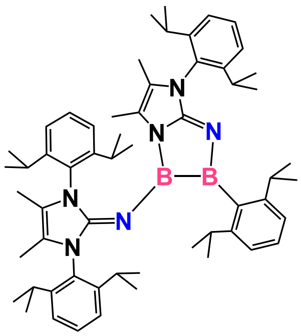

Method A: Compound **1** (400 mg, 0.4192 mmol) and sodium metal (21.2 mg, 0.9223 mmol) were placed in a 100 mL pressure Schlenk flask, and 40 mL of THF was added. The mixture was stirred vigorously at room temperature for 3 days, affording a green mixture. The mixture was then filtered through a syringe filter to remove the sodium chloride and the residual sodium, yielding an orange filtrate. The solvent was removed under reduced pressure, and the resulting oil was washed with 40 mL of pentane, and the precipitate was separated from the solution. The precipitate was then washed three times with 15 mL of pentane and dried under vacuum to afford 11.5 mg of bright-yellow solid **4**. Bright-yellow crystals suitable for X-ray diffraction analysis were obtained from a pentane solution at –30 °C for 3 days.

Method B: Compound **2** (20 mg, 0.0226 mmol) was dissolved in 0.4 mL of C_6D_6 in a J-Young NMR tube at room temperature and irradiated at 300 nm for 2 days, affording a deep orange solution. After removal of the solvent, the resulting orange solid was washed three times with 6 mL of pentane, and the residual solvent was evaporated under reduced pressure to give 18.2 mg (91.4%) of bright-yellow solid.

^{1}H NMR (400 MHz, C_6D_6, 298 K): δ (ppm) 7.27 (q, *J* = 7.5 Hz, 2H, Ar^{Dipp}-*H*), 7.20 (t, *J* = 7.8 Hz, 2H, Ar^{Dipp}-*H*), 7.14-7.06 (m, 8H, Ar^{Dipp}-*H*), 3.10 (sept, *J* = 6.9 Hz, 4H, C*H*(CH_3)$_2$), 3.05-2.90 (m, 4H, C*H*(CH_3)$_2$), 1.95 (s, 3H, CH_3C=CCH_3), 1.49 (s, 3H, CH_3C=CC*H*$_3$), 1.46 (s, 6H, C*H*$_3$C=CC*H*$_3$), 1.25 (d, *J* = 6.8 Hz, 6H, CH(C*H*$_3$)$_2$), 1.22 (d, *J* = 6.7 Hz, 6H, CH(C*H*$_3$)$_2$), 1.15 (d, *J* = 6.8 Hz, 6H, CH(C*H*$_3$)$_2$), 1.13-1.09 (m, 24H, CH(C*H*$_3$)$_2$), 1.08 (d, *J* = 6.6 Hz, 6H, CH(C*H*$_3$)$_2$).

^{13}C{^{1}H} NMR (101 MHz, C_6D_6, 298 K): δ [ppm] 176.32 (N*C*N), 149.53 (Ar^{Dipp}-*C*), 148.21 (Ar^{Dipp}-*C*), 147.84 (Ar^{Dipp}-*C*), 143.32 (N*C*N), 132.28 (Ar^{Dipp}-*C*), 132.07 (Ar^{Dipp}-*C*), 129.65 (Ar^{Dipp}-*C*), 129.45 (Ar^{Dipp}-*C*), 126.15 (Ar^{Dipp}-*C*), 124.61 (Ar^{Dipp}-*C*), 123.74 (Ar^{Dipp}-*C*), 120.85 (Ar^{Dipp}-*C*), 119.54

(CH_3C=CCH_3), 119.07 (CH_3C=CCH_3), 117.90 (CH_3C=CCH_3), 33.61 ($CH(CH_3)_2$), 29.02 ($CH(CH_3)_2$), 28.71 ($CH(CH_3)_2$), 26.14 ($CH(CH_3)_2$), 24.65 ($CH(CH_3)_2$), 24.62 ($CH(CH_3)_2$), 24.52 ($CH(CH_3)_2$), 23.81 ($CH(CH_3)_2$), 23.30 ($CH(CH_3)_2$), 10.65 (CH_3C=CCH_3), 10.08 (CH_3C=CCH_3), 8.78 (CH_3C=CCH_3).

^{11}B NMR (128 MHz, C_6D_6, 298 K): δ [ppm] 27.84 (s, br), 63.70 (s, br).

LIFDI-MS: *m*/*z* for $C_{58}H_{80}B_2N_6$, Calcd: 882.6631; Found: 882.6715.

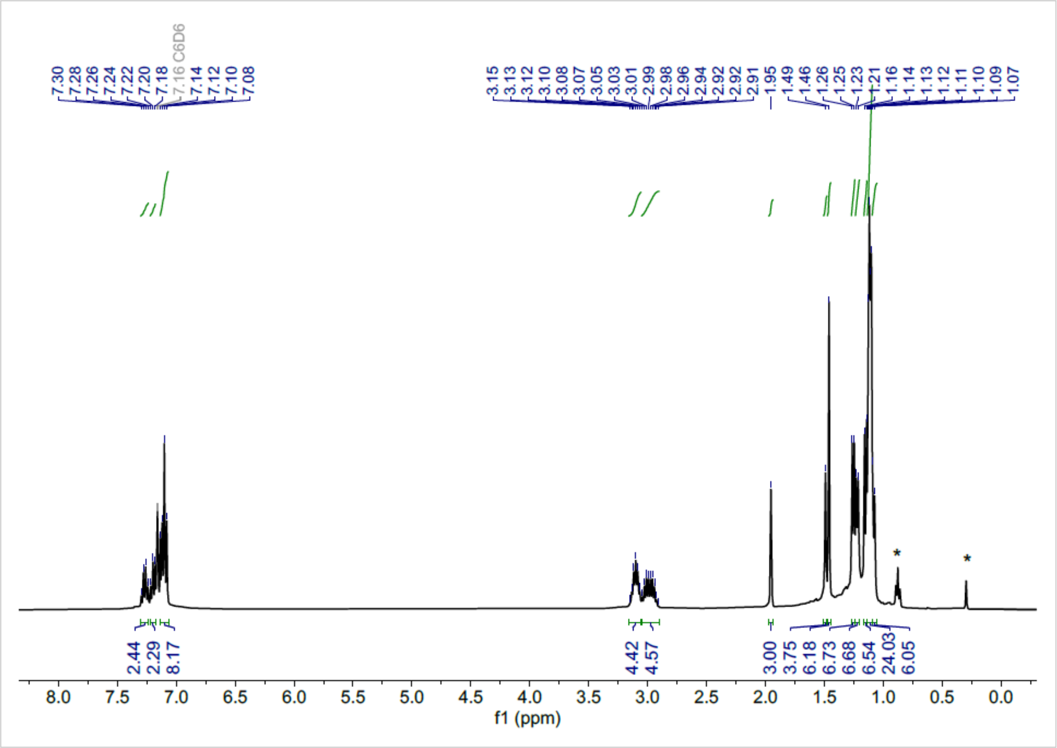

Figure S13. ^{1}H NMR spectrum of compound **4** (400 MHz, C_6D_6, 298 K). * Corresponds to residual peaks of pentane and silicone grease.

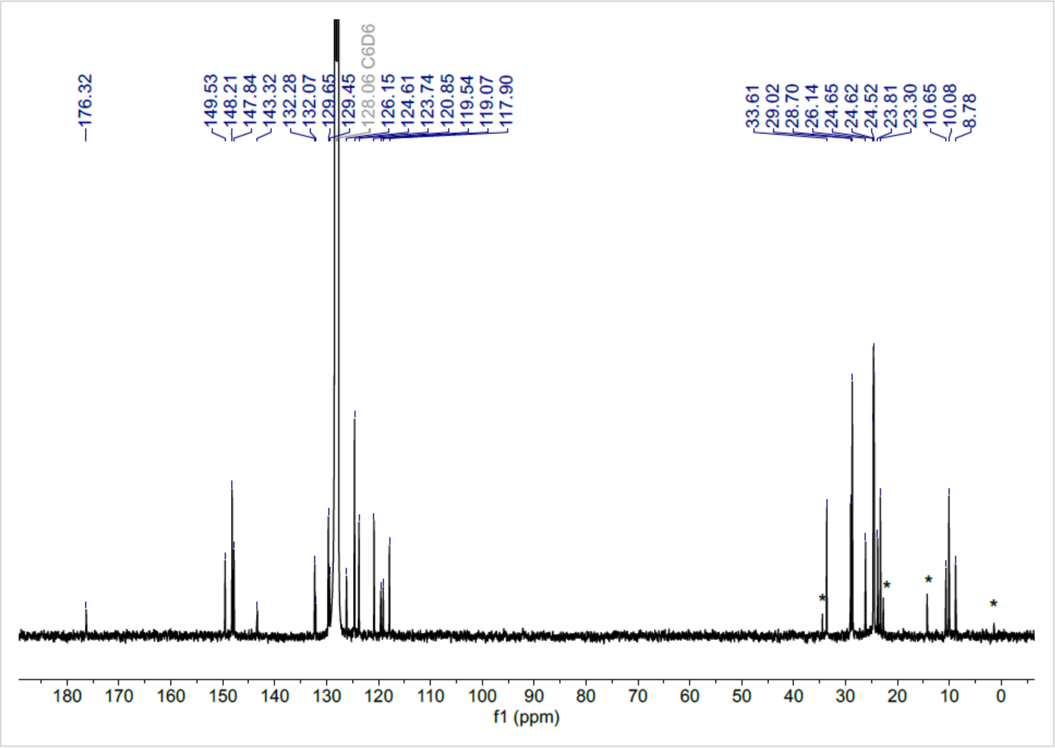

Figure S14. $^{13}C\{^{1}H\}$ NMR spectrum of compound **4** (101 MHz, C_6D_6, 298 K). * Corresponds to residual peaks of pentane and silicone grease.

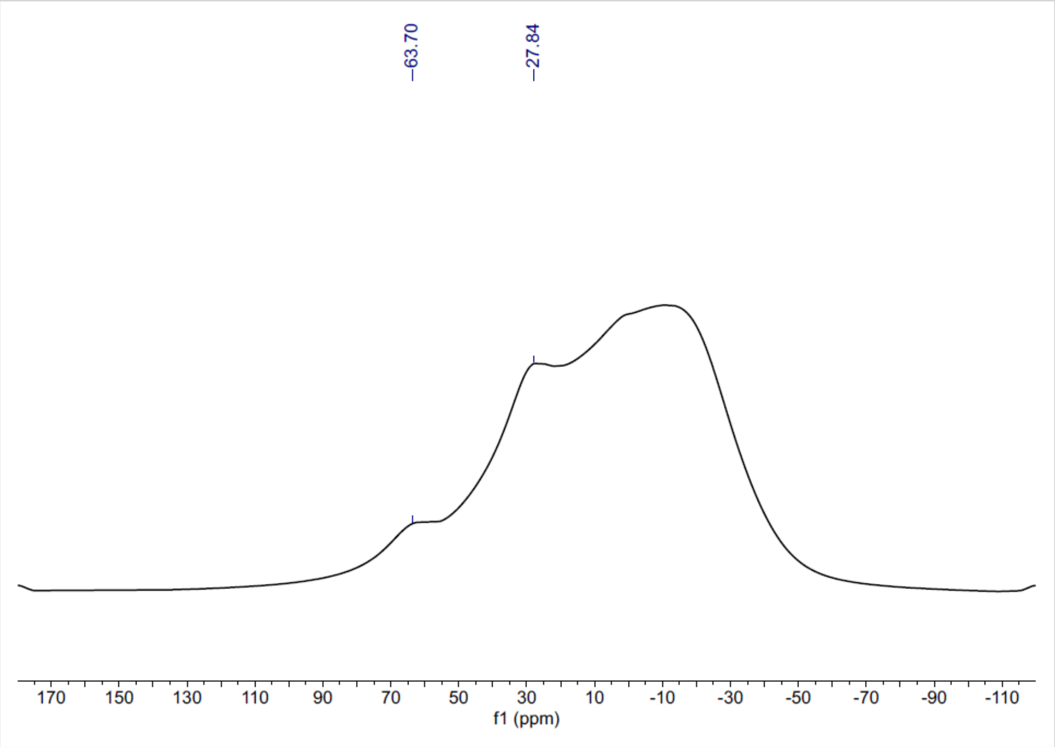

Figure S15. ^{11}B NMR spectrum of compound **4** (128 MHz, C_6D_6, 298 K).

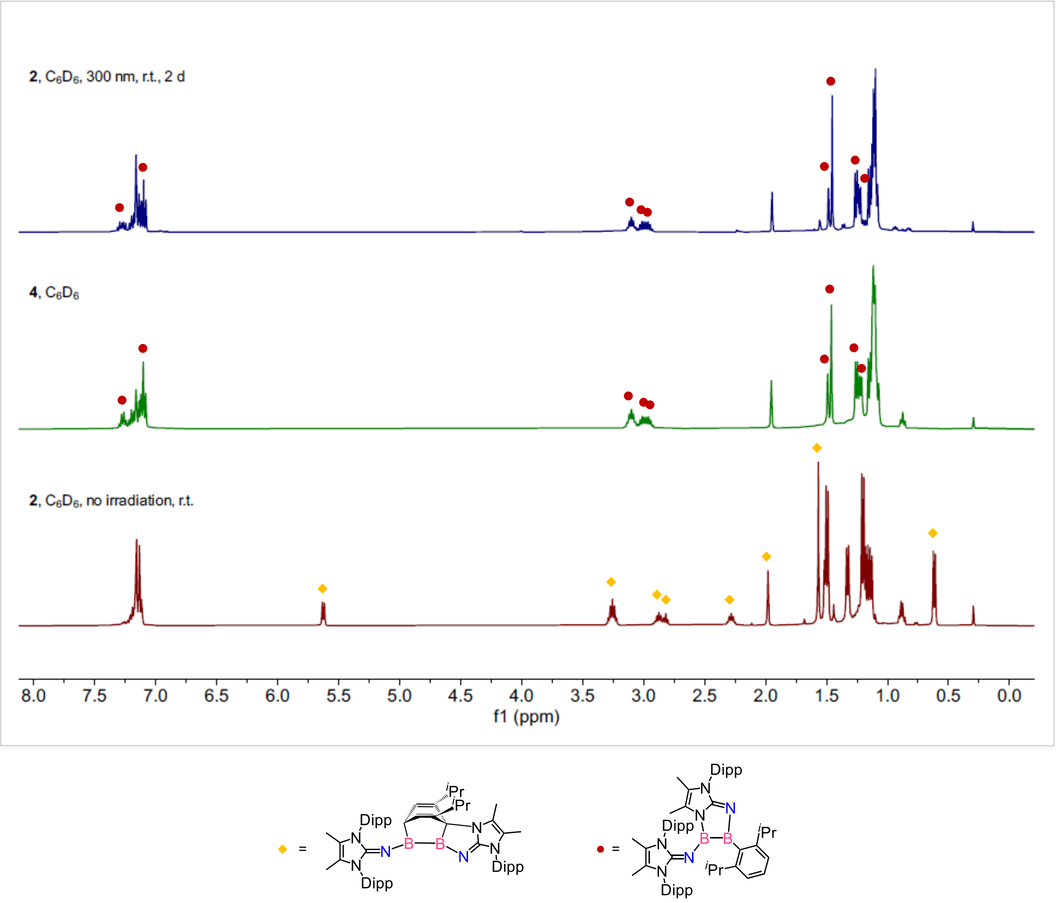

Figure S16 Stacked ^{1}H NMR spectra for the reaction of **2** in C_6D_6 in the absence of irradiation and under 300 nm irradiation for 2 d (400 MHz, C_6D_6, 298 K).

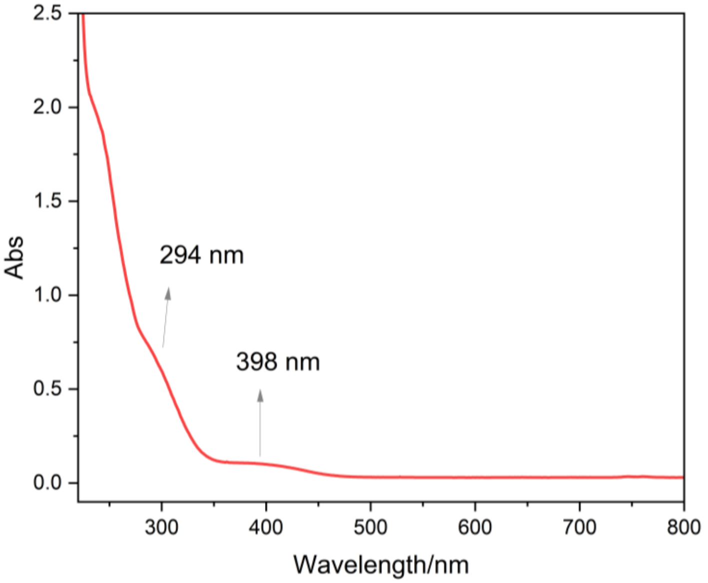

Figure S17. UV-Vis spectrum of compound **4** (hexane, 298 K).

1.2.5 Synthesis of compound 5-S^{Me}

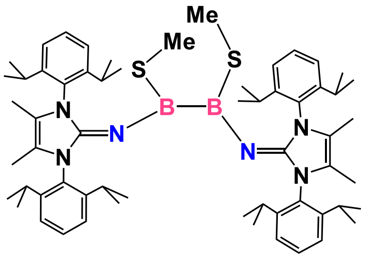

Compound **2** (40 mg, 0.0452 mmol) and 1,2-dimethyldisulfane (4.0 µL, 0.0452 mmol) were dissolved in 1.0 mL of benzene in a Schlenk tube at room temperature. The solution was irradiated at 419 nm for 4 hours, affording a pale-orange solution. Upon completion of the reaction, the solvent was removed under reduced pressure, and the residue was dissolved in 0.2 mL of pentane. The solution was filtered and stored at –30 °C in a glovebox for 2 days, yielding 18.8 mg (42.5%) colorless crystals suitable for X-ray diffraction analysis.

^{1}H NMR (400 MHz, C_6D_6, 298 K): δ [ppm] 7.22-7.18 (m, 4H, Ar^{Dipp}-*H*), 7.12 (d, *J* = 7.5 Hz, 8H, Ar^{Dipp}-*H*), 3.19 (sept, *J* = 6.8 Hz, 8H, C*H*$(CH_3)_2$), 1.48 (s, 12H, C*H*$_3$C=CC*H*$_3$), 1.31 (s, br, 24H, CH(C*H*$_3$)$_2$), 1.25 (s, 6H, SC*H*$_3$), 1.18 (d, *J* = 6.9 Hz, 24H, CH(C*H*$_3$)$_2$).

^{13}C{^{1}H} NMR (101 MHz, C_6D_6, 298 K): δ [ppm] 148.82 (Ar^{Dipp}-*C*), 145.05 (N*C*N), 132.63 (Ar^{Dipp}-*C*), 129.28 (Ar^{Dipp}-*C*), 124.22 (Ar^{Dipp}-*C*), 116.95 (CH_3*C*=*C*CH_3), 28.81 (*C*H$(CH_3)_2$), 25.34 (CH(*C*H$_3$)$_2$), 24.28 (CH(*C*H$_3$)$_2$), 9.97 (S*C*H$_3$), 9.87 (*C*H$_3$C=C*C*H$_3$).

^{11}B NMR (128 MHz, C_6D_6, 298 K): δ [ppm] 39.51(s, br).

LIFDI-MS: *m*/*z* for $C_{60}H_{86}B_2N_6S_2$, Calcd: 976.6550; Found: 976.6541.

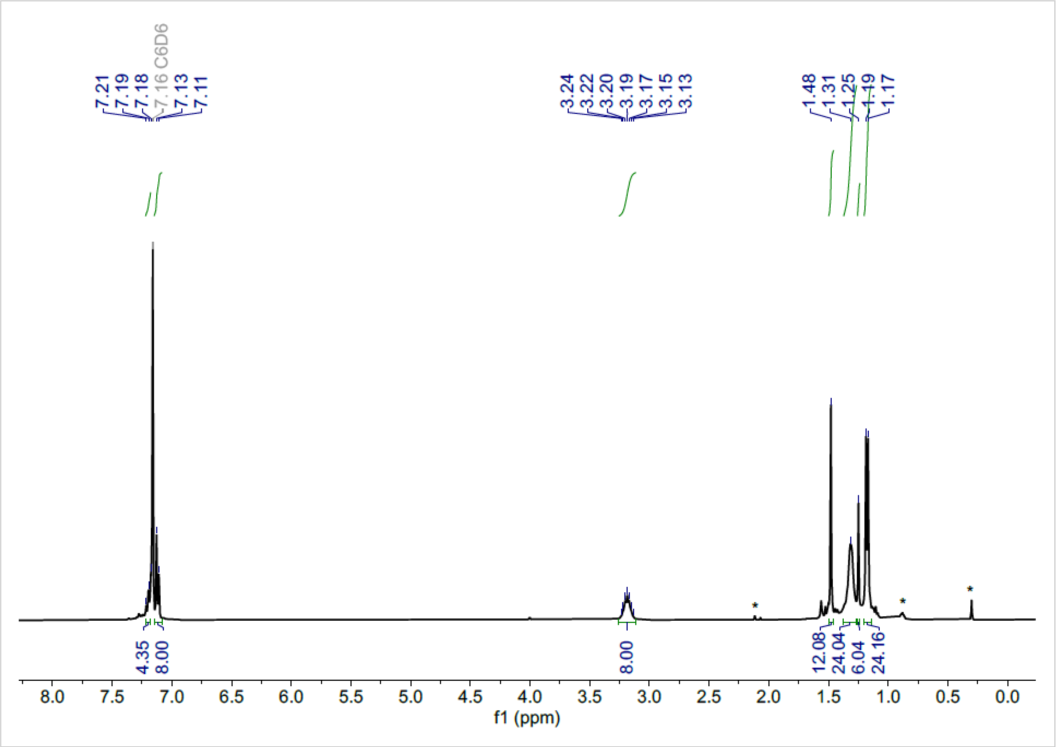

Figure S18. ^1H NMR spectrum of compound **5-S^{Me}** (400 MHz, C_6D_6, 298 K). * Corresponds to residual peaks of pentane, toluene and silicone grease.

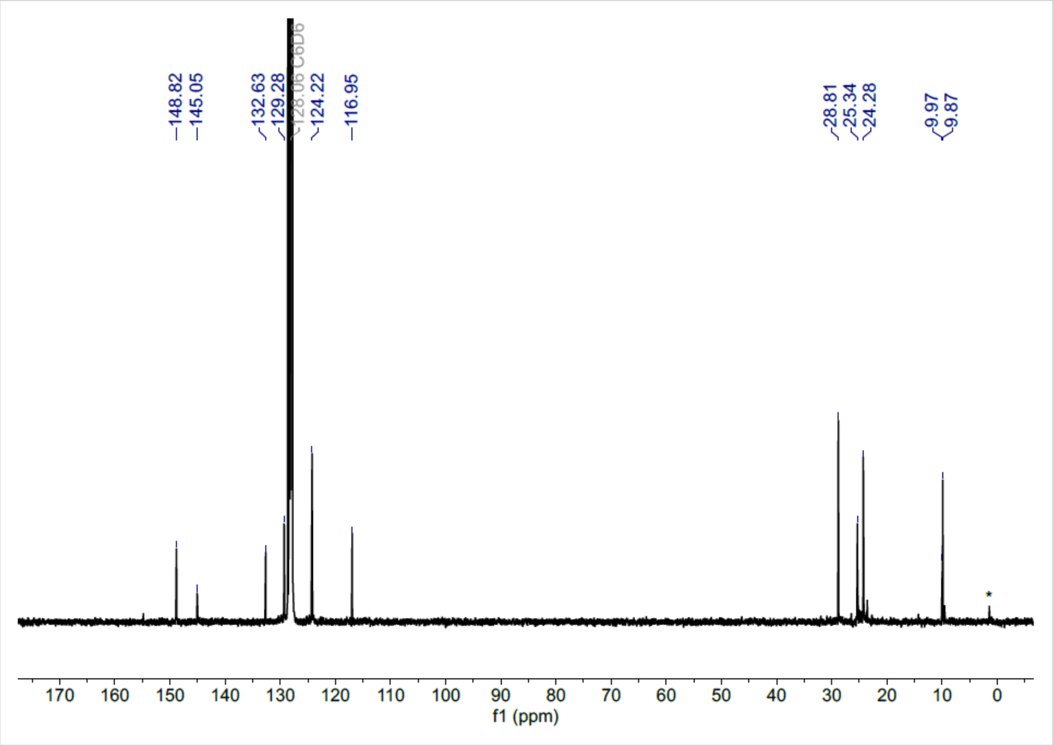

Figure S19. $^{13}C\{^1H\}$ NMR spectrum of compound **5-S^{Me}** (101 MHz, C_6D_6, 298 K). * Corresponds to residual peak of silicone grease.

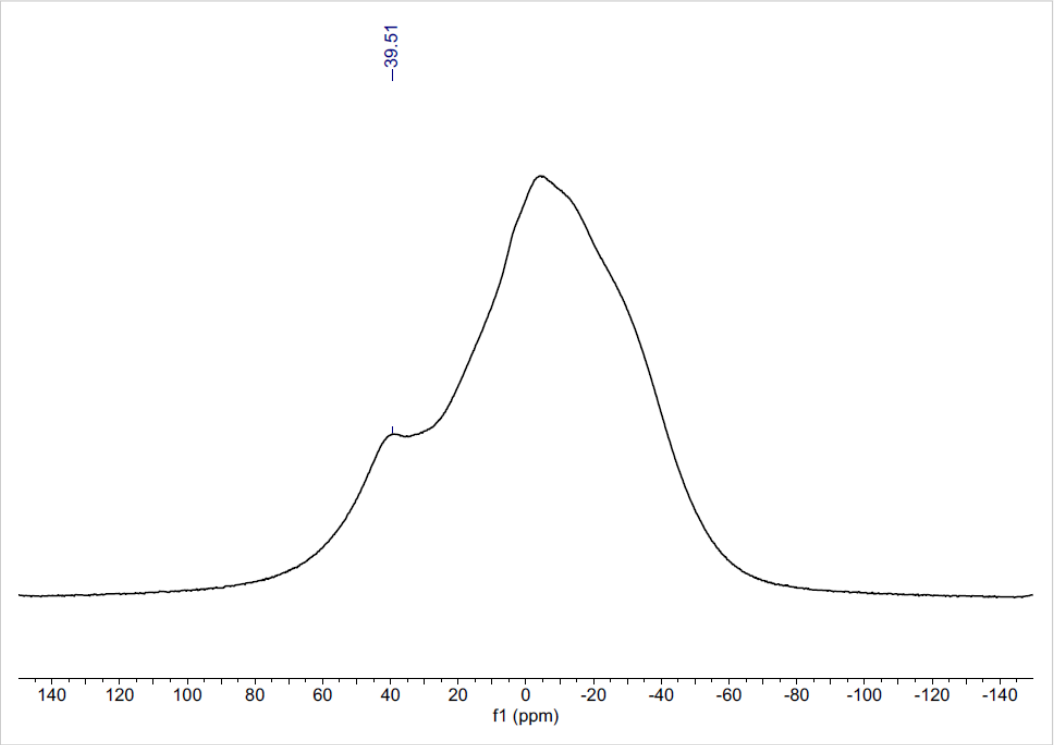

Figure S20. ^{11}B NMR spectrum of compound **5-S^{Me}** (128 MHz, C_6D_6, 298 K).

1.2.6 Synthesis of compound 5-Se^{Ph}

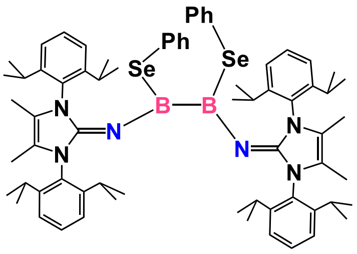

Compound **2** (20 mg, 0.0226 mmol) and 1,2-diphenyldiselenane (7.2 mg, 0.0226 mmol) were dissolved in 0.4 mL of C_6D_6 in a J-Young NMR tube at room temperature. The reaction mixture was monitored in situ by ^{1}H and ^{11}B NMR spectroscopy, and no new species was observed under ambient conditions after 2 days. The sample was then irradiated at 419 nm for 30 min, affording a pale-yellow solution. Upon completion of the reaction, the solvent was removed under reduced pressure. The resulting residue was extracted with 0.4 mL of C_6D_6, and the NMR spectra were recorded directly. (Note: Further purification with common solvents i.e. toluene, benzene, pentane, hexane and THF resulted in decomposition of the product.)

^{1}H NMR (400 MHz, C_6D_6, 298 K): δ [ppm] 7.54-7.48 (m, 2H, SePh-*H*), 7.27-7.23 (m, 4H, SePh-*H*), 7.20 (t, *J* = 7.8 Hz, 4H, Ar^{Dipp}-*H*), 7.07 (d, *J* = 7.7 Hz, 8H, Ar^{Dipp}-*H*), 6.90-6.86 (m, 2H, SePh-*H*), 6.79-6.73 (m, 2H, SePh-*H*), 3.17 (sept, *J* = 6.8 Hz, 8H, C*H*(CH_3)$_2$), 1.41 (s, 12H, C*H*$_3$C=CC*H*$_3$), 1.22 (d, *J* = 6.7 Hz, 24H, CH(C*H*$_3$)$_2$), 1.13 (d, *J* = 6.9 Hz, 24H, CH(C*H*$_3$)$_2$).

^{13}C{^{1}H} NMR (101 MHz, C_6D_6, 298 K): δ [ppm] 148.50 (Ar^{Dipp}-*C*), 144.87 (N*C*N), 135.12 (Ar^{Ph}-*C*), 133.10 (Ar^{Ph}-*C*), 132.21 (Ar^{Dipp}-*C*), 129.81 (Ar^{Dipp}-*C*), 129.25 (Ar^{Ph}-*C*), 124.56 (Ar^{Dipp}-*C*), 123.81 (Ar^{Ph}-*C*), 117.99 (CH_3*C*=*C*CH_3), 28.79 (*C*H(CH_3)$_2$), 25.47 (CH(*C*H$_3$)$_2$), 24.50 (CH(*C*H$_3$)$_2$), 10.00 (*C*H$_3$C=C*C*H$_3$).

^{11}B NMR (128 MHz, C_6D_6, 298 K): δ [ppm] 30.79 (s, br).

^{77}Se NMR (76 MHz, C_6D_6, 298 K): δ [ppm] 290.46 (s).

LIFDI-MS: *m/z* for $C_{70}H_{90}B_2N_6Se_2$, Calcd: 1196.5744; Found: 1196.5684.

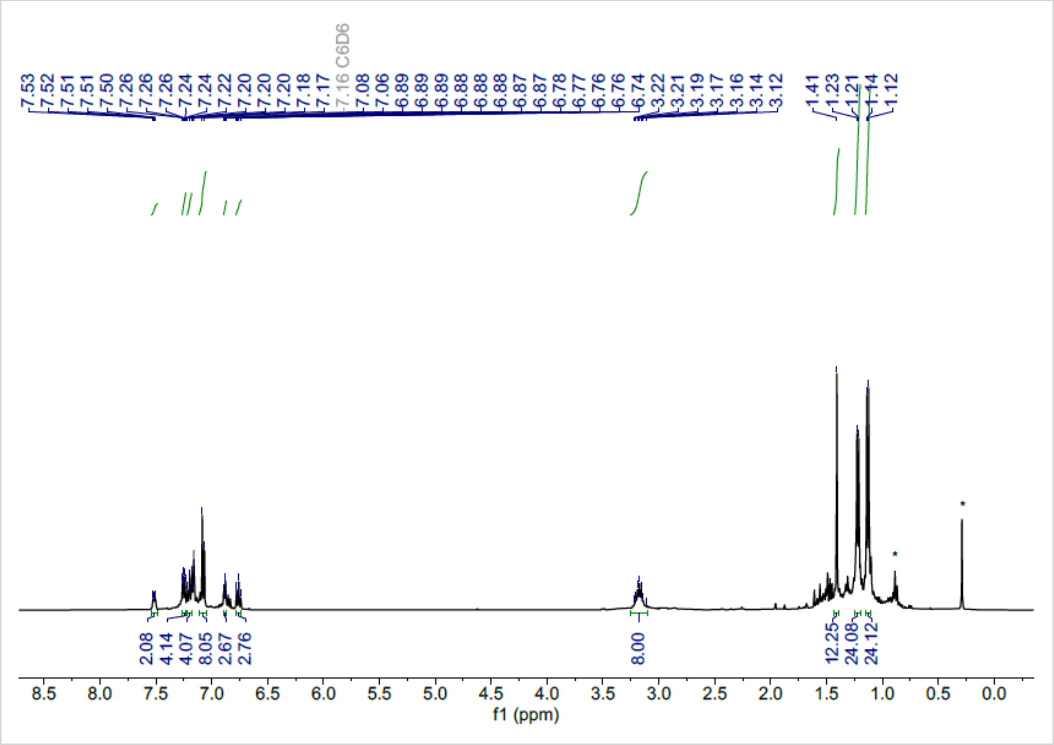

Figure S21. ^{1}H NMR spectrum of compound **5-Se^{Ph}** (400 MHz, C_6D_6, 298 K). * Corresponds to residual peaks of pentane and silicone grease.

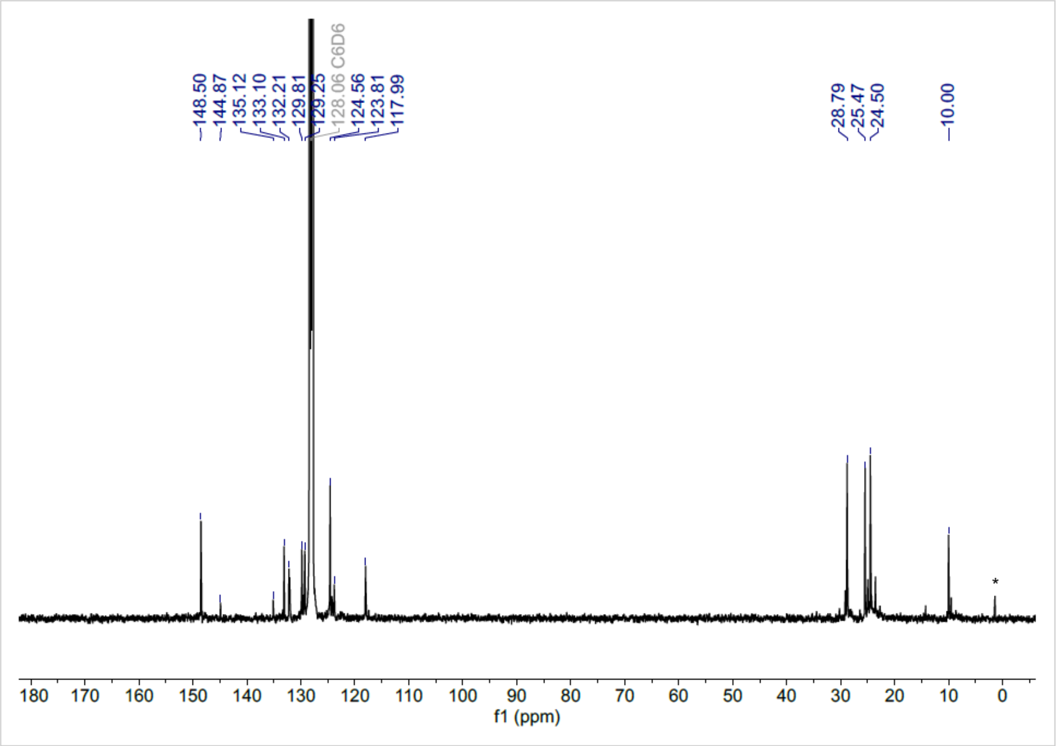

Figure S22. $^{13}C\{^{1}H\}$ NMR spectrum of compound **5-Se^{Ph}** (101 MHz, C_6D_6, 298 K). * Corresponds to residual peak of silicone grease.

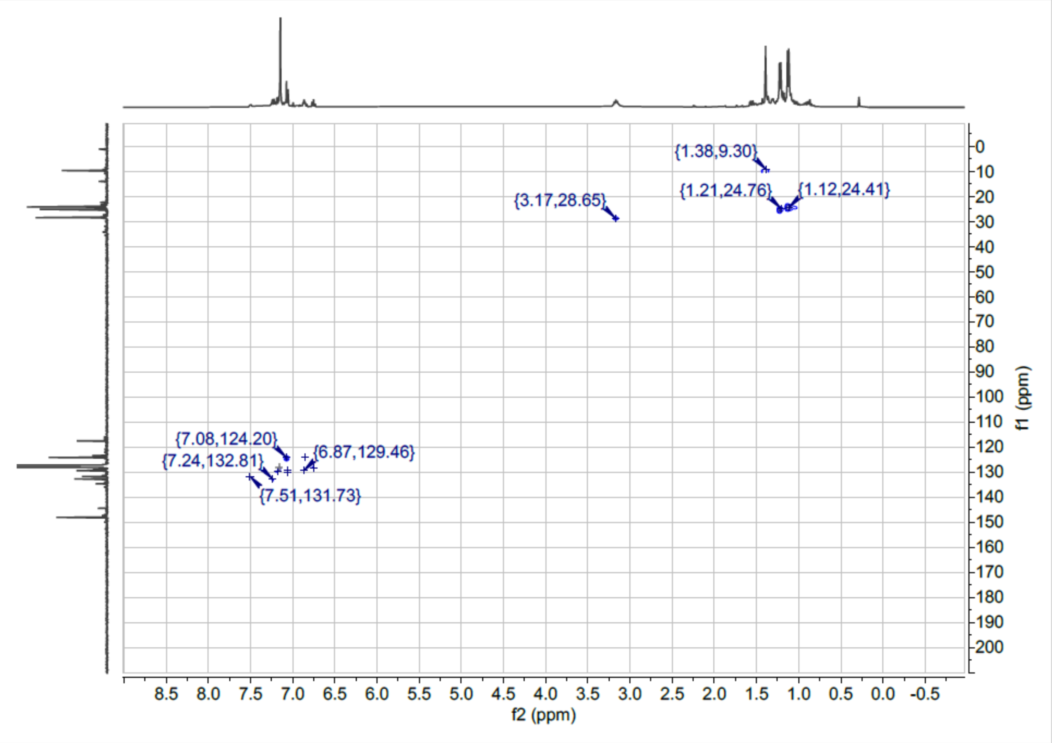

Figure S23. $^{1}H/^{13}C$ HSQC NMR spectrum of compound **5-Se^{Ph}** (C_6D_6, 298 K).

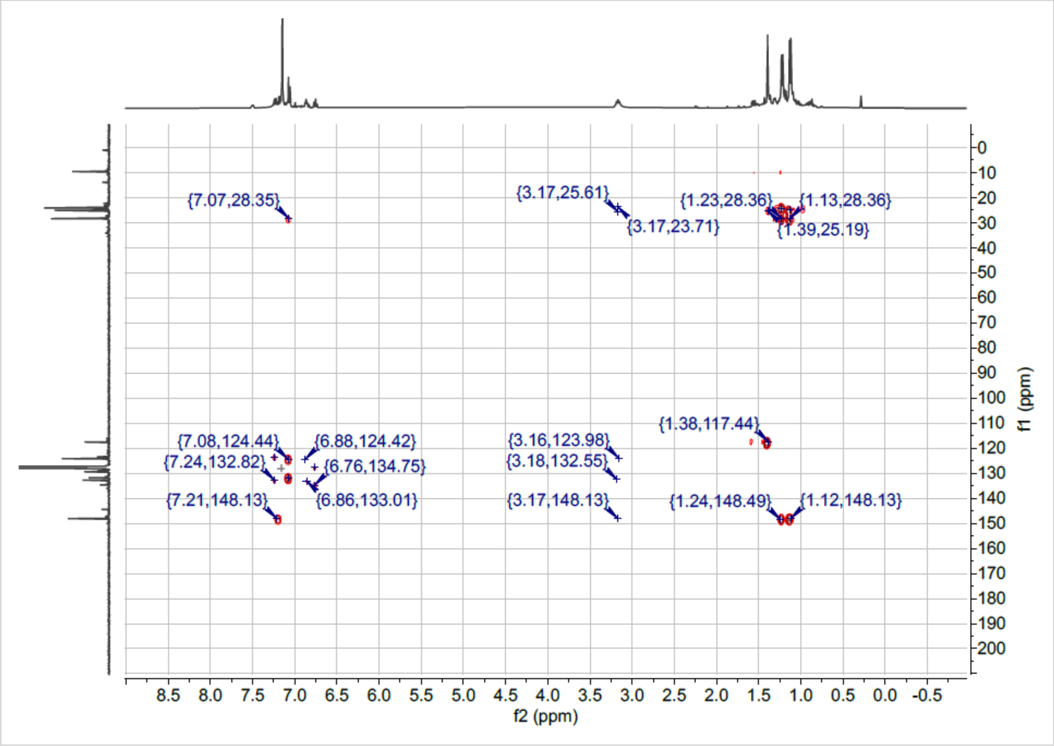

Figure S24. $^{1}H/^{13}C$ HMBC NMR spectrum of compound **5-Se^{Ph}** (C_6D_6, 298 K).

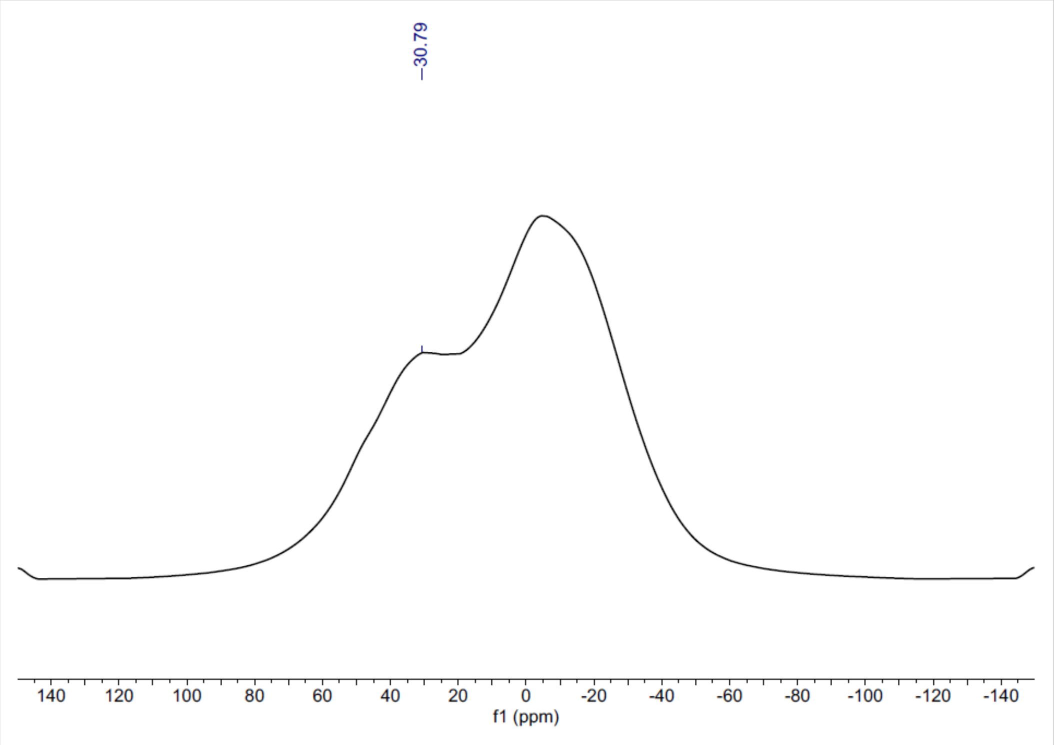

Figure S25. ^{11}B NMR spectrum of compound **5-Se^{Ph}** (128 MHz, C_6D_6, 298 K).

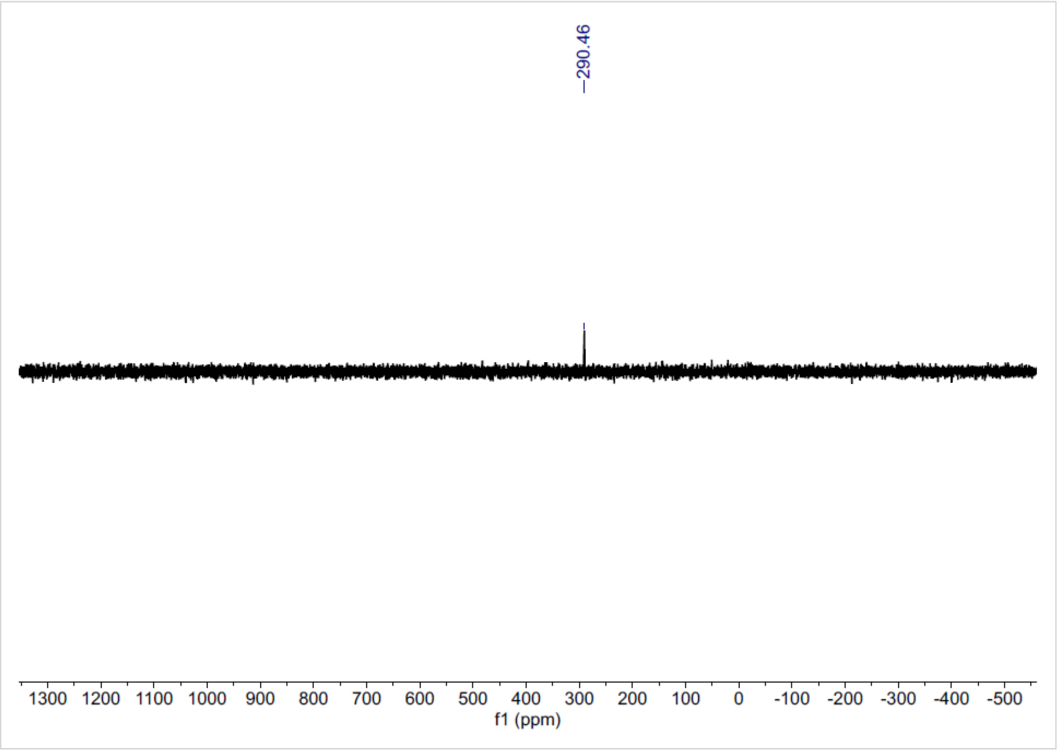

Figure S26. ^{77}Se NMR spectrum of compound **5-Se^{Ph}** (76 MHz, C_6D_6, 298 K).

1.2.7 Comparative control reactions of 2 with Me_2S_2 and Ph_2Se_2

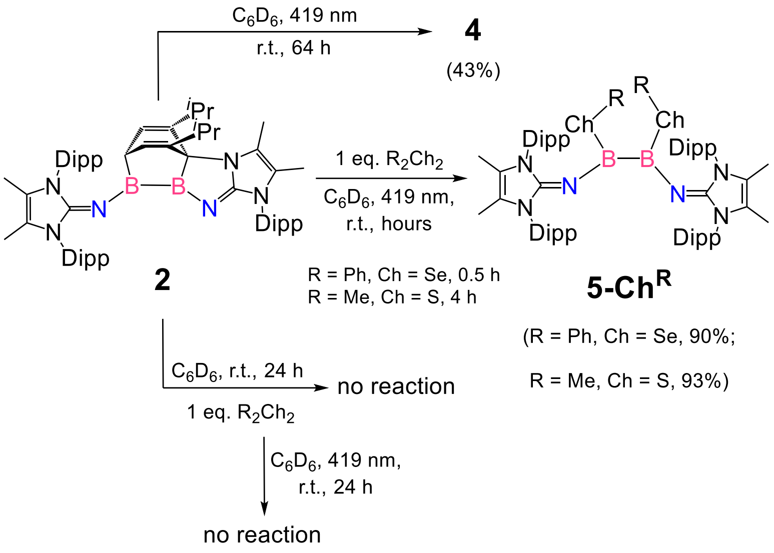

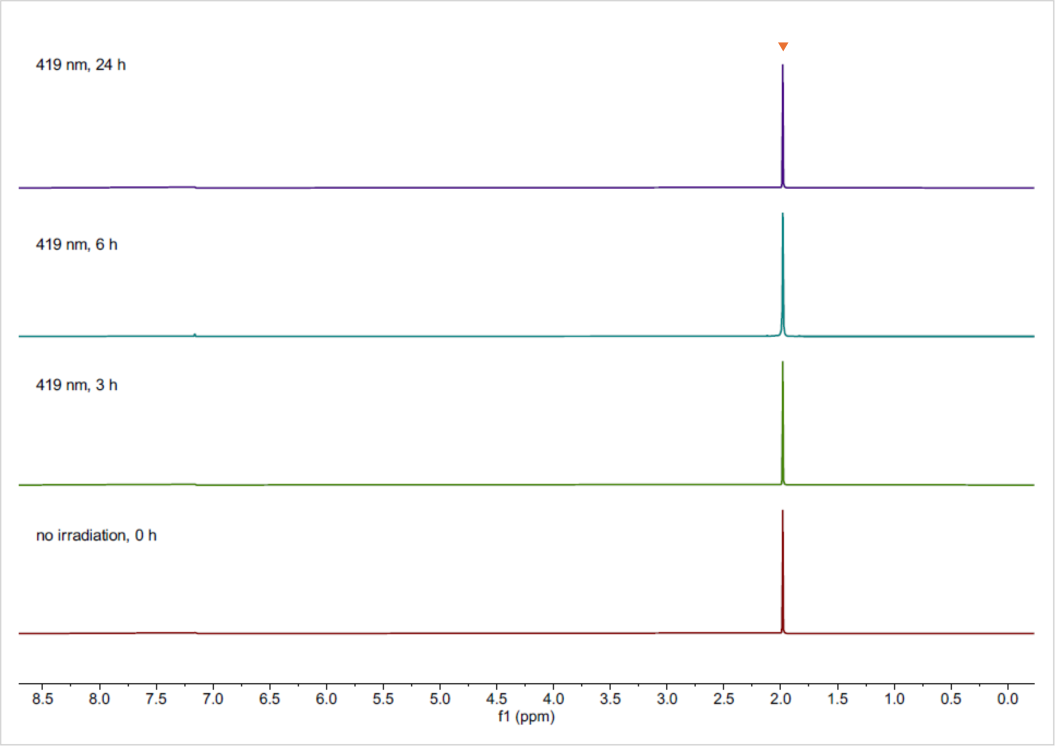

▼ = MeSSMe

Figure S27. Stacked ^{1}H NMR spectra of the blank experiment of Me_2S_2 in C_6D_6 in the absence of irradiation and under 419 nm irradiation for 3, 6 and 24 h (400 MHz, C_6D_6, 298 K).

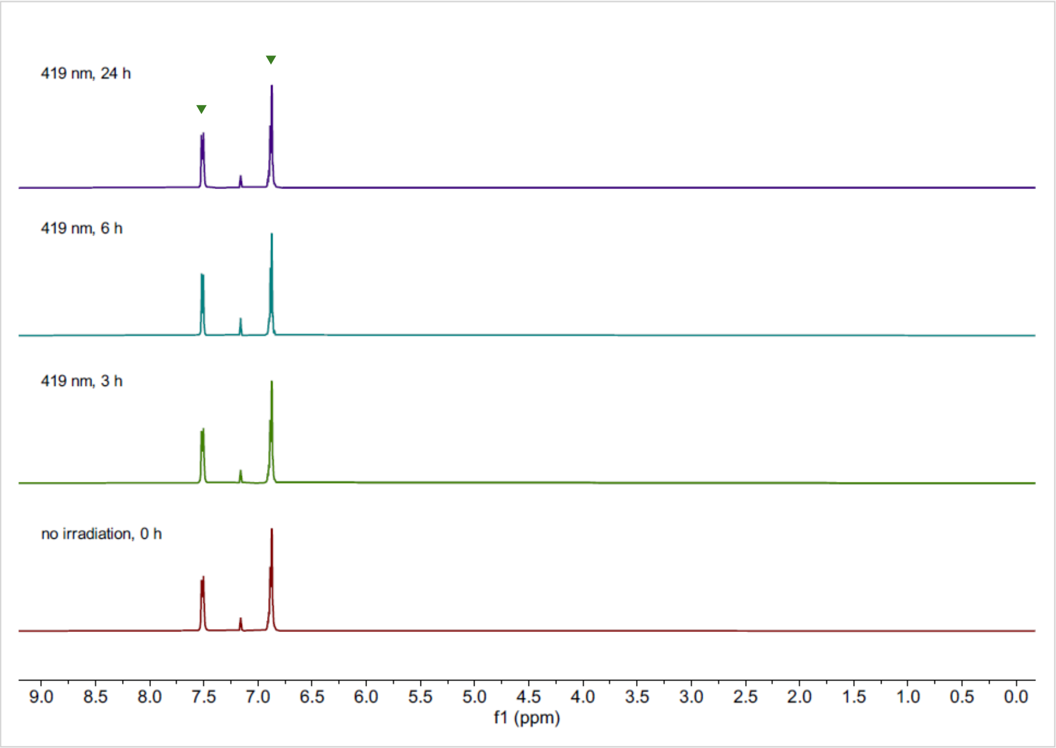

▼ = PhSeSePh

Figure S28. Stacked ^1H NMR spectra of the blank experiment of Ph_2Se_2 in C_6D_6 in the absence of irradiation and under 419 nm irradiation for 3, 6, and 24 h (400 MHz, C_6D_6, 298 K).

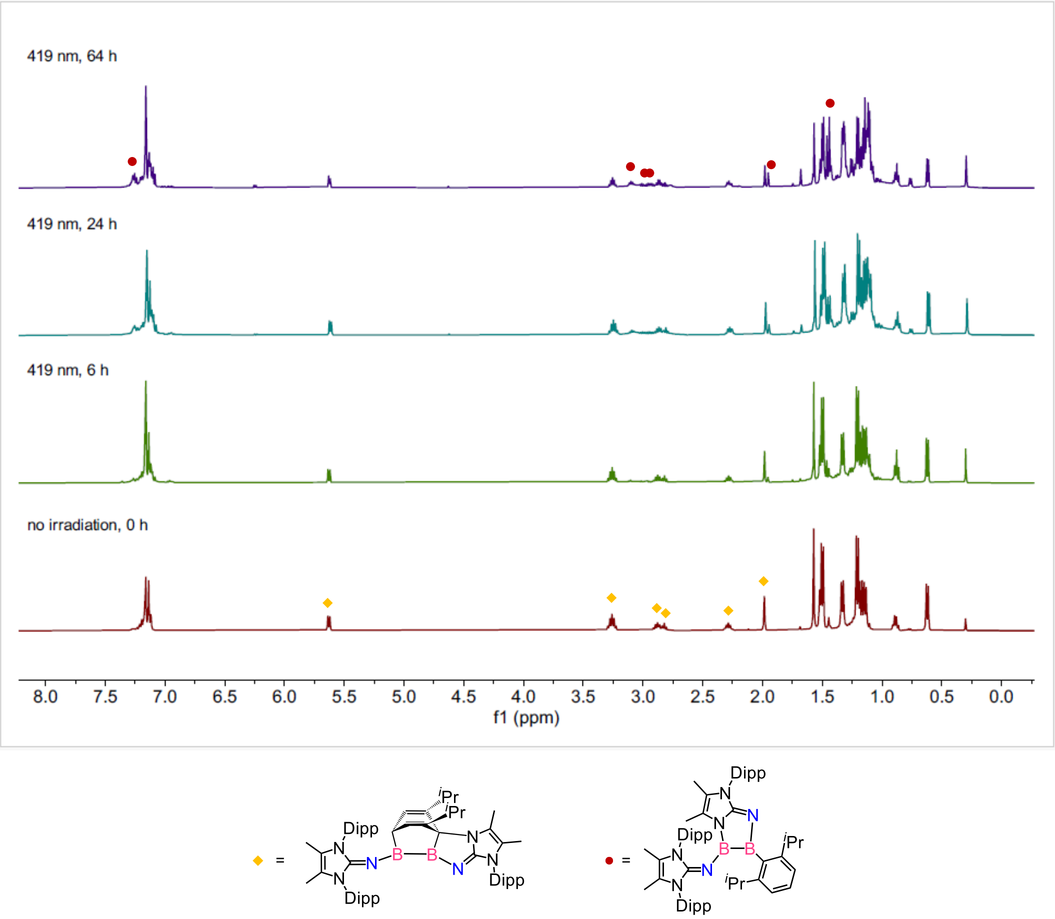

Figure S29. Stacked ^{1}H NMR spectra of the blank experiment of compound **2** in C_6D_6 in the absence of irradiation and under 419 nm irradiation for 6, 24 and 64 h (400 MHz, C_6D_6, 298 K).

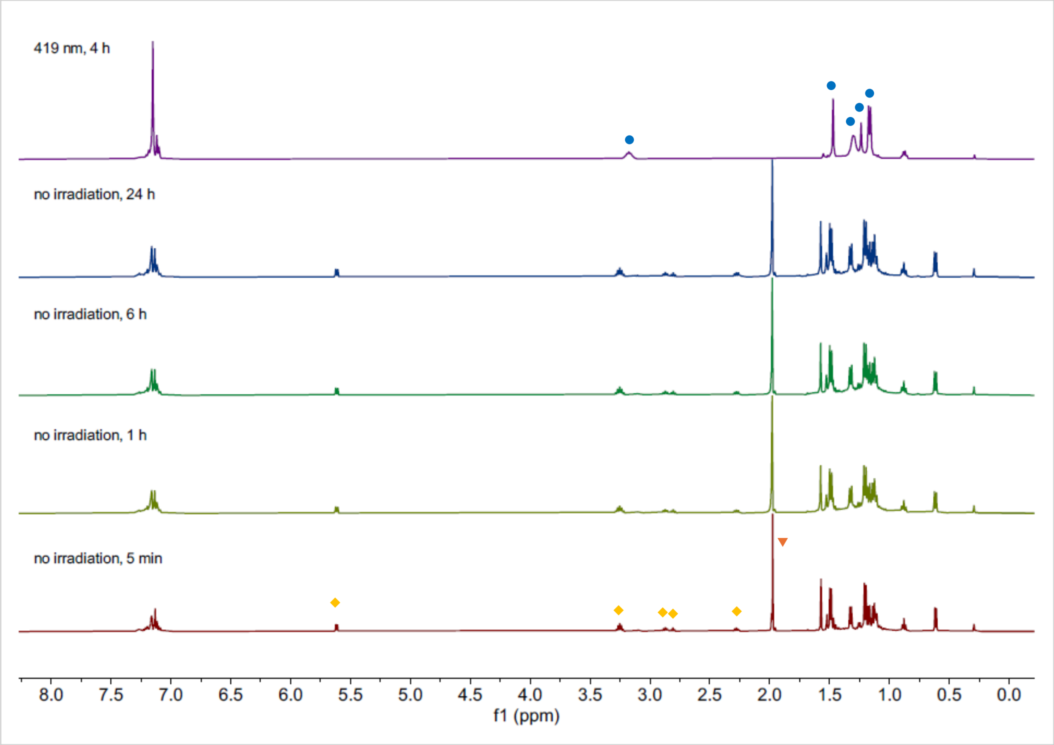

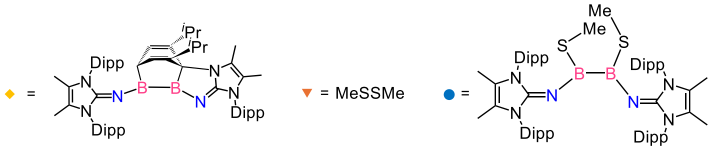

Figure S30. Stacked ^{1}H NMR spectra of the reactions of compound **2** with Me_2S_2 in C_6D_6 in the absence of irradiation for 5 min, 1 h, 6 h, and 24 h and under 419 nm irradiation for 4 h (400 MHz, C_6D_6, 298 K).

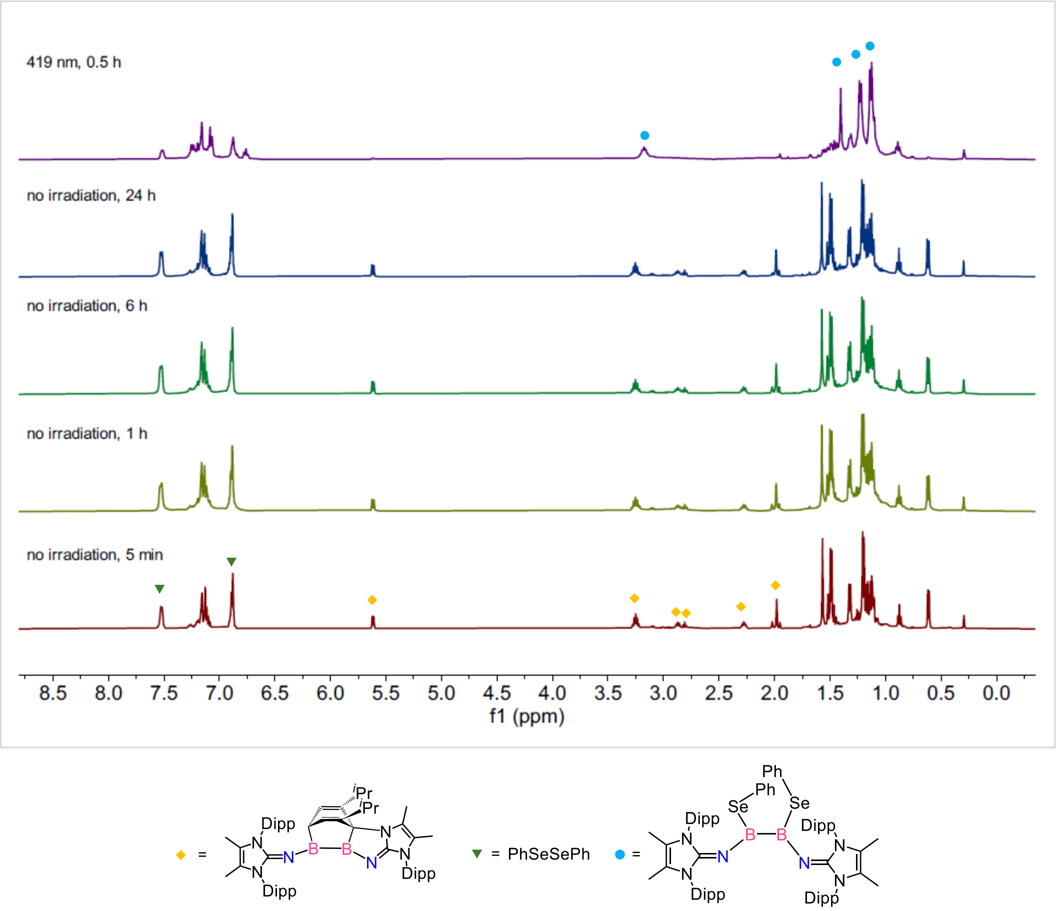

Figure S31. Stacked ^{1}H NMR spectra of the reactions of compound **2** with Ph_2Se_2 in C_6D_6 in the absence of irradiation for 5 min, 1 h, 6 h, and 24 h and under 419 nm irradiation for 0.5 h (400 MHz, C_6D_6, 298 K).

1.2.8 Synthesis of compound 6^{C}-H

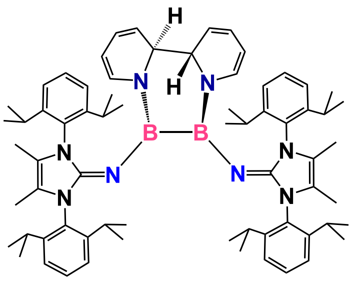

Method A: Compound **2** (80 mg, 0.0904 mmol) was dissolved in 3.0 mL of THF in a 10 mL pressure Schlenk flask at room temperature. The pyridine (14.6 μL, 0.1808 mmol) was added to the solution using a 20 μL pipette. The mixture was heated to 80 °C under stirring for 2 days, affording a deep orange solution. The solvent was removed under reduced pressure at 50 °C to yield dark red oil. The residue was extracted with 6 mL of pentane, and the volatiles evaporated. The resulting precipitate was washed with pentane (5 mL × 5), and the residual solvent was removed under vacuum to afford 5.0 mg of a bright-yellow solid.

Repetition of the washing and isolation procedure with the combined pentane extracts afforded an additional 19.4 mg of bright-yellow solid, giving a total of 24.4 mg (26.0%) compound **6^{C}-H**. Bright-yellow crystals suitable for X-ray diffraction analysis were obtained from a pentane solution at –30 °C for 5 days.

Method B: Compound **2** (20 mg, 0.0226 mmol) was dissolved in 0.4 mL of C_6D_6 or THF-d_8 in a 4 mL vial inside a glovebox. Pyridine (3.6 µL, 0.0452 mmol) was then added to the solution at room temperature. The reaction mixture was transferred to a J-Young NMR tube and irradiated at 419 nm for 24 h, affording an orange solution in C_6D_6 or a yellow solution in THF-d_8. Subsequently, NMR spectra were recorded, and the yield of compound **6^{C}-H** was determined using mesitylene as an internal standard.

^{1}H NMR (400 MHz, C_6D_6, 298 K): δ (ppm) 7.33 (t, *J* = 7.6 Hz, 4H, Ar^{Dipp}-*H*), 7.25 (t, *J* = 9.7 Hz, 6H, Ar^{Dipp}-*H*), 7.08 (d, *J* = 7.7 Hz, 2H, Ar^{Dipp}-*H*), 6.40 (d, *J* = 7.2 Hz, 2H, NC*H*=CH), 5.78 (dd, *J* = 10.1, 5.3 Hz, 2H, NCH=C*H*), 4.88 (d, *J* = 9.9 Hz, 2H, NCHC*H*), 4.76 (t, *J* = 6.3 Hz, 2H, NCH=CHC*H*), 3.47 (sept, *J* = 6.3 Hz, 2H, C*H*(CH_3)$_2$), 3.36 (s, 2H, NC*H*CH), 3.28 (sept, *J* = 6.3 Hz, 2H, C*H*(CH_3)$_2$), 3.12 (sept, *J* = 6.3 Hz, 2H, C*H*(CH_3)$_2$), 2.91 (sept, *J* = 6.3 Hz, 2H, C*H*(CH_3)$_2$), 1.54 (d, *J* = 6.9 Hz, 6H, CH(C*H*$_3$)$_2$), 1.46 (s, 12H, C*H*$_3$C=CC*H*$_3$), 1.44-1.31 (m, 18H, CH(C*H*$_3$)$_2$), 1.14-1.02 (m, 18H, CH(C*H*$_3$)$_2$), 0.70 (d, *J* = 6.8 Hz, 6H, CH(C*H*$_3$)$_2$).

^{13}C{^{1}H} NMR (101 MHz, C_6D_6, 298 K): δ [ppm] 149.51 (Ar^{Dipp}-*C*), 149.31 (Ar^{Dipp}-*C*), 148.71 (Ar^{Dipp}-*C*), 140.48 (N*C*N), 135.75 (N*C*H=CH), 134.75 (Ar^{Dipp}-*C*), 132.99 (Ar^{Dipp}-*C*), 129.61 (Ar^{Dipp}-*C*), 129.21 (Ar^{Dipp}-*C*), 125.74 (NCH=*C*H), 124.37 (Ar^{Dipp}-*C*), 124.01 (Ar^{Dipp}-*C*), 116.75 (CH_3*C*=*C*CH_3), 115.78 (CH_3*C*=*C*CH_3), 113.19 (NCH*C*H), 97.54 (NCH=CH*C*H), 62.38 (N*C*HCH), 28.63 (*C*H(CH_3)$_2$), 28.55 (*C*H(CH_3)$_2$), 28.40 (*C*H(CH_3)$_2$), 26.52 (CH(*C*H$_3$)$_2$), 25.30 (CH(*C*H$_3$)$_2$), 24.97 (CH(*C*H$_3$)$_2$), 24.73 (CH(*C*H$_3$)$_2$), 24.49 (CH(*C*H$_3$)$_2$), 24.26 (CH(*C*H$_3$)$_2$), 23.60 (CH(*C*H$_3$)$_2$), 22.42 (CH(*C*H$_3$)$_2$), 10.77 (*C*H$_3$C=C*C*H$_3$), 10.39 (*C*H$_3$C=C*C*H$_3$).

^{11}B NMR (128 MHz, C_6D_6, 298 K): δ [ppm] 33.04 (s, br).

LIFDI-MS: *m*/*z* for $C_{68}H_{90}B_2N_8$, Calcd: 1040.7475; Found: 1040.7353.

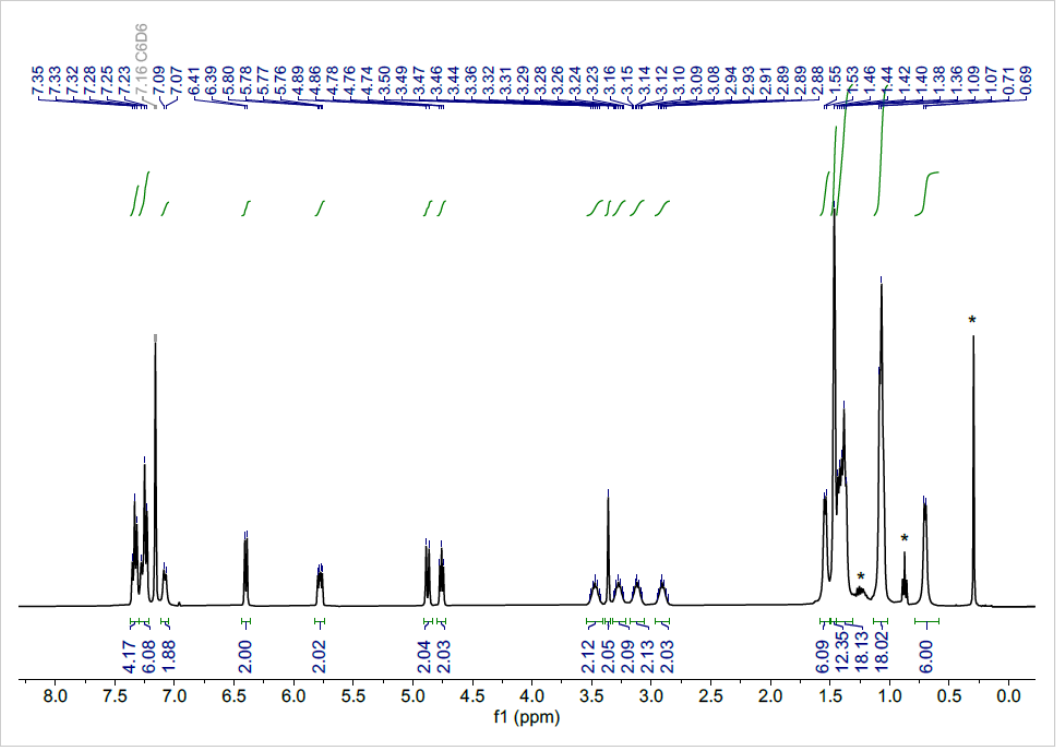

Figure S32. ^{1}H NMR spectrum of compound **6^{C}-H** (400 MHz, C_6D_6, 298 K). * Corresponds to residual peaks of pentane and silicone grease.

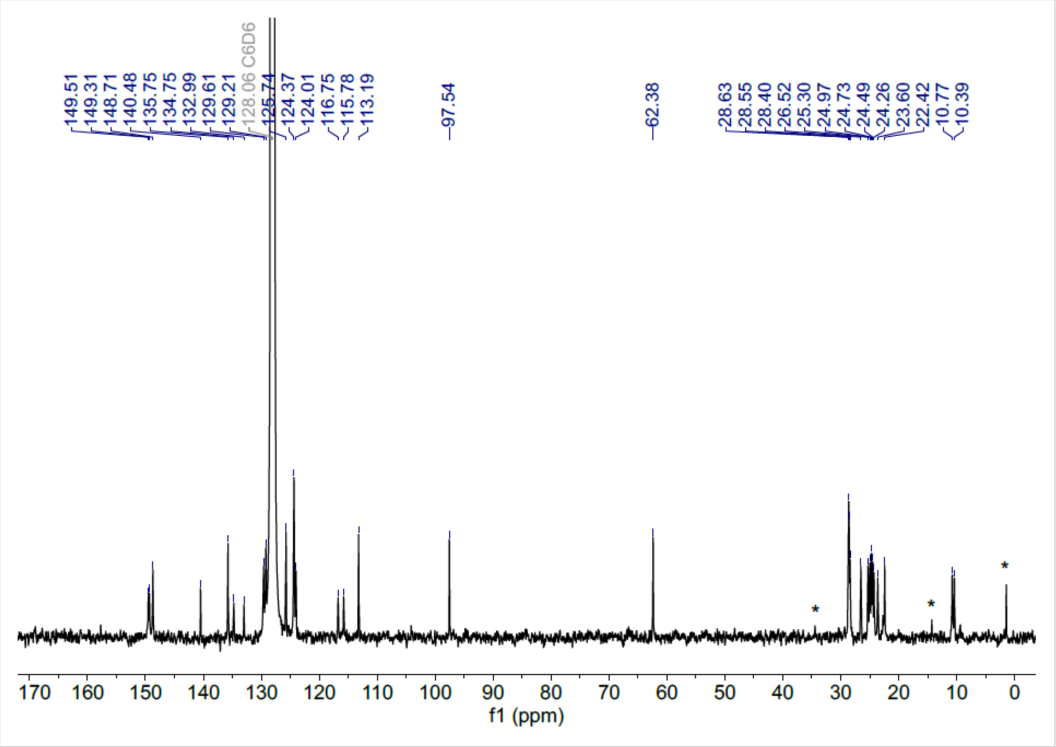

Figure S33. $^{13}C\{^1H\}$ NMR spectrum of compound **6^{C}-H** (101 MHz, C_6D_6, 298 K). * Corresponds to residual peaks of pentane and silicone grease.

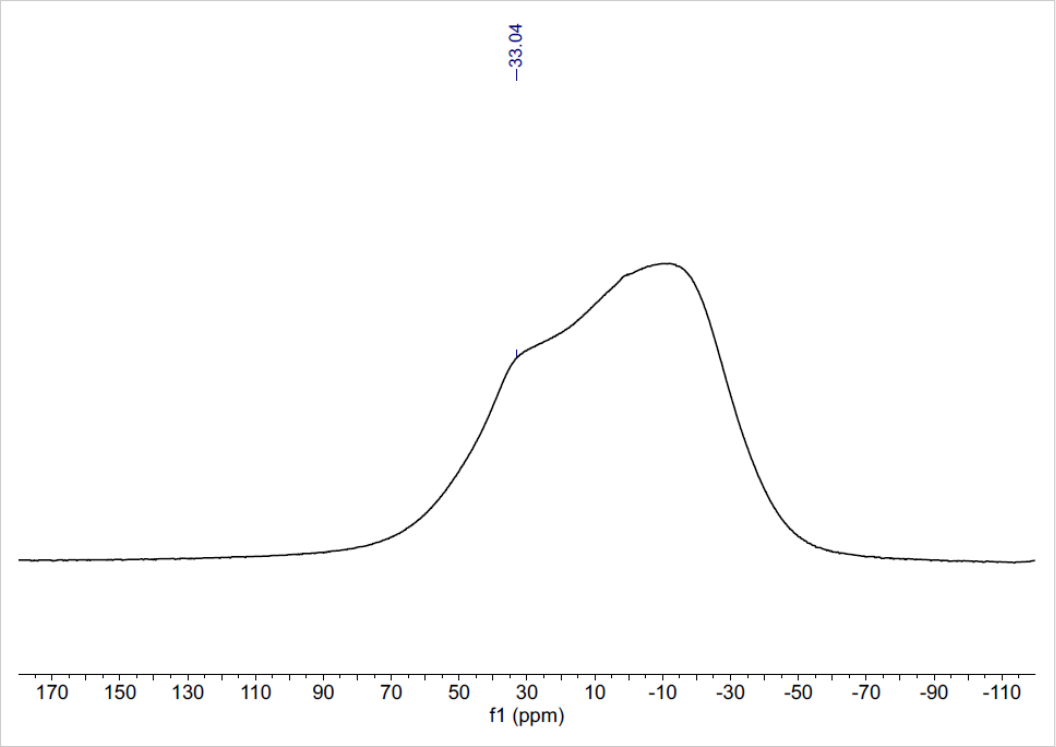

Figure S34. ^{11}B NMR spectrum of compound **6^{C}-H** (128 MHz, C_6D_6, 298 K).

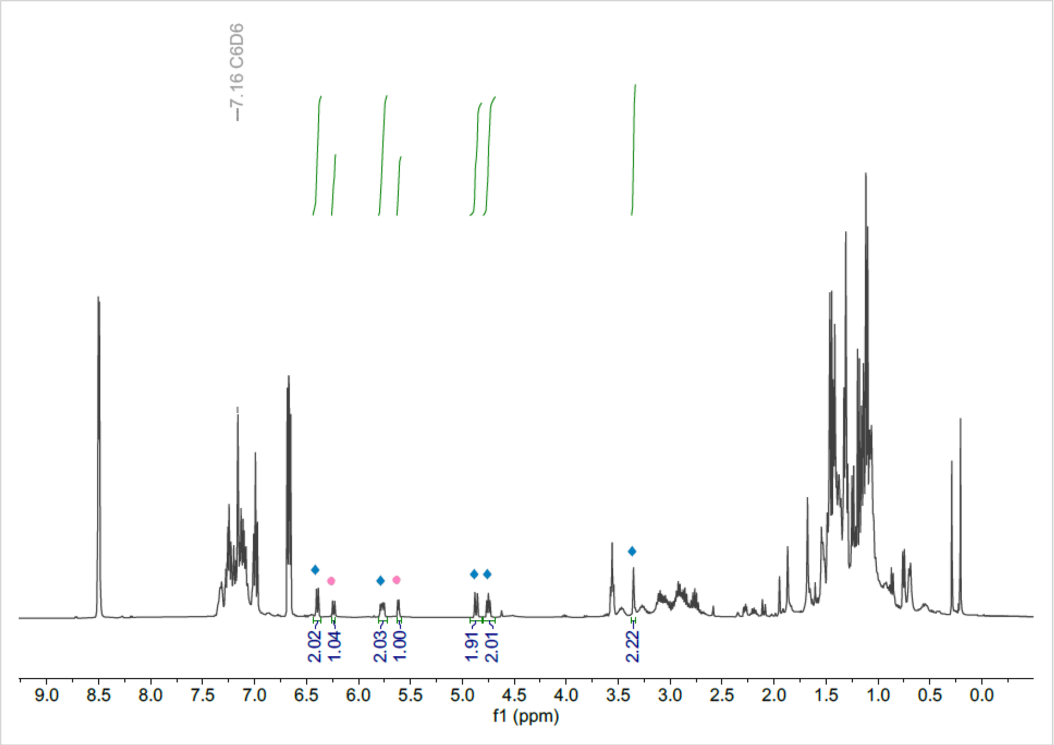

6[C]-H: 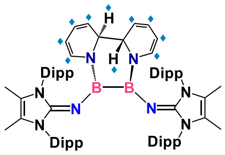

3: 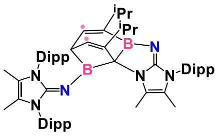

Figure S35. ^{1}H NMR spectrum of products **6^C-H** and **3** with nearly a 1:1 ratio resulting from the reaction of compound **2** with pyridine (the reaction was conducted in THF at 80 ºC for 2 days, then the solvent was removed in vacuo, and the mixture was dissolved in C_6D_6 (no precipitate was observed) for measurement).

Table S1 Ratio of products **6^C-H** and **3** for reaction of compound **2** with pyridine under different conditions

Solvent	Reaction condition	Ratio (**6^C-H**: **3**)
THF	80 ºC 2 d	~ 1: 1
THF	50 ºC 7 d	~ 1: 1
Benzene	70 ºC 3 d	~ 1: 1.8
Toluene	70 ºC 3 d	~ 1: 2.1
Pentane	70 ºC 3 d	~ 1: 2.6
Et_2O	70 °C 2 d	~ 1: 3.4
CH_2Cl_2	70 °C 2 d	decomposition

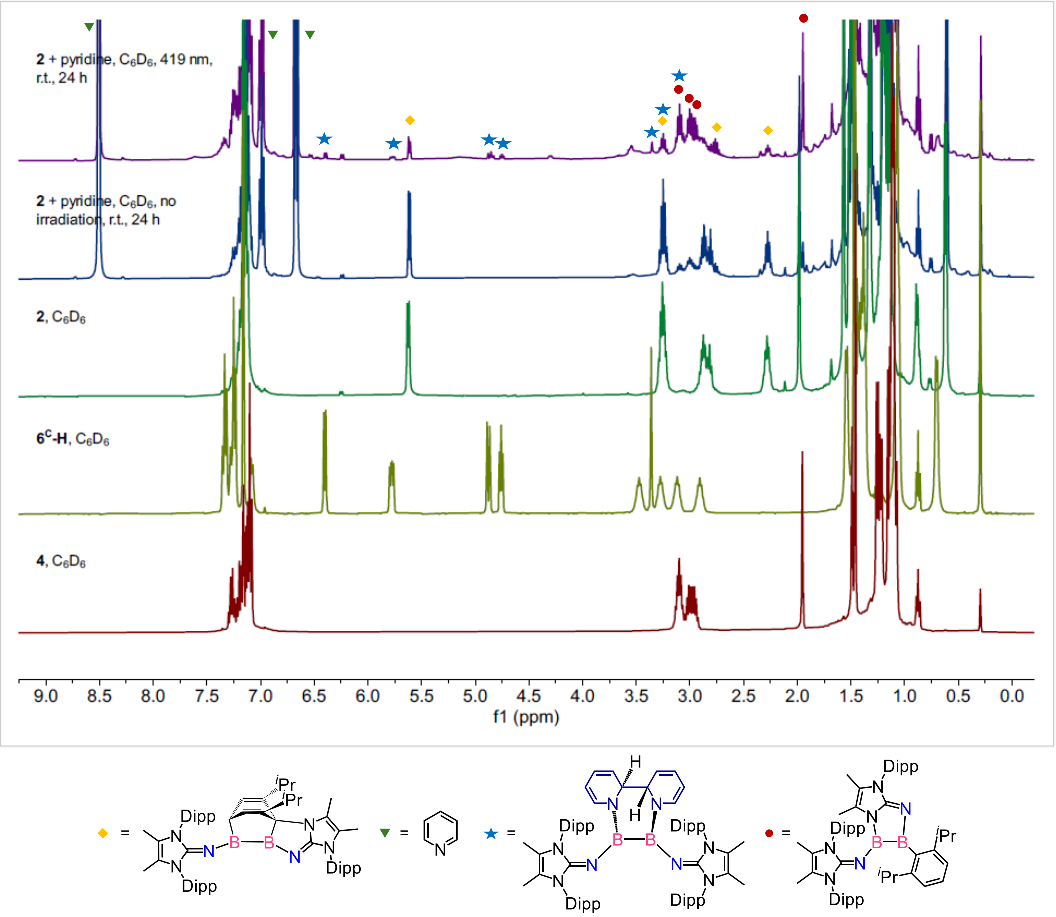

Figure S36 Stacked ^{1}H NMR spectra for the reaction of **2** with pyridine in C_6D_6 in the absence of irradiation and under 419 nm irradiation for 24 h (400 MHz, C_6D_6, 298 K).

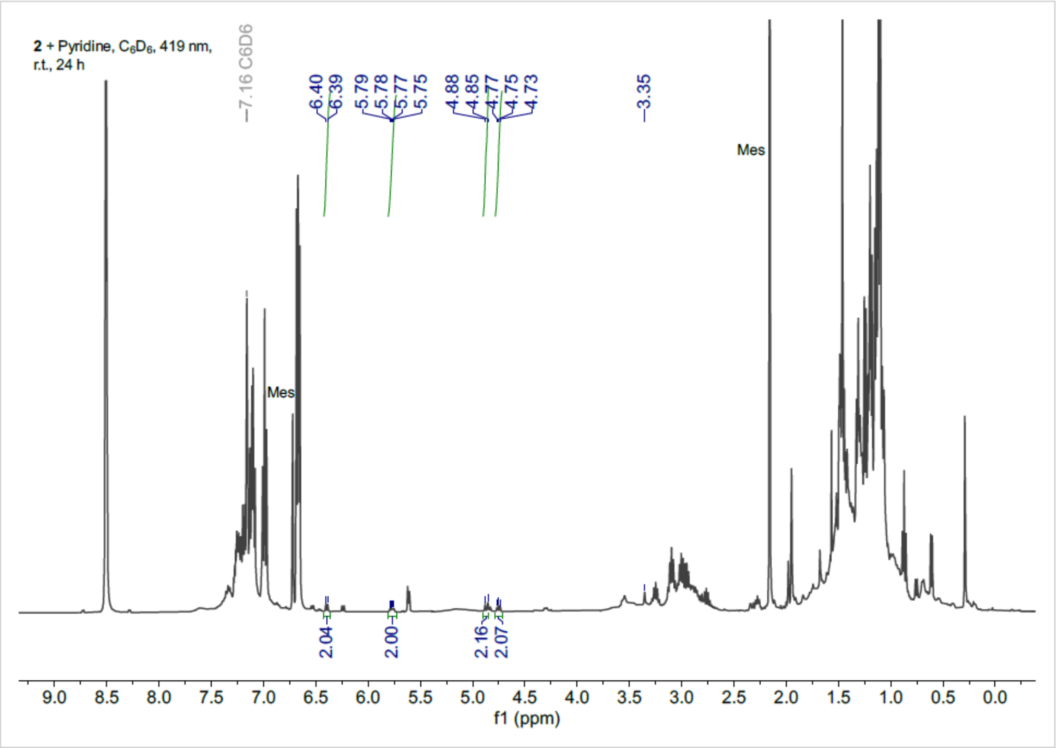

Figure S37 ^{1}H NMR spectrum of the reaction between **2** and pyridine in C_6D_6 under 419 nm irradiation for 24 h (400 MHz, C_6D_6, 298 K).

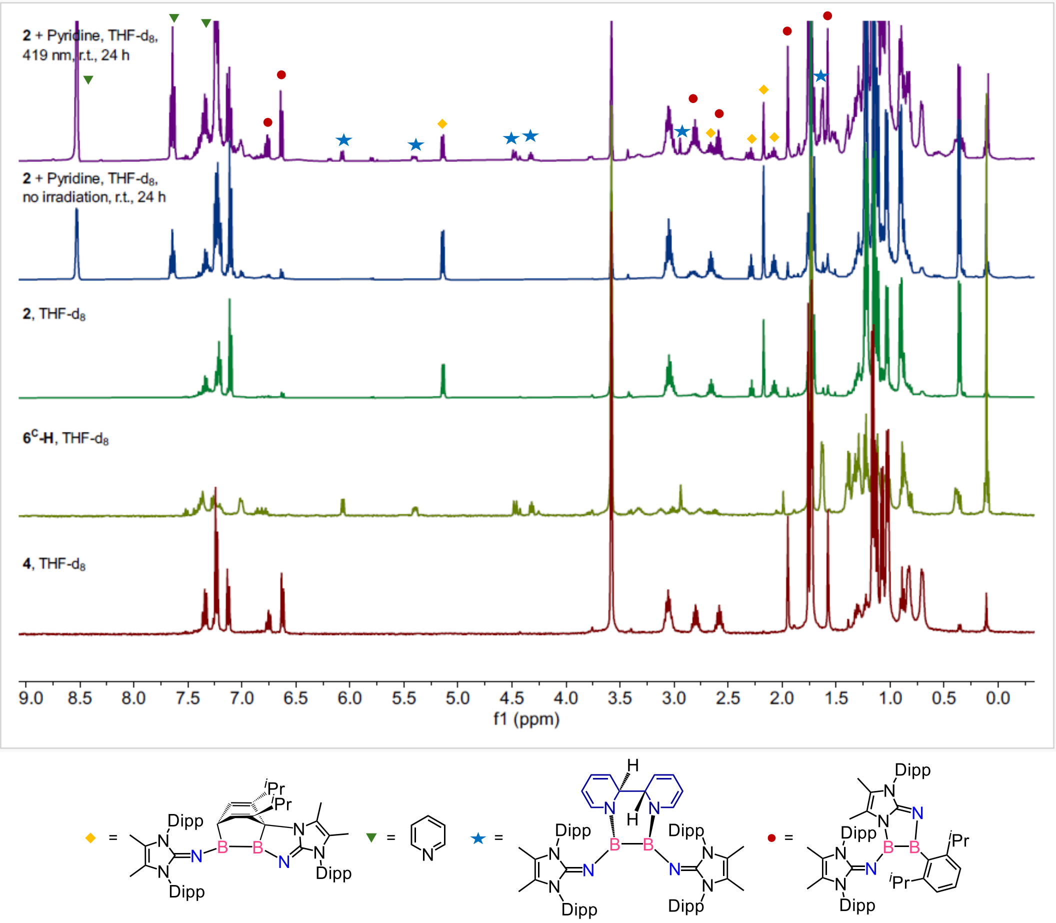

Figure S38 Stacked ^{1}H NMR spectra for the reaction of **2** with pyridine in THF-d_8 in the absence of irradiation and under 419 nm irradiation for 24 h (400 MHz, THF-d_8, 298 K).

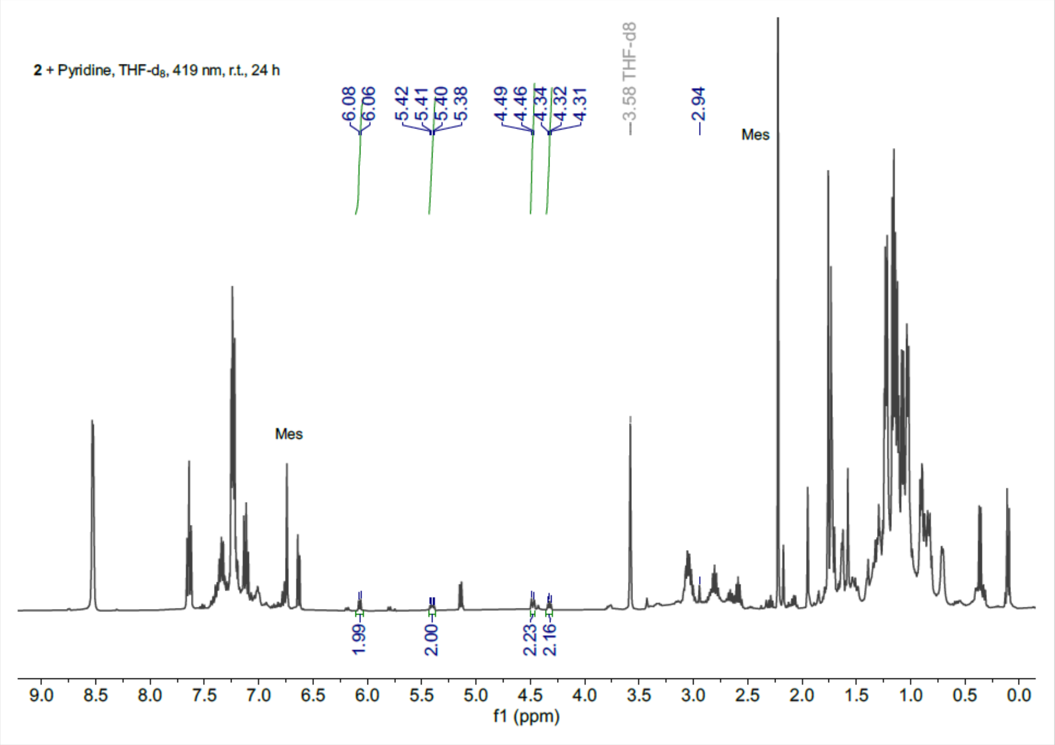

Figure S39 ^{1}H NMR spectrum of the reaction between **2** and pyridine in THF-d_8 under 419 nm irradiation for 24 h (400 MHz, THF-d_8, 298 K).

1.2.9 Synthesis of compound 6^C-CF_3

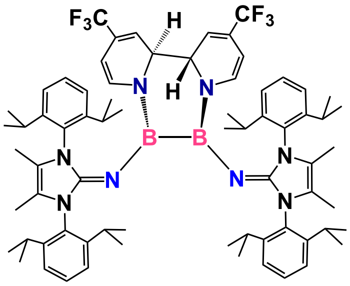

Compound **2** (40 mg, 0.0452 mmol) was dissolved in 1.0 mL of THF in a 4 mL vial and cooled to −30 °C in a glovebox and kept for 20 min. The cold 4-(trifluoromethyl) pyridine (10.6 μL, 0.0904 mmol) was added to the cold solution using a 20 μL pipette under stirring. The reaction mixture was maintained at −30 °C for 30 min. After removal of the solvent under reduced pressure, the resulting dark red oil was extracted with 3 mL of pentane. The solvent was evaporated, and the precipitate was washed with pentane (1 mL × 5). Removal of residual solvent afforded 11.0 mg of a bright-yellow solid. Repetition of the washing and isolation procedure with the combined pentane extracts yielded an additional 24.6 mg of compound **6^C-CF_3**. Yield: 35.6 mg (66.9%). Bright-yellow crystals suitable for X-ray diffraction analysis were obtained from a pentane solution at –30 °C for 5 days.

^{1}H NMR (400 MHz, C_6D_6, 298 K): δ (ppm) 7.35-7.27 (m, 2H, Ar^{Dipp}-*H*), 7.27-7.18 (m, 6H, Ar^{Dipp}-*H*), 7.14-7.08 (m, 2H, Ar^{Dipp}-*H*), 6.95-6.84 (m, 2H, Ar^{Dipp}-*H*), 6.48 (d, *J* = 7.3 Hz, 2H, NC*H*=CH), 5.30 (s, 2H, NCHC*H*), 4.99 (dd, *J* = 7.4, 1.8 Hz, 2H, NCH=C*H*), 3.32 (s, br, 2H, C*H*(CH_3)$_2$), 3.30 (s, 2H, NC*H*CH), 3.12 (s, br, 2H, C*H*(CH_3)$_2$), 2.98 (s, br, 2H, C*H*(CH_3)$_2$), 2.75 (s, br, 2H, C*H*(CH_3)$_2$), 1.46 (s, br, 6H, CH(CH_3)$_2$), 1.41 (s, 12H, CH_3C=CCH_3), 1.34 (s, br, 12H, CH(CH_3)$_2$), 1.22 (s, br, 6H, CH(CH_3)$_2$), 1.00 (s, br, 18H, CH(CH_3)$_2$), 0.60 (s, br, 6H, CH(CH_3)$_2$).

^{13}C{^{1}H} NMR (101 MHz, C_6D_6, 298 K): δ [ppm] 149.14 (Ar^{Dipp}-*C*), 148.94 (Ar^{Dipp}-*C*), 148.74 (Ar^{Dipp}-*C*), 148.40 (Ar^{Dipp}-*C*), 141.05 (N*C*N), 138.16 (N*C*H=CH), 134.18 (Ar^{Dipp}-*C*), 132.50 (Ar^{Dipp}-*C*), 130.52 (Ar^{Dipp}-*C*), 130.21 (Ar^{Dipp}-*C*), 129.89 (Ar^{Dipp}-*C*), 129.43 (Ar^{Dipp}-*C*), 125.50 (*C*F_3), 124.45 (Ar^{Dipp}-*C*), 124.23 (Ar^{Dipp}-*C*), 122.79 (*C*F_3), 116.95 (CH_3*C*=*C*CH_3), 116.15 (CH_3*C*=*C*CH_3), 111.32 (NCH*C*H), 111.26 (NCH=*C*H), 92.05 (NCH=CH*C*CF_3), 61.42 (N*C*HCH), 28.57 (*C*H(CH_3)$_2$), 28.49 (*C*H(CH_3)$_2$), 26.42 (CH(*C*H_3)$_2$), 25.03 (CH(*C*H_3)$_2$), 24.75 (CH(*C*H_3)$_2$), 24.37 (CH(*C*H_3)$_2$), 23.82 (CH(*C*H_3)$_2$), 23.55 (CH(*C*H_3)$_2$), 22.20 (CH(*C*H_3)$_2$), 10.56 (*C*H_3C=C*C*H_3), 10.24 (*C*H_3C=C*C*H_3).

^{11}B NMR (128 MHz, C_6D_6, 298 K): δ [ppm] 31.63 (s, br).

^{19}F NMR (376 MHz, C_6D_6, 298 K): δ [ppm] −68.92 (s).

LIFDI-MS: *m/z* for $C_{70}H_{88}B_2F_6N_8$, Calcd: 1176.7222; Found: 1176.7233.

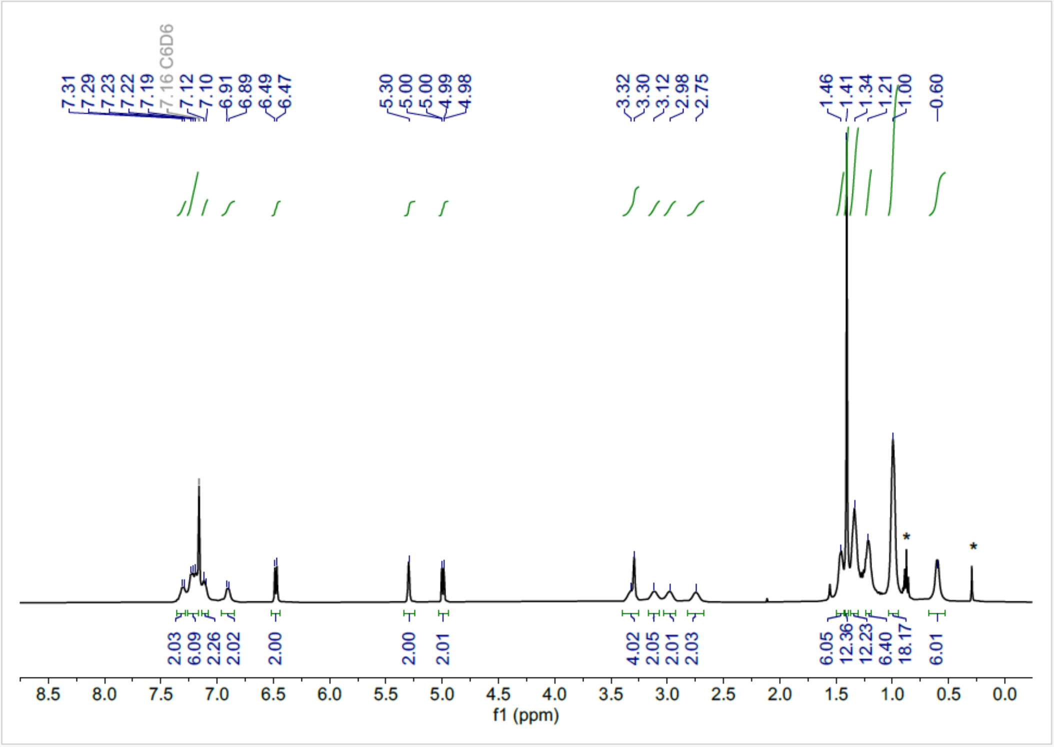

Figure S40. ^{1}H NMR spectrum of compound **6[C]-CF₃** (400 MHz, C_6D_6, 298 K). * Corresponds to residual peaks of pentane, hexane and silicone grease.

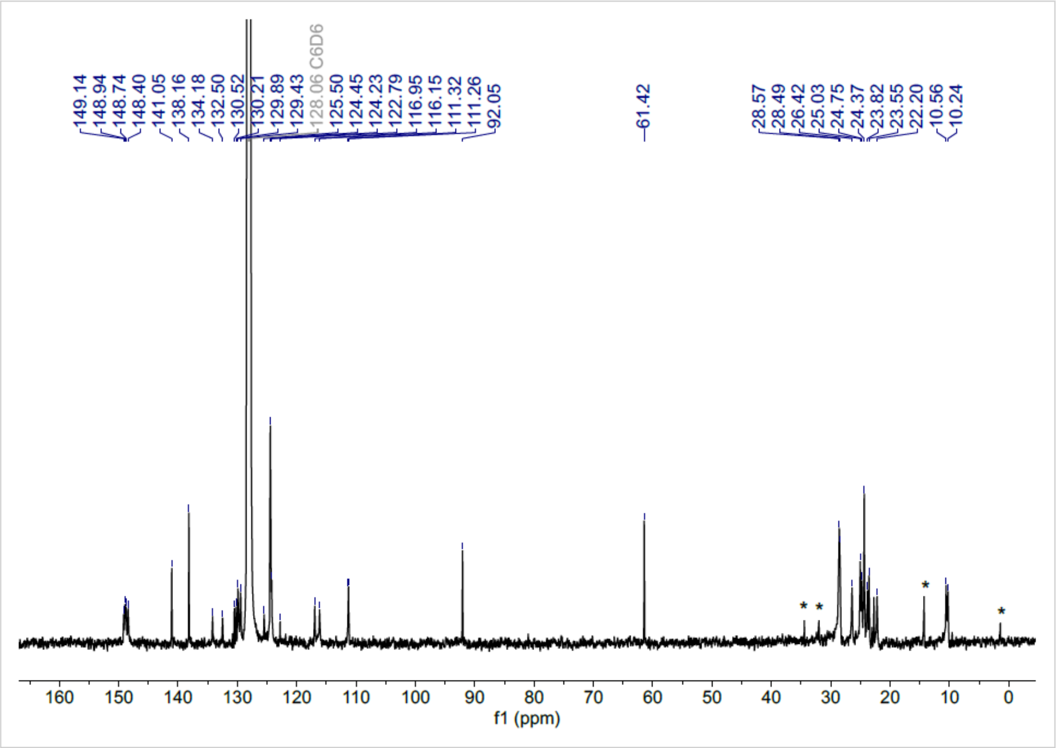

Figure S41. $^{13}C\{^1H\}$ NMR spectrum of compound **6^C-CF$_3$** (101 MHz, C_6D_6, 298 K). * Corresponds to residual peaks of pentane, hexane and silicone grease.

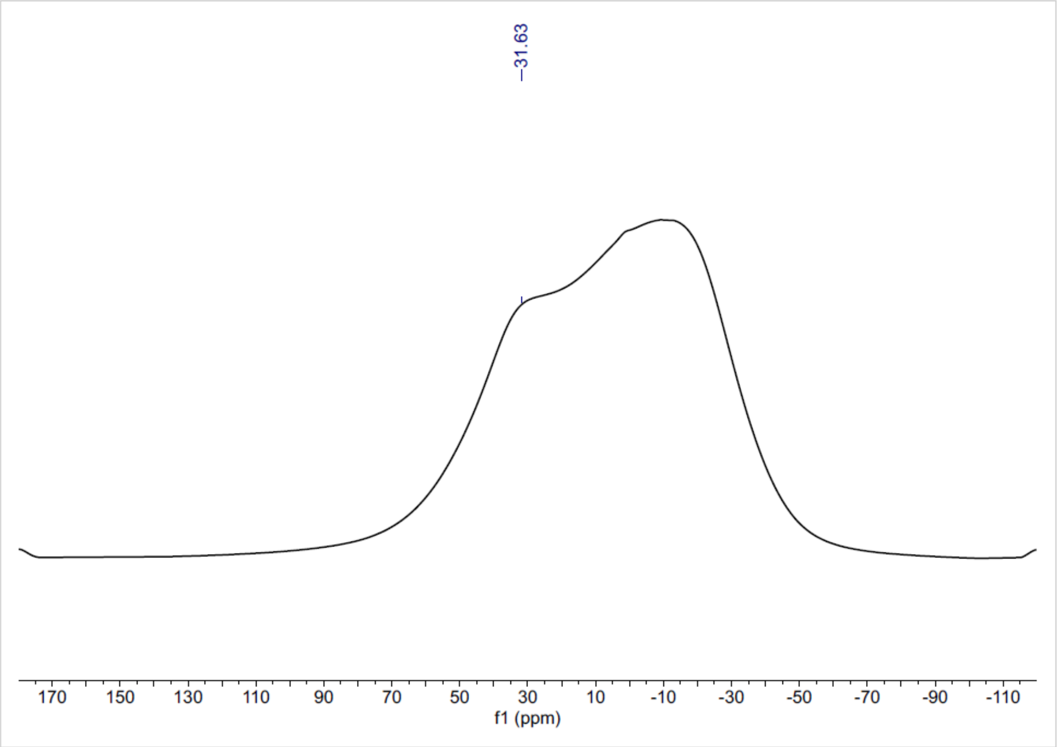

Figure S42. ^{11}B NMR spectrum of compound **6^C-CF$_3$** (128 MHz, C_6D_6, 298 K).

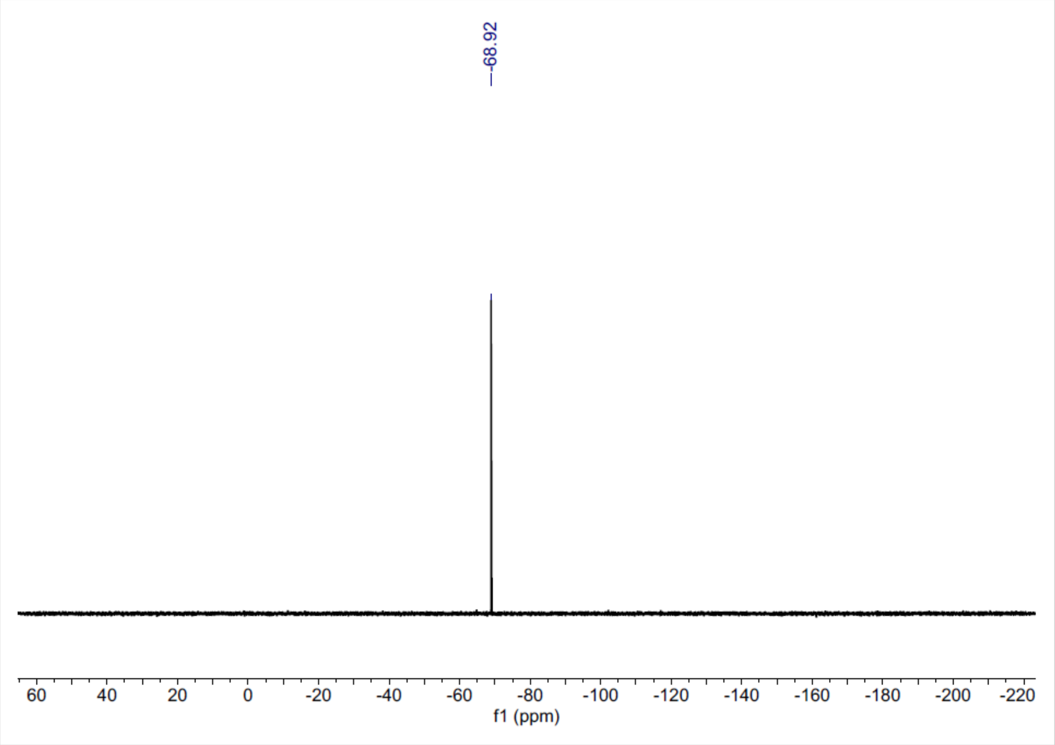

Figure S43. ^{19}F NMR spectrum of compound **6^{C}-CF$_3$** (376 MHz, C_6D_6, 298 K).

1.2.10 Synthesis of compound 6^{C}-CN

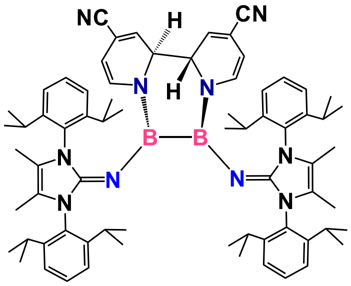

Compound **2** (40 mg, 0.0452 mmol) was dissolved in 1.0 mL of THF in a 4 mL vial and cooled to −30 °C in a glovebox and kept for 20 min. The 4-cyanopyridine (9.4 mg, 0.0904 mmol) was dissolved in 0.2 mL of THF and cooled to −30 °C and kept for 20 min. The cold 4-cyanopyridine solution was added dropwise to the cold solution of compound **2**. The reaction mixture was maintained at −30 °C for 30 min. After removal of the solvent under reduced pressure, the resulting dark red oil was extracted with 3 mL of pentane. The solvent was evaporated, and the precipitate was washed with pentane (1 mL × 5). Removal of residual solvent afforded 10.5 mg of a bright-yellow solid. Repetition of the washing and isolation procedure with the combined pentane extracts yielded an additional 19.2 mg of compound **6^{C}-CN**. Yield: 29.7 mg (60.2%).

^{1}H NMR (400 MHz, C_6D_6, 298 K): 7.29 (s, br, 2H, Ar^{Dipp}-H), 7.20 (s, br, 6H, Ar^{Dipp}-H), 7.06 (s, br, 2H, Ar^{Dipp}-H), 6.93 (s, br, 2H, Ar^{Dipp}-H), 6.28 (d, *J* = 7.7 Hz, 2H, NC*H*=CH), 4.78-4.69 (m, 4H, NCHC*H* + NCH=C*H*), 3.28 (s, br, 2H, C*H*$(CH_3)_2$), 3.04-2.95 (m, 4H, C*H*$(CH_3)_2$ + NC*H*CH), 2.90 (s, br, 2H, C*H*$(CH_3)_2$), 2.66 (s, br, 2H, C*H*$(CH_3)_2$), 1.42 (s, br, 6H, CH$(CH_3)_2$), 1.38 (s, 12H, CH_3C=CCH_3), 1.32 (s, br, 6H, CH$(CH_3)_2$), 1.22 (s, br, 6H, CH$(CH_3)_2$), 1.12 (s, br, 6H, CH$(CH_3)_2$), 0.98 (s, br, 18H, CH$(CH_3)_2$), 0.53 (s, br, 6H, CH$(CH_3)_2$).

^{13}C{^{1}H} NMR (101 MHz, C_6D_6, 298 K): δ [ppm] 148.84 (Ar^{Dipp}-*C*), 148.78 (Ar^{Dipp}-*C*), 148.68 (Ar^{Dipp}-*C*), 148.25 (Ar^{Dipp}-*C*), 141.15 (N*C*N), 137.65 (N*C*H=CH), 133.97 (Ar^{Dipp}-*C*), 132.39 (Ar^{Dipp}-*C*), 129.84 (Ar^{Dipp}-*C*), 129.53 (Ar^{Dipp}-*C*), 124.44 (Ar^{Dipp}-*C*), 122.54 (NCH*C*H), 118.41 (*C*N), 116.90 (CH_3*C*=*C*CH_3), 116.27 (CH_3*C*=*C*CH_3), 114.10 (*C*(CN)), 95.00 (NCH=*C*H), 61.28 (N*C*HCH), 28.51 (*C*H$(CH_3)_2$), 26.37 (*C*H$(CH_3)_2$), 25.02 (*C*H$(CH_3)_2$), 24.74 (*C*H$(CH_3)_2$), 24.32 (CH$(CH_3)_2$), 23.53 (CH$(CH_3)_2$), 22.16 (CH$(CH_3)_2$), 10.55 (CH_3C=CCH_3), 10.19 (CH_3C=CCH_3).

^{11}B NMR (128 MHz, C_6D_6, 298 K): δ [ppm] 31.41 (s, br).

LIFDI-MS: *m*/*z* for $C_{70}H_{88}B_2N_{10}$, Calcd: 1090.7380; Found: 1090.7295.

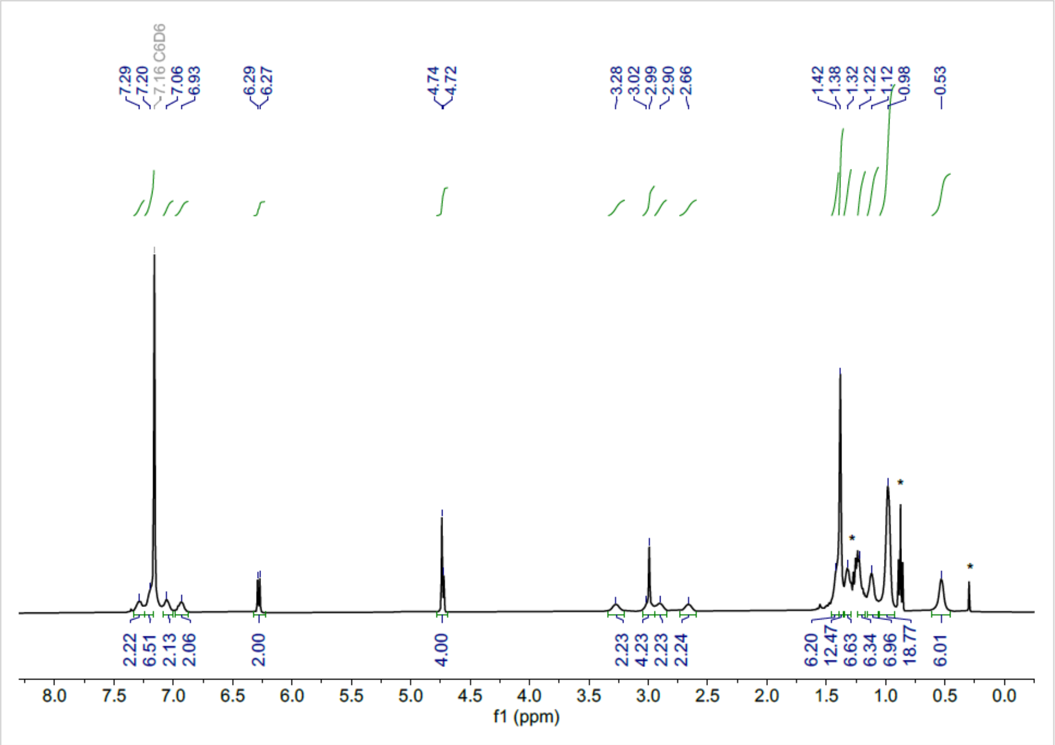

Figure S44. ^{1}H NMR spectrum of compound **6^C-CN** (400 MHz, C_6D_6, 298 K). * Corresponds to residual peaks of pentane and silicone grease.

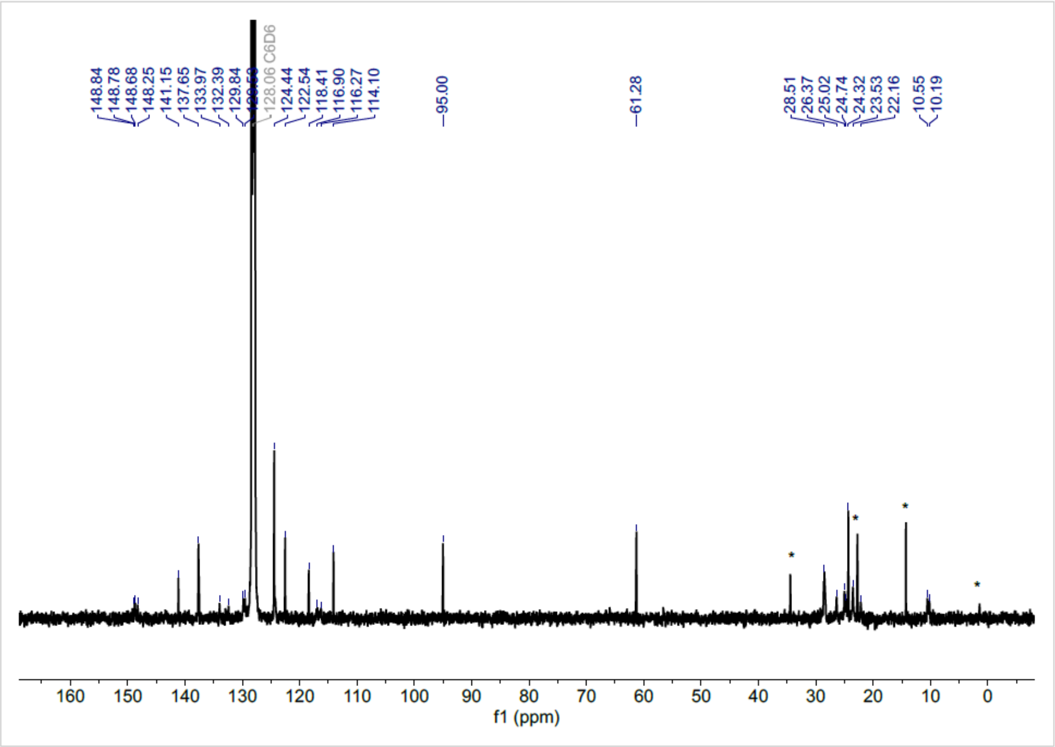

Figure S45. ^{13}C{^{1}H} NMR spectrum of compound **6^C-CN** (101 MHz, C_6D_6, 298 K). * Corresponds to residual peaks of pentane and silicone grease.

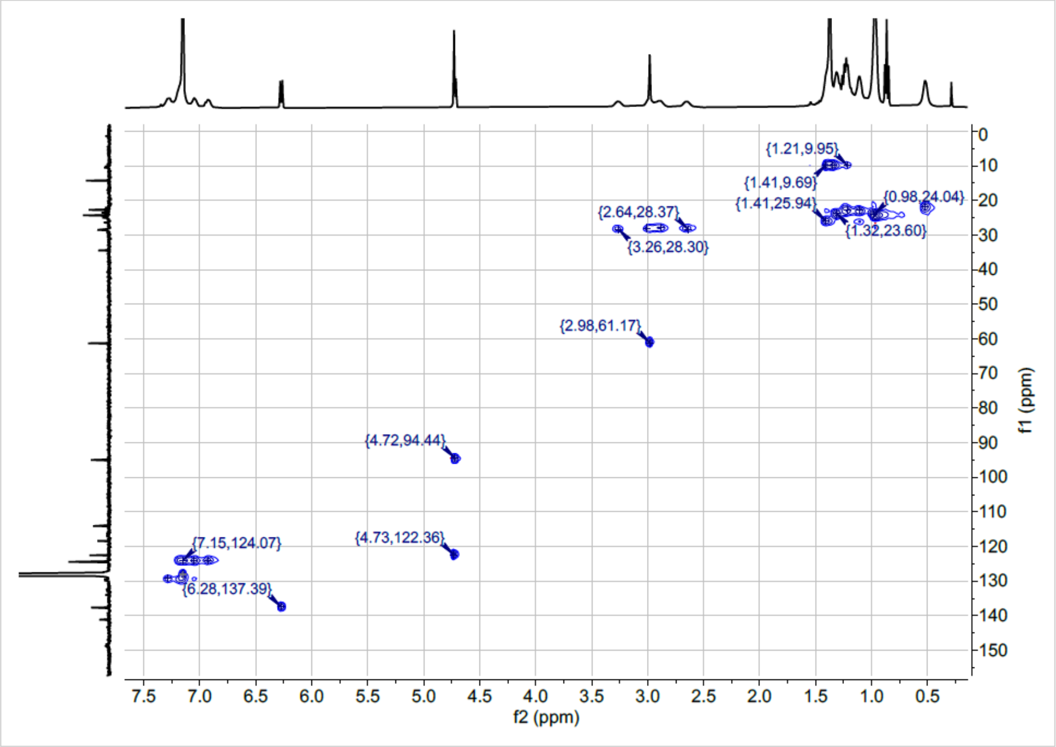

Figure S46. $^{1}H/^{13}C$ HSQC NMR spectrum of compound **6^{C}-CN** (C_6D_6, 298 K).

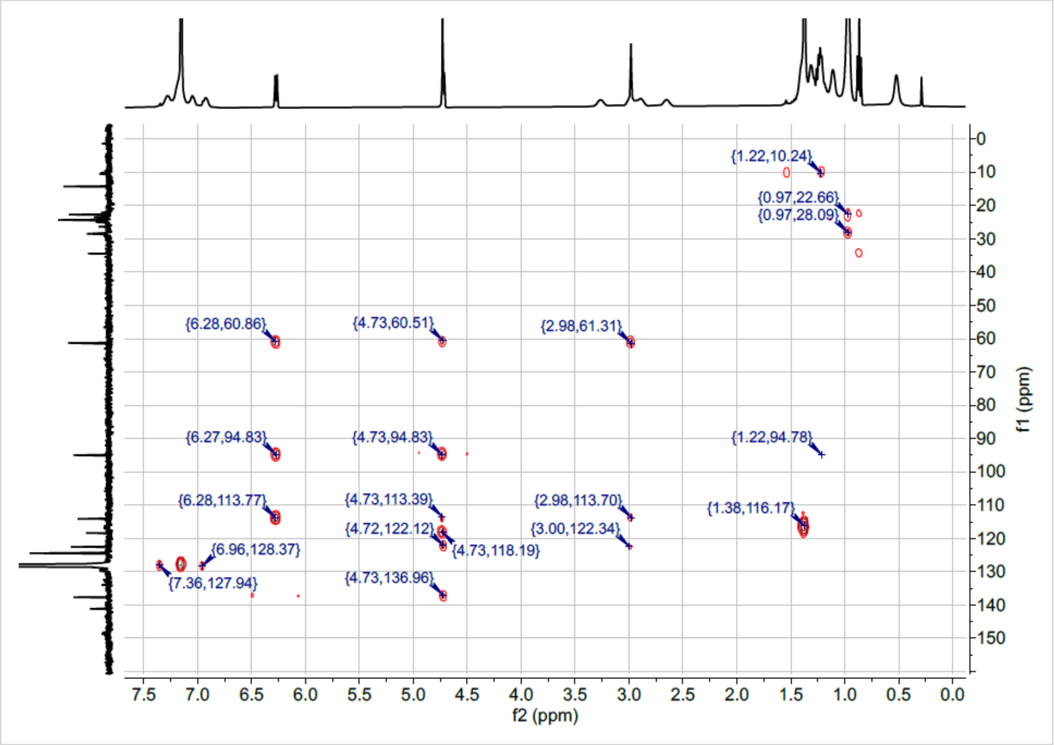

Figure S47. $^{1}H/^{13}C$ HMBC NMR spectrum of compound **6^C-CN** (C_6D_6, 298 K).

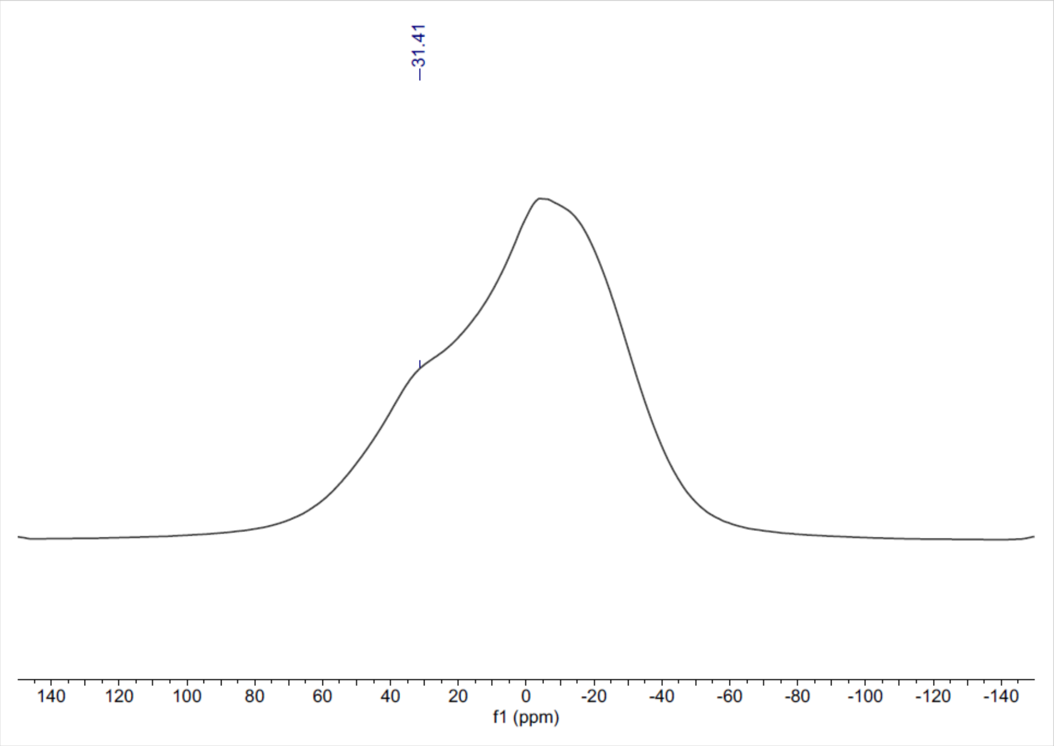

Figure S48. ^{11}B NMR spectrum of compound **6^C-CN** (128 MHz, C_6D_6, 298 K).

1.2.11 Synthesis of compound 6^N

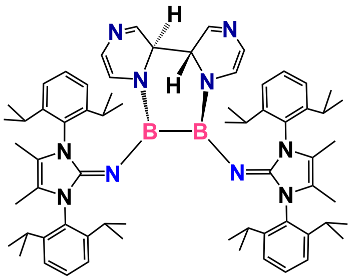

Compound **2** (40 mg, 0.0452 mmol) was dissolved in 1.0 mL of THF in a 4 mL vial in a glovebox. The pyrazine (7.2 mg, 0.0904 mmol) was dissolved in 0.2 mL of THF. The pyrazine solution was added dropwise to the solution of compound **2** at room temperature. The reaction mixture was stirred for 1 h. After removal of the solvent under reduced pressure, the resulting yellow solid was extracted with 3 mL of pentane. The solvent was evaporated, and the precipitate was washed with pentane (1 mL × 5). Removal of residual solvent afforded 13.8 mg of a bright-yellow solid. Repetition of the washing and isolation procedure with the combined pentane extracts yielded an additional 21.4 mg of compound **6^N**. Yield: 35.2 mg (74.7%).

^{1}H NMR (400 MHz, C_6D_6, 298 K): 7.37-7.28 (m, 2H, Ar^{Dipp}-H), 7.28-7.19 (m, 4H, Ar^{Dipp}-H), 7.11-7.04 (m, 2H, Ar^{Dipp}-H), 7.04-6.97 (m, 2H, Ar^{Dipp}-H), 6.95-6.86 (m, 2H, Ar^{Dipp}-H), 6.63 (s, 2H, NCHC*H*), 6.60 (d, *J* = 4.8 Hz, 2H, NCH=C*H*), 6.23 (d, *J* = 4.7 Hz, 2H, NC*H*=CH), 3.39 (s, br, 2H, C*H*$(CH_3)_2$), 3.24 (s, br, 2H, C*H*$(CH_3)_2$), 3.07 (s, br, 2H, C*H*$(CH_3)_2$), 2.73 (s, br, 2H, C*H*$(CH_3)_2$), 2.58 (s, 2H, NC*H*CH), 1.50 (d, *J* = 6.4 Hz, 6H, CH$(CH_3)_2$), 1.44 (s, br, 6H, CH$(CH_3)_2$), 1.42 (s, 12H, CH_3C=CCH_3), 1.37 (d, *J* = 6.9 Hz, 6H, CH$(CH_3)_2$), 1.15 (d, *J* = 6.5 Hz, 6H, CH$(CH_3)_2$), 1.07-0.94 (m, 18H, CH$(CH_3)_2$), 0.59 (d, 6H, *J* = 5.0 Hz, CH$(CH_3)_2$).

^{13}C{^{1}H} NMR (101 MHz, C_6D_6, 298 K): δ [ppm] 149.12 (Ar^{Dipp}-*C*), 148.87 (Ar^{Dipp}-*C*), 148.56 (Ar^{Dipp}-*C*), 141.12 (NCHCH), 141.02 (NCN), 134.33 (Ar^{Dipp}-*C*), 132.58 (Ar^{Dipp}-*C*), 129.51 (Ar^{Dipp}-*C*), 129.45 (Ar^{Dipp}-*C*), 126.35 (NCH=CH), 124.44 (Ar^{Dipp}-*C*), 124.15 (Ar^{Dipp}-*C*), 119.07 (NCH=*C*H), 116.84 (CH_3C=CCH_3), 115.97 (CH_3C=CCH_3), 53.13 (NCHCH), 28.67 (*C*H$(CH_3)_2$), 28.56 (*C*H$(CH_3)_2$), 28.38 (*C*H$(CH_3)_2$), 28.30 (*C*H$(CH_3)_2$), 26.67 (CH$(CH_3)_2$), 25.21 (CH$(CH_3)_2$), 24.72 (CH$(CH_3)_2$), 24.36 (CH$(CH_3)_2$), 23.88 (CH$(CH_3)_2$), 23.45 (CH$(CH_3)_2$), 22.18 (CH$(CH_3)_2$), 10.65 (CH_3C=CCH_3), 10.23 (CH_3C=CCH_3).

^{11}B NMR (128 MHz, C_6D_6, 298 K): δ [ppm] 31.67 (s, br).

LIFDI-MS: *m*/*z* for $C_{66}H_{88}B_2N_{10}$, Calcd: 1042.7380; Found: 1042.7350.

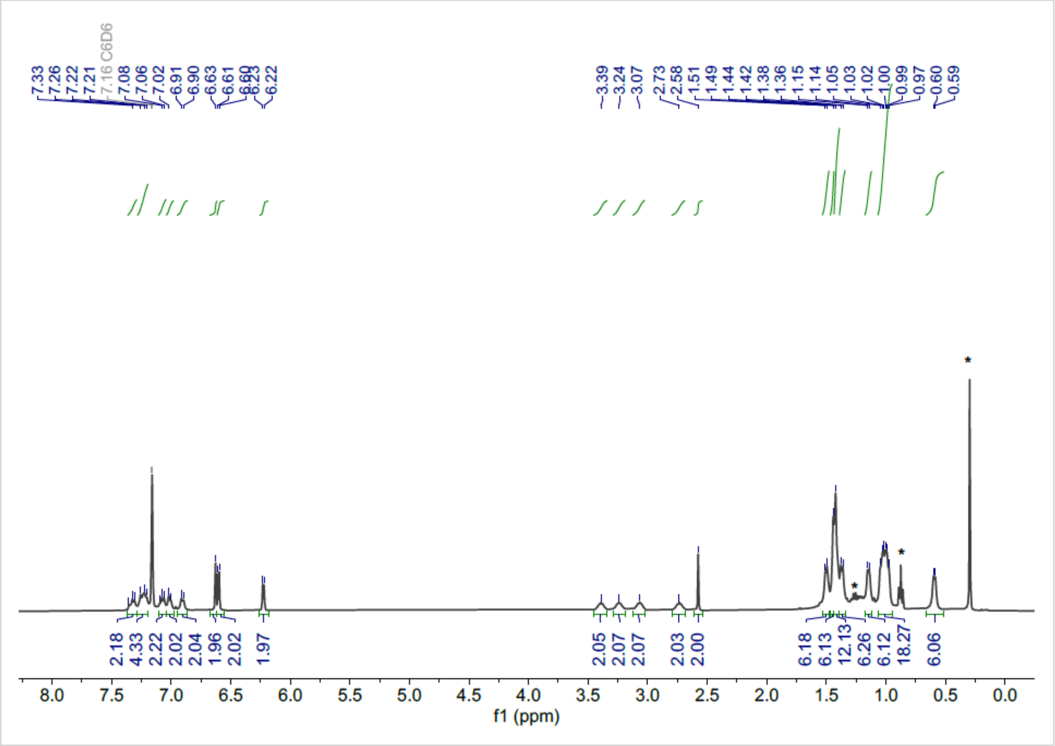

Figure S49. ^{1}H NMR spectrum of compound **6^N** (400 MHz, C_6D_6, 298 K). * Corresponds to residual peaks of pentane and silicone grease.

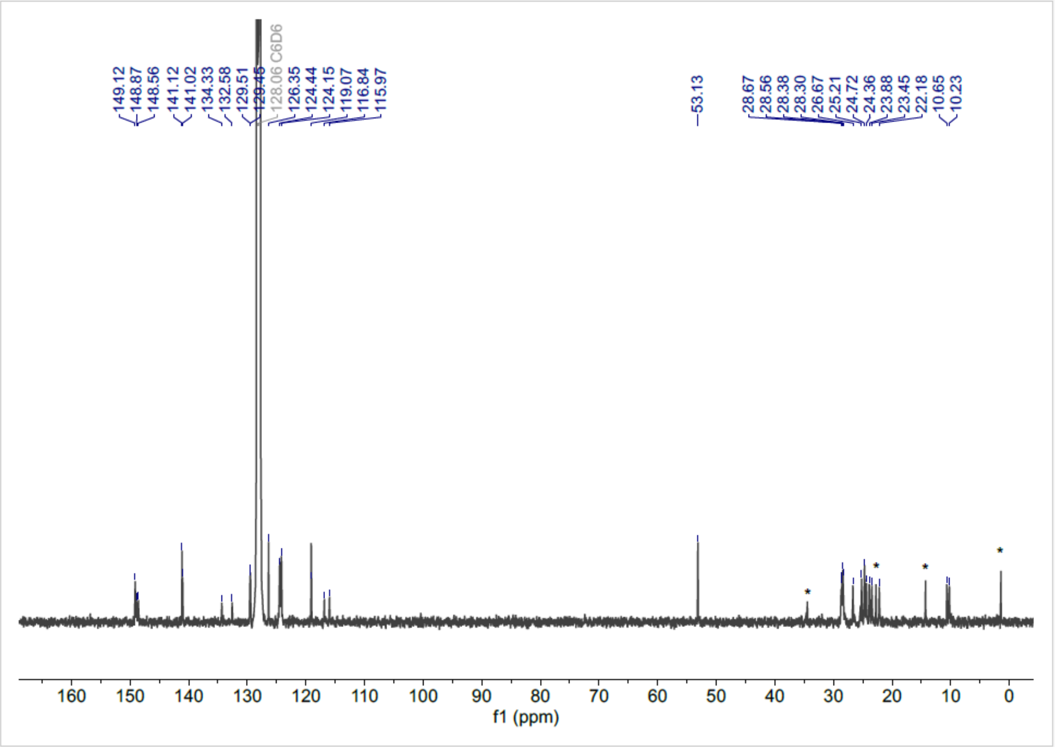

Figure S50. $^{13}C\{^1H\}$ NMR spectrum of compound $\mathbf{6^N}$ (101 MHz, C_6D_6, 298 K). * Corresponds to residual peaks of pentane and silicone grease.

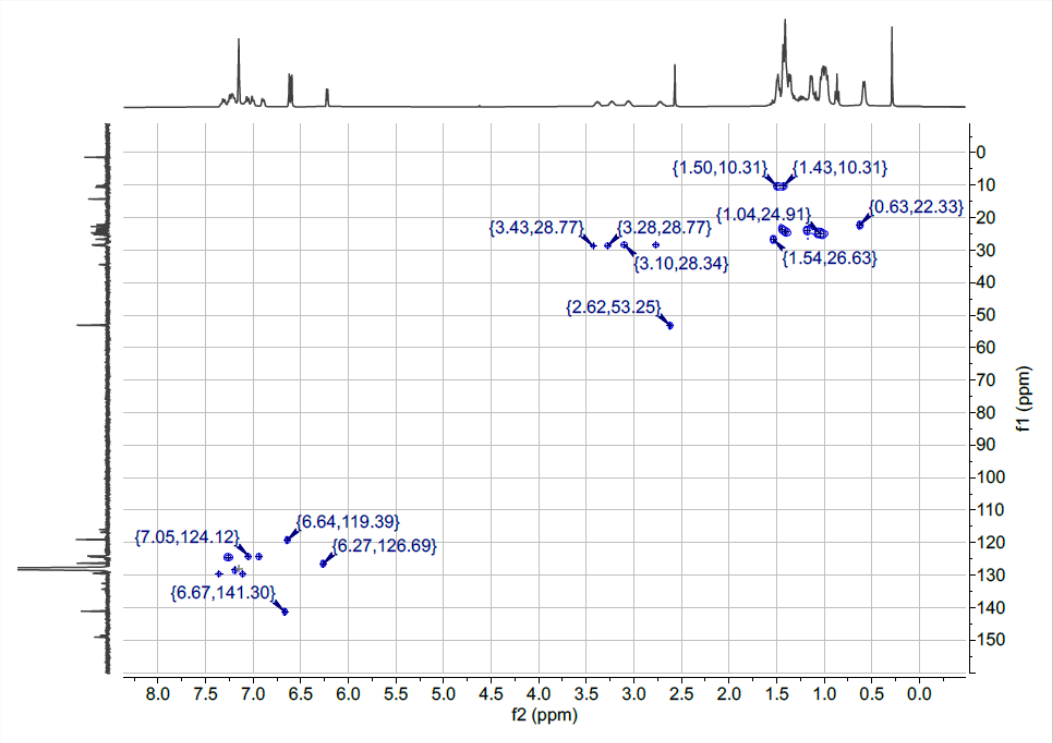

Figure S51. $^1H/^{13}C$ HSQC NMR spectrum of compound $\mathbf{6^N}$ (C_6D_6, 298 K).

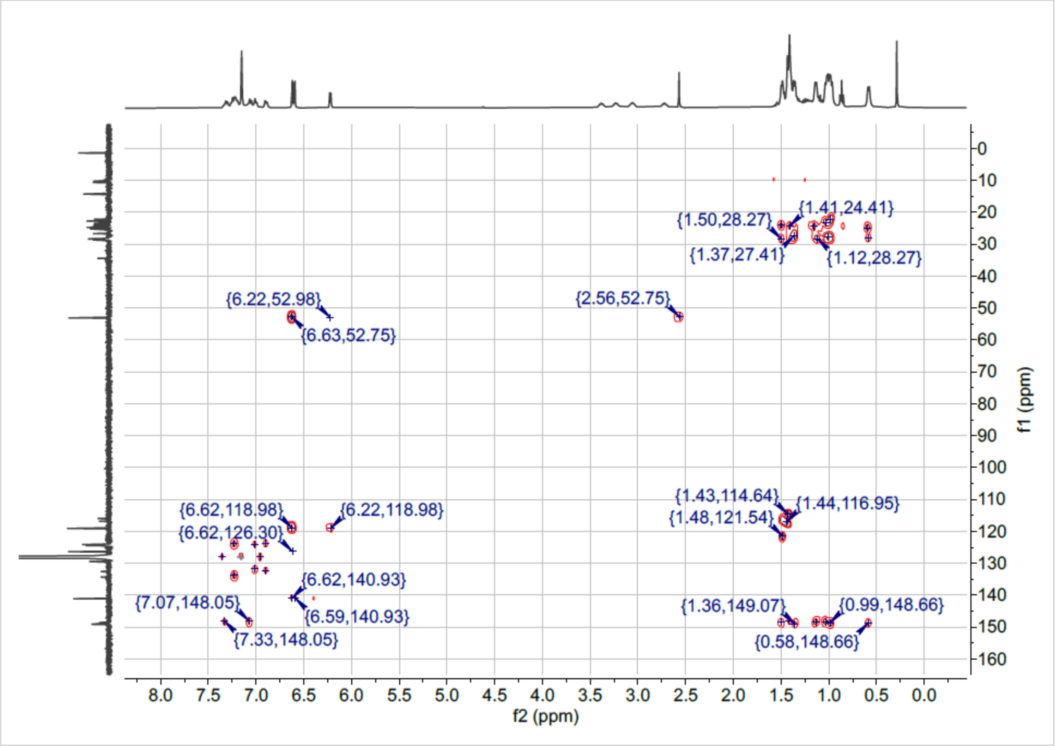

Figure S52. $^{1}H/^{13}C$ HMBC NMR spectrum of compound **6^{N}** (C_6D_6, 298 K).

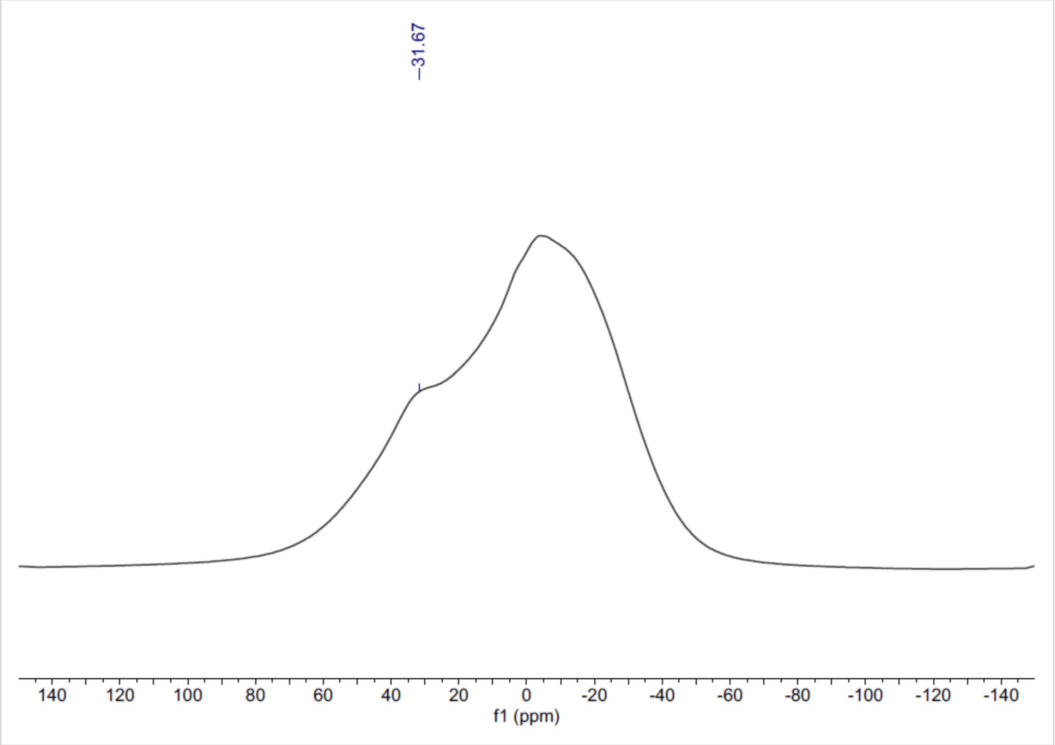

Figure S53. ^{11}B NMR spectrum of compound **6^{N}** (128 MHz, C_6D_6, 298 K).

1.2.12 Synthesis of compound 7^{C}-CF_3

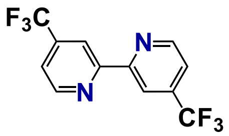

Compound **6^{C}-CF_3** (6.0 mg, 0.0051 mmol) and para-benzoquinone (6.6 mg, 0.0612 mmol) were dissolved in 0.4 mL of $CDCl_3$ in separate 4 mL vials and cooled to −30 °C for 20 min. The cold para-benzoquinone solution was added dropwise to the cold solution of compound **6^{C}-CF_3** under stirring. The reaction mixture was stirred at room temperature for 1 hour, during which the color gradually changed from orange-yellow to brownish-green, and a small amount of insoluble substance formed. The filtrate was transferred to a J-Young NMR tube for monitoring. The reaction was completed at room temperature within 24 hours. Compound **7^{C}-CF_3** and 4-(trifluoromethyl) pyridine (ratio: 1: 2) were observed. The ^{1}H and ^{19}F NMR signals were consistent with those reported in the literature.[6] **7^{C}-CF_3** was not isolated, and the yield was determined by crude ^{1}H NMR analysis using mesitylene as an internal standard (or by crude ^{19}F NMR analysis using fluorobenzene as an internal standard).

^{1}H NMR (400 MHz, $CDCl_3$, 298 K): δ (ppm) 8.89 (d, *J* = 5.0 Hz, 2H, NC*H*=CH), 8.73 (s, 2H, NC=C*H*), 7.59 (d, *J* = 5.0 Hz, 2H, N=CHC*H*).

^{19}F NMR (376 MHz, $CDCl_3$, 298 K): δ [ppm] −64.83 (s).

GC-MS: *m*/*z* for $C_{12}H_6F_6N_2$, Calcd: 292.0; Found: 292.0.

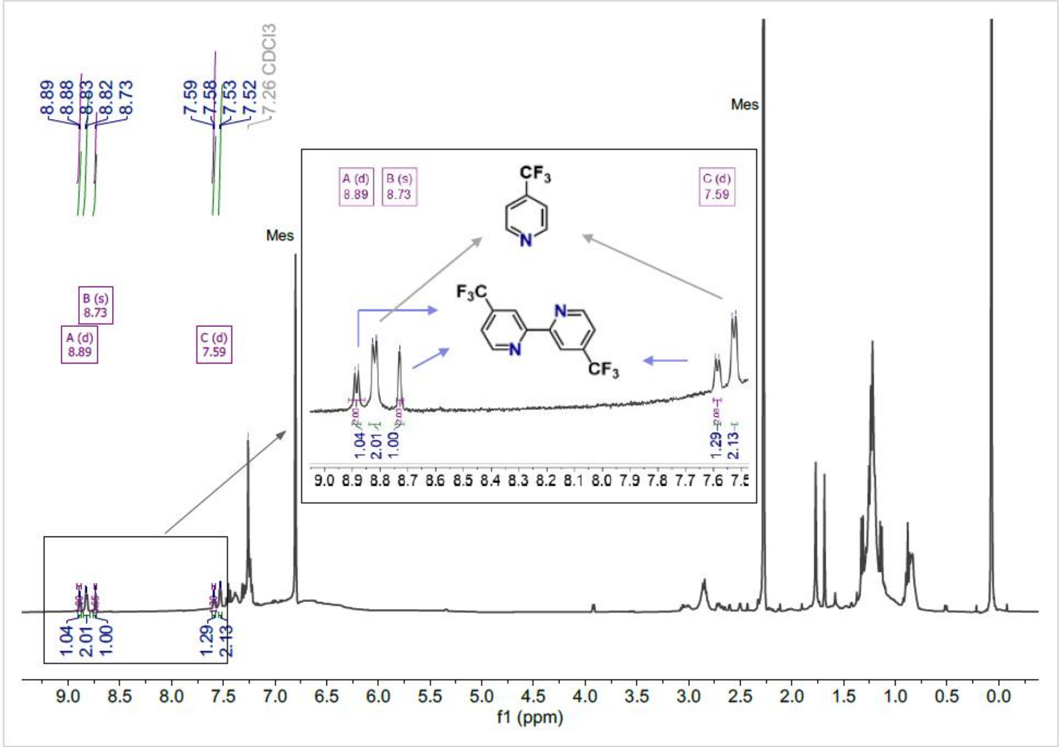

Figure S54. ^{1}H NMR spectrum for the reaction of compound **6^{C}-CF_3** with 12 equivalents of para-benzoquinone after 24 h (400 MHz, $CDCl_3$, 298 K).

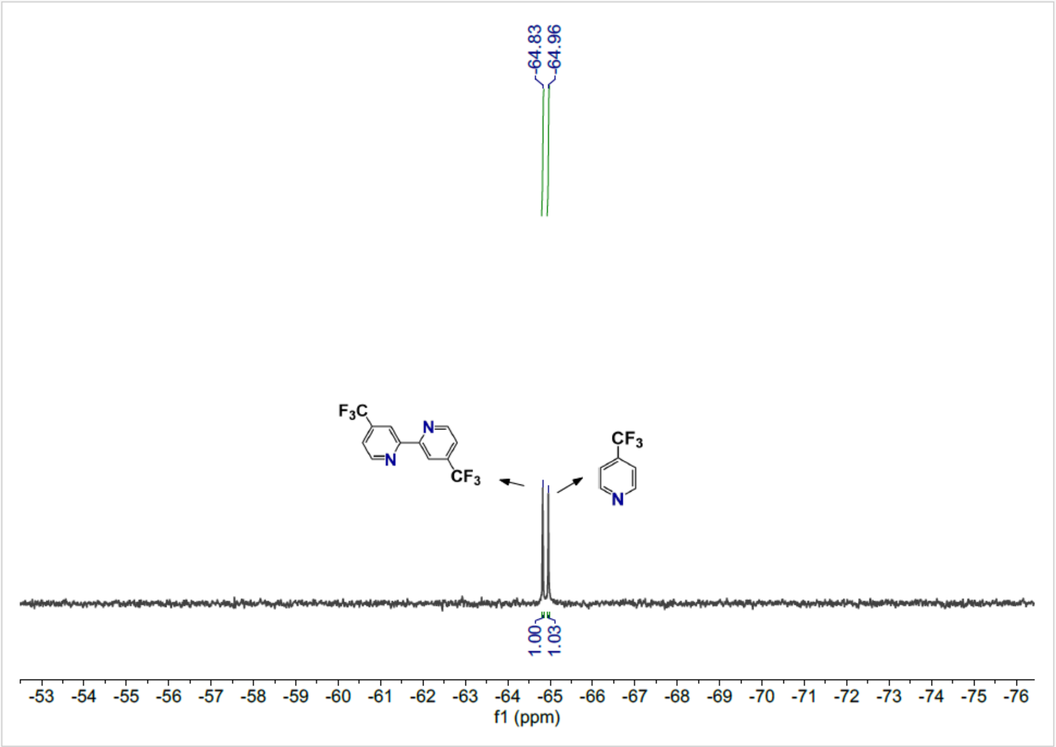

Figure S55. ^{19}F NMR spectrum for the reaction of compound **6^C-CF_3** with 12 equivalents of para-benzoquinone after 24 h (400 MHz, $CDCl_3$, 298 K).

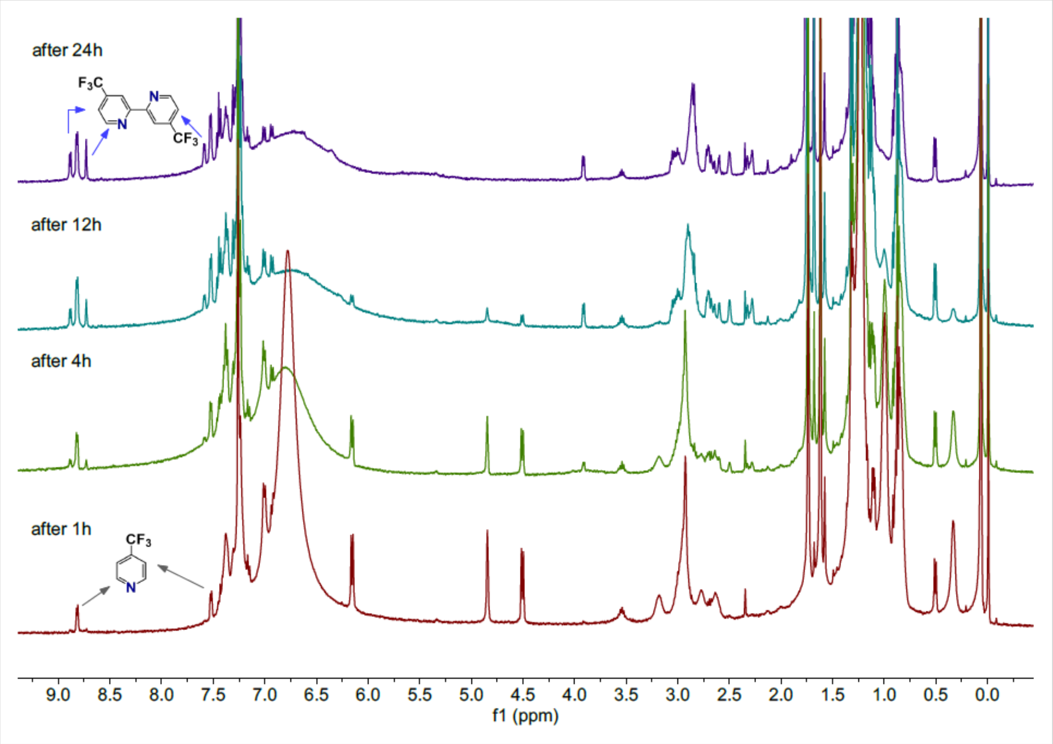

Figure S56. Stacked ^{1}H NMR spectra for the reaction of compound **6^C-CF_3** with 12 equivalents of para-benzoquinone at room temperature after 1, 4, 12, and 24 h (400 MHz, $CDCl_3$, 298 K).

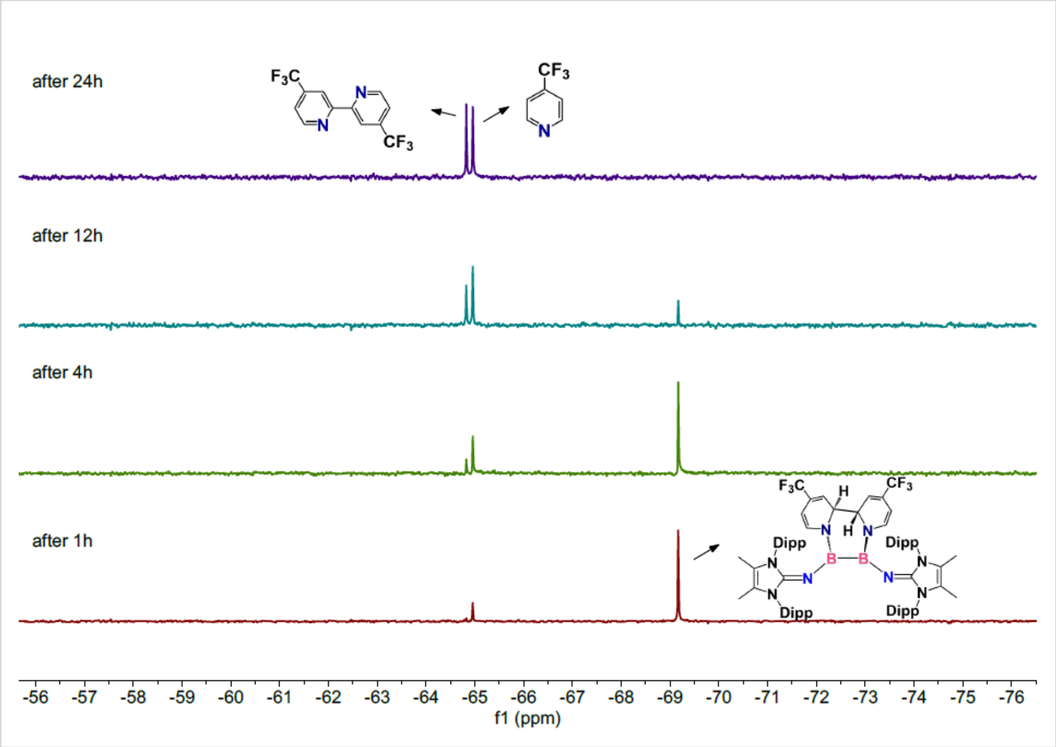

Figure S57. Stacked ^{19}F NMR spectra for the reaction of compound **6^{C}-CF_3** with 12 equivalents of para-benzoquinone at room temperature after 1, 4, 12, and 24 h (400 MHz, $CDCl_3$, 298 K).

2. Crystallographic Section

2.1 General considerations

The X-ray intensity data were collected on an X-ray single crystal diffractometer Bruker Photon CMOS system, with a rotating anode (Bruker TXS or an IMS microsource) with Mo Kα radiation (λ = 0.71073 Å) or Cu Kα radiation (λ = 1.54184 Å) and a Helios mirror optic by using the APEX III software package.[7] The measurement was performed on single crystals coated with Fomblin®Y as perfluorinated ether. The crystals were fixed on the top of a micro-sampler, transferred to the diffractometer and frozed under a stream of cold nitrogen (100 K). A matrix scan was used to determine the initial lattice parameters. Reflections were merged and corrected for Lorenz and polarization effects, scan speed, and background using SAINT.[8] Absorption corrections, including odd and even ordered spherical harmonics were performed using SADABS.[8] Space group

assignments were based upon systematic absences, E statistics, and successful refinement of the structures. Structures were solved by direct methods with the aid of successive difference Fourier maps and were refined against all data using the APEX III software in conjunction with SHELXL-2014[9] and SHELXLE.[10] Methyl hydrogen atoms were refined as part of rigid rotating groups, with a C–H distance of 0.98 Å and Uiso(H) = 1.5·Ueq(C). Other H atoms were placed in calculated positions and refined using a riding model, with methylene and aromatic C–H distances of 0.99 and 0.95 Å, respectively, and Uiso(H) = 1.2·Ueq(C). If not stated otherwise, non-hydrogen atoms were refined with anisotropic displacement parameters. Full-matrix least-squares refinements were carried out by minimizing $\Delta w(F_o^2–F_c^2)^2$ with SHELXL-97[11] weighting scheme. Neutral atom scattering factors for all atoms and anomalous dispersion corrections for the non-hydrogen atoms were taken from International Tables for Crystallography.[12] The images of the crystal structures were generated by Mercury.[13] The CCDC numbers 2498571-2498576 contain the supplementary crystallographic data for the structures. The data can be obtained free of charge from the Cambridge Crystallographic Data Centre via https://www.ccdc.cam.ac.uk/structures/.

2.2 SC-XRD data and structure refinement

	**2**	**3**	**4**
CCDC number	2498571	2498576	2498574
formula	$C_{58}H_{80}B_2N_6$	$C_{64}H_{81}B_2N_6F_5$	$C_{58}H_{80}B_2N_6$
formula weight	882.90	1050.96	882.90
temperature/K	100	100	100
crystal system	Triclinic	Monoclinic	Monoclinic
space group	P-1	P 2_1/c	P 2_1/c
a/Å	10.8635(11)	11.3573(8)	12.7651(18)
b/Å	14.5051(14)	28.244(2)	18.221(3)
c/Å	17.3112(15)	18.4271(16)	23.242(3)
α/°	91.410(3)	90	90
β/°	103.593(3)	90.824(3)	98.688(5)
γ/°	94.488(4)	90	90
volume/$Å^3$	2640.7(4)	5910.4(8)	5343.8(13)
Z	2	4	4
ρ_{calc}/g•cm^{-3}	1.110	1.181	1.097
μ/mm^{-1}	0.064	0.080	0.063
F(000)	960.0	2248.0	1920.0
2*θ* range/°	4.588 to 52.754	4.422 to 55.174	4.192 to 54.204
index ranges	−13<=*h*<=13	−14<=*h*<=13	−16<=*h*<=16
	−18<=*k*<=18	−36<=*k*<=36	−23<=*k*<=23
	−21<=*l*<=21	−23<=*l*<=23	−29<=*l*<=29
reflections collected	113229	207292	227750
independent reflections	10763	13618	11766
R_{int}	0.0367	0.0938	0.0953
GooF on F^2	1.018	1.100	1.142
final *R* indices	R_1 = 0.0421,	R_1 = 0.0579,	R_1 = 0.0810,
[*I*>=2σ (*I*)]	wR_2 = 0.1052	wR_2 = 0.1510	wR_2 = 0.2049
final *R* indices	R_1 = 0.0462,	R_1 = 0.0704,	R_1 = 0.1017,
[all data]	wR_2 = 0.1091	wR_2 = 0.1601	wR_2 = 0.2201
completeness	0.996	0.995	0.998

$R_1 = \Sigma||F_o| - |F_c||/\Sigma F_o|$, $wR_2 = [\Sigma w(F_o^2-F_c^2)^2/\Sigma w(F_o^2)^2]^{1/2}$

	**5-S^{Me}**	**6^{C}-H**	**6^{C}-CF$_3$**
CCDC number	2498575	2498573	2498572
formula	$C_{70}H_{110}N_6S_2B_2$	$C_{73}H_{95}B_2N_9$	$C_{70}H_{88}B_2F_6N_8$
formula weight	1121.37	1120.19	1177.10
temperature/K	100	100	100
crystal system	Monoclinic	Triclinic	Monoclinic
space group	$P2_1/n$	P-1	C2/c
a/Å	12.7133(6)	12.8092(4)	16.9827(14)
b/Å	24.7963(15)	14.4316(5)	15.9751(13)
c/Å	22.2662(13)	18.3718(6)	25.253(2)
α/°	90	79.5320(10)	90
β/°	103.876(2)	78.5690(10)	108.042(3)
γ/°	90	80.6560(10)	90
volume/Å^3	6814.4(7)	3245.14(19)	6514.1(10)
Z	4	2	4
ρ_{calc}/g•cm^{-3}	1.093	1.146	1.200
μ/mm^{-1}	0.121	0.067	0.082
F(000)	2456.0	1212.0	2512.0
2θ range/°	3.768 to 50.054	3.958 to 55.040	4.368 to 54.978
index ranges	−15 ≤ h ≤ 15	−16<=*h*<=16	−22<=*h*<=22
	−29 ≤ k ≤ 29	−18<=*k*<=18	−20<=*k*<=20
	−26 ≤ l ≤ 26	−23<=*l*<=23	−32<=*l*<=32
reflections collected	367380	183591	79493
independent reflections	11990	14897	7411
R_{int}	0.0461	0.0399	0.0828
GooF on F^2	1.046	1.064	1.056
final *R* indices	R_1 = 0.0453,	R_1 = 0.0448,	R_1 = 0.0411,
[*I*>=2σ (*I*)]	wR_2 =0.1238	wR_2 =0.1050	wR_2 = 0.1138
final *R* indices	R_1=0.0504,	R_1=0.0494,	R_1 = 0.0542,
[all data]	wR_2 =0.1302	wR_2 =0.1079	wR_2 = 0.1225
completeness	0.996	0.998	0.993

$R_1 = \Sigma||F_o| - |F_c||/\Sigma F_o|$, $wR_2 = [\Sigma w (F_o^2-F_c^2)^2/\Sigma w (F_o^2)^2]^{1/2}$

2.3 SC-XRD structures

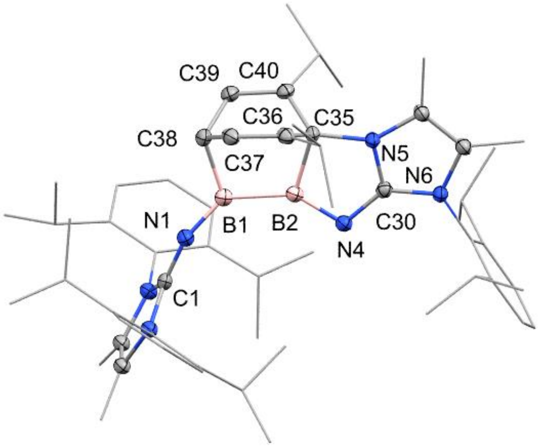

Figure S58. The thermal ellipsoid drawing of compound **2** at 50% probability level. Hydrogen atoms are omitted, and parts of groups are represented as wireframes for the sake of clarity. Selected bond lengths (Å): B1−B2 = 1.7204(18), B1−C38 = 1.6463(17), B2−C35 = 1.6655(16), C35−C36 = 1.5195(16), C36−C37 = 1.3408(17), C37−C38 = 1.5091(17), C38−C39 = 1.5041(16), C39−C40 = 1.3402(16), C40−C35 = 1.5199(16), B1−N1 = 1.3652(16), N1−C1 = 1.2686(15), B2−N4 = 1.4291(16), N4−C30 = 1.3279(15), C30−N5 = 1.3655(14), N5−C35 = 1.4611(14), N6−C30 = 1.3618(14).

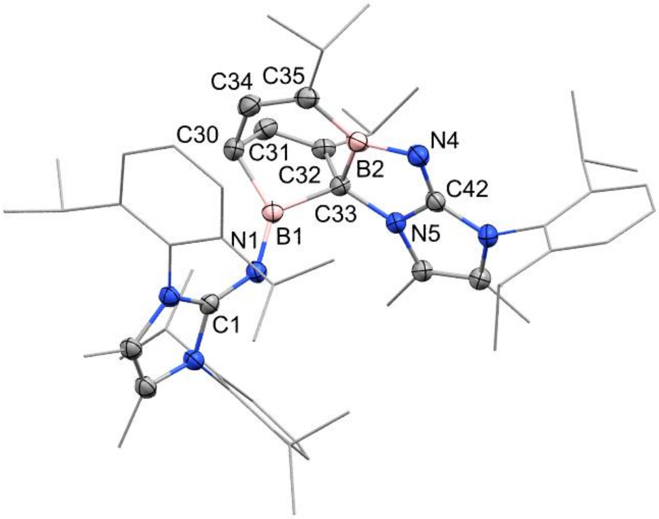

Figure S59. The thermal ellipsoid drawing of compound **3** at 50% probability level. Hydrogen atoms are omitted, and parts of groups are represented as wireframes for the sake of clarity. Selected bond lengths (Å): B1−C30 = 1.621(3), B1−C33 = 1.610(2), B2−C33 = 1.610(3), B2−C35

= 1.550(3), C30−C31 = 1.524(3), C31−C32 = 1.336(2), C32−C33 = 1.524(2), C30−C34 = 1.522(3), C34−C35 = 1.352(3), B1−N1 = 1.365(2), B2−N4 = 1.433(3), N4−C42 = 1.326(2), N5−C33 = 1.455(2), N5−C42 = 1.356(2).

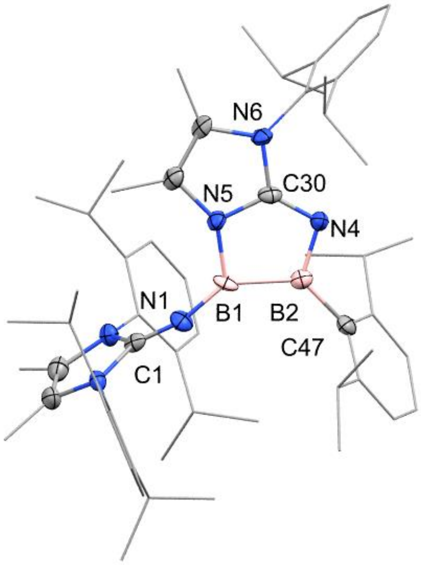

Figure S60. The thermal ellipsoid drawing of compound **4** at 50% probability level. Hydrogen atoms are omitted, and parts of groups are represented as wireframes for the sake of clarity. Selected bond lengths (Å): B1−B2 = 1.768(4), B1−N5 = 1.523(4), B2−N4 = 1.428(4), N4−C30 = 1.324(3), N5−C30 = 1.382(3), N6−C30 = 1.356(3).

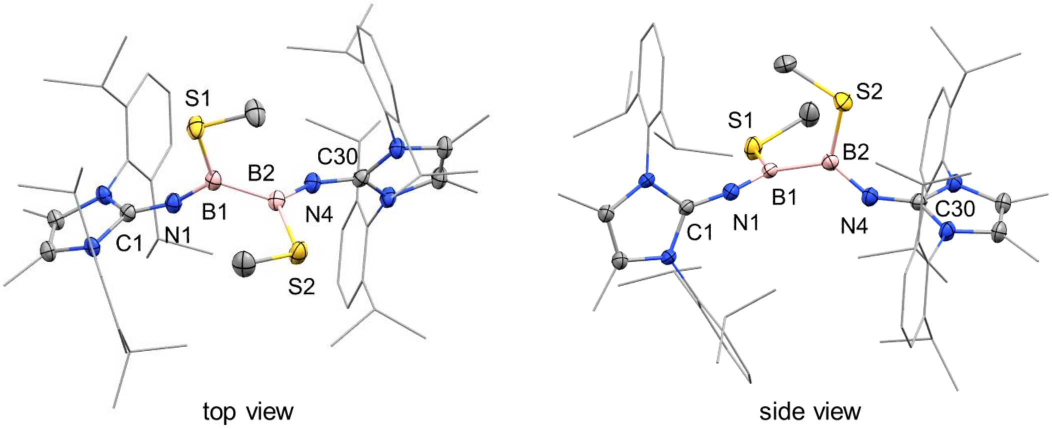

Figure S61. The thermal ellipsoid drawing of compound **5-S^{Me}** at 50% probability level. Hydrogen atoms are omitted, and parts of groups are represented as wireframes for the sake of clarity. Selected bond lengths (Å) and torsion angles (º): B1−B2 = 1.717(3), B1−S1 = 1.855(2), B2−S2 =

1.850(2), B1−N1 = 1.367(3), B2−N4 = 1.382(3), N1−C1 = 1.269(3), N4−C30 = 1.283(3); S1−B1−B2−S2 = 88.98(17).

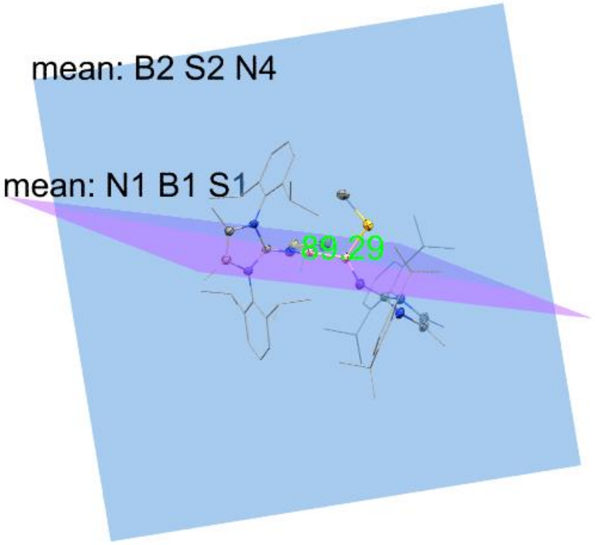

Figure S62. The dihedral angle between the plane N1B1S1 and plane N4B2S2. The 89.29° of dihedral angle suggests the two planes are orthogonal.

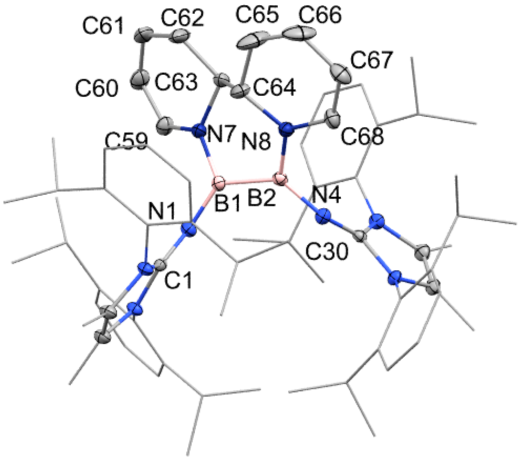

Figure S63. The thermal ellipsoid drawing of compound **6^{C}-H** at 50% probability level. Hydrogen atoms are omitted, and parts of groups are represented as wireframes for the sake of clarity. Selected bond lengths (Å): B1−B2 = 1.7121(17), B1−N7 = 1.4634(15), B2−N8 = 1.4564(15), N7−C59 = 1.3865(15), C59−C60 = 1.3462(17), C60−C61 = 1.438(2), C61−C62 = 1.332(2), C62−C63 = 1.5116(17), C63−C64 = 1.5447(16), C63−N7 = 1.4868 (14).

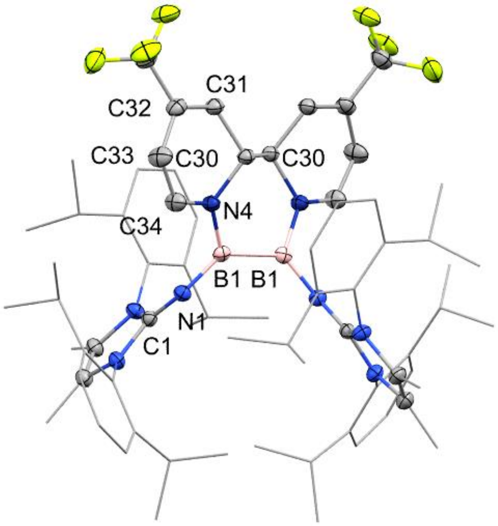

Figure S64. The thermal ellipsoid drawing of compound **6^{C}-CF_3** at 50% probability level. Hydrogen atoms are omitted, and parts of groups are represented as wireframes for the sake of clarity. Selected bond lengths (Å): B1−B1′ = 1.709(3), B1−N4 = 1.4688(16), N4−C34 = 1.3825(15), C34−C33 = 1.3464(18), C33−C32 = 1.4443(18), C32−C31 = 1.3387(18), C31−C30 = 1.5152(16), C30−C30′ = 1.546(2).

3. Computational Details

Calculations were carried out with ORCA 5.0.4.[14] Geometry optimizations were carried using the r^2SCAN-3c composite method,[15] utilizing the regularized and restored SCAN functional,[16,17] geometrical counterpoise correction gCP,[18] the atom-pairwise dispersion correction based on tight binding partial charges (D4),[19–21] the def2-mTZVPP basis set and def2-mTZVPP/J auxiliary basis set.[15] The optimized geometries were verified as minima or transition states by analytical frequency calculations. The transition states were additionally verified by IRC calculations. Single point calculations of the optimized geometries were carried out at the r^2SCAN-3c level in benzene or tetrahydrofuran using the SMD solvation module[22] to obtain electrostatic contribution and the cavity term to account for the solvent effects. To get more accurate electronic energies for the mechanistic investigations, single point calculations of the r^2SCAN-3c optimized geometries were carried using the PW6B95[23] functional, with D4 dispersion correction, the def2-QZVPP[24] basis set and def2/J[25] auxiliary basis set. The level at which the free energies were obtained are denoted

as (SMD=Benzene or Tetrahydrofuran) PW6B95-D4/def2-QZVPP//r^{2}SCAN-3c. Properties calculations and NBO analysis were carried out at the PBE0[27]-D4/def2-TZVP[24]//r^{2}SCAN-3c level of theory. NBO analysis was carried out using NBO 7 software.[28] Predictions of ^{11}B NMR chemical shifts were carried out at the B3LYP/6-311G(d)// r^{2}SCAN-3c level of theory on previously optimized structures using the Gauge Independent Atomic Orbital (GIAO) method.[29] The absolute isotropic shielding values obtained were scaled against the shielding value for $BF_3{\cdot}OEt_2$ (102.02 ppm). The summary of the thermochemistry results is presented in Table S2, S3.

Table S2 Calculated energies (E_h). Thermochemistry at 298.15 K.

E_{PW6B95} - electronic energy at the PW6B95-D4/def2-QZVPP//r^{2}SCAN-3c level; G-E_{el} - Gibbs energy minus the electronic energy at the r^{2}SCAN-3c//r^{2}SCAN-3c level; G_{cds} - cavity term and G_{enp} - electrostatic contribution at the r^{2}SCAN-3c(SMD=Benzene)//r^{2}SCAN-3c level; G_{conc} - concentration-induced free-energy shift (G_{conc} = RTln(24.5)); G_{PW6B95} - free energy at the (SMD=Benzene)PW6B95-D4/def2-QZVPP//r^{2}SCAN-3c level ($G_{PW6B95} = E_{PW6B95}$ + [G-E_{el}] + G_{cds} + G_{enp} + G_{conc}).

KEB	Compound	E_{PW6B95}	G-E_{el}(298.15K)	G_{cds}(Benzene)	G_{enp}(Benzene)	G_{conc}(298.15K)	G_{PW6B95}
1848234	**1_{INT}^S**	-2641.34624	1.16904	-0.02552	-0.02947	0.00302	-2640.22916
1871643	**1_{INT}^T**	-2641.34822	1.16774	-0.02869	-0.02833	0.00302	-2640.23448
1846525	**TS(1_{INT}^S-A^S)^S**	-2641.33857	1.16920	-0.02541	-0.02930	0.00302	-2640.22106
1895693	**TS(1_{INT}^T-A^T)^T**	-2641.33032	1.16708	-0.02684	-0.02860	0.00302	-2640.21566
1848233	**A**	-2641.35342	1.16925	-0.02879	-0.03030	0.00302	-2640.24024
1894113	**A^T**	-2641.33934	1.16729	-0.02640	-0.03026	0.00302	-2640.22569
1850052	**TS(A-B)**	-2641.35585	1.17079	-0.02808	-0.02828	0.00302	-2640.23839
1846496	**B**	-2641.36073	1.17029	-0.02792	-0.02865	0.00302	-2640.24400
1850086	**TS(B-2)**	-2641.35690	1.17295	-0.02505	-0.02864	0.00302	-2640.23462
1849643	**2**	-2641.38974	1.17327	-0.02455	-0.03005	0.00302	-2640.26804
1850150	**2^T**	-2641.32544	1.17070	-0.02346	-0.02845	0.00302	-2640.20362
1852541	**TS(2^T-1_{INT}^T)^T**	-2641.31961	1.16977	-0.02369	-0.02876	0.00302	-2640.19927
1861366	**TS(1_{INT}^T-C^T)^T**	-2641.32957	1.16762	-0.02707	-0.02773	0.00302	-2640.21372
1849550	**C^T**	-2641.33738	1.16852	-0.02590	-0.02913	0.00302	-2640.22086
1846489	**TS(C^T-D^T)^T**	-2641.31611	1.16518	-0.02561	-0.02916	0.00302	-2640.20268
1848924	**D^T**	-2641.36421	1.16714	-0.02890	-0.02832	0.00302	-2640.25127
1848944	**E**	-2641.41705	1.16942	-0.03335	-0.02942	0.00302	-2640.30739
1846524	**TS(E-F)**	-2641.41259	1.17185	-0.03013	-0.02887	0.00302	-2640.29673
1848955	**F**	-2641.43849	1.17540	-0.02498	-0.02828	0.00302	-2640.31332
1848110	**G**	-2641.44888	1.17510	-0.02308	-0.02705	0.00302	-2640.32089
1890062	**TS(G-H)**	-2641.40668	1.17081	-0.02839	-0.02915	0.00302	-2640.29038
1849611	**H**	-2641.41824	1.17090	-0.03127	-0.03093	0.00302	-2640.30652
1848096	**TS(H-4)**	-2641.41849	1.17207	-0.02980	-0.03035	0.00302	-2640.30355
1871178	**4**	-2641.45785	1.17687	-0.02425	-0.02915	0.00302	-2640.33136
3380523_1	**TS(1_{INT}-I)**	-2874.01084	1.26653	-0.02792	-0.03054	0.00302	-2872.79975
3382438_2	**I**	-2874.02985	1.26921	-0.02883	-0.02962	0.00302	-2872.81607
3380524_2	**TS(I-J)**	-2874.02525	1.27100	-0.02807	-0.03028	0.00302	-2872.80957
3380003_1	**J**	-2874.07607	1.27406	-0.02566	-0.02958	0.00302	-2872.85423

Table S3 Calculated energies (E_h). Thermochemistry at 298.15 K.

E_{PW6B95} - electronic energy at the PW6B95-D4/def2-QZVPP//r^{2}SCAN-3c level; G-E_{el} - Gibbs energy minus the electronic energy at the r^{2}SCAN-3c//r^{2}SCAN-3c level; G_{cds} - cavity term and G_{enp} - electrostatic contribution at the r^{2}SCAN-3c(SMD=Benzene)//r^{2}SCAN-3c level; G_{conc} - concentration-induced free-energy shift (G_{conc} = RTln(24.5)); G_{PW6B95} - free energy at the (SMD=THF)PW6B95-D4/def2-QZVPP//r^{2}SCAN-3c level ($G_{PW6B95} = E_{PW6B95}$ + [G-E_{el}] + G_{cds} + G_{enp} + G_{conc}).

KEB	Compound	E_{PW6B95}	G-E_{el}(298.15K)	G_{cds}(THF)	G_{enp}(THF)	G_{conc}(298.15K)	G_{PW6B95}
1848234	**1_{INT}^{S}**	-2641.34624	1.16904	-0.05019	-0.01545	0.00302	-2640.23983
1871643	**1_{INT}^{T}**	-2641.34822	1.16774	-0.05767	-0.01472	0.00302	-2640.24984
1846525	**TS(1_{INT}^{S}-A)**	-2641.33857	1.16920	-0.04926	-0.01543	0.00302	-2640.23104
1895693	**TS(1_{INT}^{T}-A^{T})^{T}**	-2641.33033	1.16708	-0.05223	-0.01498	0.00302	-2640.22744
1848233	**A**	-2641.35341	1.16925	-0.05564	-0.01627	0.00302	-2640.25305
1894113	**A^{T}**	-2641.33934	1.16729	-0.05115	-0.01621	0.00302	-2640.23638
1850052	**TS(A-B)**	-2641.35585	1.17079	-0.05413	-0.01505	0.00302	-2640.25122
1846496	**B**	-2641.36073	1.17029	-0.05354	-0.01497	0.00302	-2640.25592
1850086	**TS(B-2)**	-2641.35690	1.17295	-0.04755	-0.01530	0.00302	-2640.24378
1849643	**2**	-2641.38973	1.17327	-0.04673	-0.01646	0.00302	-2640.27662
2249307_1	**TS(2-3)**	-2641.34724	1.17200	-0.04618	-0.01613	0.00302	-2640.23453
2255530_1	**3**	-2641.43022	1.17573	-0.04472	-0.01508	0.00302	-2640.31127
2248887_2	**TS(2-INT1)**	-2890.11717	1.26130	-0.04980	-0.01536	0.00302	-2888.91800
2214949_1	**INT1**	-2890.11717	1.26130	-0.04980	-0.01536	0.00302	-2888.91800
2265372_1	**TS(INT1-INT2)**	-2890.09988	1.25928	-0.04496	-0.01582	0.00302	-2888.89835
3380005_2	**TS(INT1-INT2)$_{Py-CF3}$**	-3227.68625	1.25939	-0.04495	-0.01411	0.00302	-3226.48290
2234385_3	**INT2**	-2890.12871	1.25897	-0.05292	-0.01560	0.00302	-2888.93524
2242635_1	**TS(INT2-INT3)**	-3138.84131	1.34681	-0.05762	-0.01871	0.00302	-3137.56781
2211651_4	**INT3**	-3138.87901	1.35067	-0.04884	-0.01865	0.00302	-3137.59281
2205826_1	**TS(INT3-6^{C}-H)**	-3138.87783	1.35109	-0.04831	-0.01838	0.00302	-3137.59042
2211651_3	**6^{C}-H**	-3138.92047	1.35412	-0.04614	-0.01874	0.00302	-3137.62821

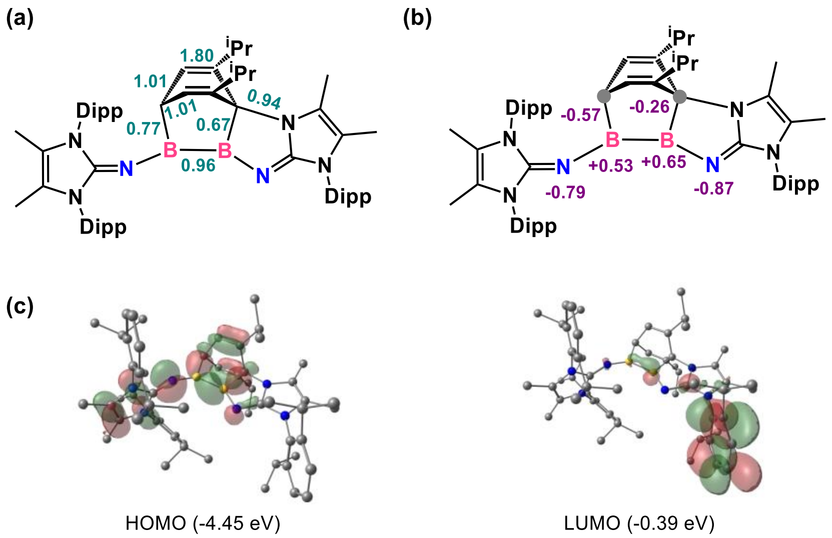

Figure S65. (a) Selected WBIs in compound **2**; (b) NPA charges distribution in compound **2**; (c) Plots of selected frontier molecular orbitals of **2**. Isovalue = 0.04.

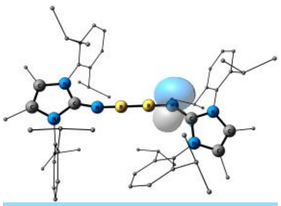

NBO 69. LP(1) N144 occ. 1.64
sp7.45

Acceptor	E(2) (kcal mol^{-1})
241. LV (1) B2	86.8
331. BD*(1) C72-N145	24.9
332. BD*(1) C72-N146	13.2
1417. RY (1) C72	1.6
246. BD*(1) B2-N144	1.5

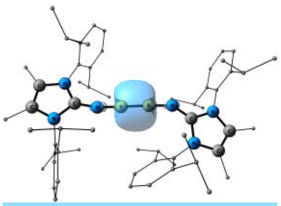

NBO 72. BD(1) B1-B2 occ. 1.98
50.16% B1 sp0.76
49.84% B2 sp0.76

Acceptor	E(2) (kcal mol^{-1})
2372. RY (2) N141	8.1
2450. RY (2) N144	4.9
2451. RY (3) N144	4.3
247. BD*(1) C3-N141	3.4
329. BD*(1) C72-N144	2.3

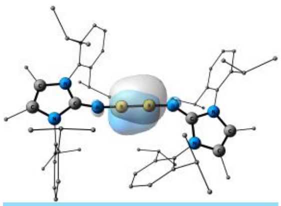

NBO 73. BD(2) B1-B2 occ. 1.67
50.47% B1 sp99.99
49.53% B2 sp99.99

Acceptor	E(2) (kcal mol^{-1})
248. BD*(2) C3-N141	43.7
330. BD*(2) C72-N144	36.6
2371. RY (1) N141	2.9
2449. RY (1) N144	2.2
290. BD*(1) C35-H36	2.0

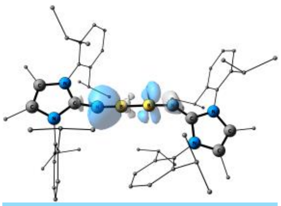

NBO 74. BD(1) B1-N141 occ. 1.98
23.31% B1 sp1.36
76.69% N141 sp0.74

Acceptor	E(2) (kcal mol^{-1})
247. BD*(1) C3-N141	5.9
464. RY (2) C3	3.6
250. BD*(1) C3-N143	1.9
439. RY (3) B2	1.7
242. BD*(1) B1-B2	1.6

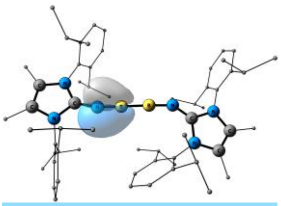

NBO 75. BD(2) B1-N141 occ. 1.89
16.15% B1 sp99.99
83.85% N141 sp99.99

Acceptor	E(2) (kcal mol^{-1})
249. BD*(1) C3-N142	16.9
250. BD*(1) C3-N143	16.4
241. LV (1) B2	5.5
466. RY (4) C3	1.0
465. RY (3) C3	0.8

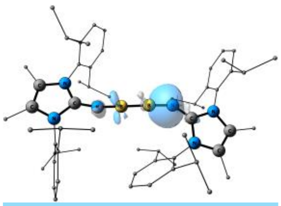

NBO 76. BD(1) B2-N144 occ. 1.98
23.75% B2 sp1.35
76.25% N144 sp1.06

Acceptor	E(2) (kcal mol^{-1})
332. BD*(1) C72-N146	6.0
329. BD*(1) C72-N144	4.7
1417. RY (1) C72	2.5
413. RY (3) B1	1.5
1419. RY (3) C72	1.3

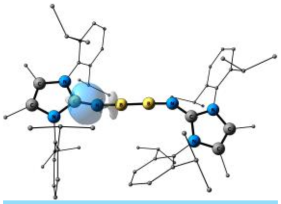

NBO 77. BD(1) C3-N141 occ. 1.98
40.94% C3 sp1.56
59.06% N141 sp1.38

Acceptor	E(2) (kcal mol^{-1})
244. BD*(1) B1-N141	4.6
412. RY (2) B1	2.4
250. BD*(1) C3-N143	1.5
256. BD*(1) C5-N143	1.4
2425. RY (3) N143	1.3

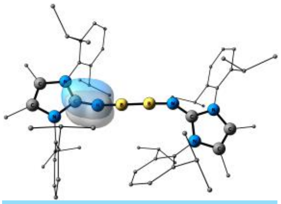

NBO 78. BD(2) C3-N141 occ. 1.92
31.71% C3 sp1.00
68.29% N141 sp99.99

Acceptor	E(2) (kcal mol^{-1})
243. BD*(2) B1-B2	15.4
411. RY (1) B1	3.7
248. BD*(2) C3-N141	3.2
2397. RY (1) N142	0.7
2423. RY (1) N143	0.6

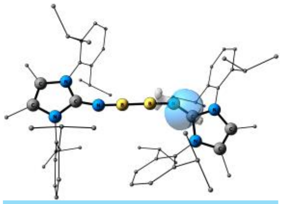

NBO 159. BD(1) C72-N144 occ. 1.98
41.42% C72 sp1.56
58.58% N144 sp1.52

Acceptor	E(2) (kcal mol^{-1})
246. BD*(1) B2-N144	3.6
438. RY (2) B2	2.2
332. BD*(1) C72-N146	1.5
338. BD*(1) C74-N146	1.5
2503. RY (3) N146	1.4

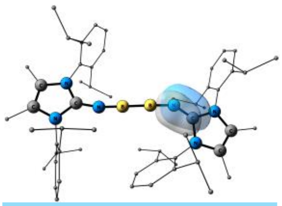

NBO 160. BD(2) C72-N144 occ. 1.91
31.64% C72 sp1.00
68.36% N144 sp1.00

Acceptor	E(2) (kcal mol^{-1})
243. BD*(2) B1-B2	15.3
437. RY (1) B2	3.4
330. BD*(2) C72-N144	3.4
241. LV (1) B2	1.6
2501. RY (1) N146	0.7

Figure S66. Plots of selected natural bond orbitals (NBOs) of **1_{INT}^{S}** their atomic orbital compositions and five largest donor-acceptor interactions, according to the second-order perturbation theory.

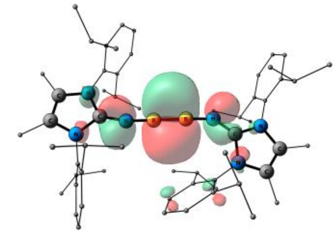

MO 241. LUMO -0.63 eV

Bonding	N.B.	A.B.
3.2%	47.8%	49.0%

-0.628*[245]	BD*(2) B1-N**	39.4%
-0.601*[241]	LV (1) B2	36.1%
0.266*[69]	LP(1) N*	7.1%

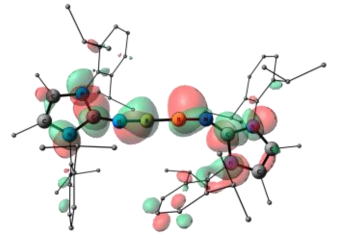

MO 250. LUMO+9 0.69 eV

Bonding	N.B.	A.B.
0.0%	99.9%	0.1%

-0.413*[248]	BD*(2) C3-N**	17.1%
-0.395*[330]	BD*(2) C72-N**	15.6%
0.379*[243]	BD*(2) B1-B2	14.4%

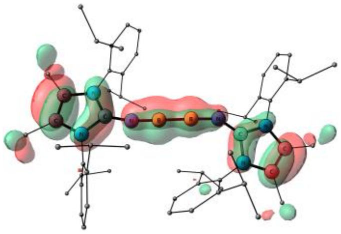

MO 238. HOMO-2 -5.85 eV

Bonding	N.B.	A.B.
67.2%	12.4%	20.4%

0.513*[82]	BD(2) C4-C5	26.3%
-0.393*[164]	BD(2) C73-C74	15.4%
-0.393*[73]	BD(2) B1-B2	15.4%
0.335*[248]	BD*(2) C3-N**	11.2%
-0.257*[330]	BD*(2) C72-N**	6.6%

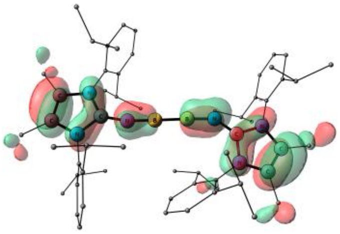

MO 239. HOMO-1 -5.48 eV

Bonding	N.B.	A.B.
55.9%	19.6%	24.5%

0.511*[164]	BD(2) C73-C74	26.1%
0.403*[82]	BD(2) C4-C5	16.2%
0.334*[330]	BD*(2) C72-N**	11.2%
0.261*[248]	BD*(2) C3-N**	6.8%
0.247*[70]	LP(1) N*	6.1%

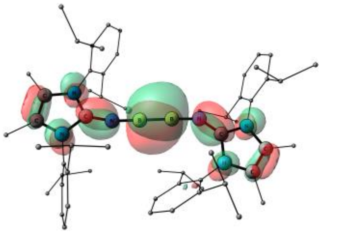

MO 240. HOMO -2.68 eV

Bonding	N.B.	A.B.
76.1%	17.5%	6.4%

0.739*[73]	BD(2) B1-B2	54.6%
0.267*[160]	BD(2) C72-N*	7.1%
0.267*[78]	BD(2) C3-N*	7.1%

Figure S67. Plots of selected canonical molecular orbitals of **1_{INT}^{S}** and their NBO analysis.

α spin NBOs

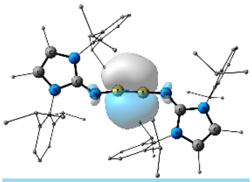

NBO 74. BD(2) B1-B2 occ. 0.97
50.07% B1 sp99.99
49.93% B2 sp99.99

Acceptor	E(2) (kcal mol^{-1})
247. BD*(1) C3-N141	2.4
329. BD*(1) C72-N144	2.1
2371. RY (1) N141	1.5
2449. RY (1) N144	1.2

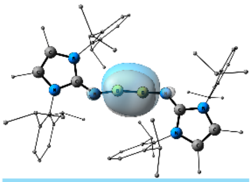

NBO 75. BD(3) B1-B2 occ. 0.89
50.64% B1 sp99.99
49.36% B2 sp1.00

Acceptor	E(2) (kcal mol^{-1})
248. BD*(2) C3-N141	14.3
330. BD*(2) C72-N144	12.5
2450. RY (2) N144	1.5
2372. RY (2) N141	1.5
285. BD*(1) C29-H31	1.1

α spin NBOs

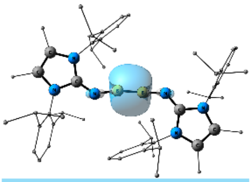

NBO 73. BD(1) B1-B2 occ. 0.99
49.73% B1 sp0.73
50.27% B2 sp0.76

Acceptor	E(2) (kcal mol^{-1})
2373. RY (3) N141	2.5
2451. RY (3) N144	2.4
2374. RY (4) N141	1.3
432. RY (22) B1	1.2
247. BD*(1) C3-N141	1.1

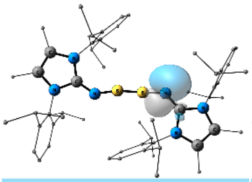

NBO 70. LP(1) N144 occ. 0.91
sp4.94

Acceptor	E(2) (kcal mol^{-1})
331. BD*(1) C72-N145	13.2
243. BD*(2) B1-B2	9.5
332. BD*(1) C72-N146	5.6
437. RY (1) B2	1.8
1417. RY (1) C72	1.2

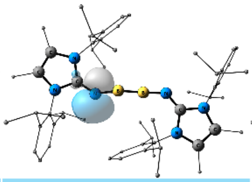

NBO 67. LP(1) N141 occ. 0.90
sp5.68

Acceptor	E(2) (kcal mol^{-1})
249. BD*(1) C3-N142	13.7
243. BD*(2) B1-B2	11.2
250. BD*(1) C3-N143	6.5
411. RY (1) B1	1.8
463. RY (1) C3	1.2

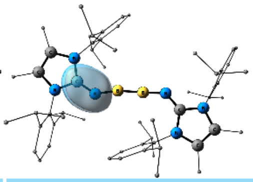

NBO 79. BD(2) C3-N141 occ. 0.96
34.13% C3 sp1.00
65.87% N141 sp1.00

Acceptor	E(2) (kcal mol^{-1})
244. BD*(3) B1-B2	7.2
248. BD*(2) C3-N141	1.9
412. RY (2) B1	1.3
2423. RY (1) N143	0.4
2397. RY (1) N142	0.4

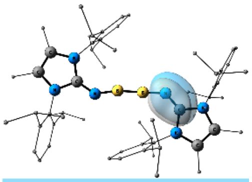

NBO 161. BD(2) C72-N144 occ. 0.96
33.69% C72 sp99.99
66.31% N144 sp1.00

Acceptor	E(2) (kcal mol^{-1})
244. BD*(3) B1-B2	7.7
330. BD*(2) C72-N144	2.2
438. RY (2) B2	1.3
2501. RY (1) N146	0.4
2475. RY (1) N145	0.4

β spin NBOs

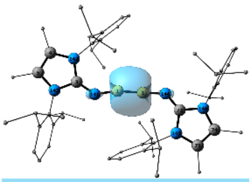

NBO 71. BD(1) B1-B2 occ. 0.99
49.68% B1 sp0.75
50.32% B2 sp0.76

Acceptor	E(2) (kcal mol^{-1})
2371. RY (1) N141	4.4
2449. RY (1) N144	3.6
2451. RY (3) N144	2.2
458. RY (22) B2	1.4
429. RY (19) B1	1.2

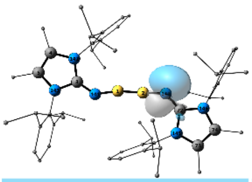

NBO 69. LP(1) N144 occ. 0.85
sp4.18

Acceptor	E(2) (kcal mol^{-1})
241. LV (1) B2	27.4
330. BD*(1) C72-N145	12.4
332. BD*(1) C72-N146	5.1
1417. RY (1) C72	1.4
242. BD*(1) B1-B2	0.7

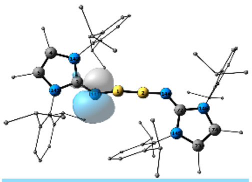

NBO 67. LP(1) N141 occ. 0.84
sp4.78

Acceptor	E(2) (kcal mol^{-1})
248. BD*(1) C3-N142	12.5
250. BD*(1) C3-N143	5.9
463. RY (1) C3	1.4
243. BD*(1) B1-N141	1.0
464. RY (2) C3	0.4

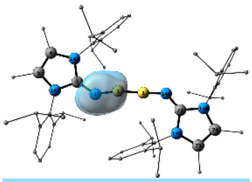

NBO 73. BD(2) B1-N141 occ. 0.84
13.45% B1 sp1.00
86.55% N141 sp1.00

Acceptor	E(2) (kcal mol^{-1})
249. BD*(2) C3-N142	55.8
246. BD*(2) B2-N144	1.9
465. RY (3) C3	1.2
467. RY (5) C3	0.3
439. RY (3) B2	0.3

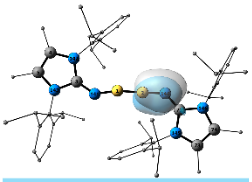

NBO 75. BD(2) B2-N144 occ. 0.84
13.97% B2 sp1.00
86.03% N144 sp1.00

Acceptor	E(2) (kcal mol^{-1})
331. BD*(2) C72-N145	56.1
244. BD*(2) B1-N141	2.0
1419. RY (3) C72	0.9
1421. RY (5) C72	0.7
413. RY (3) B1	0.3

Figure S68. Plots of selected natural bond orbitals (NBOs) of $\mathbf{1}_{INT}^{T}$ their atomic orbital compositions and five largest donor-acceptor interactions, according to the second-order perturbation theory.

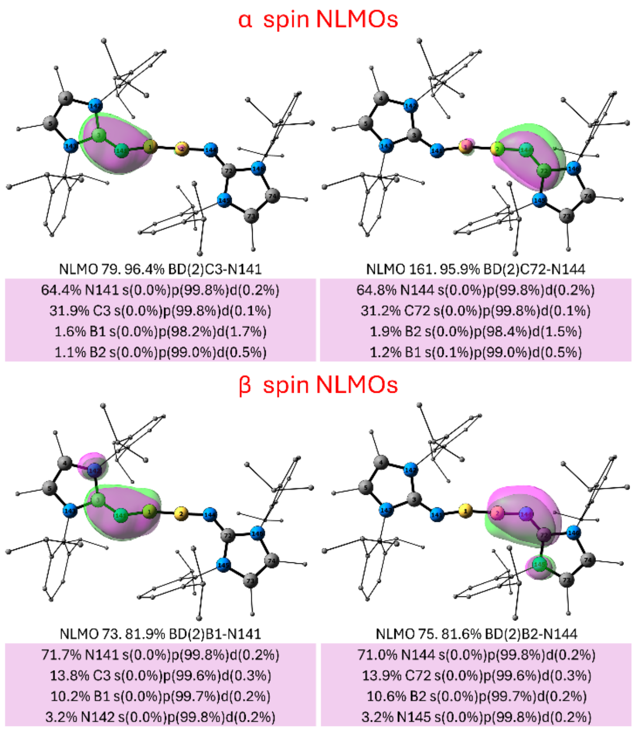

Figure S69. Plots of selected natural localized molecular orbitals (NLMOs) of $\mathbf{1_{INT}}^{T}$ their atomic orbital compositions.

The NBOs shown in **Figure S68** demonstrate that the α-spin NBO 79 and β-spin NBO 73, as well as the α-spin NBO 161 and β-spin NBO 75, essentially correspond to the same C−N−B delocalized π-bonding interaction.

Overall, a detailed discussion on the bis-NHI-diborene $\mathbf{1_{INT}}$ are as follows:

We carried out further computational analysis to elucidate the properties and electronic structure of bis-NHI-diborene $\mathbf{1_{INT}}$. As mentioned above the ground state of $\mathbf{1_{INT}}$ is triplet with a $\Delta E_{S\text{-}T}$ = $E_S - E_T$ = +1.2 kcal mol^{-1}. This is much smaller than the singlet-triplet gap for the ground-state triplet parent diborene HB=BH $\Delta E_{S\text{-}T}$ = +22.5 kcal mol^{-1} (+20.6[30] and +16.6 kcal mol^{-1}[30] in previous reports). This is in contrast to the base-stabilized diborenes and the heavier analogues ArE=EAr (E = Al, Ga, In, Tl)[31-34] all of which are ground state singlets. The optimized structure of $\mathbf{1_{INT}}^{S}$ (**Figure 4a**) exhibits a B−B bond length of 1.534 Å, which is at the lower end of the reported B−B

bonds in base-stabilized diborenes, and is similar to that of parent HB=BH (1.528 Å). The B−B bond lengths in base-stabilized diborenes (1.52-1.65 Å)[35-46] are generally longer, as the in-plane p orbitals are occupied by electrons donated by the Lewis bases, therefore each boron adopts sp^2 hybridization, which further diminishes the π-orbital overlap and results in bond elongation. The B−N bond lengths of 1.31 and 1.33 Å are in the range of B=N bonds (~1.39 Å).[47-48] The $N-C^{NHC}$ bonds are also short, 1.27 and 1.28 Å indicating double bond character.[47, 49-50] Overall, the geometry indicates a cumulene-like structure with the central −C=N=B=B=N=C− fragment having double bond character between all the neighboring atoms (**Figure 4b**). This is also reflected in the Wiberg bond indexes with WBI of 1.57 for B−B bond, 1.32 and 1.37 for the B−N bonds, and 1.45 and 1.46 for the N−C bonds. The N−B−B angles are only slightly bent 172.8° and 172.0°, consistent with the results through calculations in previous reports.[30, 51-54] The NBO calculation converges to the electronic structure corresponding to the resonance forms (ii) and (iii) (**Figure 4b**). These feature B=B, N=B and two C=N double bonds (**Figure 4c**), as well as a single bond in the second B−N moiety, with a lone pair on the nitrogen atom (**Figure 4c**). The lone pair has a low occupancy of 1.64 el. due to high delocalization to the lone vacancy p orbital of the adjacent boron atom (E(2) = 86.8 kcal mol^{-1}, according to the second order perturbation theory), which effectively results in a double bond interaction, similarly to the first N=B fragment (see more details in **Figure S66**). Taken together, these features are consistent with a cumulenic bonding pattern, allowing $\mathbf{1_{INT}^{S}}$ to be regarded as an analogue of a C_4 cumulene (resonance form (i)). Closer inspection of the individual NBOs further reveals significant polarization and delocalization effects that indicate a contribution from an additional resonance form. The π(B=N) orbital of the first B=N unit (NBO 75) is strongly polarized toward nitrogen, consistent with a description in which a nitrogen lone pair donates into an empty boron p orbital. Thus, the interpretation of $\mathbf{1_{INT}^{S}}$ in resonance form (iv) also becomes relevant. Moreover, the reduced occupancy of the π(B=B) orbital (NBO 73, 1.67 e) originates from donor-acceptor interactions with antibonding π*(N−C) orbitals with E(2) of 43.7 and 36.6 kcal mol^{-1}, which further enhances

the B−N multiple-bond character. Overall, the electronic structure of $\mathbf{1_{INT}}^{S}$ incorporates contributions from resonance forms (i)-(iv).

The considerably smaller singlet-triplet gap in $\mathbf{1_{INT}}$ compared to the parent system can be attributed to the enhanced stabilization of the singlet state in the NHI-substituted diborene. This arises from the above-mentioned ability of the π(B−B) orbital to be stabilized by delocalization into the adjacent π*(N−C) orbitals, as well as the stabilization of the empty p orbitals of the B by the lone pairs of the adjacent nitrogen atoms. These interactions lead to the N−B double bonding character, which results in cumulenic structure. Such stabilization mechanisms are not available in the parent system.

NBO analysis of the frontier canonical molecular orbitals (**Figure 4d**, see also **Figure S67**) reveals that the HOMO is predominately a bonding orbital with major contribution from the π(B=B) localized at the B=B moiety, whereas the LUMO corresponds mainly to an antibonding interaction involving the B=N fragments. As discussed above, the bonding π(B−N) interactions are strongly polarized toward the N centers, and hence, the lobe of the antibonding LUMO is predominately located on the B atoms. The perpendicular orientation of the electron-donor HOMO and the electron-acceptor LUMO with a very small energy gap of only 2.05 eV endows the boron centers in $\mathbf{1_{INT}}^{S}$ with pronounced amphiphilic character and an incredible ability to dearomatize a C_6 aromatic moiety with a barrier of merely 5.5 kcal mol^{-1}, which is significantly lower than that of most processes mediated by other main-group species.[55-67]

Moving on, the properties of the triplet state of the diborene intermediate were examined. The optimized geometry of $\mathbf{1_{INT}}^{T}$ (**Figure 5a**) exhibits a slightly shorter B=B bond of 1.514 Å in comparison to $\mathbf{1_{INT}}^{S}$, and it is nearly identical to the B−B distance in the parent triplet diborene HB=BH (1.513 Å; previously reported as 1.498[30] or 1.509[51] Å). Also, while the N=C^{NHC} are elongated by less than 0.015Å in comparison to the singlet, the B=N bonds are substantially longer (1.362 and 1.368 Å) than those in $\mathbf{1_{INT}}^{S}$. The bond length trend is consistent with Wiberg bond indexes (**Figure 5b left**). The $\mathbf{1_{INT}}^{T}$ skeleton is slightly bent away from a linear arrangement, in contrast to bis-bases-stabilized diborenes R(L)B=B(L)R and their heavier analogues ArE=EAr

(E = Al, Ga, In, Tl),[31-34] all of which exhibit consistently planar trans-bent geometries.[54] According to the NBO analysis, the electronic structure of the triplet structure can be described as featuring a delocalized π-conjugation within the C−N−B moieties (**Figures S68-S69**). In terms of the B−B bonding, in addition to the σ-bond represented by the corresponding α- and β-spin NBOs (**Figure 5c**), there are two additional mutually perpendicular α-spin bonding interactions. These can be interpreted as interaction between the unpaired electrons and the vacant p-orbitals of the adjacent boron center (**Figure 5b**). Consequently, the triplet state can be described as having a B−B linkage composed of one σ-bond and two orthogonal singly occupied π-type orbitals, corresponding to two one-electron π bonds.[40, 51-52] This situation is reflected in the nearly degenerate singly occupied molecular orbitals (SOMOs; **Figure 5d**), which correspond to the π-bonding interactions. In the triplet state, the two unpaired electrons occupy orbitals with energies of −3.02 and −2.99 eV, compared with the HOMO of the singlet state at −2.68 eV. The Mulliken atomic spin population analysis indicates that 0.77 and 0.76 of the spin density are localized on each of the boron atoms.

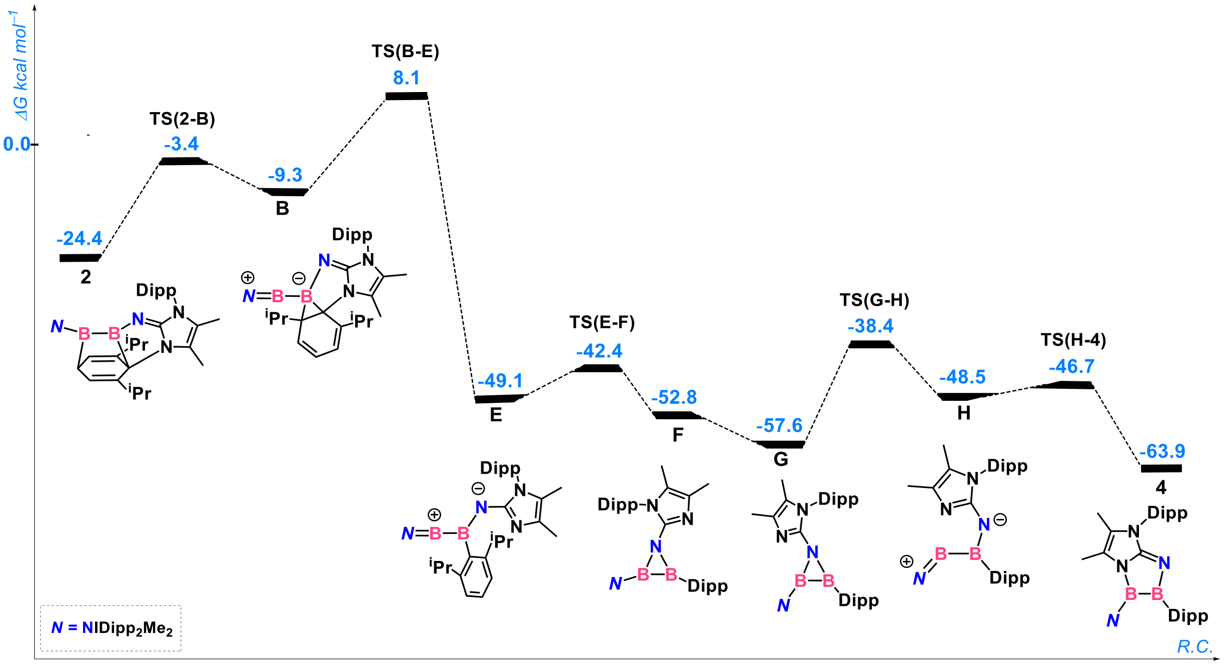

Figure S70. Calculated free energy reaction coordinate diagram of the proposed mechanism for the conversion of isomer **2** to isomer **4** in the absence of irradiation under ambient conditions at the (SMD=Benzene) PW6B95-D4/def2-QZVPP//r^{2}SCAN-3c level of theory.

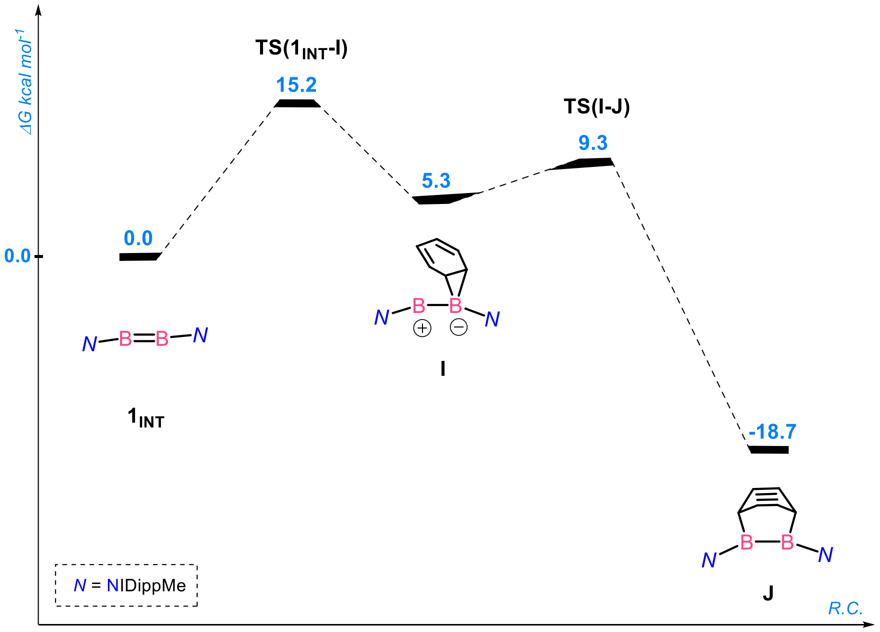

Figure S71. Free energy reaction coordinate diagram of the proposed mechanism for the dearomatization of benzene by **1$_{INT}$** at (SMD=Benzene) PW6B95-D4/def2-QZVPP//r^{2}SCAN-3c level of theory.

In response to a referee's request, who wondered whether performing the reduction of **1** in an aromatic solvent could lead to the formation of a solvent-masked species, we carried out additional calculations to address this point (Figure **S71**). The reaction of **1**$_{\mathbf{INT}}$ with benzene is indeed energetically possible. This process to form the hypothetical C6-deromatized product **J** is exergonic by 18.7 kcal mol^{-1} and proceeds in two steps. However, the rate-determining step for this process is 15.2 kcal mol^{-1}, that is 10.1 kcal mol^{-1} higher than the rate-determining transition state **TS(1**$_{\mathbf{INT}}$$^{\mathbf{S}}$**-A**$^{\mathbf{S}}$**)**$^{\mathbf{S}}$ in the formation of **2**. If we look at the difference of the enthalpies of these two transition states, we see that the reaction with benzene is actually preferred by 3.5 kcal mol^{-1}. When comparing intra- vs intermolecular reactions, entropy often plays the decisive role. In this case the entropy causes the intramolecular dearomatization to be kinetically much more favorable.

Cartesian coordinates and energies of the optimized geometries at the r^{2}SCAN-3c level.

Calculated energies and coordinates of **1**$_{\mathbf{INT}}$$^{\mathbf{S}}$

Electronic energy ... -2635.95217442 Eh
Total Enthalpy ... -2634.61577912 Eh
Final Gibbs free energy ... -2634.78313163 Eh

CARTESIAN COORDINATES (ANGSTROEM)

B	-0.020035	-0.650040	0.320833
B	-0.630899	0.744258	0.130434
C	0.702693	-3.080492	0.733599
C	0.975885	-4.994609	1.958986
C	1.459905	-5.240417	0.717529
C	0.878167	-5.856189	3.163058
H	1.409531	-5.410989	4.013472
H	1.313804	-6.837225	2.963893
H	-0.163348	-6.000639	3.476754
C	2.033097	-6.468815	0.113914
H	2.033143	-7.282678	0.841504
H	3.063598	-6.317041	-0.230926
H	1.449680	-6.787982	-0.758952
C	-0.151619	-3.009292	3.052936
C	-1.526830	-3.238189	3.230366
C	-2.168716	-2.568571	4.271243
H	-3.231808	-2.719309	4.433635
C	-1.469032	-1.691384	5.091270
H	-1.987459	-1.172068	5.892470
C	-0.115840	-1.465088	4.884127
H	0.414826	-0.765861	5.524599
C	0.571627	-2.118322	3.859350
C	-2.305835	-4.115477	2.268719
H	-1.593441	-4.772163	1.754586
C	-3.332751	-5.010253	2.971317
H	-4.156202	-4.426085	3.395873
H	-2.875725	-5.587972	3.781956
H	-3.770799	-5.711759	2.253386
C	-2.982068	-3.242299	1.197821
H	-3.488773	-3.872862	0.457432
H	-2.258324	-2.604905	0.678469
H	-3.730370	-2.586425	1.657727
C	2.048233	-1.852347	3.640633
H	2.379702	-2.466324	2.795073
C	2.300832	-0.383491	3.273233
H	1.710531	-0.088322	2.397326
H	3.361525	-0.229495	3.042540
H	2.038343	0.283424	4.102985
C	2.873868	-2.264755	4.867078
H	2.618987	-1.654157	5.740430
H	3.942869	-2.131260	4.667249
H	2.701635	-3.313952	5.130237
C	1.750600	-3.876721	-1.379826
C	0.821767	-3.878020	-2.429821
C	1.312399	-3.751248	-3.731448
H	0.617790	-3.757909	-4.567214
C	2.673337	-3.627993	-3.972694
H	3.037410	-3.545955	-4.993480
C	3.574190	-3.591960	-2.913772
H	4.633391	-3.471681	-3.118715
C	3.130795	-3.703912	-1.596563
C	-0.667691	-4.023356	-2.189088
H	-0.828914	-4.126016	-1.110502
C	-1.220290	-5.287105	-2.862769
H	-1.132320	-5.229203	-3.953435
H	-2.281434	-5.411545	-2.620164
H	-0.684278	-6.182289	-2.529543
C	-1.424900	-2.771179	-2.651866
H	-1.043645	-1.872278	-2.157392
H	-2.490386	-2.864311	-2.412095
H	-1.333667	-2.634610	-3.736230

C	4.083842	-3.555981	-0.423972
H	3.686212	-4.136973	0.417349
C	5.497303	-4.068281	-0.711653
H	5.484854	-5.090160	-1.105885
H	6.088474	-4.063867	0.209970
H	6.021751	-3.432388	-1.433208
C	4.116997	-2.084754	0.022204
H	4.533222	-1.456804	-0.774229
H	4.743669	-1.965357	0.913959
H	3.112067	-1.715599	0.249783
C	-0.905564	3.095243	-0.630302
C	-0.498112	4.540366	-2.357269
C	-1.141583	5.235818	-1.386730
C	0.017552	4.963953	-3.683649
H	-0.010337	6.052200	-3.771780
H	-0.569241	4.543083	-4.509735
H	1.052539	4.631222	-3.826977
C	-1.540343	6.662563	-1.302423
H	-2.629707	6.779779	-1.237618
H	-1.193174	7.205998	-2.183363
H	-1.114095	7.139521	-0.410973
C	0.344274	2.144234	-2.526522
C	-0.286544	1.496215	-3.602107
C	0.362008	0.405667	-4.179178
H	-0.106035	-0.124269	-5.004062
C	1.571130	-0.051910	-3.670604
H	2.046663	-0.926183	-4.103626
C	2.159471	0.577768	-2.581307
H	3.087882	0.181503	-2.184128
C	1.569714	1.694389	-1.987552
C	-1.697888	1.862269	-4.022690
H	-1.926958	2.860837	-3.635310
C	-2.685991	0.884786	-3.364702
H	-3.719944	1.174570	-3.585839
H	-2.550475	0.859350	-2.277606
H	-2.525328	-0.131797	-3.742205
C	-1.886250	1.895755	-5.542428
H	-1.801033	0.896816	-5.983645
H	-1.143103	2.539143	-6.025976
H	-2.883841	2.275011	-5.789414
C	2.239253	2.437579	-0.843597
H	1.465281	2.668754	-0.103031
C	2.838353	3.767237	-1.332530
H	3.590759	3.587740	-2.109581
H	3.326060	4.289650	-0.500953
H	2.071588	4.431859	-1.740004
C	3.304690	1.617422	-0.117595
H	2.896473	0.657550	0.211550
H	3.642024	2.164296	0.769376
H	4.185450	1.439076	-0.746484
C	-1.967953	4.691154	0.937411
C	-1.123078	4.929217	2.032389
C	-1.717645	5.240388	3.256568
H	-1.092031	5.426274	4.125181
C	-3.097904	5.322264	3.378135
H	-3.542375	5.571406	4.337816
C	-3.915248	5.083767	2.280056
H	-4.993312	5.143358	2.394572
C	-3.367839	4.754711	1.040186
C	0.386154	4.843771	1.913888
H	0.636403	4.747940	0.850833
C	1.073254	6.115732	2.428336
H	0.680364	7.009635	1.932441
H	2.150772	6.062879	2.238051
H	0.935239	6.240640	3.507749
C	0.921169	3.599290	2.639074
H	0.719477	3.663351	3.714968
H	2.006054	3.516104	2.503881
H	0.456062	2.685510	2.256665
C	-4.253153	4.391937	-0.137066
H	-3.672903	4.540388	-1.055695
C	-4.620710	2.900314	-0.065204

H	-3.724525	2.273255	-0.030327
H	-5.217533	2.610689	-0.937806
H	-5.211073	2.698469	0.836329
C	-5.510337	5.260995	-0.236963
H	-6.211427	5.056520	0.579285
H	-6.036510	5.049421	-1.173805
H	-5.265650	6.328436	-0.213641
N	0.380297	-1.897525	0.386332
N	0.517594	-3.667942	1.983390
N	1.293356	-4.069954	-0.046627
N	-1.007576	2.013403	0.048782
N	-0.325062	3.224838	-1.890560
N	-1.392080	4.355088	-0.320246

Calculated energies and coordinates of $\mathbf{1}_{INT}^{T}$

Electronic energy	...	-2635.95842788 Eh
Total Enthalpy	...	-2634.62228871 Eh
Final Gibbs free energy	...	-2634.79068712 Eh

CARTESIAN COORDINATES (ANGSTROEM)

B	0.242754	-0.596334	0.334280
B	-0.187744	0.851600	0.435585
C	0.728500	-3.015148	0.466737
C	0.466405	-4.899745	1.742809
C	1.145352	-5.261771	0.624957
C	0.030903	-5.681512	2.926630
H	0.289660	-6.735165	2.804131
H	-1.053078	-5.608239	3.074940
H	0.504776	-5.315873	3.846278
C	1.688936	-6.569300	0.180828
H	1.225029	-6.904741	-0.755360
H	1.512356	-7.332510	0.941210
H	2.768966	-6.507790	-0.000990
C	-0.459453	-2.722745	2.622843
C	-1.833649	-2.481644	2.480332
C	-2.447963	-1.656725	3.422756
H	-3.507812	-1.435180	3.331337
C	-1.719179	-1.096979	4.462238
H	-2.211702	-0.443616	5.176869
C	-0.362002	-1.363011	4.591599
H	0.195892	-0.913274	5.407474
C	0.295682	-2.183146	3.676430
C	-2.642468	-3.062875	1.336717
H	-1.967912	-3.660706	0.712187
C	-3.749032	-3.993595	1.852518
H	-4.475006	-3.444256	2.462026
H	-3.339297	-4.800753	2.469042
H	-4.289785	-4.443848	1.012583
C	-3.224408	-1.950237	0.454976
H	-3.723372	-2.378147	-0.422223
H	-2.433450	-1.272107	0.117852
H	-3.963279	-1.356518	1.005574
C	1.790196	-2.421173	3.781569
H	2.039435	-3.289350	3.158825
C	2.561880	-1.215159	3.222532
H	2.263584	-0.995717	2.192285
H	3.640879	-1.408654	3.246812
H	2.359498	-0.319910	3.821797
C	2.237594	-2.740450	5.212885
H	2.137008	-1.871875	5.872298
H	3.293408	-3.031831	5.218363
H	1.651923	-3.559480	5.644099
C	1.921028	-4.031138	-1.439157
C	1.159953	-4.362970	-2.571632
C	1.779623	-4.283859	-3.818763
H	1.220312	-4.534282	-4.715179
C	3.104537	-3.879025	-3.930475
H	3.571415	-3.822799	-4.910170
C	3.830805	-3.537598	-2.798108

H	4.861631	-3.209786	-2.901942
C	3.253654	-3.606013	-1.528285
C	-0.311302	-4.711505	-2.450042
H	-0.493791	-5.060904	-1.426283
C	-0.748918	-5.829034	-3.401849
H	-0.724076	-5.506197	-4.448153
H	-1.779794	-6.124556	-3.179895
H	-0.110143	-6.713571	-3.305564
C	-1.158847	-3.444799	-2.661362
H	-0.847150	-2.643265	-1.983871
H	-2.220357	-3.660962	-2.492605
H	-1.043208	-3.082597	-3.690219
C	4.045512	-3.186580	-0.305607
H	3.446074	-3.417455	0.582707
C	5.369764	-3.951145	-0.186728
H	5.208018	-5.034360	-0.186084
H	5.879548	-3.679363	0.743985
H	6.046380	-3.713436	-1.014778
C	4.276735	-1.668449	-0.322002
H	4.903695	-1.382235	-1.175598
H	4.785864	-1.348311	0.594169
H	3.322885	-1.137789	-0.395569
C	-0.690203	3.096032	-0.316208
C	-0.527215	4.405987	-2.188993
C	-1.165850	5.136139	-1.240316
C	-0.140501	4.732208	-3.584622
H	-0.393339	5.768717	-3.816738
H	-0.648175	4.084491	-4.310335
H	0.937048	4.599214	-3.737612
C	-1.741261	6.504092	-1.274072
H	-2.811995	6.488244	-1.036385
H	-1.618858	6.942391	-2.266540
H	-1.262537	7.169215	-0.544677
C	0.442129	2.075023	-2.272237
C	-0.297136	1.207109	-3.092049
C	0.363563	0.104261	-3.630185
H	-0.184812	-0.606490	-4.240846
C	1.710251	-0.117330	-3.371509
H	2.199027	-1.003800	-3.767276
C	2.429452	0.781154	-2.597285
H	3.483475	0.598645	-2.407805
C	1.811520	1.894786	-2.027724
C	-1.784935	1.407147	-3.315221
H	-2.023980	2.456415	-3.101482
C	-2.591114	0.545061	-2.332514
H	-3.666947	0.711525	-2.467031
H	-2.323875	0.772401	-1.296003
H	-2.381221	-0.518268	-2.498104
C	-2.209649	1.124118	-4.760254
H	-2.131080	0.058891	-5.003392
H	-1.595117	1.679354	-5.477220
H	-3.256051	1.413454	-4.905576
C	2.612106	2.850851	-1.164469
H	1.929029	3.614872	-0.774461
C	3.685640	3.568662	-1.995494
H	4.417081	2.855489	-2.391894
H	4.224770	4.295129	-1.377053
H	3.244959	4.100369	-2.845485
C	3.241695	2.136170	0.038523
H	2.481446	1.592217	0.609287
H	3.728127	2.863422	0.698511
H	4.002034	1.415429	-0.283732
C	-1.890600	4.702091	1.132079
C	-1.188490	5.522788	2.030174
C	-1.835321	5.899480	3.206684
H	-1.325484	6.536987	3.922236
C	-3.124472	5.458317	3.482548
H	-3.612088	5.760762	4.405236
C	-3.786452	4.626089	2.591590
H	-4.788346	4.276837	2.825743
C	-3.183409	4.230562	1.395615
C	0.256922	5.901842	1.765653
H	0.410089	5.924255	0.679522
C	0.636754	7.280435	2.312674
H	-0.064338	8.054546	1.982206
H	1.638862	7.553880	1.966316
H	0.661373	7.290626	3.407699
C	1.185509	4.813429	2.332123
H	1.090256	4.766009	3.423248
H	2.230388	5.038846	2.088437
H	0.934406	3.826376	1.930895
C	-3.915692	3.303438	0.445084
H	-3.292540	3.166140	-0.445801
C	-4.119207	1.920798	1.082257
H	-3.161702	1.492350	1.393726
H	-4.585143	1.239646	0.360884
H	-4.776562	1.987025	1.957459
C	-5.252011	3.902575	-0.013749
H	-5.948979	4.014243	0.824037
H	-5.724314	3.246864	-0.753552
H	-5.111979	4.889060	-0.468299
N	0.717051	-1.824010	-0.016907
N	0.205370	-3.524149	1.648211
N	1.304739	-4.109139	-0.155863
N	-0.634657	2.139075	0.550833
N	-0.238268	3.152825	-1.628865
N	-1.256663	4.336866	-0.091782

Calculated energies and coordinates of **TS(1_{INT}^{S}-A)**

Electronic energy ... -2635.94693993 Eh
Total Enthalpy ... -2634.61223972 Eh
Final Gibbs free energy ... -2634.77774359 Eh

CARTESIAN COORDINATES (ANGSTROEM)

B	0.087650	-0.657074	0.005719
B	-0.211298	0.863414	-0.065209
C	0.811316	-3.035784	0.612923
C	0.880919	-4.902420	1.930100
C	1.490586	-5.213163	0.759577
C	0.627652	-5.707212	3.150387
H	1.065495	-5.231946	4.036661
H	1.063104	-6.702932	3.046218
H	-0.446113	-5.820359	3.345343
C	2.094696	-6.477781	0.271791
H	1.965823	-7.268766	1.013237
H	3.167927	-6.371562	0.069032
H	1.623493	-6.803668	-0.663655
C	-0.289289	-2.848597	2.825251
C	-1.680508	-3.036456	2.873197
C	-2.398965	-2.327803	3.835648
H	-3.476958	-2.444255	3.895690
C	-1.756331	-1.454567	4.704280
H	-2.333963	-0.903909	5.441339
C	-0.383906	-1.268676	4.623052
H	0.104235	-0.568631	5.295388
C	0.378786	-1.960760	3.680691
C	-2.394930	-3.902780	1.854173
H	-1.653965	-4.561205	1.384374
C	-3.475006	-4.794044	2.476684
H	-4.314588	-4.204302	2.859681
H	-3.077949	-5.390904	3.304902
H	-3.876720	-5.477847	1.721413
C	-2.986986	-3.013026	0.748350
H	-3.430286	-3.630013	-0.042193
H	-2.224453	-2.363892	0.303095
H	-3.771629	-2.365920	1.157373
C	1.872998	-1.723273	3.590529
H	2.271183	-2.362343	2.793833
C	2.171075	-0.266408	3.208587
H	1.635398	0.017691	2.296344
H	3.244976	-0.131231	3.035592

H	1.869972	0.422804	4.006164
C	2.582647	-2.109256	4.895377
H	2.249767	-1.480142	5.728244
H	3.665532	-1.979523	4.791188
H	2.385695	-3.152573	5.164401
C	1.968284	-3.956287	-1.376751
C	1.085626	-4.060393	-2.460397
C	1.625143	-3.972071	-3.746081
H	0.968714	-4.054444	-4.608212
C	2.984960	-3.775543	-3.937624
H	3.385215	-3.708169	-4.945507
C	3.838160	-3.653345	-2.846564
H	4.897729	-3.487907	-3.014027
C	3.347729	-3.739775	-1.544277
C	-0.405610	-4.262958	-2.275304
H	-0.610794	-4.325328	-1.200580
C	-0.872507	-5.579750	-2.911591
H	-0.739722	-5.566311	-3.999053
H	-1.936890	-5.740392	-2.708117
H	-0.314054	-6.434880	-2.515909
C	-1.196955	-3.069956	-2.829532
H	-0.887206	-2.133923	-2.354430
H	-2.268291	-3.211079	-2.647878
H	-1.048751	-2.968893	-3.911177
C	4.255237	-3.541708	-0.343342
H	3.820293	-4.086981	0.503479
C	5.672910	-4.079817	-0.557541
H	5.662980	-5.116910	-0.909548
H	6.229195	-4.041773	0.384729
H	6.230620	-3.479796	-1.284587
C	4.291093	-2.054416	0.045192
H	4.725776	-1.460150	-0.767239
H	4.904819	-1.905997	0.941408
H	3.286501	-1.668603	0.243314
C	-0.870498	3.098940	-0.450138
C	-0.703414	4.466993	-2.277694
C	-1.242212	5.192148	-1.264000
C	-0.316627	4.869235	-3.656376
H	-0.115079	5.942691	-3.699844
H	-1.091653	4.643444	-4.398829
H	0.588154	4.334655	-3.966719
C	-1.635611	6.621139	-1.193900
H	-2.712755	6.743469	-1.022446
H	-1.384655	7.126869	-2.128646
H	-1.121079	7.133305	-0.371261
C	0.125489	2.042591	-2.387537
C	-0.509442	1.417552	-3.482816
C	0.077444	0.283860	-4.022554
H	-0.397152	-0.226644	-4.855365
C	1.217652	-0.281055	-3.427078
H	1.633269	-1.203234	-3.822232
C	1.803088	0.305860	-2.327859
H	2.673510	-0.166511	-1.883711
C	1.289869	1.494088	-1.749865
C	-1.893443	1.846123	-3.936117
H	-2.105202	2.840928	-3.532733
C	-2.927947	0.889017	-3.319233
H	-3.947692	1.216281	-3.554561
H	-2.813475	0.843574	-2.230699
H	-2.791059	-0.125910	-3.710760
C	-2.040355	1.913267	-5.458656
H	-1.963714	0.920726	-5.915388
H	-1.268011	2.546751	-5.907820
H	-3.021771	2.320048	-5.727212
C	2.185055	2.381240	-0.888447
H	1.564831	2.873044	-0.129906
C	2.825285	3.482307	-1.752554
H	3.405577	3.039149	-2.570387
H	3.499593	4.100049	-1.147443
H	2.067289	4.140363	-2.186769
C	3.268965	1.602241	-0.147160
H	2.834431	0.749578	0.382964
H	3.758883	2.251599	0.586224
H	4.044395	1.237297	-0.831356
C	-1.848532	4.732220	1.139723
C	-0.946016	5.042007	2.167399
C	-1.472495	5.401730	3.409531
H	-0.799451	5.642808	4.227573
C	-2.844186	5.462525	3.610939
H	-3.236071	5.752120	4.582118
C	-3.720383	5.150464	2.578214
H	-4.790467	5.193995	2.755777
C	-3.241295	4.769762	1.325064
C	0.554029	4.965898	1.962735
H	0.740814	4.857582	0.887651
C	1.268664	6.242328	2.423978
H	0.842789	7.132557	1.949130
H	2.331696	6.189877	2.164909
H	1.201853	6.374296	3.509364
C	1.127864	3.726389	2.667128
H	0.984911	3.800937	3.751880
H	2.203231	3.638633	2.472105
H	0.635355	2.815166	2.314406
C	-4.184868	4.322289	0.223798
H	-3.687966	4.490720	-0.739556
C	-4.436283	2.809200	0.346278
H	-3.496157	2.249243	0.326548
H	-5.071481	2.459747	-0.476080
H	-4.943688	2.585185	1.292117
C	-5.506204	5.095420	0.203931
H	-6.132787	4.852626	1.069050
H	-6.078339	4.830160	-0.691310
H	-5.341867	6.178495	0.197654
N	0.610120	-1.845886	0.191851
N	0.463778	-3.564394	1.849053
N	1.456309	-4.066756	-0.052426
N	-0.852815	2.023634	0.257908
N	-0.463495	3.172034	-1.781230
N	-1.343793	4.358571	-0.137956

Calculated energies and coordinates of **TS(1_{INT}^{T}-A^{T})^{T}**

Electronic energy ... -2635.94374033 Eh
Total Enthalpy ... -2634.60985722 Eh
Final Gibbs free energy ... -2634.77666355 Eh

CARTESIAN COORDINATES (ANGSTROEM)

B	0.497700	-0.543739	0.274037
B	0.136257	0.951283	0.020181
C	0.895745	-2.990025	0.514201
C	0.486563	-4.854854	1.770391
C	1.277495	-5.230300	0.732849
C	-0.084472	-5.628249	2.900796
H	0.265716	-5.248393	3.868633
H	0.199420	-6.679595	2.823504
H	-1.179434	-5.568186	2.910794
C	1.849720	-6.546193	0.355858
H	1.574945	-7.305315	1.090672
H	2.944442	-6.500722	0.301456
H	1.493115	-6.876805	-0.627619
C	-0.482553	-2.639063	2.531345
C	-1.806025	-2.298655	2.213419
C	-2.472846	-1.419970	3.068320
H	-3.492444	-1.121240	2.840594
C	-1.844682	-0.907509	4.193714
H	-2.374854	-0.213103	4.839294
C	-0.536738	-1.267235	4.493865
H	-0.057185	-0.846988	5.372690
C	0.173912	-2.135899	3.666858
C	-2.511913	-2.850046	0.989825
H	-1.796603	-3.466701	0.433048
C	-3.687157	-3.750594	1.397609

H	-4.453237	-3.176814	1.930826
H	-3.360365	-4.563325	2.054782
H	-4.154684	-4.191846	0.510333
C	-2.981479	-1.725927	0.058430
H	-3.416622	-2.146878	-0.854713
H	-2.147865	-1.073032	-0.221575
H	-3.747563	-1.107783	0.539788
C	1.631991	-2.453481	3.941964
H	1.895782	-3.361194	3.385069
C	2.525174	-1.317410	3.417384
H	2.354726	-1.137321	2.351055
H	3.583024	-1.561319	3.570338
H	2.306777	-0.383505	3.947752
C	1.911071	-2.726053	5.423990
H	1.795644	-1.821651	6.030366
H	2.941975	-3.072693	5.552604
H	1.237874	-3.490349	5.827048
C	2.188099	-4.034396	-1.302420
C	1.443489	-4.336862	-2.453152
C	2.087698	-4.240939	-3.686618
H	1.539152	-4.461398	-4.597908
C	3.417328	-3.846754	-3.766617
H	3.899628	-3.766959	-4.736931
C	4.130709	-3.547203	-2.614114
H	5.167513	-3.232516	-2.692780
C	3.531676	-3.638943	-1.356463
C	-0.029999	-4.688214	-2.377419
H	-0.268697	-4.931073	-1.334978
C	-0.387981	-5.912142	-3.228434
H	-0.284918	-5.704049	-4.298674
H	-1.430072	-6.199318	-3.051717
H	0.249890	-6.769821	-2.989655
C	-0.881045	-3.472776	-2.778116
H	-0.632728	-2.593111	-2.176301
H	-1.947137	-3.697361	-2.655783
H	-0.706320	-3.215143	-3.829393
C	4.309795	-3.276854	-0.106721
H	3.704515	-3.563480	0.761865
C	5.641376	-4.032222	-0.017490
H	5.493537	-5.114836	-0.092373
H	6.133093	-3.817230	0.937406
H	6.328929	-3.731897	-0.815470
C	4.525608	-1.758141	-0.038203
H	5.144349	-1.415691	-0.876440
H	5.032644	-1.480991	0.892990
H	3.567640	-1.232529	-0.080836
C	-0.786708	3.075283	-0.363648
C	-0.897463	4.392679	-2.224906
C	-1.371324	5.119386	-1.178505
C	-0.707323	4.761846	-3.653633
H	-0.518296	5.834048	-3.750431
H	-1.576857	4.515266	-4.275659
H	0.147127	4.220053	-4.072242
C	-1.895241	6.505728	-1.110914
H	-2.932219	6.525478	-0.753007
H	-1.870173	6.963901	-2.102058
H	-1.308013	7.130844	-0.426396
C	0.009268	1.993812	-2.376799
C	-0.746556	1.262194	-3.291431
C	-0.194161	0.085673	-3.815104
H	-0.750683	-0.497082	-4.541772
C	1.073558	-0.350405	-3.398238
H	1.499509	-1.255253	-3.825714
C	1.777762	0.335763	-2.440116
H	2.743773	-0.043891	-2.121526
C	1.231167	1.489309	-1.789450
C	-2.189154	1.628229	-3.590206
H	-2.344340	2.681097	-3.336890
C	-3.108114	0.812149	-2.666031
H	-4.159189	1.085001	-2.820688
H	-2.851164	0.984697	-1.614744
H	-2.994057	-0.259960	-2.863155
C	-2.575557	1.444424	-5.060165
H	-2.572251	0.388524	-5.350529
H	-1.884578	1.976614	-5.722815
H	-3.587338	1.826606	-5.236081
C	2.220530	2.496244	-1.180452
H	1.661005	3.195670	-0.548694
C	2.880952	3.312791	-2.302381
H	3.448238	2.654873	-2.970905
H	3.567115	4.060169	-1.886246
H	2.131474	3.835548	-2.905323
C	3.289747	1.853881	-0.296768
H	2.835718	1.227711	0.478983
H	3.884802	2.632784	0.192731
H	3.981380	1.235714	-0.880155
C	-1.708358	4.674896	1.281951
C	-0.810156	5.388885	2.089540
C	-1.229869	5.745158	3.371281
H	-0.559154	6.297117	4.022924
C	-2.492086	5.390056	3.830289
H	-2.801466	5.673386	4.832601
C	-3.356608	4.668976	3.018504
H	-4.336206	4.388361	3.395300
C	-2.983810	4.298048	1.725320
C	0.600034	5.686232	1.618641
H	0.612187	5.626317	0.523308
C	1.083310	7.086563	2.009314
H	0.375348	7.860086	1.692671
H	2.050104	7.292281	1.537764
H	1.224792	7.181666	3.091327
C	1.555541	4.604722	2.150662
H	1.600262	4.644123	3.245537
H	2.567873	4.762827	1.760647
H	1.217920	3.604233	1.861359
C	-3.921571	3.478164	0.861139
H	-3.512608	3.456081	-0.156029
C	-3.981456	2.028201	1.367909
H	-2.981847	1.582214	1.386393
H	-4.624028	1.426907	0.713820
H	-4.399069	1.992905	2.381672
C	-5.323913	4.094158	0.781315
H	-5.834675	4.062348	1.749813
H	-5.938945	3.534924	0.067958
H	-5.283823	5.138984	0.455615
N	0.907145	-1.793234	0.045868
N	0.245512	-3.478893	1.637696
N	1.534601	-4.085730	-0.035091
N	-0.552018	2.079766	0.437681
N	-0.559798	3.125661	-1.726330
N	-1.303651	4.307744	-0.035338

Calculated energies and coordinates of **A**

Electronic energy	...	-2635.96506977 Eh
Total Enthalpy	...	-2634.62963509 Eh
Final Gibbs free energy	...	-2634.79582117 Eh

CARTESIAN COORDINATES (ANGSTROEM)

B	0.297161	-0.633227	-0.168841
B	0.123959	0.915722	-0.603123
C	0.777663	-2.989022	0.758064
C	0.720474	-4.807280	2.116747
C	1.433456	-5.148210	1.010697
C	0.372544	-5.574957	3.337990
H	0.728761	-5.064454	4.240466
H	0.827405	-6.566604	3.303298
H	-0.712085	-5.698032	3.444366
C	2.099224	-6.415322	0.620250
H	1.890968	-7.194720	1.355575
H	3.187179	-6.296262	0.545847
H	1.746546	-6.761945	-0.358179

C	-0.445452	-2.695088	2.883226
C	-1.840383	-2.852322	2.909288
C	-2.559321	-2.075468	3.816666
H	-3.640040	-2.164513	3.863085
C	-1.912267	-1.170156	4.648674
H	-2.491031	-0.564688	5.340151
C	-0.535023	-1.021411	4.588228
H	-0.044376	-0.295828	5.230568
C	0.230915	-1.781084	3.702137
C	-2.549660	-3.760142	1.921762
H	-1.837893	-4.524003	1.584377
C	-3.751245	-4.490758	2.529356
H	-4.573166	-3.802784	2.753188
H	-3.482736	-5.010156	3.455562
H	-4.135052	-5.230357	1.819009
C	-2.972406	-2.947502	0.686007
H	-3.442381	-3.600284	-0.058735
H	-2.117674	-2.446304	0.221176
H	-3.694582	-2.173416	0.968984
C	1.732791	-1.581982	3.638101
H	2.140130	-2.257110	2.876536
C	2.073982	-0.146336	3.213800
H	1.565932	0.119266	2.281756
H	3.153879	-0.040207	3.061438
H	1.769900	0.575747	3.979522
C	2.401314	-1.938259	4.973125
H	2.061674	-1.274448	5.775520
H	3.488801	-1.834355	4.891537
H	2.175702	-2.967594	5.272038
C	2.069788	-3.929046	-1.116976
C	1.249705	-4.034504	-2.248749
C	1.862535	-3.921598	-3.498067
H	1.257786	-3.996348	-4.397313
C	3.227928	-3.702346	-3.606425
H	3.683820	-3.609738	-4.588067
C	4.014886	-3.584478	-2.467011
H	5.078526	-3.393520	-2.570769
C	3.450951	-3.689917	-1.196390
C	-0.247214	-4.258549	-2.155397
H	-0.531217	-4.253041	-1.096704
C	-0.634630	-5.630407	-2.726549
H	-0.398178	-5.694925	-3.794437
H	-1.711182	-5.797438	-2.611245
H	-0.104560	-6.442087	-2.216447
C	-1.024912	-3.130667	-2.847166
H	-0.726496	-2.148461	-2.466597
H	-2.099969	-3.259801	-2.680456
H	-0.853251	-3.135482	-3.929275
C	4.282821	-3.457004	0.051083
H	3.758448	-3.909199	0.901866
C	5.673442	-4.094436	-0.025085
H	5.616539	-5.155486	-0.290661
H	6.176880	-4.008112	0.943468
H	6.308178	-3.595014	-0.764408
C	4.383759	-1.947633	0.327179
H	4.917401	-1.445178	-0.487326
H	4.929818	-1.762171	1.259386
H	3.392927	-1.489417	0.406668
C	-0.921818	2.955956	-0.554054
C	-0.933006	4.551434	-2.190970
C	-1.405069	5.120337	-1.048633
C	-0.573105	5.193895	-3.484505
H	-0.234645	6.220333	-3.315825
H	-1.398409	5.227143	-4.204895
H	0.243669	4.636182	-3.955487
C	-1.817263	6.516347	-0.760531
H	-2.875708	6.576382	-0.475641
H	-1.670747	7.141854	-1.643821
H	-1.238217	6.941377	0.069029
C	-0.083585	2.166520	-2.666682
C	-0.604768	1.779020	-3.897277
C	-0.028606	0.687923	-4.562319
H	-0.424715	0.370608	-5.521329
C	0.924346	-0.115718	-3.906278
H	1.266104	-1.031028	-4.387597
C	1.360344	0.168428	-2.645003
H	2.041248	-0.523248	-2.150584
C	0.991300	1.419122	-1.911027
C	-1.887876	2.370856	-4.458834
H	-2.170574	3.240508	-3.859814
C	-3.031666	1.354228	-4.305149
H	-3.983337	1.792209	-4.630137
H	-3.129751	1.042654	-3.259891
H	-2.842127	0.458676	-4.906681
C	-1.749366	2.820289	-5.917491
H	-1.578459	1.965817	-6.580640
H	-0.909468	3.510568	-6.045961
H	-2.664950	3.320355	-6.254854
C	2.206729	2.334365	-1.560823
H	1.802013	3.200664	-1.014067
C	2.898998	2.841353	-2.826907
H	3.323253	1.999547	-3.387739
H	3.709466	3.540733	-2.589560
H	2.187012	3.348442	-3.487686
C	3.202429	1.630553	-0.642733
H	2.693331	1.193911	0.225414
H	3.964035	2.330368	-0.279479
H	3.721137	0.822802	-1.173200
C	-1.772977	4.347040	1.313827
C	-0.776800	4.688979	2.239417
C	-1.159821	4.877593	3.568663
H	-0.410505	5.137599	4.311113
C	-2.486452	4.739975	3.953252
H	-2.767407	4.895103	4.991513
C	-3.457722	4.405827	3.017604
H	-4.490835	4.299473	3.334459
C	-3.120220	4.200753	1.679662
C	0.678120	4.807904	1.831752
H	0.718447	4.851268	0.736628
C	1.336612	6.083680	2.369814
H	0.762750	6.976564	2.099672
H	2.346005	6.186033	1.957030
H	1.431044	6.061490	3.460936
C	1.452473	3.556650	2.274046
H	1.455282	3.475393	3.367975
H	2.492504	3.607931	1.931419
H	0.992011	2.654732	1.859614
C	-4.158329	3.759784	0.666118
H	-3.788140	4.028872	-0.330817
C	-4.300455	2.228372	0.706367
H	-3.333296	1.740985	0.546952
H	-5.001393	1.890358	-0.065896
H	-4.687799	1.912569	1.682854
C	-5.516261	4.442138	0.857169
H	-6.013990	4.106274	1.773391
H	-6.177841	4.192464	0.020946
H	-5.418024	5.532126	0.904207
N	0.607318	-1.810106	0.275508
N	0.310383	-3.476957	1.954843
N	1.470745	-4.022737	0.177460
N	-0.770698	1.816693	0.047911
N	-0.658865	3.201102	-1.900671
N	-1.413200	4.142670	-0.050118

Calculated energies and coordinates of **A**^{T}

Electronic energy ... -2635.95242208 Eh
Total Enthalpy ... -2634.61784758 Eh
Final Gibbs free energy ... -2634.78512864 Eh

CARTESIAN COORDINATES (ANGSTROEM)

B	0.396809	-0.563030	0.007984

B	0.234280	0.950019	-0.506534
C	0.732997	-3.003183	0.771667
C	0.698285	-4.900179	2.036252
C	1.392337	-5.181970	0.904166
C	0.372643	-5.731656	3.221384
H	0.730443	-5.259692	4.144426
H	0.841044	-6.714035	3.135624
H	-0.709059	-5.876513	3.334245
C	2.046818	-6.429548	0.438295
H	1.878275	-7.235829	1.154804
H	3.129630	-6.300472	0.316260
H	1.650486	-6.746335	-0.534017
C	-0.458839	-2.837053	2.934649
C	-1.851890	-3.015366	2.982255
C	-2.563529	-2.303874	3.947013
H	-3.641102	-2.416526	4.014167
C	-1.912939	-1.433326	4.813327
H	-2.485547	-0.880081	5.552313
C	-0.540379	-1.254395	4.728374
H	-0.047272	-0.556987	5.399670
C	0.217675	-1.952822	3.786417
C	-2.565128	-3.872912	1.953522
H	-1.858712	-4.627592	1.585911
C	-3.778582	-4.617208	2.518248
H	-4.595500	-3.931715	2.767458
H	-3.522594	-5.182467	3.420881
H	-4.164397	-5.318891	1.771425
C	-2.970681	-3.002483	0.751362
H	-3.438662	-3.618309	-0.025673
H	-2.107379	-2.488837	0.316980
H	-3.690334	-2.236561	1.062539
C	1.716250	-1.734005	3.707648
H	2.112060	-2.357135	2.897492
C	2.044383	-0.272507	3.369531
H	1.533110	0.043083	2.454202
H	3.123403	-0.151840	3.220452
H	1.740239	0.401228	4.178589
C	2.409510	-2.169693	5.006220
H	2.076789	-1.562628	5.855330
H	3.494903	-2.051487	4.915158
H	2.196400	-3.218334	5.240013
C	2.013491	-3.862658	-1.162396
C	1.196279	-3.935456	-2.299281
C	1.808946	-3.794570	-3.545742
H	1.204183	-3.848012	-4.446900
C	3.175129	-3.577151	-3.649194
H	3.633050	-3.468168	-4.628504
C	3.960656	-3.483678	-2.506327
H	5.025311	-3.295178	-2.605385
C	3.395167	-3.619362	-1.238973
C	-0.303007	-4.144069	-2.209845
H	-0.574558	-4.230557	-1.151422
C	-0.727375	-5.444952	-2.905236
H	-0.520890	-5.405503	-3.980465
H	-1.803217	-5.607932	-2.778125
H	-0.197346	-6.310367	-2.493135
C	-1.059292	-2.937488	-2.783978
H	-0.759341	-2.007358	-2.291225
H	-2.138686	-3.070400	-2.649622
H	-0.865267	-2.823246	-3.856580
C	4.224035	-3.419668	0.016223
H	3.711399	-3.921872	0.845876
C	5.631089	-4.015404	-0.086238
H	5.602878	-5.065029	-0.397928
H	6.130993	-3.958537	0.886322
H	6.253456	-3.466937	-0.801256
C	4.286750	-1.921268	0.357683
H	4.804384	-1.371645	-0.437067
H	4.834212	-1.762818	1.294167
H	3.284933	-1.492084	0.463017
C	-0.796605	3.022238	-0.560207
C	-0.886678	4.524961	-2.287634
C	-1.339220	5.151556	-1.170601
C	-0.629466	5.072346	-3.648025
H	-0.303924	6.114345	-3.582808
H	-1.509863	5.036484	-4.300712
H	0.161321	4.498316	-4.141103
C	-1.786582	6.548857	-0.952541
H	-2.842957	6.598894	-0.657897
H	-1.666049	7.128130	-1.870776
H	-1.209631	7.034150	-0.155487
C	-0.025073	2.121633	-2.656489
C	-0.617151	1.607642	-3.787282
C	-0.016546	0.478889	-4.404906
H	-0.442386	0.079530	-5.319714
C	1.100242	-0.151661	-3.824699
H	1.531654	-1.021990	-4.313418
C	1.628035	0.281957	-2.642771
H	2.457910	-0.260820	-2.195574
C	1.065450	1.432523	-1.892206
C	-1.958597	2.093367	-4.303844
H	-2.241823	2.991319	-3.746623
C	-3.029804	1.031812	-4.001452
H	-4.022098	1.387918	-4.302614
H	-3.050086	0.800413	-2.931134
H	-2.822145	0.100739	-4.540424
C	-1.937278	2.441003	-5.796497
H	-1.766845	1.550132	-6.410454
H	-1.145454	3.160399	-6.028978
H	-2.897092	2.871853	-6.103990
C	2.193802	2.433559	-1.441441
H	1.706268	3.228414	-0.861013
C	2.870282	3.075221	-2.655546
H	3.373076	2.312682	-3.262113
H	3.617151	3.814368	-2.342543
H	2.139613	3.577869	-3.298262
C	3.236368	1.785446	-0.530089
H	2.766651	1.281910	0.321585
H	3.924669	2.546519	-0.145145
H	3.838065	1.047452	-1.072863
C	-1.698975	4.479845	1.229514
C	-0.726405	4.826709	2.176508
C	-1.151215	5.078433	3.482819
H	-0.420263	5.343760	4.241645
C	-2.493285	4.994262	3.823790
H	-2.806230	5.198804	4.844103
C	-3.440178	4.644370	2.868300
H	-4.485270	4.574108	3.153799
C	-3.061421	4.374448	1.553767
C	0.746279	4.891666	1.825134
H	0.834994	4.819684	0.735098
C	1.387496	6.217579	2.253139
H	0.845782	7.075686	1.841248
H	2.423626	6.264028	1.900648
H	1.406474	6.324329	3.343190
C	1.490736	3.692566	2.432441
H	1.456024	3.731835	3.527902
H	2.542853	3.701823	2.125039
H	1.036094	2.754141	2.101064
C	-4.068168	3.897583	0.524280
H	-3.682252	4.155605	-0.469840
C	-4.181601	2.364278	0.589671
H	-3.202845	1.888116	0.472038
H	-4.852430	1.997778	-0.196249
H	-4.591687	2.059828	1.560116
C	-5.443907	4.553797	0.670169
H	-5.959112	4.219738	1.577295
H	-6.078092	4.279228	-0.179330
H	-5.369579	5.646158	0.704405
N	0.556454	-1.803228	0.359654
N	0.285652	-3.560998	1.957041
N	1.419975	-4.015437	0.124674
N	-0.568327	1.947920	0.131068
N	-0.590986	3.199943	-1.925534

N	-1.302058	4.224243	-0.116487

Calculated energies and coordinates of **TS[A-B]**

Electronic energy ... -2635.96238780 Eh
Total Enthalpy ... -2634.62815360 Eh
Final Gibbs free energy ... -2634.79159936 Eh

CARTESIAN COORDINATES (ANGSTROEM)

B	0.727075	-0.534233	-0.517911
B	0.497102	1.048692	-0.627830
C	0.955970	-2.874162	0.325737
C	0.457770	-4.365359	1.959318
C	1.100774	-5.042850	0.971066
C	-0.103720	-4.814727	3.256940
H	0.349263	-4.271018	4.094025
H	0.078940	-5.881222	3.400753
H	-1.185035	-4.639903	3.306873
C	1.459021	-6.475822	0.828843
H	1.032021	-6.900964	-0.087095
H	1.081260	-7.045811	1.679633
H	2.544881	-6.619462	0.774794
C	-0.234321	-1.939838	2.265831
C	-1.617620	-1.744246	2.138703
C	-2.171389	-0.633758	2.773068
H	-3.234612	-0.440089	2.677975
C	-1.376488	0.254485	3.484054
H	-1.819771	1.142660	3.926755
C	-0.013478	0.029713	3.603494
H	0.598174	0.739067	4.153193
C	0.589561	-1.073198	2.998655
C	-2.477206	-2.647288	1.274661
H	-1.912051	-3.563700	1.062426
C	-3.778311	-3.063241	1.971582
H	-4.446825	-2.208236	2.116378
H	-3.584521	-3.507758	2.953659
H	-4.314348	-3.796771	1.359845
C	-2.771950	-1.959978	-0.067506
H	-3.320713	-2.634421	-0.735617
H	-1.850993	-1.642364	-0.567113
H	-3.381840	-1.063485	0.090155
C	2.085020	-1.288110	3.130017
H	2.357202	-2.190140	2.567582
C	2.868923	-0.113012	2.530255
H	2.555243	0.082547	1.499898
H	3.943278	-0.328559	2.536176
H	2.706825	0.805151	3.105381
C	2.481536	-1.521247	4.594768
H	2.266026	-0.637260	5.204575
H	3.554891	-1.727619	4.671134
H	1.936192	-2.366488	5.028125
C	2.093221	-4.375227	-1.261287
C	1.344371	-4.484022	-2.440256
C	2.042680	-4.736910	-3.623031
H	1.495487	-4.824690	-4.557314
C	3.422773	-4.874293	-3.622096
H	3.946420	-5.073770	-4.552745
C	4.142357	-4.744405	-2.440205
H	5.223862	-4.834814	-2.460958
C	3.492964	-4.480994	-1.235048
C	-0.163700	-4.326143	-2.469777
H	-0.513452	-4.142553	-1.447236
C	-0.847079	-5.608078	-2.966268
H	-0.579805	-5.822255	-4.006715
H	-1.935927	-5.498788	-2.917884
H	-0.561041	-6.474865	-2.360870
C	-0.574639	-3.116137	-3.321976
H	-0.090197	-2.198530	-2.975097
H	-1.659839	-2.973258	-3.271890
H	-0.306308	-3.265788	-4.374053
C	4.277382	-4.218791	0.037208
H	3.620174	-4.412898	0.893725
C	5.505262	-5.121975	0.188835
H	5.246599	-6.179294	0.067271
H	5.945199	-4.987950	1.182463
H	6.282461	-4.877421	-0.542755
C	4.679739	-2.734997	0.095682
H	5.360131	-2.494503	-0.729291
H	5.192716	-2.514425	1.038674
H	3.807805	-2.078765	0.010738
C	-0.796028	2.841387	-0.226081
C	-1.336318	4.232022	-1.963926
C	-1.770978	4.815319	-0.815523
C	-1.303395	4.775103	-3.352430
H	-1.033536	5.835849	-3.347401
H	-2.263115	4.679569	-3.875277
H	-0.555842	4.239235	-3.945978
C	-2.400030	6.139346	-0.586619
H	-3.426487	6.057908	-0.204400
H	-2.438235	6.701360	-1.523033
H	-1.832382	6.726106	0.147353
C	-0.019004	2.006244	-2.299135
C	-0.548775	1.366168	-3.470460
C	0.269626	0.551655	-4.203308
H	-0.107585	0.018159	-5.068350
C	1.618992	0.311038	-3.769448
H	2.236429	-0.375068	-4.345820
C	2.139371	0.898372	-2.667968
H	3.169187	0.681144	-2.390052
C	1.385503	1.824622	-1.795660
C	-2.038245	1.466174	-3.745519
H	-2.367928	2.492654	-3.556068
C	-2.783537	0.569922	-2.740805
H	-3.869237	0.674710	-2.856794
H	-2.511158	0.822602	-1.711883
H	-2.515824	-0.480097	-2.909115
C	-2.435619	1.117555	-5.178884
H	-2.262835	0.057828	-5.398158
H	-1.873772	1.711300	-5.907737
H	-3.503831	1.310093	-5.326950
C	2.158534	3.082578	-1.360011
H	1.485207	3.678457	-0.730240
C	2.553846	3.934414	-2.568919
H	3.242854	3.383163	-3.220379
H	3.045528	4.863992	-2.258202
H	1.673028	4.195379	-3.165182
C	3.373937	2.728911	-0.504055
H	3.079639	2.074425	0.323115
H	3.829159	3.633762	-0.085114
H	4.146524	2.218073	-1.091024
C	-1.864159	4.099002	1.612565
C	-0.929559	4.445367	2.599714
C	-1.370829	4.521398	3.922715
H	-0.665889	4.782634	4.707531
C	-2.698052	4.279531	4.248082
H	-3.025805	4.353406	5.281604
C	-3.610868	3.941498	3.255118
H	-4.644521	3.746844	3.525708
C	-3.210379	3.831720	1.923744
C	0.522240	4.717520	2.263901
H	0.613870	4.720820	1.172433
C	0.979365	6.089196	2.775841
H	0.326523	6.888735	2.409621
H	2.000057	6.295216	2.435326
H	0.981682	6.131771	3.870664
C	1.418409	3.591621	2.797896
H	1.373965	3.546303	3.893883
H	2.461470	3.764369	2.508131
H	1.092377	2.633392	2.381996
C	-4.168061	3.333100	0.857536
H	-3.801991	3.672027	-0.118364
C	-4.141777	1.796219	0.836173

H	-3.116520	1.425367	0.730431
H	-4.745059	1.410831	0.005452
H	-4.554324	1.402003	1.773449
C	-5.597481	3.854338	1.026185
H	-6.083635	3.432086	1.912550
H	-6.203024	3.568745	0.159424
H	-5.620906	4.945745	1.115729
N	1.063581	-1.787501	-0.369088
N	0.368593	-3.027837	1.556257
N	1.411989	-4.113138	-0.031400
N	-0.281418	1.816660	0.352173
N	-0.778465	2.989691	-1.611769
N	-1.436442	3.970455	0.263127

Calculated energies and coordinates of **B**

Electronic energy ... -2635.96420176 Eh
Total Enthalpy ... -2634.62880111 Eh
Final Gibbs free energy ... -2634.79391361 Eh

CARTESIAN COORDINATES (ANGSTROEM)

B	0.286100	-0.618838	-0.461759
B	0.164636	0.967556	-0.607131
C	0.670819	-3.001215	0.295837
C	0.452705	-4.653840	1.831561
C	1.224273	-5.133966	0.819426
C	-0.045623	-5.294240	3.074525
H	0.130773	-4.655988	3.946944
H	0.459038	-6.248498	3.238425
H	-1.125441	-5.482081	3.028296
C	1.874743	-6.450744	0.611101
H	1.532304	-6.918438	-0.319565
H	1.642406	-7.123902	1.438234
H	2.964758	-6.352223	0.541691
C	-0.749411	-2.455667	2.236367
C	-2.133579	-2.665435	2.142405
C	-2.965018	-1.810101	2.865037
H	-4.041965	-1.938365	2.817517
C	-2.432264	-0.773627	3.618842
H	-3.094310	-0.105536	4.161714
C	-1.062010	-0.557383	3.652672
H	-0.670703	0.291179	4.202931
C	-0.185587	-1.393871	2.961161
C	-2.716295	-3.699989	1.197997
H	-1.949470	-4.456975	0.992718
C	-3.936200	-4.426230	1.773387
H	-4.799254	-3.758619	1.864498
H	-3.727510	-4.842549	2.764748
H	-4.231063	-5.246321	1.110341
C	-3.060254	-3.027478	-0.142737
H	-3.425795	-3.769665	-0.861925
H	-2.187240	-2.525074	-0.571813
H	-3.840287	-2.270325	-0.002518
C	1.314097	-1.158174	3.003191
H	1.740040	-1.530036	2.064419
C	1.675169	0.327900	3.095648
H	1.137746	0.917459	2.343726
H	2.751961	0.451494	2.935538
H	1.448815	0.738168	4.086614
C	1.955629	-1.954072	4.150384
H	1.532355	-1.647764	5.113983
H	3.036014	-1.773235	4.179533
H	1.796779	-3.031584	4.035407
C	2.136188	-4.120609	-1.322104
C	1.503765	-4.403910	-2.539476
C	2.288635	-4.372678	-3.693497
H	1.831747	-4.579477	-4.656951
C	3.640263	-4.065696	-3.629062
H	4.232573	-4.042383	-4.539445
C	4.237561	-3.772673	-2.409810
H	5.291221	-3.512379	-2.377974
C	3.495684	-3.787354	-1.228933
C	0.016298	-4.682550	-2.635548
H	-0.386148	-4.754734	-1.617469
C	-0.272328	-6.013832	-3.341212
H	0.041362	-5.986163	-4.390008
H	-1.347241	-6.223448	-3.324490
H	0.248549	-6.846515	-2.856706
C	-0.698921	-3.517004	-3.336831
H	-0.489749	-2.565860	-2.838753
H	-1.782184	-3.681887	-3.340226
H	-0.367359	-3.428282	-4.377929
C	4.127828	-3.371969	0.086025
H	3.465425	-3.686343	0.902399
C	5.490416	-4.032902	0.323656
H	5.432074	-5.122189	0.226647
H	5.849204	-3.795614	1.330617
H	6.244437	-3.671453	-0.383179
C	4.243725	-1.839210	0.145946
H	4.912924	-1.475251	-0.642232
H	4.653941	-1.524200	1.112308
H	3.271736	-1.356067	0.004937
C	-0.638918	2.975371	-0.152469
C	-0.928567	4.290087	-2.005022
C	-1.189317	5.052136	-0.909470
C	-0.886332	4.663625	-3.448279
H	-0.459244	5.662564	-3.577426
H	-1.876786	4.661809	-3.920638
H	-0.261464	3.955209	-4.001449
C	-1.611738	6.469524	-0.795032
H	-2.598732	6.561724	-0.322888
H	-1.669583	6.921062	-1.788431
H	-0.912252	7.059842	-0.188299
C	-0.136848	1.767488	-2.071764
C	-0.793154	1.162356	-3.234356
C	-0.130700	0.206139	-3.929112
H	-0.616830	-0.318525	-4.745533
C	1.223400	-0.161178	-3.588576
H	1.716143	-0.937238	-4.169779
C	1.913349	0.477806	-2.611071
H	2.964198	0.232965	-2.464597
C	1.334405	1.493092	-1.747341
C	-2.259313	1.476954	-3.446287
H	-2.398990	2.563045	-3.389759
C	-3.072781	0.866080	-2.288700
H	-4.133619	1.127971	-2.383566
H	-2.709884	1.214792	-1.317379
H	-2.981283	-0.227044	-2.305104
C	-2.810259	1.013801	-4.793121
H	-2.810102	-0.079553	-4.871085
H	-2.223014	1.414057	-5.626572
H	-3.846502	1.349371	-4.909842
C	2.261426	2.615812	-1.291591
H	1.680102	3.302511	-0.665059
C	2.803894	3.414692	-2.483136
H	3.412123	2.777317	-3.136011
H	3.425955	4.252681	-2.145731
H	1.983458	3.819907	-3.084718
C	3.395262	2.077102	-0.415339
H	2.983270	1.518515	0.431968
H	4.008216	2.898145	-0.026695
H	4.060954	1.410194	-0.976632
C	-1.393573	4.596316	1.566490
C	-0.483957	5.267731	2.399132
C	-0.898695	5.597328	3.689712
H	-0.219915	6.118570	4.357793
C	-2.171252	5.260025	4.135338
H	-2.477583	5.524217	5.143991
C	-3.051021	4.585592	3.300085
H	-4.040421	4.322506	3.664502
C	-2.681745	4.244119	1.997455
C	0.931155	5.542490	1.929232

H	0.908430	5.626300	0.835530
C	1.517518	6.842156	2.486017
H	0.852243	7.694386	2.309146
H	2.478454	7.051778	2.004344
H	1.705414	6.775759	3.563401
C	1.824878	4.340009	2.278134
H	1.879740	4.214440	3.366582
H	2.841847	4.494783	1.898885
H	1.423580	3.416453	1.847562
C	-3.636038	3.475709	1.103489
H	-3.230764	3.485282	0.084753
C	-3.714926	2.009367	1.556064
H	-2.720039	1.551702	1.561948
H	-4.359016	1.434689	0.879575
H	-4.137998	1.944242	2.566240
C	-5.028810	4.114832	1.050526
H	-5.538916	4.056183	2.018365
H	-5.653025	3.590689	0.318556
H	-4.973489	5.169736	0.761511
N	0.592354	-1.874632	-0.327930
N	0.117474	-3.331188	1.505710
N	1.351600	-4.108428	-0.125215
N	-0.261962	1.913781	0.476882
N	-0.650814	2.997542	-1.539220
N	-0.996809	4.252640	0.243386

Calculated energies and coordinates of **TS[B-2]**

Electronic energy ... -2635.96079238 Eh
Total Enthalpy ... -2634.62626976 Eh
Final Gibbs free energy ... -2634.78784134 Eh

CARTESIAN COORDINATES (ANGSTROEM)

B	0.132924	-0.725085	-0.894470
B	-0.295996	0.825574	-0.754330
C	0.754054	-2.905587	0.080995
C	0.113972	-4.500803	1.578749
C	1.258995	-4.933052	0.992301
C	-0.775553	-5.134281	2.584453
H	-1.016705	-4.437768	3.394780
H	-0.295890	-6.015393	3.015915
H	-1.726322	-5.448358	2.136308
C	2.073760	-6.154394	1.208209
H	3.099066	-5.897045	1.500277
H	2.142699	-6.769617	0.302530
H	1.634400	-6.764062	1.999949
C	-1.287612	-2.408230	1.392525
C	-2.538198	-2.682116	0.821051
C	-3.608841	-1.862235	1.180897
H	-4.589611	-2.036687	0.748205
C	-3.427670	-0.812777	2.068091
H	-4.266841	-0.173781	2.329100
C	-2.173489	-0.549735	2.604563
H	-2.048634	0.298681	3.266327
C	-1.069594	-1.335403	2.278658
C	-2.722148	-3.768017	-0.221946
H	-1.850491	-4.433103	-0.188540
C	-3.967483	-4.626412	0.029695
H	-4.888227	-4.052622	-0.118580
H	-3.983898	-5.026403	1.049208
H	-3.992503	-5.467602	-0.671362
C	-2.764811	-3.137934	-1.622847
H	-2.824202	-3.915299	-2.393569
H	-1.878066	-2.523728	-1.810199
H	-3.641016	-2.486887	-1.724722
C	0.311982	-1.025822	2.839511
H	1.017371	-1.079283	1.997727
C	0.424549	0.383417	3.426053
H	0.100956	1.137902	2.702142
H	1.471012	0.582438	3.681804
H	-0.156918	0.480040	4.351139
C	0.750859	-2.054705	3.896292
H	0.034067	-2.078397	4.725717
H	1.727220	-1.769832	4.304688
H	0.844854	-3.065227	3.490580
C	2.851676	-3.953589	-0.700081
C	2.878637	-4.671366	-1.905937
C	4.061916	-4.651082	-2.643282
H	4.122300	-5.194370	-3.580883
C	5.166355	-3.935329	-2.196139
H	6.077509	-3.925238	-2.787782
C	5.113297	-3.235369	-0.999655
H	5.984953	-2.680970	-0.663722
C	3.955343	-3.235870	-0.218931
C	1.639424	-5.381534	-2.419100
H	1.032899	-5.672089	-1.552094
C	1.953404	-6.655750	-3.207540
H	2.422882	-6.431933	-4.171366
H	1.026008	-7.197422	-3.420250
H	2.620789	-7.322360	-2.650789
C	0.795745	-4.410829	-3.260971
H	0.528601	-3.517942	-2.688234
H	-0.125825	-4.897694	-3.600281
H	1.359518	-4.087906	-4.144229
C	3.912041	-2.464866	1.086617
H	2.984485	-2.728068	1.609137
C	5.076183	-2.840966	2.012420
H	5.117894	-3.921486	2.185888
H	4.959738	-2.341768	2.980285
H	6.039741	-2.529263	1.595518
C	3.881815	-0.951904	0.823287
H	4.802330	-0.625161	0.325304
H	3.793368	-0.400904	1.766895
H	3.034289	-0.685456	0.186417
C	-0.715068	2.872771	-0.093300
C	-1.434165	4.241886	-1.772500
C	-1.206420	5.011918	-0.673739
C	-1.804665	4.644727	-3.159423
H	-1.304878	5.578320	-3.434491
H	-2.883725	4.795357	-3.287436
H	-1.493949	3.875168	-3.872681
C	-1.320522	6.478350	-0.479995
H	-2.096900	6.741839	0.250498
H	-1.575942	6.959940	-1.426938
H	-0.379558	6.909339	-0.114831
C	-0.994535	1.633502	-2.016643
C	-2.058101	1.046366	-2.864125
C	-1.751324	-0.108350	-3.490864
H	-2.504466	-0.663164	-4.041204
C	-0.413974	-0.655248	-3.427800
H	-0.219053	-1.615329	-3.895989
C	0.657260	0.152171	-3.087596
H	1.670531	-0.169400	-3.322294
C	0.471859	1.356924	-2.370144
C	-3.461731	1.589049	-2.742797
H	-3.428732	2.682885	-2.814616
C	-4.005410	1.244969	-1.341924
H	-4.998184	1.686119	-1.190099
H	-3.339381	1.605642	-0.552989
H	-4.083894	0.157065	-1.231724
C	-4.417616	1.086715	-3.823695
H	-4.584789	0.007125	-3.736274
H	-4.030597	1.290458	-4.827582
H	-5.392322	1.576402	-3.722225
C	1.542189	2.427517	-2.336498
H	1.172708	3.250169	-1.711613
C	1.801544	2.995024	-3.740377
H	2.213660	2.220729	-4.397833
H	2.516584	3.825771	-3.701857
H	0.875574	3.359233	-4.196818
C	2.836739	1.918030	-1.697726
H	2.636350	1.558251	-0.684792

H	3.584462	2.718549	-1.647790
H	3.272002	1.094705	-2.276497
C	-0.550914	4.549657	1.729464
C	0.757272	4.704767	2.207164
C	0.923051	5.074755	3.544647
H	1.926003	5.199833	3.943749
C	-0.171451	5.289199	4.368292
H	-0.021304	5.583873	5.403325
C	-1.461147	5.120393	3.877341
H	-2.308506	5.276997	4.537591
C	-1.675390	4.737137	2.554463
C	1.972509	4.469939	1.332931
H	1.622248	4.257321	0.317274
C	2.870087	5.712883	1.268114
H	2.308561	6.593266	0.937800
H	3.693327	5.546877	0.564406
H	3.310527	5.942605	2.244754
C	2.756397	3.241612	1.816178
H	3.104856	3.378402	2.846862
H	3.634043	3.078759	1.180650
H	2.122164	2.351506	1.770157
C	-3.069832	4.430769	2.041238
H	-3.080705	4.596130	0.957102
C	-3.378904	2.942254	2.274428
H	-2.617945	2.302809	1.815219
H	-4.355014	2.681840	1.848228
H	-3.402250	2.725861	3.350025
C	-4.158191	5.315366	2.654656
H	-4.316332	5.090872	3.715213
H	-5.110278	5.140180	2.142925
H	-3.911624	6.378900	2.565944
N	0.808689	-1.851833	-0.663444
N	-0.181626	-3.241395	1.034882
N	1.646889	-3.952611	0.067216
N	-0.235750	1.765531	0.385519
N	-1.203140	2.913576	-1.388652
N	-0.767219	4.168585	0.373179

Calculated energies and coordinates of **2**

Electronic energy ... -2635.99318150 Eh
Total Enthalpy ... -2634.65656834 Eh
Final Gibbs free energy ... -2634.81990726 Eh

CARTESIAN COORDINATES (ANGSTROEM)

B	4.509919	2.234553	11.080926
B	4.392880	3.810566	11.688072
C	5.256294	0.322776	12.609568
C	5.102253	-1.076216	14.425162
C	6.345420	-1.219909	13.910665
C	4.402549	-1.838766	15.495393
H	3.538216	-1.278905	15.861327
H	5.065598	-2.034180	16.342876
H	4.032059	-2.803253	15.125003
C	7.463889	-2.114452	14.296170
H	7.701211	-2.834952	13.502897
H	7.203231	-2.674023	15.197457
H	8.379573	-1.543768	14.494717
C	3.209470	0.543693	13.984364
C	2.071224	0.254682	13.215539
C	0.890916	0.936758	13.511298
H	-0.003867	0.738909	12.927579
C	0.843541	1.859425	14.547694
H	-0.081764	2.387502	14.762227
C	1.973204	2.106148	15.315021
H	1.922869	2.825887	16.126897
C	3.182182	1.461163	15.051185
C	2.088674	-0.808517	12.133733
H	3.129077	-1.104853	11.964880
C	1.315662	-2.049817	12.603747
H	0.258436	-1.811955	12.768175
H	1.720780	-2.437045	13.544998
H	1.371514	-2.844412	11.851020
C	1.544597	-0.290524	10.799823
H	1.664703	-1.052176	10.021221
H	2.086170	0.607009	10.492787
H	0.477870	-0.046354	10.862358
C	4.390181	1.753592	15.927063
H	5.242772	1.187732	15.533870
C	4.775581	3.239291	15.916922
H	4.926417	3.618333	14.902492
H	5.694773	3.382398	16.497914
H	3.997278	3.855005	16.385169
C	4.144271	1.292181	17.373713
H	3.333104	1.870284	17.830564
H	5.045683	1.451668	17.975803
H	3.875187	0.233976	17.435686
C	7.568588	-0.236567	11.937257
C	7.650074	-1.100955	10.834536
C	8.728648	-0.944191	9.963709
H	8.816405	-1.590653	9.095132
C	9.688204	0.034266	10.188384
H	10.518769	0.145643	9.496697
C	9.593135	0.870088	11.292898
H	10.352262	1.630113	11.455522
C	8.532635	0.749938	12.192444
C	6.586600	-2.147938	10.562845
H	5.969158	-2.242603	11.464253
C	7.190102	-3.527055	10.267936
H	7.726180	-3.537697	9.312802
H	6.395373	-4.278068	10.203950
H	7.892266	-3.834727	11.050030
C	5.666815	-1.694925	9.418635
H	5.208766	-0.730287	9.655789
H	4.872698	-2.432193	9.252070
H	6.235080	-1.590184	8.486762
C	8.417740	1.684341	13.380588
H	7.641901	1.287686	14.046298
C	9.720688	1.764604	14.185665
H	10.067427	0.771426	14.490693
H	9.565279	2.367464	15.087002
H	10.522730	2.238687	13.609591
C	7.966266	3.079768	12.928164
H	8.724013	3.538833	12.281386
H	7.816671	3.730287	13.796854
H	7.022623	3.036938	12.376820
C	4.269353	5.834670	12.460598
C	3.644218	7.429019	10.975180
C	3.811331	8.027879	12.188509
C	3.267920	8.023467	9.659018
H	3.996886	8.774563	9.334217
H	2.285398	8.506372	9.697923
H	3.227897	7.245177	8.892139
C	3.646309	9.446299	12.592042
H	2.780688	9.592809	13.251107
H	3.499798	10.065701	11.703427
H	4.525743	9.819082	13.130661
C	3.936175	4.811794	10.436687
C	2.604310	4.256542	9.964742
C	2.703855	3.011118	9.473649
H	1.848166	2.474786	9.076704
C	4.058931	2.352517	9.495797
H	4.070124	1.399311	8.958265
C	5.073319	3.325149	8.949749
H	5.811709	2.987534	8.229588
C	5.037329	4.586644	9.414931
C	1.314710	5.013359	10.168431
H	1.484335	6.066784	9.903610
C	0.898083	4.973239	11.648101
H	-0.005826	5.572611	11.813457
H	1.685299	5.354777	12.301568
H	0.696116	3.939233	11.951557

C	0.172403	4.496433	9.292583
H	-0.103620	3.473481	9.572595
H	0.446351	4.498054	8.232870
H	-0.716283	5.123713	9.423051
C	6.044001	5.665683	9.096918
H	5.516722	6.629907	9.073030
C	6.716149	5.473710	7.736091
H	7.334946	4.569811	7.725526
H	7.372970	6.322724	7.516036
H	5.976284	5.388952	6.933961
C	7.109521	5.753137	10.202217
H	6.658098	5.892517	11.188223
H	7.796251	6.587386	10.015293
H	7.686133	4.822018	10.231660
C	4.437229	7.229769	14.510227
C	5.755967	7.241747	14.995934
C	5.937600	7.499023	16.355014
H	6.939410	7.517829	16.770022
C	4.852980	7.722677	17.194325
H	5.019939	7.926235	18.248413
C	3.559508	7.660477	16.698691
H	2.719781	7.798428	17.372840
C	3.325111	7.401430	15.347052
C	6.932210	6.972031	14.073407
H	6.639149	6.137330	13.423533
C	7.246068	8.190798	13.190370
H	6.416090	8.437100	12.521956
H	8.123987	7.988047	12.567351
H	7.463528	9.066734	13.812993
C	8.195443	6.530940	14.815683
H	8.638159	7.350871	15.393294
H	8.945493	6.201448	14.089511
H	7.993986	5.696879	15.495865
C	1.913632	7.181281	14.838963
H	1.906692	7.329333	13.752647
C	1.523264	5.717713	15.105736
H	2.244171	5.028970	14.653543
H	0.531056	5.499242	14.694444
H	1.500595	5.525982	16.185628
C	0.885901	8.142464	15.442999
H	0.719833	7.944811	16.507366
H	-0.077975	8.019463	14.938192
H	1.197742	9.187216	15.338112
N	4.965116	1.042994	11.594921
N	4.436575	-0.105871	13.650605
N	6.451940	-0.349881	12.815227
N	4.560706	4.606660	12.855109
N	3.933734	6.077744	11.153036
N	4.211888	7.030228	13.110691

Calculated energies and coordinates of **2**^{T}

Electronic energy ... -2635.93689855 Eh
Total Enthalpy ... -2634.60216930 Eh
Final Gibbs free energy ... -2634.76619441 Eh

CARTESIAN COORDINATES (ANGSTROEM)

B	4.751905	2.330821	11.406001
B	4.214216	3.579860	12.277963
C	5.594944	0.325464	12.667905
C	5.362042	-0.990989	14.545152
C	6.439402	-1.452086	13.863349
C	4.699159	-1.490022	15.777154
H	4.684939	-0.739938	16.577405
H	5.218507	-2.374931	16.150626
H	3.655352	-1.764175	15.581019
C	7.378428	-2.566551	14.145688
H	7.344654	-3.335735	13.363913
H	7.133391	-3.037721	15.099541
H	8.413919	-2.207933	14.196015
C	3.692769	0.847443	14.197412
C	2.478854	0.550722	13.563758
C	1.357068	1.296359	13.929232
H	0.401122	1.097645	13.452129
C	1.447311	2.286653	14.894443
H	0.564539	2.859865	15.162717
C	2.658486	2.551984	15.523976
H	2.706204	3.334556	16.274452
C	3.806616	1.835250	15.193043
C	2.355027	-0.548724	12.526786
H	3.348777	-0.980507	12.364997
C	1.434782	-1.673010	13.023333
H	0.412058	-1.309702	13.174043
H	1.786585	-2.085739	13.974648
H	1.397823	-2.485982	12.289473
C	1.871010	0.003324	11.181532
H	1.875935	-0.785623	10.421193
H	2.523457	0.815796	10.848894
H	0.849130	0.393275	11.255341
C	5.145657	2.117028	15.856808
H	5.679985	1.162885	15.947633
C	6.018635	3.036275	14.990050
H	6.151417	2.644686	13.976411
H	7.009787	3.150421	15.445795
H	5.552893	4.022697	14.901617
C	5.009417	2.707393	17.262690
H	4.629438	3.735177	17.229916
H	5.992204	2.741326	17.744761
H	4.339051	2.112486	17.892555
C	7.585409	-0.805523	11.721324
C	7.387169	-1.765910	10.715891
C	8.373404	-1.899732	9.739221
H	8.250902	-2.631512	8.946407
C	9.508718	-1.098968	9.760383
H	10.264614	-1.213110	8.988245
C	9.680980	-0.153437	10.761271
H	10.572302	0.468013	10.762447
C	8.725030	0.009375	11.765818
C	6.106783	-2.577527	10.650520
H	5.673749	-2.606438	11.657929
C	6.331492	-4.025817	10.204765
H	6.627292	-4.086887	9.151954
H	5.402573	-4.595826	10.311974
H	7.106978	-4.516606	10.802794
C	5.090116	-1.873065	9.736450
H	4.909010	-0.847353	10.073795
H	4.139221	-2.419267	9.729901
H	5.468577	-1.832195	8.708095
C	8.918387	1.057217	12.844308
H	8.138905	0.911404	13.601747
C	10.275246	0.916116	13.545820
H	10.419223	-0.096434	13.937720
H	10.341416	1.620846	14.382060
H	11.105910	1.134599	12.865983
C	8.739860	2.466361	12.261800
H	9.505527	2.673040	11.504258
H	8.832049	3.222140	13.050842
H	7.754316	2.569893	11.797926
C	3.749730	5.958346	12.416208
C	3.415296	7.710899	10.995912
C	3.356930	8.198057	12.263075
C	3.286891	8.384375	9.679542
H	3.093753	9.450989	9.809981
H	2.467768	7.955737	9.089776
H	4.203177	8.268382	9.088232
C	3.135916	9.573341	12.776402
H	2.185456	9.666526	13.316880
H	3.124000	10.284995	11.948309
H	3.929457	9.870019	13.473015
C	3.934923	5.343471	10.100377
C	2.881535	4.444374	9.746368
C	3.225069	3.157912	9.439553

H	2.466628	2.435421	9.148355
C	4.595949	2.686175	9.708745
H	4.847947	1.747445	9.206171
C	5.614323	3.740609	9.521732
H	6.637281	3.438727	9.311343
C	5.309079	5.038481	9.817556
C	1.432974	4.896937	9.808311
H	1.426754	5.975345	10.014223
C	0.684922	4.204075	10.956240
H	-0.330301	4.606664	11.057919
H	1.212225	4.332764	11.906843
H	0.609124	3.126862	10.770105
C	0.716824	4.673805	8.471536
H	0.657568	3.606555	8.232155
H	1.246116	5.171318	7.652376
H	-0.307386	5.062674	8.512888
C	6.370015	6.120928	9.904968
H	5.916849	6.991881	10.398279
C	6.820339	6.555756	8.502575
H	7.305856	5.721141	7.984497
H	7.535221	7.385578	8.558663
H	5.967311	6.870603	7.892179
C	7.573099	5.692058	10.752945
H	7.250325	5.322699	11.732241
H	8.260186	6.533469	10.900640
H	8.133383	4.886301	10.267051
C	3.699302	7.210990	14.560485
C	4.979573	7.157317	15.144527
C	5.056978	7.283264	16.531542
H	6.023222	7.245347	17.022767
C	3.912745	7.458431	17.302221
H	4.000250	7.561194	18.380301
C	2.661360	7.490053	16.704519
H	1.776175	7.606821	17.322269
C	2.529564	7.357124	15.321111
C	6.226141	6.975836	14.292174
H	6.002982	6.181323	13.568376
C	6.569511	8.252919	13.506306
H	5.785566	8.524298	12.794753
H	7.494282	8.102245	12.938719
H	6.724262	9.093419	14.193207
C	7.453639	6.529545	15.089364
H	7.835099	7.334431	15.728825
H	8.254854	6.258603	14.393849
H	7.242322	5.658468	15.716161
C	1.160589	7.256018	14.675164
H	1.256505	7.526984	13.617467
C	0.698074	5.791428	14.725380
H	1.438142	5.132202	14.261521
H	-0.257641	5.667312	14.202941
H	0.567518	5.471965	15.766823
C	0.117993	8.185570	15.301860
H	-0.145233	7.875618	16.318854
H	-0.803065	8.163985	14.710057
H	0.473399	9.220755	15.345695
N	5.448208	1.140802	11.688536
N	4.857270	0.111789	13.834498
N	6.586167	-0.651488	12.725241
N	3.946271	4.753600	12.856352
N	3.654668	6.338336	11.082579
N	3.578044	7.115973	13.142468

Calculated energies and coordinates of $\mathbf{TS[2^{T}\text{-}1_{INT}^{T}]^{T}}$

Electronic energy ... -2635.92999477 Eh
Total Enthalpy ... -2634.59596157 Eh
Final Gibbs free energy ... -2634.76022844 Eh

CARTESIAN COORDINATES (ANGSTROEM)

B	0.123522	-0.784503	-0.930375
B	-0.323805	0.560873	-0.254775
C	0.808810	-2.951773	0.058716
C	0.349088	-4.474056	1.713715
C	1.438211	-4.926101	1.043737
C	-0.415038	-5.055777	2.846088
H	-0.571229	-4.317671	3.640977
H	0.121367	-5.909096	3.266763
H	-1.408199	-5.401091	2.531313
C	2.295542	-6.118304	1.260135
H	2.276102	-6.804559	0.404144
H	1.962153	-6.668611	2.142188
H	3.342037	-5.826353	1.411344
C	-1.105613	-2.431865	1.579858
C	-2.404663	-2.778849	1.183644
C	-3.459131	-1.986674	1.641528
H	-4.477221	-2.217299	1.342149
C	-3.218182	-0.896635	2.463576
H	-4.048115	-0.284576	2.806159
C	-1.920390	-0.565543	2.837187
H	-1.755641	0.313503	3.450525
C	-0.832882	-1.319943	2.401222
C	-2.652674	-3.909170	0.202509
H	-1.787949	-4.583202	0.227682
C	-3.894865	-4.739315	0.541760
H	-4.817118	-4.165724	0.400732
H	-3.873517	-5.091669	1.578660
H	-3.953295	-5.612215	-0.117209
C	-2.745852	-3.343216	-1.223845
H	-2.842301	-4.154447	-1.954878
H	-1.858896	-2.749785	-1.468502
H	-3.622061	-2.690700	-1.316392
C	0.595522	-0.940760	2.768383
H	1.184140	-1.003000	1.841842
C	0.732891	0.491951	3.289907
H	0.288464	1.220439	2.605719
H	1.797201	0.727173	3.397972
H	0.275521	0.609038	4.279902
C	1.212109	-1.912252	3.789736
H	0.606679	-1.941370	4.703496
H	2.217911	-1.572725	4.063055
H	1.301893	-2.928294	3.397977
C	2.858884	-4.011995	-0.824597
C	2.840282	-4.830911	-1.965425
C	3.966974	-4.826577	-2.786926
H	3.988892	-5.448154	-3.676584
C	5.062193	-4.024270	-2.489049
H	5.928675	-4.029618	-3.144570
C	5.053225	-3.215497	-1.361683
H	5.914324	-2.589715	-1.143684
C	3.953696	-3.196803	-0.501709
C	1.598112	-5.621983	-2.332618
H	1.066747	-5.867934	-1.405209
C	1.904246	-6.937435	-3.053427
H	2.294377	-6.766775	-4.062663
H	0.986588	-7.525454	-3.159096
H	2.636325	-7.539135	-2.503874
C	0.656252	-4.743148	-3.172751
H	0.390590	-3.828918	-2.633053
H	-0.262504	-5.289269	-3.416467
H	1.144195	-4.454272	-4.111169
C	3.961012	-2.305318	0.725296
H	3.062235	-2.527094	1.311346
C	5.169673	-2.589092	1.627057
H	5.215645	-3.645954	1.910776
H	5.104173	-1.990780	2.542575
H	6.111985	-2.332294	1.130958
C	3.903278	-0.823135	0.325410
H	4.813989	-0.528318	-0.209708
H	3.817244	-0.190243	1.217147
H	3.041519	-0.627248	-0.319116
C	-0.882220	2.909883	-0.263660
C	-1.356942	4.506676	-1.821945

C	-1.378964	5.108122	-0.603613
C	-1.585449	5.038176	-3.188082
H	-1.792583	6.109625	-3.155034
H	-2.432148	4.537953	-3.674348
H	-0.707242	4.873358	-3.824130
C	-1.667534	6.508369	-0.203359
H	-2.610093	6.590750	0.353042
H	-1.742431	7.144378	-1.087906
H	-0.879011	6.911097	0.443526
C	-0.817310	2.084215	-2.521171
C	-1.886733	1.187738	-2.783964
C	-1.553577	-0.098033	-3.144188
H	-2.325890	-0.845994	-3.303021
C	-0.185420	-0.500453	-3.091815
H	0.064365	-1.517024	-3.384556
C	0.806836	0.508800	-3.276480
H	1.822418	0.214376	-3.525986
C	0.518128	1.806580	-2.916591
C	-3.328406	1.594799	-2.543393
H	-3.343147	2.665365	-2.301586
C	-3.925618	0.845984	-1.342780
H	-4.934905	1.213563	-1.121131
H	-3.302688	0.965098	-0.450867
H	-3.991219	-0.227480	-1.551168
C	-4.182814	1.389396	-3.800103
H	-4.233167	0.329534	-4.072126
H	-3.766021	1.932966	-4.654484
H	-5.207722	1.739662	-3.630310
C	1.611230	2.852341	-2.801898
H	1.165692	3.768320	-2.391245
C	2.197806	3.191392	-4.178615
H	2.678587	2.311838	-4.620730
H	2.951509	3.983632	-4.096704
H	1.416622	3.523953	-4.870187
C	2.714786	2.402039	-1.833008
H	2.286730	2.073167	-0.879833
H	3.422347	3.218179	-1.643943
H	3.276578	1.557479	-2.246325
C	-0.900721	4.344390	1.756903
C	0.403922	4.425822	2.275592
C	0.538852	4.680555	3.640064
H	1.528476	4.748361	4.079568
C	-0.578195	4.842679	4.451681
H	-0.449277	5.045513	5.511258
C	-1.855136	4.729232	3.921077
H	-2.716760	4.833224	4.573459
C	-2.041804	4.467464	2.562445
C	1.614060	4.244126	1.375476
H	1.364571	3.450060	0.663418
C	1.916941	5.520681	0.574052
H	1.097062	5.783679	-0.100257
H	2.815298	5.375519	-0.036391
H	2.096903	6.364963	1.250074
C	2.868438	3.790086	2.124469
H	3.284436	4.589220	2.749113
H	3.639552	3.508432	1.399797
H	2.666378	2.922521	2.759688
C	-3.426019	4.210166	1.998959
H	-3.395102	4.388642	0.917758
C	-3.778035	2.727108	2.201441
H	-3.016574	2.079517	1.755796
H	-4.746145	2.492410	1.743775
H	-3.836515	2.494608	3.272112
C	-4.505901	5.118684	2.592604
H	-4.699086	4.885582	3.645207
H	-5.448344	4.978390	2.053110
H	-4.225856	6.175550	2.526507
N	0.786913	-1.984095	-0.786413
N	-0.022008	-3.247220	1.134959
N	1.717078	-4.001576	0.028778
N	-0.611819	1.765719	0.284086
N	-1.050043	3.159457	-1.619795
N	-1.073276	4.122205	0.357831

Calculated energies and coordinates of $\mathbf{TS[1_{INT}^{T}\text{-}C^{T}]^{T}}$

Electronic energy ... -2635.94209782 Eh
Total Enthalpy ... -2634.60810564 Eh
Final Gibbs free energy ... -2634.77447346 Eh

CARTESIAN COORDINATES (ANGSTROEM)

B	0.227890	-0.541364	0.268524
B	-0.187933	0.953972	0.119828
C	0.725851	-3.001735	0.467857
C	0.576313	-4.925963	1.692409
C	1.174308	-5.239400	0.515191
C	0.222357	-5.761212	2.866895
H	0.485760	-6.804614	2.682890
H	-0.851535	-5.710963	3.082661
H	0.746631	-5.430137	3.771891
C	1.685374	-6.525407	-0.019995
H	1.167530	-6.809932	-0.944439
H	1.545145	-7.324061	0.710865
H	2.754053	-6.460059	-0.258230
C	-0.301431	-2.784595	2.711149
C	-1.664575	-2.464884	2.628287
C	-2.212379	-1.694470	3.655666
H	-3.263266	-1.422082	3.618757
C	-1.431400	-1.263194	4.717297
H	-1.874705	-0.658348	5.503173
C	-0.081763	-1.588328	4.775249
H	0.518027	-1.229668	5.606054
C	0.512830	-2.350189	3.770931
C	-2.540184	-2.936081	1.483274
H	-1.899392	-3.434833	0.746825
C	-3.572995	-3.960802	1.974603
H	-4.258535	-3.507483	2.699200
H	-3.090559	-4.815876	2.459760
H	-4.169362	-4.334018	1.134626
C	-3.226475	-1.760778	0.775103
H	-3.773687	-2.118811	-0.104262
H	-2.489268	-1.020488	0.447555
H	-3.944476	-1.259250	1.433562
C	2.004722	-2.625894	3.778665
H	2.191780	-3.503572	3.147708
C	2.755035	-1.440834	3.147749
H	2.396231	-1.236552	2.134226
H	3.830575	-1.648422	3.104339
H	2.603558	-0.532640	3.742369
C	2.553276	-2.937405	5.174566
H	2.539819	-2.054992	5.823109
H	3.595192	-3.266159	5.099904
H	1.977798	-3.728161	5.667856
C	1.822438	-3.943028	-1.546414
C	0.998893	-4.197876	-2.654511
C	1.568285	-4.092240	-3.923252
H	0.961485	-4.281032	-4.803568
C	2.905677	-3.747467	-4.076050
H	3.332402	-3.670554	-5.072429
C	3.696785	-3.492384	-2.965008
H	4.737678	-3.212308	-3.101560
C	3.170905	-3.583674	-1.674939
C	-0.473180	-4.518423	-2.478926
H	-0.613414	-4.908454	-1.462735
C	-0.977896	-5.585183	-3.455601
H	-0.998070	-5.214691	-4.485994
H	-2.002314	-5.872113	-3.195973
H	-0.352574	-6.484110	-3.431404
C	-1.303789	-3.230041	-2.597435
H	-0.949648	-2.463814	-1.901328
H	-2.361538	-3.434435	-2.393085
H	-1.224372	-2.819263	-3.610957

C	4.033175	-3.260212	-0.470868
H	3.467527	-3.519141	0.432289
C	5.332094	-4.075307	-0.453422
H	5.132017	-5.150642	-0.509313
H	5.888440	-3.876940	0.469095
H	5.983492	-3.811171	-1.293409
C	4.322554	-1.753036	-0.420923
H	4.928648	-1.448072	-1.282515
H	4.875310	-1.497414	0.490126
H	3.392863	-1.177376	-0.440861
C	-0.835253	3.063426	-0.228470
C	-0.411009	4.189871	-2.156998
C	-1.097112	5.028568	-1.333698
C	0.100448	4.376495	-3.538815
H	0.023107	5.424479	-3.836678
H	-0.458553	3.770604	-4.262771
H	1.148856	4.068656	-3.615623
C	-1.589490	6.414640	-1.528217
H	-2.681517	6.468250	-1.434598
H	-1.315889	6.772856	-2.522759
H	-1.170279	7.107433	-0.787714
C	0.418661	1.759802	-1.834235
C	-0.188349	0.985659	-2.903802
C	0.597694	0.045942	-3.542905
H	0.155968	-0.606632	-4.290056
C	1.970858	-0.064745	-3.286931
H	2.558406	-0.819198	-3.799530
C	2.590261	0.841353	-2.417553
H	3.668963	0.793890	-2.285012
C	1.865360	1.789896	-1.726253
C	-1.658060	1.173700	-3.235663
H	-1.864463	2.253812	-3.214668
C	-2.589832	0.537644	-2.190467
H	-3.638028	0.736378	-2.447543
H	-2.400231	0.927109	-1.186760
H	-2.443283	-0.547379	-2.158645
C	-2.030737	0.673096	-4.633556
H	-1.989579	-0.420729	-4.692286
H	-1.363571	1.079609	-5.401162
H	-3.056841	0.971248	-4.873913
C	2.545402	2.738348	-0.756345
H	1.769350	3.327823	-0.252925
C	3.475759	3.724148	-1.476487
H	4.268222	3.192192	-2.014897
H	3.951647	4.400662	-0.756440
H	2.927916	4.332987	-2.202887
C	3.311358	1.976421	0.335625
H	2.651113	1.261627	0.837971
H	3.706860	2.673622	1.083895
H	4.157235	1.423256	-0.088943
C	-2.067160	4.828281	0.993039
C	-1.386593	5.655818	1.901149
C	-2.105290	6.157243	2.985562
H	-1.613847	6.803320	3.706269
C	-3.444508	5.829264	3.163798
H	-3.987969	6.226421	4.016760
C	-4.087807	4.991150	2.264733
H	-5.130861	4.732813	2.424871
C	-3.413336	4.475400	1.155698
C	0.101956	5.908393	1.752399
H	0.355277	5.838685	0.686892
C	0.538777	7.292417	2.239531
H	-0.058354	8.089712	1.783739
H	1.589430	7.460541	1.981152
H	0.456087	7.385469	3.327805
C	0.882975	4.800409	2.481162
H	0.686053	4.848088	3.558734
H	1.960602	4.925924	2.322814
H	0.588854	3.806027	2.129567
C	-4.120972	3.536788	0.197351
H	-3.462655	3.369187	-0.663447
C	-4.365141	2.176650	0.868909
H	-3.421223	1.746330	1.217259
H	-4.825994	1.479578	0.159457
H	-5.040304	2.286270	1.726067
C	-5.428724	4.135785	-0.335358
H	-6.170315	4.261721	0.460978
H	-5.864805	3.471470	-1.089367
H	-5.260492	5.114928	-0.796206
N	0.650160	-1.781261	0.077219
N	0.294556	-3.551221	1.666173
N	1.263571	-4.060440	-0.238541
N	-0.821618	2.085477	0.638862
N	-0.267752	2.979319	-1.473247
N	-1.353952	4.327315	-0.135744

Calculated energies and coordinates of **C**^{T}

Electronic energy ... -2635.95138492 Eh
Total Enthalpy ... -2634.61759213 Eh
Final Gibbs free energy ... -2634.78286716 Eh

CARTESIAN COORDINATES (ANGSTROEM)

B	0.170014	-0.637294	-0.224385
B	-0.187345	0.892985	-0.513867
C	0.768515	-3.003052	0.528741
C	0.722686	-4.757827	1.979815
C	1.450243	-5.143642	0.900247
C	0.357282	-5.477931	3.224748
H	0.721513	-4.943333	4.110464
H	0.793259	-6.478688	3.225657
H	-0.729702	-5.576540	3.334417
C	2.124965	-6.424719	0.576256
H	1.743379	-6.843175	-0.363213
H	1.954810	-7.154695	1.369809
H	3.207695	-6.297529	0.454009
C	-0.622063	-2.683313	2.540538
C	-1.991292	-2.973283	2.411293
C	-2.887512	-2.213683	3.161370
H	-3.953156	-2.406773	3.084813
C	-2.436581	-1.190271	3.986561
H	-3.151122	-0.598147	4.551265
C	-1.082307	-0.907140	4.077660
H	-0.746768	-0.088986	4.708191
C	-0.144371	-1.647649	3.355952
C	-2.486829	-4.001323	1.411048
H	-1.672558	-4.707840	1.207348
C	-3.682616	-4.812880	1.918501
H	-4.583702	-4.196643	2.004996
H	-3.482001	-5.257150	2.899365
H	-3.909773	-5.620265	1.214564
C	-2.824839	-3.303120	0.082234
H	-3.125212	-4.038734	-0.673436
H	-1.967535	-2.739750	-0.300340
H	-3.650092	-2.595075	0.220472
C	1.331640	-1.320025	3.467817
H	1.875931	-1.963209	2.766962
C	1.618618	0.137673	3.081283
H	1.215881	0.377948	2.092218
H	2.700232	0.315161	3.069589
H	1.172885	0.836456	3.797788
C	1.852016	-1.628258	4.879347
H	1.356729	-0.997577	5.626007
H	2.929246	-1.436658	4.939098
H	1.672803	-2.673790	5.152907
C	2.289284	-3.935765	-1.165650
C	1.678584	-4.001396	-2.425113
C	2.490603	-3.829878	-3.548201
H	2.047562	-3.870967	-4.539290
C	3.851917	-3.599887	-3.414847
H	4.467102	-3.466808	-4.300344
C	4.432818	-3.527027	-2.154157

H	5.496910	-3.329419	-2.067780
C	3.662852	-3.683389	-1.002395
C	0.190841	-4.240131	-2.590501
H	-0.252434	-4.323579	-1.591668
C	-0.079205	-5.558248	-3.329660
H	0.313031	-5.526137	-4.352065
H	-1.157108	-5.744545	-3.391079
H	0.388534	-6.405999	-2.817599
C	-0.485044	-3.058975	-3.300046
H	-0.272520	-2.114357	-2.791725
H	-1.571610	-3.201245	-3.319324
H	-0.140290	-2.965555	-4.336053
C	4.273120	-3.498802	0.375679
H	3.629968	-4.002471	1.107352
C	5.673078	-4.108647	0.500312
H	5.688688	-5.153325	0.171694
H	6.004008	-4.071354	1.543461
H	6.410817	-3.557270	-0.092201
C	4.293879	-2.005989	0.741697
H	4.943758	-1.452844	0.054172
H	4.672689	-1.866289	1.761142
H	3.293305	-1.566013	0.679270
C	-0.670034	3.005567	-0.280610
C	-0.579585	4.220374	-2.200863
C	-0.992332	5.051460	-1.201338
C	-0.353567	4.458029	-3.649809
H	0.697876	4.306506	-3.921386
H	-0.639161	5.475967	-3.924565
H	-0.937879	3.754946	-4.255483
C	-1.354830	6.489949	-1.198574
H	-2.381432	6.645967	-0.843355
H	-1.277853	6.899171	-2.208070
H	-0.696767	7.075006	-0.542689
C	-0.079261	1.591406	-2.063052
C	-1.186074	1.098278	-2.954907
C	-0.897045	0.556952	-4.179561
H	-1.708860	0.184331	-4.802306
C	0.423543	0.457360	-4.661285
H	0.614437	0.028121	-5.639551
C	1.496952	0.907032	-3.866331
H	2.509755	0.798298	-4.249627
C	1.302132	1.470732	-2.633010
C	-2.608130	1.191704	-2.435294
H	-2.556325	1.528941	-1.392375
C	-3.295783	-0.180469	-2.423071
H	-4.287031	-0.109398	-1.959151
H	-2.697078	-0.898491	-1.854008
H	-3.427992	-0.571373	-3.438410
C	-3.449383	2.214789	-3.212650
H	-3.521935	1.941386	-4.271779
H	-3.014767	3.218005	-3.147982
H	-4.467056	2.261087	-2.805397
C	2.454253	1.945407	-1.770676
H	2.051329	2.120059	-0.762622
C	3.047392	3.271490	-2.269763
H	3.407943	3.172578	-3.300524
H	3.893494	3.575889	-1.641931
H	2.302051	4.072980	-2.242119
C	3.545116	0.873639	-1.646005
H	3.109803	-0.085367	-1.348357
H	4.287778	1.169110	-0.895385
H	4.071813	0.725726	-2.595770
C	-1.556949	4.742772	1.246984
C	-0.684061	5.343507	2.166373
C	-1.217835	5.775359	3.380397
H	-0.573788	6.245848	4.116868
C	-2.568568	5.607689	3.662311
H	-2.966855	5.949348	4.613848
C	-3.410856	5.005124	2.738245
H	-4.462720	4.875294	2.976934
C	-2.922335	4.562895	1.507312
C	0.799557	5.445676	1.868122
H	0.917689	5.481637	0.777683
C	1.449467	6.704189	2.448870
H	0.905549	7.610023	2.159946
H	2.478826	6.791501	2.085717
H	1.496011	6.669418	3.542846
C	1.515680	4.177764	2.365835
H	1.438393	4.106284	3.457571
H	2.578576	4.210537	2.099293
H	1.070983	3.274430	1.935295
C	-3.835175	3.868494	0.515560
H	-3.308615	3.808355	-0.444194
C	-4.123072	2.431126	0.977700
H	-3.191614	1.875428	1.127535
H	-4.728795	1.906543	0.228888
H	-4.680150	2.436230	1.922560
C	-5.136404	4.641739	0.273425
H	-5.770167	4.661485	1.166712
H	-5.711438	4.161215	-0.525500
H	-4.938014	5.677331	-0.022484
N	0.588525	-1.853100	-0.011913
N	0.308008	-3.434969	1.760688
N	1.479152	-4.064913	0.002469
N	-0.600893	1.904900	0.430867
N	-0.360062	2.978763	-1.613137
N	-1.032306	4.295410	-0.002427

Calculated energies and coordinates of **TS[C^T-D^T]^T**

Electronic energy ... -2635.93282800 Eh
Total Enthalpy ... -2634.60029131 Eh
Final Gibbs free energy ... -2634.76765120 Eh

CARTESIAN COORDINATES (ANGSTROEM)

B	0.231561	-0.559120	-0.225751
B	-0.080998	0.956813	-0.643172
C	0.745137	-2.934250	0.582201
C	0.656085	-4.700890	2.014488
C	1.377621	-5.092613	0.933237
C	0.266245	-5.426766	3.248519
H	0.627775	-4.903852	4.142252
H	0.688165	-6.433490	3.246303
H	-0.823218	-5.511032	3.345132
C	2.025947	-6.385185	0.601727
H	1.640256	-6.786319	-0.343606
H	1.836390	-7.118367	1.387876
H	3.111512	-6.279563	0.485189
C	-0.654743	-2.603777	2.583162
C	-2.026999	-2.872334	2.439795
C	-2.920081	-2.098900	3.179719
H	-3.987648	-2.275503	3.090060
C	-2.463294	-1.081930	4.009368
H	-3.174813	-0.478886	4.566346
C	-1.105846	-0.818597	4.113665
H	-0.764866	-0.004797	4.746980
C	-0.171414	-1.572663	3.401347
C	-2.529845	-3.887182	1.430099
H	-1.719932	-4.595090	1.215435
C	-3.728173	-4.698816	1.931609
H	-4.624788	-4.078341	2.032701
H	-3.525125	-5.158982	2.904603
H	-3.964308	-5.494236	1.217039
C	-2.868142	-3.169532	0.111898
H	-3.164795	-3.893545	-0.656278
H	-2.011997	-2.596254	-0.258537
H	-3.695891	-2.466300	0.260096
C	1.307033	-1.260981	3.523509
H	1.849619	-1.916308	2.832964
C	1.613172	0.190050	3.126248
H	1.228494	0.424576	2.128579
H	2.696457	0.357114	3.128515

H	1.164163	0.900154	3.829438
C	1.812533	-1.562697	4.941830
H	1.319811	-0.919541	5.679472
H	2.891549	-1.383729	5.008382
H	1.618421	-2.603613	5.222800
C	2.316219	-3.861579	-1.070429
C	1.785700	-3.823775	-2.367155
C	2.679698	-3.679614	-3.430445
H	2.298505	-3.649108	-4.447151
C	4.043656	-3.567658	-3.206485
H	4.723506	-3.458412	-4.046892
C	4.543269	-3.568562	-1.910006
H	5.609821	-3.443366	-1.749754
C	3.690429	-3.699105	-0.814752
C	0.297386	-3.920483	-2.640066
H	-0.224308	-3.945808	-1.677340
C	-0.040989	-5.217094	-3.389298
H	0.436868	-5.238119	-4.375103
H	-1.123260	-5.299441	-3.538606
H	0.297966	-6.097820	-2.833328
C	-0.206712	-2.690660	-3.408537
H	0.060213	-1.761185	-2.895966
H	-1.297687	-2.724939	-3.503850
H	0.214016	-2.648719	-4.419493
C	4.221374	-3.550948	0.600148
H	3.503256	-4.006364	1.292178
C	5.569616	-4.245045	0.818196
H	5.540973	-5.291872	0.497454
H	5.834881	-4.218715	1.880277
H	6.376995	-3.746631	0.271519
C	4.313017	-2.057501	0.956963
H	5.045965	-1.557144	0.314053
H	4.627020	-1.931355	1.999929
H	3.350875	-1.552920	0.818839
C	-0.513069	3.123468	-0.425245
C	-0.556358	4.457665	-2.223062
C	-1.139148	5.153897	-1.195224
C	-0.307142	4.849224	-3.636333
H	0.767885	4.900247	-3.847441
H	-0.752546	5.819927	-3.866656
H	-0.732238	4.102136	-4.318091
C	-1.672838	6.535677	-1.109190
H	-2.705317	6.539809	-0.736760
H	-1.663626	7.005286	-2.095078
H	-1.085409	7.162706	-0.424915
C	0.008382	1.356262	-2.227359
C	-1.189845	1.181631	-3.031516
C	-1.080163	0.932732	-4.389787
H	-1.979699	0.800758	-4.987096
C	0.165786	0.820736	-5.009042
H	0.226039	0.621024	-6.075248
C	1.338203	0.954343	-4.254539
H	2.301308	0.846951	-4.748753
C	1.287869	1.214262	-2.901228
C	-2.556512	1.250982	-2.381100
H	-2.408361	1.520173	-1.329823
C	-3.239196	-0.124026	-2.398963
H	-4.203294	-0.082720	-1.877829
H	-2.608901	-0.866763	-1.899623
H	-3.422018	-0.461697	-3.425971
C	-3.451462	2.318872	-3.023255
H	-3.684364	2.075846	-4.066171
H	-2.969292	3.302509	-3.000411
H	-4.401415	2.391788	-2.480301
C	2.558869	1.388498	-2.099119
H	2.258743	1.587967	-1.061135
C	3.371390	2.594069	-2.591691
H	3.723112	2.444277	-3.619066
H	4.250176	2.746384	-1.954706
H	2.763303	3.503645	-2.564436
C	3.403153	0.106830	-2.107527
H	2.831577	-0.746379	-1.725864
H	4.292916	0.232817	-1.479840
H	3.738768	-0.141167	-3.121052
C	-1.585568	4.640076	1.238718
C	-0.744157	5.320929	2.134566
C	-1.250337	5.637432	3.394577
H	-0.627500	6.166427	4.109402
C	-2.544398	5.276978	3.751458
H	-2.923174	5.530564	4.737943
C	-3.351568	4.588857	2.857305
H	-4.356494	4.301924	3.154713
C	-2.889854	4.258533	1.581207
C	0.697193	5.617102	1.767118
H	0.757350	5.696361	0.674611
C	1.220177	6.929368	2.357241
H	0.555889	7.769237	2.126464
H	2.210289	7.151725	1.945677
H	1.329707	6.871590	3.445674
C	1.583865	4.432541	2.190486
H	1.575059	4.328065	3.282265
H	2.619240	4.595430	1.869399
H	1.223060	3.493363	1.758540
C	-3.766812	3.469374	0.631106
H	-3.279159	3.467682	-0.349921
C	-3.878751	2.010878	1.102603
H	-2.885508	1.562452	1.205790
H	-4.458486	1.421354	0.381948
H	-4.390354	1.958271	2.071402
C	-5.151818	4.103135	0.453025
H	-5.734157	4.072076	1.380351
H	-5.719186	3.557199	-0.308911
H	-5.075494	5.149351	0.138035
N	0.593661	-1.777941	0.051208
N	0.273065	-3.365936	1.810021
N	1.429444	-4.008780	0.040056
N	-0.454902	2.010890	0.264490
N	-0.082363	3.262730	-1.722514
N	-1.072536	4.329340	-0.055745

Calculated energies and coordinates of **D**^{T}

Electronic energy ... -2635.97819815 Eh
Total Enthalpy ... -2634.64365089 Eh
Final Gibbs free energy ... -2634.81105644 Eh

CARTESIAN COORDINATES (ANGSTROEM)

B	0.574423	-0.287910	-0.420999
B	-0.134418	0.938181	-1.189095
C	1.078835	-2.536883	0.652802
C	0.792077	-4.084236	2.296867
C	1.493511	-4.678935	1.297173
C	0.301926	-4.593361	3.600965
H	0.732685	-4.026692	4.435887
H	0.570150	-5.643972	3.726653
H	-0.788401	-4.503113	3.678479
C	2.022443	-6.058029	1.155557
H	1.651940	-6.530530	0.238205
H	1.713022	-6.669893	2.005211
H	3.118163	-6.073287	1.104810
C	-0.274643	-1.783042	2.545687
C	-1.627416	-1.696750	2.182896
C	-2.364455	-0.626772	2.690131
H	-3.408202	-0.512294	2.411601
C	-1.775263	0.310668	3.527575
H	-2.353727	1.158861	3.881666
C	-0.445570	0.177161	3.904567
H	-0.003077	0.915380	4.566991
C	0.334992	-0.872108	3.419465
C	-2.276604	-2.712639	1.262144
H	-1.542800	-3.501670	1.057001
C	-3.489522	-3.373549	1.931684

H	-4.290627	-2.646602	2.103549
H	-3.224617	-3.813698	2.898967
H	-3.891217	-4.165429	1.290119
C	-2.671184	-2.086842	-0.082531
H	-3.043567	-2.860468	-0.764278
H	-1.821217	-1.581330	-0.554691
H	-3.468109	-1.345304	0.046185
C	1.801964	-0.984353	3.787336
H	2.166622	-1.956460	3.433073
C	2.623558	0.104068	3.082163
H	2.451707	0.092047	2.001147
H	3.693506	-0.041438	3.268601
H	2.346393	1.095780	3.454518
C	2.016067	-0.933681	5.305326
H	1.746321	0.045615	5.714810
H	3.070221	-1.111050	5.544621
H	1.412897	-1.688910	5.820309
C	2.419502	-3.887311	-0.923381
C	1.734070	-4.067825	-2.133156
C	2.499730	-4.218982	-3.291481
H	2.000895	-4.356055	-4.246405
C	3.885145	-4.185148	-3.242290
H	4.461263	-4.301382	-4.155919
C	4.540297	-3.982518	-2.034106
H	5.624656	-3.932883	-2.016122
C	3.821101	-3.819507	-0.851051
C	0.220767	-4.104718	-2.218394
H	-0.184595	-3.887123	-1.223591
C	-0.267777	-5.503624	-2.621768
H	0.082403	-5.768022	-3.625663
H	-1.362901	-5.535872	-2.628963
H	0.094254	-6.269684	-1.927566
C	-0.311909	-3.034316	-3.179993
H	0.033293	-2.034069	-2.902890
H	-1.407130	-3.029979	-3.168845
H	0.005561	-3.224227	-4.211009
C	4.528646	-3.476798	0.446990
H	3.859762	-3.722119	1.280701
C	5.828821	-4.260315	0.653729
H	5.674687	-5.338184	0.535213
H	6.217304	-4.077256	1.661034
H	6.606286	-3.951346	-0.052817
C	4.787343	-1.961991	0.501082
H	5.467691	-1.663936	-0.304678
H	5.243236	-1.684241	1.458293
H	3.856859	-1.398736	0.379683
C	-0.691743	3.238860	-0.667075
C	-0.204853	5.197039	-1.497436
C	-1.101136	5.443287	-0.440535
C	0.383160	6.210809	-2.421311
H	1.227667	5.766001	-2.951981
H	0.730429	7.099137	-1.883176
H	-0.348867	6.540700	-3.168590
C	-1.662470	6.711269	0.084045
H	-2.759267	6.686302	0.096991
H	-1.342971	7.549887	-0.538549
H	-1.339557	6.903300	1.115783
C	-0.114496	0.782809	-2.777405
C	-1.314480	0.662697	-3.517908
C	-1.269958	0.397339	-4.887091
H	-2.196070	0.304179	-5.449389
C	-0.058123	0.250029	-5.548659
H	-0.037752	0.035685	-6.614186
C	1.125169	0.395840	-4.839686
H	2.074395	0.310070	-5.363636
C	1.116358	0.663449	-3.469622
C	-2.673111	0.853307	-2.865156
H	-2.523636	0.874854	-1.779569
C	-3.650755	-0.287241	-3.173030
H	-4.570407	-0.172393	-2.587127
H	-3.208795	-1.257605	-2.926215
H	-3.937071	-0.305213	-4.230432
C	-3.263932	2.207385	-3.284809
H	-3.418841	2.239814	-4.369795
H	-2.582147	3.023988	-3.022129
H	-4.233024	2.385930	-2.802166
C	2.453011	0.882948	-2.778452
H	2.247071	1.075273	-1.717274
C	3.150532	2.133270	-3.332364
H	3.409369	2.009825	-4.390694
H	4.077077	2.333063	-2.780592
H	2.492901	3.003144	-3.237587
C	3.359141	-0.350539	-2.861284
H	2.865467	-1.232093	-2.438850
H	4.290312	-0.182278	-2.307108
H	3.626687	-0.582575	-3.898902
C	-2.030238	4.017561	1.353994
C	-1.291538	4.340675	2.508705
C	-1.927539	4.227711	3.744383
H	-1.385472	4.476848	4.651771
C	-3.246780	3.798448	3.832685
H	-3.729505	3.726944	4.803784
C	-3.941364	3.446448	2.685043
H	-4.966246	3.094040	2.765520
C	-3.346503	3.545054	1.424613
C	0.177889	4.710928	2.428105
H	0.387774	5.087018	1.420638
C	0.588626	5.803607	3.420412
H	-0.059237	6.683682	3.346095
H	1.617989	6.118770	3.220009
H	0.556154	5.447756	4.455851
C	1.028455	3.446691	2.628445
H	0.893989	3.066511	3.648029
H	2.091509	3.669550	2.481528
H	0.737642	2.654817	1.930520
C	-4.116075	3.133366	0.188969
H	-3.456171	3.261554	-0.673892
C	-4.485155	1.647159	0.264946
H	-3.582830	1.047579	0.416593
H	-4.966686	1.324651	-0.664859
H	-5.181404	1.449296	1.089027
C	-5.356220	4.009403	-0.026529
H	-6.072323	3.899085	0.795753
H	-5.865288	3.725659	-0.954401
H	-5.086032	5.068437	-0.095674
N	1.041624	-1.444039	-0.024437
N	0.541937	-2.763068	1.904011
N	1.671947	-3.724017	0.283089
N	-0.834357	1.974436	-0.453243
N	0.060829	3.891024	-1.617422
N	-1.412101	4.216101	0.080367

Calculated energies and coordinates of **E**

Electronic energy ... -2636.02187944 Eh
Total Enthalpy ... -2634.68584072 Eh
Final Gibbs free energy ... -2634.85245615 Eh

CARTESIAN COORDINATES (ANGSTROEM)

B	0.310586	-0.361834	-0.562100
B	0.069395	1.063351	-1.365068
C	0.572126	-2.606623	0.677694
C	0.364408	-4.272368	2.187830
C	1.248252	-4.686674	1.239455
C	-0.147668	-4.935974	3.412021
H	0.008526	-4.304230	4.293739
H	0.369091	-5.884010	3.572251
H	-1.223263	-5.137063	3.345735
C	2.008898	-5.951424	1.088719
H	1.819464	-6.410196	0.111680
H	1.716329	-6.661968	1.863759
H	3.089978	-5.785485	1.163684

C	-1.018901	-2.192366	2.533793
C	-2.375309	-2.529531	2.393244
C	-3.295757	-1.810838	3.153465
H	-4.353368	-2.042615	3.078393
C	-2.879193	-0.779413	3.987075
H	-3.612999	-0.225248	4.566039
C	-1.540559	-0.428849	4.057463
H	-1.236411	0.413755	4.671226
C	-0.574606	-1.132302	3.333681
C	-2.833209	-3.548458	1.366609
H	-2.009254	-4.247418	1.173819
C	-4.039635	-4.374905	1.820231
H	-4.947191	-3.765681	1.881641
H	-3.872634	-4.833011	2.801135
H	-4.235957	-5.172869	1.096826
C	-3.135113	-2.822745	0.042501
H	-3.401791	-3.542437	-0.740082
H	-2.275510	-2.237299	-0.299470
H	-3.972680	-2.127637	0.170042
C	0.879245	-0.715557	3.417557
H	1.464494	-1.354192	2.746417
C	1.057643	0.736567	2.951539
H	0.617089	0.915321	1.964373
H	2.124221	0.982115	2.899032
H	0.587600	1.439130	3.648864
C	1.433889	-0.915655	4.834876
H	0.904875	-0.284924	5.557338
H	2.494405	-0.643030	4.866848
H	1.336175	-1.956846	5.161590
C	2.294656	-3.607674	-0.788095
C	1.823676	-3.847665	-2.084953
C	2.766188	-3.849400	-3.116129
H	2.439342	-4.025804	-4.136819
C	4.107359	-3.613276	-2.858583
H	4.822517	-3.616399	-3.676200
C	4.539555	-3.340966	-1.566239
H	5.587874	-3.123223	-1.388783
C	3.640369	-3.322021	-0.501744
C	0.358940	-4.078824	-2.401144
H	-0.214854	-3.996428	-1.470384
C	0.129764	-5.489699	-2.960901
H	0.655030	-5.626824	-3.912106
H	-0.937510	-5.656330	-3.141911
H	0.485366	-6.259353	-2.267183
C	-0.168022	-3.006518	-3.366269
H	-0.006531	-1.995808	-2.979304
H	-1.242471	-3.140692	-3.530626
H	0.325068	-3.065293	-4.342053
C	4.084912	-2.906431	0.888897
H	3.366275	-3.303272	1.617381
C	5.466156	-3.447226	1.270623
H	5.534264	-4.529770	1.119103
H	5.669713	-3.234232	2.324932
H	6.261952	-2.973516	0.686473
C	4.052950	-1.371508	0.997660
H	4.784204	-0.927451	0.312955
H	4.299079	-1.053438	2.017176
H	3.069942	-0.967832	0.735608
C	-0.365683	3.379284	-0.862385
C	-0.639477	5.284218	-1.868761
C	-1.061703	5.497714	-0.570682
C	-0.620680	6.246573	-3.008827
H	0.397449	6.363475	-3.398147
H	-0.989479	7.233434	-2.715424
H	-1.238992	5.878234	-3.835264
C	-1.644967	6.678452	0.119199
H	-2.622929	6.447594	0.561228
H	-1.781954	7.495484	-0.592434
H	-1.008241	7.046818	0.935357
C	0.005654	0.951715	-2.953433
C	-1.222134	0.725954	-3.606840
C	-1.245210	0.468755	-4.978790
H	-2.193621	0.309582	-5.487293
C	-0.063995	0.420284	-5.709535
H	-0.090973	0.214084	-6.776656
C	1.150221	0.655871	-5.074959
H	2.069051	0.636425	-5.657040
C	1.194192	0.929273	-3.707207
C	-2.524651	0.820225	-2.830310
H	-2.261282	0.863613	-1.765510
C	-3.434877	-0.396410	-3.029657
H	-4.324190	-0.318255	-2.392298
H	-2.911724	-1.324689	-2.774255
H	-3.780204	-0.478486	-4.066627
C	-3.259361	2.124661	-3.169564
H	-3.577579	2.127841	-4.219218
H	-2.600971	2.984604	-3.010496
H	-4.153378	2.241918	-2.543819
C	2.524446	1.201659	-3.025000
H	2.296172	1.577468	-2.017670
C	3.328087	2.294491	-3.737411
H	3.652266	1.972943	-4.733800
H	4.227040	2.546046	-3.162368
H	2.722432	3.199079	-3.848446
C	3.340915	-0.087790	-2.874930
H	2.779716	-0.847210	-2.317968
H	4.282777	0.100066	-2.344368
H	3.583589	-0.512088	-3.856711
C	-1.124810	4.010968	1.444717
C	-0.219767	4.493031	2.407040
C	-0.501637	4.253757	3.751913
H	0.174685	4.621349	4.517800
C	-1.636959	3.543436	4.126614
H	-1.846917	3.375736	5.179973
C	-2.498806	3.047596	3.158943
H	-3.377977	2.483764	3.460447
C	-2.260706	3.270325	1.800720
C	1.075461	5.158946	1.980934
H	0.911482	5.629680	1.004980
C	1.555371	6.243868	2.948078
H	0.769672	6.979013	3.153938
H	2.413063	6.771157	2.517133
H	1.883871	5.821519	3.904354
C	2.156005	4.081579	1.785700
H	2.383978	3.599804	2.744414
H	3.078035	4.530336	1.398277
H	1.817516	3.310573	1.085732
C	-3.222587	2.724953	0.763605
H	-2.816780	2.950507	-0.227463
C	-3.347695	1.200898	0.876793
H	-2.360687	0.730509	0.812093
H	-3.973817	0.809782	0.065986
H	-3.807861	0.905505	1.826320
C	-4.595282	3.402485	0.871363
H	-5.065330	3.200029	1.840997
H	-5.265368	3.031861	0.087229
H	-4.507873	4.488283	0.760272
N	0.436413	-1.481484	0.054994
N	-0.051680	-2.984618	1.828655
N	1.370932	-3.647927	0.309748
N	-0.120690	2.111956	-0.478733
N	-0.215899	3.992619	-2.040896
N	-0.868405	4.284996	0.072884

Calculated energies and coordinates of **TS[E-F]**

Electronic energy ... -2636.01949916 Eh
Total Enthalpy ... -2634.68417156 Eh
Final Gibbs free energy ... -2634.84764742 Eh

CARTESIAN COORDINATES (ANGSTROEM)

B	0.248761	-0.342169	-0.644316

B	0.077284	0.741574	-1.844292
C	0.536562	-2.177458	0.996186
C	0.612683	-3.950685	2.408427
C	1.546885	-4.160601	1.444290
C	0.196265	-4.764931	3.576593
H	0.347226	-4.223659	4.518248
H	0.773095	-5.690575	3.617936
H	-0.867959	-5.022164	3.519355
C	2.530448	-5.255104	1.249493
H	2.525456	-5.613260	0.214442
H	2.296910	-6.094532	1.907554
H	3.552311	-4.922865	1.470533
C	-1.130409	-2.172327	2.810634
C	-2.409433	-2.489586	2.334741
C	-3.497464	-1.920189	2.997918
H	-4.505729	-2.132190	2.653932
C	-3.306071	-1.079253	4.084955
H	-4.165481	-0.639795	4.583915
C	-2.025343	-0.795427	4.543206
H	-1.897751	-0.137669	5.396687
C	-0.905460	-1.343838	3.918988
C	-2.625230	-3.396101	1.136963
H	-1.666237	-3.865901	0.883943
C	-3.615476	-4.526660	1.445230
H	-4.626002	-4.140592	1.614786
H	-3.317652	-5.091511	2.334940
H	-3.666232	-5.219369	0.598522
C	-3.083716	-2.587279	-0.086006
H	-3.197752	-3.243852	-0.956274
H	-2.366277	-1.800160	-0.334555
H	-4.050897	-2.108618	0.104638
C	0.502969	-1.030302	4.388436
H	1.146516	-1.872256	4.100126
C	1.037963	0.225528	3.682576
H	0.992948	0.134076	2.594098
H	2.077385	0.411455	3.977413
H	0.439850	1.100088	3.955620
C	0.603691	-0.876766	5.908938
H	0.104294	0.034499	6.254620
H	1.655096	-0.798528	6.204069
H	0.160701	-1.729438	6.434901
C	2.297357	-2.896717	-0.598028
C	1.747530	-3.206430	-1.853076
C	2.597065	-3.126530	-2.956112
H	2.213319	-3.349256	-3.945953
C	3.925234	-2.748992	-2.807900
H	4.570317	-2.701126	-3.679815
C	4.423591	-2.389674	-1.564080
H	5.451307	-2.050934	-1.477747
C	3.614297	-2.445785	-0.429087
C	0.291843	-3.614449	-2.007522
H	-0.284061	-3.086632	-1.237730
C	0.110002	-5.122167	-1.771844
H	0.707827	-5.698183	-2.487566
H	-0.941592	-5.400296	-1.905603
H	0.407221	-5.415908	-0.759910
C	-0.302238	-3.202730	-3.357716
H	-0.129736	-2.142271	-3.570671
H	-1.383712	-3.373317	-3.344901
H	0.106165	-3.796910	-4.182664
C	4.113140	-1.937480	0.910361
H	3.489213	-2.372072	1.701740
C	5.565115	-2.328706	1.202878
H	5.725770	-3.405440	1.082725
H	5.825240	-2.054607	2.230415
H	6.266052	-1.807142	0.543067
C	3.933585	-0.410001	0.968248
H	4.543885	0.075494	0.198133
H	4.240101	-0.022093	1.946135
H	2.891862	-0.122103	0.796387
C	-0.336344	3.045865	-1.108889
C	-0.654665	5.014874	-1.959203

C	-0.902912	5.155562	-0.609153
C	-0.729673	6.048021	-3.032616
H	-1.057233	7.015573	-2.642709
H	-1.428627	5.736744	-3.817042
H	0.248161	6.183564	-3.508814
C	-1.324143	6.312714	0.224065
H	-0.553499	6.608238	0.948955
H	-2.231440	6.090225	0.800071
H	-1.530456	7.174704	-0.414351
C	-0.086479	0.680178	-3.409766
C	-1.382509	0.650271	-3.958960
C	-1.550499	0.531123	-5.340928
H	-2.551146	0.510100	-5.767040
C	-0.450700	0.441786	-6.180544
H	-0.589741	0.350964	-7.254905
C	0.834039	0.471932	-5.646154
H	1.684241	0.403211	-6.317870
C	1.029816	0.588160	-4.270454
C	-2.611817	0.748277	-3.069176
H	-2.267475	0.771645	-2.027446
C	-3.527248	-0.473589	-3.217920
H	-4.356844	-0.423356	-2.502096
H	-2.972685	-1.400327	-3.035714
H	-3.957676	-0.532716	-4.224049
C	-3.381300	2.051421	-3.322163
H	-3.793001	2.074376	-4.338326
H	-2.718739	2.914073	-3.201121
H	-4.219495	2.148790	-2.620082
C	2.427937	0.665952	-3.677360
H	2.423820	0.041906	-2.767450
C	2.757319	2.105519	-3.249793
H	2.797860	2.757068	-4.130788
H	3.731834	2.142758	-2.746864
H	1.996736	2.512725	-2.579869
C	3.528801	0.140399	-4.599837
H	3.294417	-0.855486	-4.990069
H	4.476560	0.080503	-4.053328
H	3.685122	0.812631	-5.451542
C	-0.889783	3.568621	1.304488
C	0.108677	3.907061	2.235160
C	-0.164965	3.708337	3.587290
H	0.575741	3.979035	4.333648
C	-1.375976	3.158077	3.995890
H	-1.573551	3.013707	5.055308
C	-2.323850	2.780578	3.057060
H	-3.255839	2.327464	3.384972
C	-2.104397	2.988933	1.692582
C	1.464973	4.390535	1.758113
H	1.315434	4.947842	0.825241
C	2.173763	5.315078	2.749537
H	1.532959	6.148578	3.057360
H	3.076589	5.730026	2.289496
H	2.489410	4.777263	3.650744
C	2.343953	3.170194	1.426429
H	2.540123	2.590190	2.336822
H	3.304944	3.491028	1.007709
H	1.847547	2.511926	0.706211
C	-3.157144	2.583352	0.681122
H	-2.793004	2.851045	-0.316151
C	-3.361832	1.063527	0.708953
H	-2.406747	0.544976	0.582107
H	-4.039276	0.755087	-0.096268
H	-3.799152	0.740406	1.661061
C	-4.481023	3.324809	0.907099
H	-4.920371	3.070519	1.878420
H	-5.203851	3.051710	0.130178
H	-4.338996	4.410016	0.873814
N	0.191917	-1.041573	0.468896
N	0.003186	-2.722103	2.129460
N	1.484996	-3.066522	0.568198
N	-0.077430	1.734858	-0.880614
N	-0.308460	3.723079	-2.256937

N	-0.683306	3.896810	-0.066668

Calculated energies and coordinates of **F**

Electronic energy	...	-2636.04178504 Eh
Total Enthalpy	...	-2634.70429014 Eh
Final Gibbs free energy	...	-2634.86638260 Eh

CARTESIAN COORDINATES (ANGSTROEM)

B	0.185678	0.039913	-0.527527
B	0.083304	0.442071	-2.082685
C	0.515747	-1.794655	1.066181
C	0.632826	-3.677657	2.376115
C	1.581078	-3.799338	1.418484
C	0.216405	-4.589215	3.471021
H	0.324020	-4.121933	4.457435
H	0.821922	-5.497468	3.457717
H	-0.836822	-4.876612	3.365849
C	2.581263	-4.867936	1.165429
H	2.558504	-5.197633	0.120595
H	2.376214	-5.730595	1.803146
H	3.604337	-4.529824	1.372263
C	-1.184364	-2.020342	2.850569
C	-2.432904	-2.370233	2.318705
C	-3.568727	-1.900911	2.981477
H	-4.554691	-2.140322	2.592471
C	-3.453061	-1.133330	4.131609
H	-4.348177	-0.772178	4.631232
C	-2.201984	-0.844927	4.666122
H	-2.133974	-0.270135	5.584176
C	-1.039038	-1.295092	4.042459
C	-2.563795	-3.254944	1.091180
H	-1.569198	-3.647997	0.848890
C	-3.473039	-4.461326	1.364912
H	-4.514570	-4.155377	1.510966
H	-3.156426	-5.009240	2.258570
H	-3.448188	-5.149183	0.512693
C	-3.062063	-2.474612	-0.132675
H	-3.154937	-3.144086	-0.995729
H	-2.375263	-1.665264	-0.396101
H	-4.047163	-2.034760	0.060116
C	0.341378	-1.028916	4.616527
H	0.983045	-1.869565	4.323374
C	0.959820	0.240745	4.015731
H	0.925647	0.229073	2.922294
H	2.001934	0.343614	4.342111
H	0.406841	1.122884	4.349798
C	0.349313	-0.958821	6.145987
H	-0.160510	-0.060203	6.510304
H	1.381649	-0.911510	6.507813
H	-0.134280	-1.832041	6.597274
C	2.266675	-2.539271	-0.601991
C	1.687204	-2.949192	-1.815456
C	2.517968	-3.003683	-2.934530
H	2.108474	-3.308203	-3.891773
C	3.862132	-2.667211	-2.845096
H	4.492077	-2.730450	-3.727285
C	4.400115	-2.225624	-1.645014
H	5.447319	-1.944795	-1.600164
C	3.611984	-2.155092	-0.495419
C	0.222711	-3.345031	-1.905435
H	-0.331554	-2.682018	-1.230123
C	0.000461	-4.796960	-1.448585
H	0.589698	-5.486424	-2.064903
H	-1.056554	-5.065852	-1.560766
H	0.273033	-4.948525	-0.400360
C	-0.368735	-3.151027	-3.303529
H	-0.176023	-2.146715	-3.690968
H	-1.453416	-3.294342	-3.257183
H	0.020363	-3.887443	-4.016289
C	4.171919	-1.632845	0.814726
H	3.645962	-2.138296	1.634666
C	5.669123	-1.896942	0.990265
H	5.921851	-2.948174	0.814074
H	5.973350	-1.638072	2.009626
H	6.270741	-1.284239	0.310205
C	3.865682	-0.132445	0.940423
H	4.333578	0.424329	0.119799
H	4.250201	0.261952	1.887953
H	2.788875	0.052714	0.902365
C	-0.156247	2.777860	-1.063367
C	-0.389369	4.772954	-1.850353
C	-0.663220	4.871072	-0.506570
C	-0.399713	5.839563	-2.891794
H	-0.718507	6.801151	-2.481482
H	-1.075558	5.570353	-3.710736
H	0.598562	5.966919	-3.325535
C	-1.078702	6.013700	0.350593
H	-0.301033	6.310151	1.066457
H	-1.975212	5.778122	0.936405
H	-1.300648	6.878955	-0.278036
C	-0.132084	0.488431	-3.618305
C	-1.450808	0.515197	-4.111565
C	-1.672727	0.501975	-5.489430
H	-2.689068	0.522990	-5.875312
C	-0.605964	0.466050	-6.375878
H	-0.787573	0.454805	-7.447328
C	0.698646	0.445827	-5.892441
H	1.522425	0.418517	-6.599002
C	0.952277	0.456944	-4.521968
C	-2.633909	0.581910	-3.159479
H	-2.251528	0.442744	-2.137818
C	-3.655542	-0.533865	-3.408178
H	-4.434123	-0.517721	-2.636430
H	-3.173826	-1.516505	-3.390506
H	-4.149504	-0.417091	-4.379105
C	-3.300752	1.963493	-3.216588
H	-3.726623	2.144176	-4.210557
H	-2.571850	2.754111	-3.011806
H	-4.114851	2.034081	-2.484289
C	2.369125	0.482952	-3.976539
H	2.372459	-0.145318	-3.070011
C	2.766461	1.907155	-3.555190
H	2.812861	2.560256	-4.435078
H	3.754108	1.901895	-3.077714
H	2.040351	2.345350	-2.866215
C	3.415954	-0.078352	-4.939311
H	3.136140	-1.067739	-5.316086
H	4.381174	-0.165197	-4.429469
H	3.560594	0.587119	-5.798222
C	-0.785629	3.270660	1.368507
C	0.142437	3.666930	2.349094
C	-0.239497	3.552945	3.683097
H	0.441685	3.866272	4.467698
C	-1.483082	3.030601	4.027157
H	-1.760116	2.944320	5.074660
C	-2.362655	2.609461	3.043738
H	-3.324406	2.189396	3.324463
C	-2.041202	2.745105	1.690076
C	1.523597	4.147334	1.944412
H	1.419992	4.736880	1.025027
C	2.204292	5.029344	2.991304
H	1.564294	5.862520	3.301515
H	3.128685	5.445801	2.578310
H	2.479253	4.458420	3.885496
C	2.400484	2.930874	1.607861
H	2.534505	2.304999	2.497048
H	3.387767	3.248254	1.253620
H	1.937463	2.314061	0.832805
C	-3.052321	2.369820	0.626580
H	-2.575579	2.472787	-0.353623
C	-3.484988	0.909552	0.778970

H	-2.612503	0.254141	0.852895
H	-4.088716	0.600715	-0.082185
H	-4.087709	0.760550	1.681988
C	-4.262135	3.313876	0.652918
H	-4.782612	3.257789	1.615770
H	-4.973460	3.038439	-0.133627
H	-3.961270	4.353644	0.488791
N	0.156881	-0.603234	0.700153
N	-0.008332	-2.451383	2.166456
N	1.500502	-2.653251	0.601718
N	0.034896	1.406160	-1.034760
N	-0.080890	3.477981	-2.180994
N	-0.495703	3.581874	0.003515

Calculated energies and coordinates of **G**

Electronic energy ... -2636.04975398 Eh
Total Enthalpy ... -2634.71232895 Eh
Final Gibbs free energy ... -2634.87465738 Eh

CARTESIAN COORDINATES (ANGSTROEM)

B	0.119848	-0.239575	-0.310331
B	-0.416592	0.625595	-1.575329
C	0.751527	-2.308268	0.918790
C	1.187951	-4.282392	1.998547
C	1.831304	-4.328467	0.807419
C	1.121605	-5.250517	3.121238
H	1.475490	-4.797928	4.055434
H	1.743082	-6.122151	2.907098
H	0.095890	-5.596028	3.301432
C	2.714000	-5.362429	0.210060
H	2.682140	-6.275214	0.808776
H	3.757301	-5.027327	0.154948
H	2.404189	-5.609943	-0.811767
C	-0.287270	-2.597643	3.148110
C	-1.619764	-3.037615	3.221590
C	-2.389957	-2.601778	4.299292
H	-3.423058	-2.922518	4.389594
C	-1.854041	-1.747586	5.256118
H	-2.470286	-1.412156	6.085743
C	-0.539977	-1.314824	5.153286
H	-0.136961	-0.642360	5.905827
C	0.275143	-1.735288	4.099234
C	-2.219724	-3.885560	2.115034
H	-1.408261	-4.454293	1.643759
C	-3.260525	-4.891461	2.612928
H	-4.174640	-4.394878	2.955769
H	-2.874170	-5.499307	3.438152
H	-3.546244	-5.562968	1.796636
C	-2.819003	-2.967172	1.036925
H	-3.201652	-3.556277	0.195061
H	-2.073835	-2.260215	0.660753
H	-3.649072	-2.385019	1.454739
C	1.708454	-1.249824	4.006570
H	2.181289	-1.742937	3.149717
C	1.759087	0.262464	3.750695
H	1.223937	0.516004	2.832282
H	2.796681	0.593631	3.632828
H	1.317989	0.819458	4.586011
C	2.510323	-1.626625	5.259891
H	2.126483	-1.114751	6.149135
H	3.559246	-1.335792	5.137035
H	2.472900	-2.704418	5.451197
C	2.096142	-2.756126	-1.131548
C	1.292434	-2.855154	-2.280913
C	1.890129	-2.561758	-3.506708
H	1.306265	-2.621270	-4.418703
C	3.224974	-2.182971	-3.578962
H	3.669969	-1.967755	-4.546201
C	3.984688	-2.052321	-2.425871
H	5.016862	-1.724253	-2.498653
C	3.433293	-2.331656	-1.174062
C	-0.166770	-3.271417	-2.187766
H	-0.589092	-2.761172	-1.312583
C	-0.310399	-4.786376	-1.967256
H	0.161129	-5.341364	-2.786945
H	-1.371000	-5.061027	-1.938647
H	0.142075	-5.106488	-1.024761
C	-0.993483	-2.838807	-3.399947
H	-0.889754	-1.767172	-3.600358
H	-2.051801	-3.042659	-3.209039
H	-0.714516	-3.396421	-4.301657
C	4.229081	-2.100650	0.097145
H	3.799887	-2.726669	0.889123
C	5.707167	-2.480675	-0.037639
H	5.834007	-3.494973	-0.432020
H	6.192918	-2.430458	0.942458
H	6.244735	-1.791536	-0.697594
C	4.077788	-0.635182	0.536938
H	4.486184	0.040732	-0.222633
H	4.617375	-0.459310	1.475249
H	3.031436	-0.356619	0.685829
C	0.829315	2.290652	0.156138
C	2.165290	3.545020	1.275734
C	0.986224	4.243945	1.195688
C	3.446270	3.943866	1.925157
H	3.802997	3.146098	2.585673
H	3.330546	4.857147	2.515091
H	4.228830	4.121840	1.178093
C	0.571703	5.568837	1.728128
H	-0.461460	5.548892	2.093577
H	0.634174	6.360505	0.970975
H	1.220457	5.855077	2.559726
C	-0.995234	1.088921	-2.940506
C	-2.387764	1.224147	-3.132547
C	-2.871125	1.590801	-4.387613
H	-3.938241	1.710653	-4.546575
C	-1.998501	1.816080	-5.446240
H	-2.389095	2.103474	-6.418857
C	-0.628970	1.678864	-5.262270
H	0.040129	1.862019	-6.097517
C	-0.111734	1.317817	-4.017330
C	-3.332929	0.945132	-1.974403
H	-2.863305	1.355114	-1.067240
C	-3.501475	-0.569769	-1.774937
H	-4.137970	-0.775900	-0.906165
H	-2.534236	-1.055005	-1.615193
H	-3.970326	-1.015224	-2.660517
C	-4.703669	1.608628	-2.110083
H	-5.301428	1.136908	-2.898857
H	-4.614100	2.676717	-2.330459
H	-5.257061	1.503223	-1.171034
C	1.388886	1.237322	-3.788591
H	1.567582	0.468914	-3.019509
C	1.920680	2.576198	-3.250488
H	1.793914	3.361294	-4.005888
H	2.985572	2.498916	-3.003944
H	1.390783	2.888065	-2.346525
C	2.182236	0.827693	-5.031714
H	1.780308	-0.085169	-5.483510
H	3.226827	0.645158	-4.759652
H	2.176719	1.617800	-5.790872
C	-1.285041	3.643923	0.320424
C	-2.139817	2.962519	1.206110
C	-3.507159	3.230064	1.126034
H	-4.188587	2.720307	1.801336
C	-4.006374	4.137464	0.202673
H	-5.074464	4.331231	0.155127
C	-3.145686	4.787594	-0.669345
H	-3.548206	5.486975	-1.396731
C	-1.768879	4.560086	-0.627735
C	-1.628620	1.980085	2.242430

H	-0.573591	1.774895	2.040677
C	-1.710204	2.576969	3.654283
H	-1.111581	3.490981	3.732175
H	-1.332419	1.853409	4.385754
H	-2.745367	2.822112	3.919118
C	-2.367441	0.639108	2.169934
H	-3.402830	0.726224	2.520567
H	-1.858833	-0.100648	2.795189
H	-2.381072	0.253165	1.146051
C	-0.862137	5.290604	-1.599928
H	0.176234	5.049952	-1.343186
C	-1.113837	4.818856	-3.038991
H	-0.976850	3.739327	-3.137657
H	-0.424062	5.319238	-3.728024
H	-2.136522	5.054878	-3.354998
C	-1.034876	6.813936	-1.500125
H	-2.017653	7.126450	-1.869687
H	-0.278386	7.319026	-2.110474
H	-0.939926	7.167216	-0.468870
N	0.366578	-1.097403	0.739946
N	0.518109	-3.051430	2.061721
N	1.543758	-3.126469	0.131267
N	0.265518	1.192069	-0.476446
N	2.053650	2.328832	0.633658
N	0.125823	3.437263	0.444123

Calculated energies and coordinates of **TS[G-H]**

Electronic energy ... -2636.00974651 Eh
Total Enthalpy ... -2634.67463800 Eh
Final Gibbs free energy ... -2634.83893577 Eh

CARTESIAN COORDINATES (ANGSTROEM)

B	-0.433415	-0.592822	-0.318938
B	-1.461432	0.429833	-1.020293
C	1.136814	-2.233434	0.652534
C	1.405924	-4.282316	1.603176
C	2.625157	-3.865917	1.170959
C	0.986499	-5.525730	2.298730
H	0.736889	-5.344605	3.351302
H	1.791228	-6.263125	2.265645
H	0.098348	-5.962214	1.828935
C	3.961966	-4.507016	1.229694
H	3.878634	-5.524212	1.617002
H	4.646837	-3.948139	1.878964
H	4.422424	-4.547811	0.236275
C	-0.915440	-3.301759	1.558293
C	-1.815580	-3.568697	0.513259
C	-3.172422	-3.607808	0.836279
H	-3.904833	-3.791295	0.058310
C	-3.604511	-3.395368	2.138516
H	-4.666661	-3.426628	2.363888
C	-2.693954	-3.120836	3.148548
H	-3.052601	-2.935951	4.155930
C	-1.326606	-3.061346	2.877935
C	-1.332832	-3.820954	-0.905748
H	-0.513450	-3.118565	-1.104625
C	-0.777863	-5.246316	-1.062606
H	-1.543812	-5.987852	-0.807520
H	0.095323	-5.417224	-0.426507
H	-0.471733	-5.416738	-2.100843
C	-2.404816	-3.552081	-1.962374
H	-1.949031	-3.593512	-2.956486
H	-2.855223	-2.561714	-1.837506
H	-3.198668	-4.307450	-1.937291
C	-0.333312	-2.654776	3.950542
H	0.643041	-3.086294	3.700264
C	-0.167796	-1.127795	3.934962
H	0.149826	-0.772901	2.950569
H	0.577628	-0.813475	4.674739
H	-1.118567	-0.637437	4.173953
C	-0.708282	-3.150785	5.349663
H	-1.589947	-2.633045	5.741603
H	0.114731	-2.953950	6.044314
H	-0.914898	-4.226425	5.357197
C	3.477773	-1.855778	-0.069712
C	3.600813	-1.972649	-1.459346
C	4.667653	-1.308685	-2.067524
H	4.791118	-1.370313	-3.144782
C	5.563885	-0.563833	-1.317481
H	6.390578	-0.058408	-1.808821
C	5.389960	-0.427652	0.054261
H	6.077470	0.192784	0.620854
C	4.333354	-1.060227	0.706256
C	2.596180	-2.728350	-2.306046
H	1.883311	-3.224681	-1.636661
C	3.256822	-3.822900	-3.153206
H	3.954035	-3.397679	-3.882877
H	2.495032	-4.379747	-3.709774
H	3.812356	-4.531910	-2.529820
C	1.806141	-1.742204	-3.183059
H	1.405082	-0.916820	-2.587107
H	0.980748	-2.259517	-3.687264
H	2.452276	-1.307748	-3.954366
C	4.051665	-0.799666	2.172979
H	3.446292	-1.629280	2.561016
C	5.315546	-0.711146	3.032757
H	5.960236	-1.586142	2.896564
H	5.041448	-0.647400	4.090993
H	5.903587	0.182522	2.798991
C	3.211104	0.485286	2.293519
H	3.783124	1.347972	1.932144
H	2.934495	0.666233	3.339054
H	2.301409	0.420822	1.688399
C	0.649120	2.060864	-0.861058
C	2.660638	2.827161	-1.099494
C	1.956303	3.848725	-0.504329
C	4.098494	2.807418	-1.492276
H	4.538960	3.808467	-1.467867
H	4.208635	2.403364	-2.504688
H	4.681382	2.159633	-0.826537
C	2.347220	5.202677	-0.030458
H	1.628695	5.968694	-0.346451
H	3.327503	5.469309	-0.432989
H	2.411070	5.262136	1.065383
C	-2.907598	0.571842	-1.624951
C	-3.954904	1.131901	-0.863205
C	-5.216595	1.280323	-1.441432
H	-6.031948	1.715120	-0.871327
C	-5.444697	0.888543	-2.755981
H	-6.430552	1.017262	-3.195549
C	-4.416503	0.336883	-3.509349
H	-4.609654	0.038069	-4.535529
C	-3.145568	0.171970	-2.954654
C	-3.696415	1.531826	0.582936
H	-2.706128	2.009027	0.613415
C	-3.645646	0.283070	1.478437
H	-3.411224	0.556790	2.514556
H	-2.889599	-0.431922	1.136439
H	-4.615227	-0.229578	1.468336
C	-4.701649	2.538153	1.143597
H	-5.691879	2.087776	1.282820
H	-4.803250	3.408438	0.487566
H	-4.361085	2.896025	2.121068
C	-1.992217	-0.373006	-3.783719
H	-1.345108	-0.939390	-3.094692
C	-1.144972	0.772000	-4.360801
H	-1.748356	1.379737	-5.045692
H	-0.286429	0.373986	-4.915159
H	-0.765115	1.421187	-3.567241
C	-2.424077	-1.321548	-4.904962
H	-3.116718	-2.089115	-4.544639

H	-1.547090	-1.820054	-5.333163
H	-2.914722	-0.778733	-5.720851
C	-0.465789	4.078798	0.131747
C	-0.618831	4.223740	1.519662
C	-1.710696	4.954437	1.989391
H	-1.854936	5.083058	3.058210
C	-2.622053	5.514730	1.104664
H	-3.468428	6.080322	1.485106
C	-2.465415	5.341532	-0.263680
H	-3.192605	5.776983	-0.942735
C	-1.387740	4.619936	-0.779724
C	0.320964	3.527457	2.483972
H	1.254930	3.307876	1.953604
C	0.666690	4.373877	3.712747
H	1.026799	5.367914	3.427250
H	1.450215	3.879078	4.297031
H	-0.197238	4.504214	4.373606
C	-0.288941	2.182104	2.901547
H	-1.245651	2.336390	3.415433
H	0.386403	1.651963	3.581823
H	-0.471443	1.546818	2.027969
C	-1.203822	4.478631	-2.278300
H	-0.506150	3.653841	-2.455002
C	-2.510974	4.138866	-3.002191
H	-2.988910	3.255143	-2.567229
H	-2.304709	3.928113	-4.056968
H	-3.223619	4.970830	-2.970834
C	-0.570011	5.753749	-2.854764
H	-1.218285	6.621459	-2.683598
H	-0.415719	5.648967	-3.934565
H	0.400555	5.956192	-2.390463
N	0.667476	-1.109503	0.203123
N	0.487477	-3.272797	1.273045
N	2.451107	-2.602447	0.597470
N	-0.473597	1.360452	-0.907369
N	1.862870	1.723501	-1.303151
N	0.659905	3.362347	-0.363520

Calculated energies and coordinates of **H**

Electronic energy ... -2636.02383915 Eh
Total Enthalpy ... -2634.68744182 Eh
Final Gibbs free energy ... -2634.85294402 Eh

CARTESIAN COORDINATES (ANGSTROEM)

B	-0.212461	-0.401464	-0.508523
B	-1.391773	0.678664	-0.988065
C	1.148526	-2.280204	0.560731
C	1.392568	-4.284361	1.584872
C	2.634906	-3.835952	1.259512
C	0.934604	-5.529034	2.249974
H	0.424584	-5.314573	3.196631
H	1.784022	-6.181382	2.459682
H	0.225599	-6.074522	1.617190
C	3.978623	-4.432037	1.460376
H	3.885924	-5.447506	1.849864
H	4.578250	-3.847076	2.168007
H	4.537639	-4.470940	0.518855
C	-0.946442	-3.398532	1.244449
C	-1.656719	-3.879966	0.137435
C	-3.050044	-3.866204	0.213567
H	-3.638327	-4.186797	-0.641052
C	-3.693574	-3.434908	1.362799
H	-4.778883	-3.419199	1.397034
C	-2.960644	-3.018947	2.467518
H	-3.480808	-2.699975	3.364801
C	-1.568341	-2.978456	2.429188
C	-0.967635	-4.416381	-1.101527
H	0.115382	-4.410400	-0.926866
C	-1.375457	-5.872355	-1.371694
H	-2.437920	-5.945530	-1.626060
H	-1.196329	-6.509252	-0.499063
H	-0.802605	-6.273437	-2.214702
C	-1.256810	-3.536520	-2.321162
H	-0.736722	-3.923756	-3.204771
H	-0.937461	-2.503949	-2.152695
H	-2.330026	-3.511395	-2.536580
C	-0.767402	-2.444259	3.601050
H	0.251230	-2.847782	3.532074
C	-0.659135	-0.915103	3.509574
H	-0.193582	-0.602650	2.570421
H	-0.061877	-0.521482	4.339854
H	-1.653055	-0.454725	3.552957
C	-1.337734	-2.869839	4.957569
H	-2.292593	-2.377079	5.167966
H	-0.645686	-2.584801	5.756642
H	-1.496590	-3.952274	5.008607
C	3.555778	-1.852257	0.049526
C	3.799252	-1.988630	-1.322630
C	4.962320	-1.401463	-1.826927
H	5.184555	-1.484327	-2.886771
C	5.831814	-0.712533	-0.996064
H	6.736538	-0.272666	-1.405894
C	5.529829	-0.538817	0.349819
H	6.194531	0.049154	0.974255
C	4.375155	-1.092823	0.899497
C	2.837652	-2.691082	-2.259875
H	2.018827	-3.111839	-1.666239
C	3.501663	-3.854125	-3.006495
H	4.305063	-3.502430	-3.662431
H	2.764449	-4.367418	-3.633397
H	3.929324	-4.585297	-2.311680
C	2.222036	-1.672033	-3.233573
H	1.794708	-0.817472	-2.698389
H	1.432809	-2.147443	-3.826958
H	2.979958	-1.289441	-3.926591
C	3.956058	-0.789628	2.324815
H	3.326414	-1.614098	2.684646
C	5.130890	-0.648103	3.295699
H	5.807965	-1.507710	3.244546
H	4.756429	-0.567148	4.321156
H	5.714478	0.256426	3.095357
C	3.091703	0.486398	2.322247
H	3.677363	1.342184	1.966545
H	2.735627	0.708222	3.334806
H	2.226781	0.387664	1.658657
C	0.231314	2.427812	-1.017238
C	2.384321	2.660042	-1.212767
C	1.937213	3.850476	-0.687441
C	3.772720	2.270575	-1.585613
H	4.491678	3.065085	-1.365384
H	3.840064	2.032172	-2.654375
H	4.077787	1.371979	-1.038765
C	2.622369	5.122489	-0.337762
H	3.688418	5.046693	-0.564879
H	2.522336	5.373172	0.727074
H	2.215277	5.971530	-0.902430
C	-2.855103	0.130108	-1.293422
C	-3.863237	0.126503	-0.306564
C	-5.103789	-0.452233	-0.585934
H	-5.884496	-0.463003	0.169559
C	-5.356768	-1.019650	-1.829132
H	-6.323391	-1.474475	-2.032409
C	-4.385370	-0.979204	-2.823114
H	-4.609172	-1.394935	-3.801658
C	-3.140175	-0.400141	-2.571041
C	-3.607893	0.823296	1.021643
H	-2.558059	0.622947	1.290413
C	-4.487266	0.334989	2.174927
H	-4.134334	0.764922	3.119481
H	-4.473036	-0.755319	2.263970
H	-5.528114	0.655005	2.048171

C	-3.762067	2.346080	0.861277
H	-4.779882	2.583083	0.528143
H	-3.050504	2.735336	0.128409
H	-3.585760	2.856467	1.816538
C	-2.114700	-0.228990	-3.682999
H	-1.115221	-0.368484	-3.237479
C	-2.168372	1.213849	-4.213335
H	-3.162238	1.423464	-4.627152
H	-1.424072	1.361998	-5.005144
H	-1.971037	1.928455	-3.410058
C	-2.254570	-1.211220	-4.848200
H	-2.302379	-2.251723	-4.513286
H	-1.398942	-1.109195	-5.524743
H	-3.155712	-1.003451	-5.436793
C	-0.341714	4.597988	0.071625
C	-0.320378	4.671717	1.475508
C	-1.197138	5.556621	2.101848
H	-1.204208	5.632822	3.185289
C	-2.077655	6.332105	1.357550
H	-2.757834	7.013990	1.860701
C	-2.097667	6.228393	-0.025553
H	-2.799815	6.829742	-0.596829
C	-1.232572	5.361329	-0.696102
C	0.553527	3.740012	2.293529
H	1.382562	3.399425	1.661331
C	1.156856	4.401647	3.535976
H	1.669635	5.336645	3.286375
H	1.883138	3.725602	4.001077
H	0.394441	4.625830	4.290171
C	-0.256701	2.493821	2.680605
H	-1.115953	2.768416	3.304809
H	0.368654	1.793795	3.245842
H	-0.637012	1.984566	1.788615
C	-1.284788	5.258904	-2.206701
H	-0.562260	4.497335	-2.517298
C	-2.671419	4.798779	-2.676244
H	-2.945581	3.861045	-2.184496
H	-2.669048	4.633754	-3.759694
H	-3.437891	5.550239	-2.452908
C	-0.887324	6.586881	-2.866030
H	-1.593995	7.384511	-2.609527
H	-0.880312	6.483307	-3.956921
H	0.109837	6.907065	-2.545314
N	0.638007	-1.221444	0.013453
N	0.482045	-3.315332	1.156279
N	2.473561	-2.592530	0.635639
N	-1.048950	2.016817	-1.040963
N	1.335853	1.790343	-1.421201
N	0.556576	3.703909	-0.579507

Calculated energies and coordinates of **TS[H-4]**

Electronic energy ... -2636.02358368 Eh
Total Enthalpy ... -2634.68808554 Eh
Final Gibbs free energy ... -2634.85151642 Eh

CARTESIAN COORDINATES (ANGSTROEM)

B	-0.173346	-0.307644	-0.542435
B	-1.515553	0.668793	-0.860644
C	1.169164	-2.232599	0.476328
C	1.391555	-4.227878	1.535825
C	2.640547	-3.789313	1.225661
C	0.918292	-5.466510	2.202052
H	0.395106	-5.246322	3.140098
H	1.761902	-6.121323	2.427343
H	0.216543	-6.012481	1.561492
C	3.978582	-4.385926	1.460375
H	3.876126	-5.386293	1.884855
H	4.573797	-3.778924	2.152974
H	4.547947	-4.461329	0.527139
C	-0.933704	-3.354875	1.160566
C	-1.630807	-3.873600	0.062889
C	-3.024032	-3.892758	0.139403
H	-3.605469	-4.237586	-0.710406
C	-3.676395	-3.467682	1.285827
H	-4.761630	-3.480675	1.321589
C	-2.953139	-3.025557	2.387099
H	-3.479285	-2.721373	3.285685
C	-1.563351	-2.943396	2.344329
C	-0.923208	-4.421274	-1.161231
H	0.155827	-4.429963	-0.962605
C	-1.343184	-5.871646	-1.441554
H	-2.397930	-5.932240	-1.728723
H	-1.198315	-6.509551	-0.563299
H	-0.750522	-6.281340	-2.266562
C	-1.173562	-3.537372	-2.386025
H	-0.655307	-3.940990	-3.263592
H	-0.826331	-2.514315	-2.215505
H	-2.244110	-3.485162	-2.609639
C	-0.769908	-2.393541	3.514680
H	0.249979	-2.795219	3.453484
C	-0.661778	-0.864926	3.415301
H	-0.204720	-0.554656	2.470910
H	-0.056171	-0.470110	4.239079
H	-1.654362	-0.402910	3.468187
C	-1.343700	-2.808684	4.872894
H	-2.299878	-2.315432	5.076449
H	-0.654404	-2.515874	5.671556
H	-1.501134	-3.890892	4.932828
C	3.596257	-1.829311	0.022414
C	3.906548	-2.014986	-1.330380
C	5.094259	-1.447248	-1.798130
H	5.369181	-1.568527	-2.841870
C	5.922787	-0.727662	-0.950180
H	6.847968	-0.304068	-1.330356
C	5.555965	-0.506405	0.372366
H	6.190714	0.102307	1.008149
C	4.375712	-1.042702	0.884994
C	2.981211	-2.744800	-2.283486
H	2.156915	-3.175418	-1.704062
C	3.681732	-3.898671	-3.009933
H	4.483881	-3.535872	-3.661275
H	2.964132	-4.435978	-3.639219
H	4.118381	-4.611860	-2.302400
C	2.367318	-1.743583	-3.276124
H	1.879866	-0.914913	-2.751941
H	1.623522	-2.244832	-3.905549
H	3.138565	-1.324506	-3.932474
C	3.890189	-0.698480	2.279567
H	3.222671	-1.500882	2.619748
C	5.016213	-0.565108	3.307911
H	5.670259	-1.443760	3.312127
H	4.591153	-0.448790	4.309986
H	5.634392	0.318139	3.116961
C	3.058932	0.597666	2.206648
H	3.694732	1.438212	1.904124
H	2.625297	0.829886	3.186187
H	2.249558	0.520576	1.473314
C	0.039024	2.390933	-0.903744
C	2.205401	2.411829	-1.171501
C	1.883201	3.650776	-0.675242
C	3.531141	1.887137	-1.596461
H	4.343158	2.575542	-1.344847
H	3.555999	1.706023	-2.678688
H	3.734340	0.929220	-1.108588
C	2.686835	4.872666	-0.410252
H	3.733606	4.693612	-0.667233
H	2.645389	5.179723	0.643700
H	2.333494	5.726817	-1.002680
C	-2.943631	0.049050	-1.181962
C	-3.975001	-0.010541	-0.221503
C	-5.182439	-0.637834	-0.539440

H	-5.979611	-0.693208	0.196677
C	-5.382318	-1.196585	-1.795887
H	-6.322732	-1.690888	-2.027880
C	-4.393589	-1.091978	-2.767869
H	-4.578412	-1.494980	-3.759814
C	-3.182187	-0.461473	-2.478299
C	-3.787036	0.684399	1.118455
H	-2.735977	0.530628	1.411593
C	-4.679116	0.150671	2.241904
H	-4.364267	0.574459	3.202482
H	-4.638013	-0.939330	2.314286
H	-5.724872	0.443183	2.091395
C	-4.011892	2.199159	0.966343
H	-5.034409	2.387457	0.616658
H	-3.307073	2.628481	0.250265
H	-3.880614	2.706371	1.930586
C	-2.154182	-0.200150	-3.571247
H	-1.153066	-0.306401	-3.119296
C	-2.275899	1.257670	-4.048672
H	-3.270253	1.426989	-4.479363
H	-1.523089	1.476543	-4.815677
H	-2.138530	1.951340	-3.215298
C	-2.231567	-1.139547	-4.776851
H	-2.228206	-2.193986	-4.486853
H	-1.375335	-0.964094	-5.437191
H	-3.136414	-0.952619	-5.366620
C	-0.287299	4.654712	0.101227
C	-0.218811	4.800311	1.498098
C	-0.962111	5.820410	2.089555
H	-0.928828	5.957772	3.166188
C	-1.760366	6.657512	1.318806
H	-2.336586	7.445846	1.795484
C	-1.831790	6.481220	-0.054822
H	-2.470555	7.132281	-0.645531
C	-1.096145	5.479270	-0.692240
C	0.556625	3.806927	2.343119
H	1.358254	3.384791	1.724650
C	1.205627	4.429462	3.582139
H	1.806889	5.308170	3.325043
H	1.860934	3.696237	4.065592
H	0.459490	4.734002	4.324333
C	-0.372198	2.648545	2.739954
H	-1.194518	3.017292	3.365135
H	0.182539	1.894556	3.309698
H	-0.808088	2.172066	1.855444
C	-1.205932	5.298241	-2.192746
H	-0.540564	4.478424	-2.483602
C	-2.634899	4.894549	-2.581969
H	-2.935412	3.996879	-2.033964
H	-2.691225	4.681043	-3.655520
H	-3.346994	5.697917	-2.359266
C	-0.758954	6.558241	-2.945743
H	-1.416001	7.408156	-2.728853
H	-0.787574	6.383123	-4.026996
H	0.261953	6.844876	-2.671471
N	0.690683	-1.174717	-0.097644
N	0.493059	-3.260767	1.079207
N	2.493157	-2.556972	0.580588
N	-1.256897	2.033991	-0.853629
N	1.067923	1.645330	-1.329698
N	0.494312	3.636169	-0.517744

Calculated energies and coordinates of **4**

Electronic energy ... -2636.05974117 Eh
Total Enthalpy ... -2634.72160570 Eh
Final Gibbs free energy ... -2634.88287410 Eh

CARTESIAN COORDINATES (ANGSTROEM)

B	0.320185	0.264103	-0.314293
B	-1.350554	0.434597	-0.954774
C	1.642113	-1.773966	0.405559
C	1.791987	-3.513877	1.905558
C	2.769559	-3.690410	0.988322
C	1.437069	-4.306635	3.112197
H	2.234061	-5.018243	3.338970
H	0.507957	-4.874795	2.980578
H	1.293096	-3.661383	3.985342
C	3.801133	-4.751578	0.863410
H	3.567140	-5.455664	0.056751
H	3.867812	-5.320460	1.793435
H	4.787646	-4.328511	0.644556
C	-0.110256	-1.905361	2.181101
C	-1.284161	-2.644758	1.958700
C	-2.447366	-2.216746	2.599838
H	-3.374166	-2.760379	2.452573
C	-2.439393	-1.090399	3.409388
H	-3.359988	-0.763280	3.884852
C	-1.264934	-0.378068	3.617112
H	-1.282895	0.499438	4.254337
C	-0.069870	-0.777818	3.019575
C	-1.299579	-3.835195	1.014715
H	-0.310967	-4.306144	1.044360
C	-2.325414	-4.903212	1.404561
H	-3.351912	-4.560312	1.235292
H	-2.232631	-5.195002	2.456582
H	-2.180316	-5.795220	0.786443
C	-1.525950	-3.375062	-0.431184
H	-1.454218	-4.227059	-1.116309
H	-0.794836	-2.621634	-0.737718
H	-2.515030	-2.925730	-0.552483
C	1.235189	-0.041408	3.270980
H	1.728926	0.060449	2.297110
C	1.038212	1.363455	3.840215
H	0.348726	1.951741	3.229245
H	1.997600	1.891226	3.858789
H	0.662099	1.332938	4.869571
C	2.177772	-0.839889	4.187504
H	1.688313	-1.066818	5.141858
H	3.077475	-0.251329	4.400653
H	2.499102	-1.776660	3.726176
C	3.499462	-2.535308	-1.104130
C	3.241674	-3.407541	-2.172774
C	4.086750	-3.346290	-3.283498
H	3.907334	-4.002681	-4.130040
C	5.146140	-2.454371	-3.322988
H	5.793708	-2.419800	-4.194631
C	5.381616	-1.597483	-2.253839
H	6.212765	-0.902808	-2.308045
C	4.567513	-1.619719	-1.122500
C	2.060113	-4.360998	-2.180607
H	1.646918	-4.413462	-1.166631
C	2.452580	-5.782957	-2.606768
H	2.707935	-5.825498	-3.670972
H	1.610890	-6.465170	-2.447291
H	3.312977	-6.159771	-2.044510
C	0.962441	-3.818697	-3.104570
H	0.623299	-2.830120	-2.783534
H	0.096241	-4.489004	-3.116571
H	1.341062	-3.732741	-4.128999
C	4.824977	-0.701794	0.060131
H	3.844518	-0.341265	0.393413
C	5.478564	-1.449615	1.234535
H	4.830484	-2.234046	1.633986
H	5.692278	-0.750026	2.050791
H	6.425981	-1.904313	0.921839
C	5.673009	0.520235	-0.299168
H	6.713341	0.243663	-0.505858
H	5.686180	1.218802	0.543774
H	5.279024	1.048277	-1.173516
C	-0.746985	2.308273	0.039292
C	1.404266	2.601396	0.685002

C	0.733093	3.747938	0.962190
C	2.851734	2.301987	0.806833
H	3.053409	1.498869	1.525006
H	3.403792	3.189608	1.125151
H	3.251454	1.974201	-0.158480
C	1.166437	5.037523	1.551362
H	0.974365	5.878564	0.873440
H	2.235232	5.012338	1.773060
H	0.631174	5.251788	2.485823
C	-2.235032	-0.388153	-1.966750
C	-3.562243	-0.759409	-1.651749
C	-4.330708	-1.458989	-2.584976
H	-5.347815	-1.752324	-2.340082
C	-3.813544	-1.785672	-3.829472
H	-4.420775	-2.331440	-4.547246
C	-2.519990	-1.402129	-4.162838
H	-2.137120	-1.647279	-5.148179
C	-1.724129	-0.707065	-3.251541
C	-4.188680	-0.382258	-0.315978
H	-3.373635	-0.214071	0.401219
C	-5.101993	-1.469255	0.266581
H	-5.362980	-1.218430	1.301185
H	-4.622962	-2.454152	0.262970
H	-6.041490	-1.552067	-0.290950
C	-4.974214	0.931214	-0.454429
H	-5.779023	0.810232	-1.190067
H	-4.313078	1.738526	-0.777573
H	-5.427533	1.212294	0.504557
C	-0.338946	-0.216946	-3.659613
H	0.353469	-0.483387	-2.845200
C	-0.319728	1.314810	-3.796378
H	-1.012866	1.628090	-4.586340
H	0.686164	1.666329	-4.056599
H	-0.630441	1.811208	-2.873273
C	0.199830	-0.830089	-4.954204
H	0.127094	-1.920367	-4.959993
H	1.254365	-0.561183	-5.079399
H	-0.343224	-0.449422	-5.827562
C	-1.633171	4.559428	0.587634
C	-2.433821	4.699472	1.730430
C	-3.392092	5.713114	1.734941
H	-4.031303	5.843956	2.602777
C	-3.545315	6.551946	0.638805
H	-4.297040	7.336100	0.658768
C	-2.750300	6.385133	-0.485970
H	-2.891590	7.036788	-1.343344
C	-1.776388	5.385866	-0.535537
C	-2.327306	3.726701	2.886257
H	-1.334685	3.264033	2.842158
C	-2.465718	4.393644	4.257724
H	-1.782789	5.243250	4.364911
H	-2.237530	3.668841	5.046931
H	-3.485030	4.753030	4.435186
C	-3.369165	2.610921	2.704641
H	-4.379313	3.037593	2.696756
H	-3.308499	1.890582	3.528015
H	-3.214737	2.079293	1.759694
C	-0.960874	5.180444	-1.796537
H	-0.160010	4.463851	-1.578470
C	-1.849279	4.566869	-2.890133
H	-2.303870	3.635229	-2.538979
H	-1.257310	4.350846	-3.786476
H	-2.650921	5.260758	-3.169131
C	-0.299920	6.475771	-2.283422
H	-1.044571	7.211687	-2.604641
H	0.346087	6.266107	-3.142594
H	0.310594	6.935527	-1.498841
N	1.339810	-0.659933	-0.148611
N	1.092925	-2.344806	1.552525
N	2.685506	-2.628556	0.066991
N	-1.793272	1.722423	-0.507467
N	0.494147	1.696713	0.118286
N	-0.617931	3.551013	0.573466

Calculated energies and coordinates of **TS[1$_{INT}$-I]**

Electronic energy ... -2868.14016191 Eh
Total Enthalpy ... -2866.69817497 Eh
Final Gibbs free energy ... -2866.87363367 Eh

CARTESIAN COORDINATES (ANGSTROEM)

B	-0.708711	-0.483800	-0.135496
B	0.759634	-0.336578	-0.649256
C	-3.190685	-0.231050	0.467815
C	-5.451730	-0.324421	0.817772
C	-4.988149	0.748530	1.499678
C	-6.826483	-0.852727	0.636606
H	-6.920207	-1.877153	1.018446
H	-7.105686	-0.880674	-0.423748
H	-7.545829	-0.225354	1.166365
C	-5.694140	1.768683	2.314405
H	-5.378938	1.748343	3.364868
H	-6.771974	1.598540	2.278357
H	-5.490399	2.779834	1.941682
C	-4.484984	-1.935576	-0.819993
C	-4.540444	-3.284113	-0.434313
C	-4.745181	-4.235658	-1.433350
H	-4.801097	-5.288243	-1.173073
C	-4.873189	-3.853792	-2.763479
H	-5.024658	-4.609672	-3.529295
C	-4.813615	-2.513424	-3.119070
H	-4.923530	-2.230697	-4.162438
C	-4.630969	-1.523195	-2.152445
C	-4.342843	-3.684062	1.015746
H	-4.652784	-2.836344	1.639488
C	-5.182205	-4.896312	1.428358
H	-4.825795	-5.816793	0.953408
H	-6.238152	-4.766453	1.167448
H	-5.111579	-5.045436	2.510868
C	-2.852020	-3.934086	1.293534
H	-2.694727	-4.160021	2.354601
H	-2.240390	-3.068027	1.022455
H	-2.497996	-4.788476	0.704687
C	-4.613687	-0.056848	-2.541982
H	-4.695008	0.537881	-1.623991
C	-3.292878	0.321965	-3.228246
H	-2.431932	0.121364	-2.583668
H	-3.297868	1.387310	-3.487953
H	-3.162138	-0.251371	-4.153643
C	-5.811955	0.315811	-3.425452
H	-5.750024	-0.156082	-4.411836
H	-5.836443	1.399636	-3.582160
H	-6.759353	0.013657	-2.966603
C	-2.760720	1.799435	1.887082
C	-2.511245	1.711004	3.270014
C	-1.849905	2.775558	3.879202
H	-1.656356	2.752281	4.947070
C	-1.414207	3.863259	3.129942
H	-0.903042	4.686318	3.621982
C	-1.604037	3.890991	1.756582
H	-1.223711	4.728503	1.179404
C	-2.288376	2.862544	1.105180
C	-2.861464	0.449215	4.037129
H	-3.763571	0.010291	3.592433
C	-3.148507	0.688051	5.521118
H	-2.243774	0.976190	6.067483
H	-3.520369	-0.234057	5.979499
H	-3.899800	1.471429	5.670082
C	-1.724483	-0.574322	3.863758
H	-1.517841	-0.776161	2.807503
H	-1.982192	-1.519473	4.355500
H	-0.801574	-0.191562	4.314312

C	-2.529121	2.936926	-0.389589
H	-2.966943	1.987930	-0.713384
C	-3.528887	4.051278	-0.729042
H	-4.482241	3.903526	-0.209995
H	-3.725808	4.068568	-1.807042
H	-3.136303	5.033216	-0.441179
C	-1.215196	3.113183	-1.159734
H	-0.738312	4.074232	-0.938410
H	-1.404747	3.071733	-2.238350
H	-0.508152	2.315153	-0.912197
C	3.209384	0.406365	-0.153768
C	5.114654	1.514857	0.526069
C	5.489256	0.262983	0.174786
C	5.906995	2.674733	1.006541
H	6.968296	2.420383	1.039148
H	5.601191	2.995130	2.010741
H	5.780001	3.540320	0.344403
C	6.797555	-0.431545	0.285830
H	7.586565	0.283458	0.528952
H	7.069283	-0.943290	-0.644208
H	6.778865	-1.196045	1.073600
C	2.997829	2.835992	0.377066
C	2.535718	3.299277	1.620529
C	1.897997	4.538804	1.654865
H	1.542378	4.935549	2.599894
C	1.711405	5.276706	0.491573
H	1.216794	6.243696	0.539244
C	2.152616	4.787284	-0.729180
H	1.993073	5.372586	-1.630677
C	2.813505	3.559691	-0.810179
C	2.693130	2.444144	2.864506
H	3.633873	1.887386	2.768833
C	1.556147	1.411372	2.942999
H	1.693377	0.767355	3.819992
H	1.514006	0.779117	2.051505
H	0.590585	1.919332	3.037069
C	2.764445	3.258255	4.158370
H	3.518680	4.050965	4.101720
H	3.021156	2.599920	4.994806
H	1.799228	3.719087	4.397765
C	3.296961	3.036537	-2.148855
H	3.943071	2.170919	-1.960853
C	4.136850	4.071929	-2.907178
H	3.537548	4.937088	-3.210977
H	4.551356	3.624331	-3.817160
H	4.968947	4.436023	-2.295256
C	2.108673	2.559419	-2.997183
H	1.527964	1.796466	-2.468163
H	2.462362	2.137162	-3.945568
H	1.439195	3.396742	-3.228687
C	4.307533	-1.785784	-0.646915
C	4.350693	-2.134345	-2.008923
C	4.359682	-3.491726	-2.328341
H	4.381015	-3.802786	-3.367211
C	4.321517	-4.460472	-1.332126
H	4.324521	-5.512069	-1.604678
C	4.254128	-4.093804	0.003773
H	4.197859	-4.863296	0.768123
C	4.240011	-2.747120	0.373791
C	4.374800	-1.062926	-3.089256
H	3.707548	-0.256647	-2.754528
C	5.774069	-0.449753	-3.263314
H	6.101409	0.079774	-2.365204
H	5.765651	0.270649	-4.089426
H	6.509065	-1.228160	-3.499751
C	3.862405	-1.560645	-4.444049
H	4.579298	-2.241094	-4.918121
H	3.730859	-0.708722	-5.119259
H	2.902475	-2.079066	-4.356643
C	4.070463	-2.344987	1.827112
H	4.378702	-1.297411	1.930229
C	2.586176	-2.428597	2.221027
H	1.960103	-1.794255	1.584085
H	2.449907	-2.112398	3.261779
H	2.224922	-3.460093	2.128401
C	4.934339	-3.178420	2.780373
H	5.984805	-3.189960	2.470064
H	4.589387	-4.216430	2.837110
H	4.879513	-2.763417	3.792363
N	-2.008990	-0.474461	0.042433
N	-4.354296	-0.941265	0.194194
N	-3.598930	0.813714	1.292714
N	1.994303	0.137202	-0.378444
N	3.726861	1.614589	0.333952
N	4.343311	-0.409655	-0.289902
C	-1.215780	-3.611547	-2.028762
C	0.814385	-1.680684	-2.330349
C	-0.151964	-3.588286	-1.148445
C	0.882160	-2.654538	-1.293169
C	-1.245415	-2.702733	-3.113453
C	-0.245363	-1.779204	-3.275599
H	-0.095225	-4.313095	-0.340156
H	1.763686	-2.731148	-0.669135
H	-2.061157	-2.755249	-3.828823
H	-0.260236	-1.096511	-4.121550
H	1.706768	-1.142530	-2.623534
H	-2.016443	-4.333993	-1.910801

Calculated energies and coordinates of **I**

Electronic energy ... -2868.15714939 Eh
Total Enthalpy ... -2866.71436422 Eh
Final Gibbs free energy ... -2866.88793882 Eh

CARTESIAN COORDINATES (ANGSTROEM)

B	-0.663011	-0.504965	-0.514960
B	0.775049	-0.626668	-1.253421
C	-3.010310	-0.138590	0.500272
C	-5.248577	-0.302323	0.874893
C	-4.792238	0.741105	1.610368
C	-6.606620	-0.882685	0.727634
H	-6.600545	-1.955187	0.953084
H	-6.988699	-0.771266	-0.293931
H	-7.302075	-0.391246	1.410417
C	-5.502495	1.706650	2.485575
H	-5.166700	1.644330	3.527054
H	-6.576788	1.515157	2.458582
H	-5.327227	2.736716	2.153710
C	-4.247657	-1.914760	-0.746610
C	-3.913947	-3.206053	-0.322479
C	-4.102323	-4.247459	-1.232759
H	-3.847926	-5.263171	-0.943280
C	-4.606119	-4.002472	-2.501228
H	-4.745503	-4.826711	-3.195180
C	-4.926778	-2.708784	-2.895733
H	-5.314728	-2.536767	-3.894726
C	-4.751059	-1.632960	-2.027573
C	-3.370904	-3.488442	1.065089
H	-3.378166	-2.550002	1.632311
C	-4.253710	-4.490279	1.821876
H	-4.246141	-5.473066	1.338360
H	-5.293665	-4.150554	1.873764
H	-3.883383	-4.621663	2.844386
C	-1.916705	-3.974738	0.997236
H	-1.515921	-4.106424	2.008584
H	-1.285828	-3.262656	0.458537
H	-1.849468	-4.937243	0.476987
C	-5.058991	-0.208929	-2.457516
H	-5.293549	0.373662	-1.557729
C	-3.826785	0.439105	-3.109387
H	-2.941651	0.370171	-2.470196
H	-4.027168	1.494567	-3.328359

H	-3.584545	-0.067487	-4.049749
C	-6.271323	-0.112492	-3.389515
H	-6.057760	-0.539026	-4.375202
H	-6.536874	0.938334	-3.544357
H	-7.143960	-0.631230	-2.978032
C	-2.585879	1.824715	2.004573
C	-2.314651	1.687179	3.378017
C	-1.675091	2.748319	4.015037
H	-1.459495	2.690919	5.077022
C	-1.290369	3.877966	3.300260
H	-0.793757	4.696324	3.814244
C	-1.512035	3.956813	1.934541
H	-1.165253	4.824791	1.382155
C	-2.178547	2.934238	1.254641
C	-2.630573	0.393478	4.107775
H	-3.527759	-0.050205	3.657628
C	-2.913193	0.581674	5.599992
H	-2.011157	0.872201	6.148947
H	-3.262529	-0.361469	6.032314
H	-3.679476	1.344143	5.777345
C	-1.478425	-0.606345	3.899558
H	-1.257246	-0.756760	2.837740
H	-1.729241	-1.575983	4.345291
H	-0.565899	-0.234192	4.377812
C	-2.430888	3.052947	-0.234553
H	-3.007098	2.179544	-0.559334
C	-3.265750	4.297258	-0.566908
H	-4.217551	4.300150	-0.024134
H	-3.482297	4.326757	-1.640243
H	-2.728555	5.217005	-0.311759
C	-1.104969	3.053012	-1.008582
H	-0.517607	3.951242	-0.788602
H	-1.299501	3.030398	-2.086820
H	-0.485409	2.186058	-0.758516
C	2.977938	0.413609	-0.391885
C	4.868806	1.406549	0.544829
C	5.104026	0.077541	0.507886
C	5.731149	2.537617	0.973203
H	6.700361	2.167250	1.313936
H	5.281737	3.119894	1.787582
H	5.902445	3.235278	0.143003
C	6.277852	-0.713915	0.958798
H	7.079860	-0.045752	1.280644
H	6.666936	-1.363540	0.165521
H	6.021367	-1.366129	1.803735
C	2.910977	2.866431	0.089386
C	2.475402	3.296357	1.358643
C	1.917070	4.569736	1.461080
H	1.596016	4.940632	2.429167
C	1.764844	5.374464	0.336465
H	1.334482	6.368031	0.435908
C	2.154186	4.910938	-0.911541
H	2.014890	5.541666	-1.785600
C	2.739489	3.650643	-1.058648
C	2.567462	2.362874	2.553168
H	3.505567	1.799042	2.471265
C	1.423252	1.335908	2.507912
H	1.538575	0.606910	3.319406
H	1.398506	0.799558	1.555427
H	0.459051	1.837507	2.633040
C	2.583121	3.084801	3.901827
H	3.351438	3.865105	3.938334
H	2.788887	2.366083	4.702176
H	1.613798	3.545716	4.123476
C	3.152952	3.158704	-2.429937
H	3.686741	2.211700	-2.294186
C	4.103323	4.137180	-3.131194
H	3.609253	5.088679	-3.357692
H	4.448952	3.712284	-4.080178
H	4.980839	4.352616	-2.512104
C	1.910169	2.873671	-3.286178
H	1.269069	2.145813	-2.780840
H	2.203041	2.468314	-4.262077
H	1.336181	3.792440	-3.459716
C	4.004961	-1.923228	-0.416154
C	4.426790	-2.285725	-1.709877
C	4.565062	-3.644021	-1.994635
H	4.887169	-3.957223	-2.982546
C	4.261207	-4.607961	-1.040434
H	4.367416	-5.661447	-1.284395
C	3.796443	-4.230449	0.211411
H	3.530355	-4.994770	0.936408
C	3.665142	-2.881823	0.549551
C	4.661814	-1.227590	-2.776514
H	3.973551	-0.399661	-2.561559
C	6.093454	-0.671458	-2.732284
H	6.299018	-0.161455	-1.787011
H	6.241616	0.051896	-3.542435
H	6.823352	-1.479801	-2.860677
C	4.330615	-1.726427	-4.187196
H	5.069235	-2.451162	-4.548422
H	4.339378	-0.882040	-4.884588
H	3.339635	-2.190573	-4.225787
C	3.097744	-2.478748	1.896721
H	3.288974	-1.407868	2.032800
C	1.575067	-2.681206	1.892177
H	1.111479	-2.131387	1.065646
H	1.135316	-2.335057	2.835829
H	1.329401	-3.743293	1.769680
C	3.745400	-3.227901	3.066655
H	4.836902	-3.139358	3.048964
H	3.495613	-4.294442	3.051447
H	3.383810	-2.822037	4.017967
N	-1.814202	-0.354684	0.064250
N	-4.151173	-0.836512	0.187724
N	-3.410232	0.832178	1.387262
N	1.814431	0.361229	-0.883583
N	3.597880	1.625559	-0.004131
N	3.975021	-0.545313	-0.064778
C	-0.949678	-3.311692	-2.563215
C	0.612899	-0.929603	-2.906017
C	0.259397	-3.246701	-1.961063
C	1.102624	-2.062622	-2.018457
C	-1.391699	-2.246971	-3.440839
C	-0.613016	-1.160195	-3.648775
H	0.650158	-4.110338	-1.424283
H	2.166854	-2.266116	-2.005932
H	-2.313626	-2.378439	-3.999905
H	-0.886803	-0.431999	-4.410823
H	1.368773	-0.324889	-3.409629
H	-1.557902	-4.207395	-2.477226

Calculated energies and coordinates of **TS[I-J]**

Electronic energy ... -2868.15196147 Eh
Total Enthalpy ... -2866.70996768 Eh
Final Gibbs free energy ... -2866.88096144 Eh

CARTESIAN COORDINATES (ANGSTROEM)

B	-0.249311	-0.490258	1.338871
B	-0.039924	1.123510	1.161524
C	-0.321656	-3.001298	0.919609
C	0.143791	-5.173313	1.459627
C	-0.144732	-5.138864	0.135229
C	0.528868	-6.300905	2.346683
H	0.727341	-7.193436	1.749687
H	-0.257557	-6.544381	3.071296
H	1.430389	-6.062621	2.921985
C	-0.173341	-6.205887	-0.895642
H	0.145930	-7.156975	-0.465430
H	0.489026	-5.963689	-1.734676
H	-1.180600	-6.338450	-1.309136

C	0.248295	-3.509704	3.312515
C	-0.744494	-3.863132	4.240064
C	-0.484361	-3.613939	5.587550
H	-1.225339	-3.869535	6.337821
C	0.709829	-3.023056	5.980367
H	0.893923	-2.829944	7.033503
C	1.659652	-2.654729	5.037168
H	2.569794	-2.163946	5.363727
C	1.452992	-2.891424	3.677561
C	-2.090871	-4.393739	3.779552
H	-1.936489	-4.987925	2.870414
C	-2.778378	-5.296814	4.806108
H	-3.117794	-4.731472	5.680600
H	-2.114654	-6.095735	5.154196
H	-3.664368	-5.758188	4.358083
C	-3.002189	-3.215568	3.395247
H	-3.964527	-3.582698	3.020591
H	-2.542285	-2.592520	2.622066
H	-3.191722	-2.579980	4.268156
C	2.487966	-2.499865	2.636142
H	1.944702	-2.130517	1.757618
C	3.398387	-1.359345	3.093195
H	2.813481	-0.498632	3.433999
H	4.018179	-1.034351	2.252153
H	4.076980	-1.674025	3.894825
C	3.327482	-3.711790	2.199925
H	3.854141	-4.144583	3.058711
H	4.076798	-3.401272	1.462902
H	2.713988	-4.493454	1.741473
C	-0.660506	-3.328112	-1.512979
C	-1.954880	-3.398675	-2.047212
C	-2.133644	-2.965730	-3.361002
H	-3.122660	-2.993856	-3.808145
C	-1.058442	-2.497520	-4.106920
H	-1.214281	-2.168237	-5.130655
C	0.211566	-2.441484	-3.550937
H	1.042603	-2.071478	-4.142929
C	0.439093	-2.846775	-2.235580
C	-3.126722	-3.870806	-1.207233
H	-2.729600	-4.473575	-0.380791
C	-4.108551	-4.750095	-1.988360
H	-4.649926	-4.176691	-2.748197
H	-4.856207	-5.170265	-1.307375
H	-3.597268	-5.577933	-2.491314
C	-3.846615	-2.661953	-0.588675
H	-3.151324	-2.033956	-0.022698
H	-4.648557	-2.993880	0.080640
H	-4.290974	-2.044430	-1.377300
C	1.824742	-2.734828	-1.629460
H	1.799204	-3.178916	-0.627241
C	2.867503	-3.513234	-2.442976
H	2.588293	-4.566522	-2.556033
H	3.841341	-3.467989	-1.943512
H	2.990957	-3.087031	-3.444474
C	2.233334	-1.261811	-1.479897
H	2.373263	-0.790256	-2.459106
H	3.185146	-1.183447	-0.941602
H	1.482126	-0.680253	-0.936939
C	0.239384	2.902917	-0.625520
C	0.819708	4.662854	-2.017020
C	-0.343613	5.005515	-1.424543
C	1.713802	5.452704	-2.902011
H	1.288610	6.442781	-3.079525
H	1.880450	4.970266	-3.871703
H	2.700845	5.584273	-2.439927
C	-1.153675	6.239749	-1.590984
H	-0.938122	6.699800	-2.558327
H	-0.948768	6.983859	-0.812107
H	-2.225462	6.013853	-1.557574
C	2.323095	2.670981	-1.990665
C	2.349282	2.215466	-3.322868
C	3.501385	1.573783	-3.778056
H	3.550058	1.219589	-4.803840
C	4.582372	1.363446	-2.931155
H	5.470479	0.857557	-3.300376
C	4.524858	1.791697	-1.612805
H	5.373641	1.618784	-0.956393
C	3.402301	2.459548	-1.118415
C	1.125263	2.312876	-4.216122
H	0.467471	3.096360	-3.824112
C	0.354103	0.988185	-4.140847
H	-0.597138	1.055961	-4.682765
H	0.148972	0.707292	-3.102866
H	0.947878	0.187481	-4.595297
C	1.450824	2.663010	-5.672055
H	1.973480	1.844591	-6.179255
H	2.078528	3.557282	-5.746781
H	0.524591	2.847820	-6.226843
C	3.393279	2.951633	0.314974
H	2.424158	3.420731	0.508758
C	4.481355	4.011166	0.540543
H	5.483185	3.592046	0.392940
H	4.427383	4.397626	1.564484
H	4.364943	4.852823	-0.150826
C	3.540682	1.784437	1.297330
H	2.758074	1.039763	1.126768
H	3.444600	2.138104	2.329896
H	4.520755	1.302373	1.196573
C	-2.034180	3.793661	-0.044982
C	-2.494822	4.530871	1.066187
C	-3.804535	4.316952	1.501346
H	-4.174676	4.868310	2.360892
C	-4.633641	3.400829	0.872608
H	-5.643318	3.238469	1.239564
C	-4.172211	2.699645	-0.231565
H	-4.832532	1.997644	-0.733399
C	-2.880156	2.892132	-0.721817
C	-1.649058	5.546929	1.814766
H	-0.647794	5.548183	1.366976
C	-2.249854	6.959770	1.694894
H	-2.473347	7.233031	0.661359
H	-1.560615	7.703201	2.111318
H	-3.186146	7.024748	2.260340
C	-1.512507	5.202329	3.308168
H	-2.472067	5.322799	3.823131
H	-0.797983	5.883668	3.784029
H	-1.176725	4.176528	3.469481
C	-2.465825	2.157916	-1.984099
H	-1.424261	2.401717	-2.207288
C	-2.558086	0.638415	-1.805359
H	-1.926698	0.305275	-0.975819
H	-2.229689	0.122015	-2.714286
H	-3.589587	0.328788	-1.603360
C	-3.306836	2.631453	-3.179321
H	-4.367074	2.388935	-3.044870
H	-2.964335	2.141861	-4.098293
H	-3.223571	3.715297	-3.316048
N	-0.512883	-1.725983	0.915406
N	0.033515	-3.856846	1.941909
N	-0.442711	-3.811215	-0.185569
N	0.273105	1.748963	-0.104298
N	1.189600	3.398684	-1.538144
N	-0.699403	3.972641	-0.524867
C	-0.727293	-0.235630	3.895125
C	-0.050011	2.056860	2.464977
C	-1.710040	0.319879	3.094704
C	-1.456422	1.486539	2.336988
C	0.343806	0.612432	4.384276
C	0.613399	1.768444	3.753283
H	-2.675320	-0.171985	2.989432
H	-2.273687	2.040361	1.890504
H	0.838643	0.339494	5.313412
H	1.291011	2.505861	4.177421
H	0.039069	3.095522	2.141355

H	-0.914738	-1.182539	4.389215

Calculated energies and coordinates of **J**

Electronic energy ... -2868.19798998 Eh
Total Enthalpy ... -2866.75380984 Eh
Final Gibbs free energy ... -2866.92392613 Eh

CARTESIAN COORDINATES (ANGSTROEM)

B	0.816877	0.280199	1.561626
B	-0.817353	-0.279594	1.561966
C	2.649706	-0.008869	-0.213832
C	4.285071	-0.113535	-1.832859
C	4.333809	-1.275555	-1.146407
C	5.067027	0.348914	-3.007237
H	4.477286	0.312150	-3.931686
H	5.949815	-0.278854	-3.144304
H	5.401081	1.385144	-2.879318
C	5.194657	-2.471609	-1.328049
H	5.836310	-2.345427	-2.202439
H	4.596026	-3.379111	-1.470417
H	5.833853	-2.649179	-0.455989
C	3.082966	2.063568	-1.548266
C	3.715113	2.988732	-0.695635
C	3.617403	4.345622	-1.003375
H	4.106500	5.074967	-0.364334
C	2.906830	4.780095	-2.113245
H	2.840124	5.841386	-2.335675
C	2.277422	3.857451	-2.933906
H	1.717069	4.205348	-3.797472
C	2.355150	2.487046	-2.672796
C	4.516693	2.556947	0.515881
H	4.294783	1.505267	0.712321
C	6.022639	2.671197	0.239882
H	6.314851	2.060221	-0.620865
H	6.596095	2.330532	1.109592
H	6.305964	3.709557	0.031982
C	4.122674	3.345189	1.770389
H	3.044511	3.281050	1.945844
H	4.395571	4.402831	1.688964
H	4.638987	2.941911	2.647487
C	1.640612	1.520459	-3.595015
H	1.891203	0.503525	-3.273299
C	0.121278	1.697605	-3.475667
H	-0.401171	1.038175	-4.175694
H	-0.169700	2.725561	-3.714606
H	-0.224164	1.473845	-2.460916
C	2.076148	1.690889	-5.057779
H	3.164775	1.667985	-5.164870
H	1.722862	2.643211	-5.468378
H	1.651642	0.890114	-5.674100
C	3.277825	-2.143869	0.950219
C	4.317034	-2.118187	1.898801
C	4.288189	-3.052879	2.935482
H	5.078369	-3.048921	3.680651
C	3.254417	-3.970374	3.039557
H	3.242997	-4.686687	3.856458
C	2.224277	-3.962644	2.110356
H	1.409536	-4.670750	2.215434
C	2.213170	-3.056539	1.049787
C	5.409063	-1.063467	1.891782
H	5.407411	-0.559539	0.918121
C	6.813170	-1.642883	2.110962
H	6.938673	-2.022133	3.130707
H	7.566063	-0.861826	1.959391
H	7.030394	-2.464539	1.421382
C	5.100047	-0.011806	2.966937
H	5.098479	-0.468766	3.963268
H	4.116515	0.437300	2.802867
H	5.855324	0.782436	2.957628
C	1.101924	-3.096817	0.018402
H	0.901143	-2.066065	-0.298604
C	1.542848	-3.912382	-1.208314
H	0.692248	-4.058997	-1.881796
H	1.905144	-4.899541	-0.897541
H	2.338363	-3.410013	-1.767745
C	-0.211121	-3.659803	0.564914
H	-0.488205	-3.176461	1.506687
H	-0.155458	-4.743312	0.726090
H	-1.012095	-3.474177	-0.154811
C	-2.650043	0.008740	-0.213734
C	-4.333653	1.275229	-1.147428
C	-4.285064	0.112780	-1.833161
C	-5.194321	2.471300	-1.329828
H	-5.835665	2.344884	-2.204409
H	-4.595563	3.378711	-1.472237
H	-5.833821	2.649203	-0.458051
C	-5.066933	-0.350242	-3.007366
H	-5.400943	-1.386425	-2.879002
H	-4.477131	-0.313850	-3.931797
H	-5.949703	0.277467	-3.144822
C	-3.278023	2.144487	0.948999
C	-2.213275	3.057077	1.048192
C	-2.224319	3.963685	2.108331
H	-1.409508	4.671754	2.213101
C	-3.254498	3.971946	3.037490
H	-3.243047	4.688642	3.854056
C	-4.288368	3.054522	2.933795
H	-5.078531	3.050935	3.678985
C	-4.317251	2.119326	1.897566
C	-1.102045	3.096779	0.016770
H	-0.901607	2.065913	-0.300070
C	0.211216	3.659395	0.563162
H	0.155900	4.742949	0.724158
H	1.012107	3.473414	-0.156551
H	0.488173	3.176073	1.504984
C	-1.542728	3.912277	-1.210094
H	-1.904699	4.899606	-0.899516
H	-2.338436	3.410081	-1.769429
H	-0.692089	4.058368	-1.883644
C	-5.409384	1.064735	1.890950
H	-5.408065	0.560736	0.917328
C	-6.813364	1.644319	2.110577
H	-6.938553	2.023406	3.130424
H	-7.566404	0.863388	1.959088
H	-7.030700	2.466128	1.421214
C	-5.100189	0.013076	2.966047
H	-5.098412	0.470036	3.962372
H	-4.116727	-0.436121	2.801777
H	-5.855496	-0.781141	2.956795
C	-3.082907	-2.064209	-1.547486
C	-3.714986	-2.989165	-0.694595
C	-3.616823	-4.346166	-1.001724
H	-4.105852	-5.075370	-0.362468
C	-2.905896	-4.780928	-2.111249
H	-2.838859	-5.842297	-2.333203
C	-2.276604	-3.858461	-2.932206
H	-1.716000	-4.206564	-3.795525
C	-2.354770	-2.487967	-2.671707
C	-4.516978	-2.557112	0.516550
H	-4.295575	-1.505239	0.712524
C	-6.022823	-2.672104	0.240312
H	-6.305783	-3.710727	0.033236
H	-6.315050	-2.061911	-0.620984
H	-6.596553	-2.330917	1.109640
C	-4.122875	-3.344599	1.771498
H	-3.044847	-3.279536	1.947388
H	-4.394871	-4.402489	1.690276
H	-4.639902	-2.941475	2.648244
C	-1.640388	-1.521563	-3.594251
H	-1.891234	-0.504560	-3.272963
C	-0.121032	-1.698326	-3.474747

H	0.170205	-2.726257	-3.713373
H	0.224188	-1.474110	-2.460004
H	0.401341	-1.038732	-4.174676
C	-2.075868	-1.692648	-5.056974
H	-3.164495	-1.670041	-5.164090
H	-1.722342	-2.645032	-5.467224
H	-1.651489	-0.892030	-5.673589
N	1.719912	0.418492	0.527233
N	3.239006	0.665016	-1.295075
N	3.326771	-1.240664	-0.159128
N	-1.720620	-0.418488	0.527880
N	-3.326899	1.240691	-0.159866
N	-3.239134	-0.665579	-1.294806
C	1.178586	0.652159	3.112952
C	-1.178661	-0.650886	3.113540
C	1.185662	-0.752428	3.668810
C	0.011471	-1.397342	3.664490
C	-0.011416	1.398862	3.663858
C	-1.185607	0.753945	3.668778
H	2.114126	-1.215351	3.991849
H	-0.094845	-2.419102	4.018737
H	0.094982	2.420770	4.017660
H	-2.113986	1.217031	3.991823
H	-2.124699	-1.178349	3.263719
H	2.124654	1.179721	3.262616

Calculated energies and coordinates of **TS[2-3]**

Electronic energy ... -2635.95373927 Eh
Total Enthalpy ... -2634.61981607 Eh
Final Gibbs free energy ... -2634.78174148 Eh

CARTESIAN COORDINATES (ANGSTROEM)

B	0.218219	1.682984	0.440747
B	0.945529	0.204801	0.877206
C	2.404026	0.343503	1.433481
C	3.186352	1.564451	1.339413
C	2.481622	2.718271	1.263336
H	2.986826	3.658437	1.056452
C	1.017343	2.749331	1.502392
H	0.617843	3.768300	1.500978
C	0.605065	1.947253	2.692682
H	0.096972	2.423653	3.528827
C	0.813022	0.617213	2.598339
C	0.585934	-0.396820	3.682726
H	0.766125	-1.394053	3.258065
C	-0.876170	-0.348801	4.143899
H	-1.071118	-1.114959	4.903607
H	-1.552366	-0.510969	3.299409
H	-1.112065	0.626937	4.584065
C	1.549466	-0.200712	4.859456
H	1.373376	0.764440	5.348027
H	2.589448	-0.217133	4.519042
H	1.420122	-0.993372	5.606335
C	4.698912	1.534042	1.179972
H	5.003701	0.483826	1.131386
C	5.410111	2.194045	2.369127
H	6.496758	2.056858	2.307777
H	5.061927	1.787041	3.323588
H	5.202497	3.269702	2.380652
C	5.149259	2.179203	-0.137633
H	4.894964	3.243939	-0.165632
H	4.666664	1.697162	-0.992851
H	6.236037	2.091645	-0.254686
C	1.821744	-1.790358	0.998557
C	3.456418	-3.092327	1.868745
C	3.822403	-1.806778	2.132325
C	4.122877	-4.373676	2.209161
H	4.392590	-4.933664	1.304909
H	3.465183	-5.021769	2.802036
H	5.035979	-4.190897	2.778581
C	4.971375	-1.316083	2.939726
H	5.845808	-1.043201	2.336800
H	5.283636	-2.086760	3.649230
H	4.685025	-0.431235	3.516078
C	1.556975	-4.232792	0.645529
C	0.627931	-4.909123	1.450524
C	-0.030712	-6.011729	0.904515
H	-0.760881	-6.549599	1.502196
C	0.224114	-6.422991	-0.396432
H	-0.301047	-7.281675	-0.805615
C	1.141991	-5.735901	-1.178318
H	1.325336	-6.060257	-2.198708
C	1.826871	-4.629814	-0.673560
C	2.781614	-3.856004	-1.560501
H	3.341154	-3.155376	-0.929651
C	1.983717	-3.029758	-2.580886
H	2.661142	-2.429165	-3.198680
H	1.282678	-2.360844	-2.070590
H	1.410553	-3.688364	-3.244455
C	3.799298	-4.764115	-2.261324
H	3.316642	-5.429447	-2.985166
H	4.342108	-5.386461	-1.542061
H	4.528082	-4.157178	-2.809155
C	0.277259	-4.422390	2.841168
H	1.057914	-3.721868	3.161871
C	-1.051804	-3.652336	2.785974
H	-0.986094	-2.810690	2.088748
H	-1.316998	-3.267196	3.776426
H	-1.858379	-4.316183	2.451060
C	0.217259	-5.554990	3.872296
H	-0.622236	-6.232080	3.681955
H	0.078594	-5.138680	4.875766
H	1.136730	-6.150177	3.871082
C	-1.736923	1.884266	-1.201395
C	-3.723348	1.476706	-2.268986
C	-2.906607	2.085422	-3.160850
C	-3.138037	2.487068	-4.570651
H	-3.124173	3.576265	-4.700960
H	-4.108226	2.116618	-4.909572
H	-2.361633	2.077425	-5.227903
C	-5.173567	1.151788	-2.353108
H	-5.447680	0.392973	-1.616196
H	-5.440819	0.779208	-3.345722
H	-5.788457	2.038235	-2.149324
C	-3.401185	0.520689	0.040240
C	-3.834093	1.166862	1.207889
C	-4.225080	0.373749	2.287083
H	-4.553361	0.846473	3.208990
C	-4.202928	-1.011024	2.196684
H	-4.508746	-1.614886	3.046864
C	-3.783146	-1.628637	1.026544
H	-3.765961	-2.713125	0.970550
C	-3.364357	-0.882366	-0.076117
C	-2.913867	-1.602327	-1.338304
H	-2.484477	-0.857263	-2.018980
C	-1.831883	-2.653048	-1.052714
H	-1.467160	-3.070552	-1.999009
H	-2.232212	-3.489926	-0.467342
H	-0.984764	-2.223990	-0.509319
C	-4.101692	-2.272313	-2.050688
H	-4.553697	-3.033531	-1.404483
H	-3.755173	-2.770993	-2.962575
H	-4.883029	-1.561143	-2.329063
C	-3.905106	2.678316	1.312283
H	-3.587306	3.101161	0.352688
C	-5.344057	3.145392	1.570667
H	-6.028428	2.761815	0.806279
H	-5.395492	4.239945	1.561899
H	-5.706357	2.802740	2.546273
C	-2.946343	3.207880	2.384887

H	-3.220060	2.838141	3.380000
H	-2.971468	4.303303	2.411483
H	-1.923960	2.887683	2.167904
C	-0.637360	3.154166	-3.020773
C	0.581148	2.574343	-3.396036
C	1.554272	3.410761	-3.948372
H	2.505974	2.989249	-4.258800
C	1.326315	4.770325	-4.098088
H	2.093283	5.403175	-4.536003
C	0.131483	5.332480	-3.664136
H	-0.015929	6.404451	-3.751195
C	-0.868517	4.539571	-3.102924
C	-2.110182	5.167046	-2.494045
H	-2.912327	4.419349	-2.492332
C	-1.826785	5.529799	-1.024932
H	-1.041820	6.292916	-0.967760
H	-1.488667	4.653842	-0.461503
H	-2.730188	5.929391	-0.548857
C	-2.621254	6.387462	-3.265370
H	-3.589382	6.703147	-2.862735
H	-2.748028	6.169578	-4.331377
H	-1.940867	7.240738	-3.173192
C	0.867309	1.097384	-3.207075
H	0.081868	0.671464	-2.574561
C	2.197670	0.882266	-2.473623
H	3.054375	1.130382	-3.111466
H	2.293069	-0.164640	-2.169727
H	2.248103	1.494727	-1.567999
C	0.843118	0.356523	-4.550793
H	-0.123923	0.474444	-5.051952
H	1.021117	-0.714036	-4.399831
H	1.619427	0.740568	-5.223091
N	-0.841016	2.083955	-0.312902
N	-1.681754	2.322312	-2.516228
N	-2.998823	1.323223	-1.072095
N	0.740674	-1.240585	0.567899
N	2.844803	-0.978494	1.537361
N	2.238421	-3.092572	1.168224

Calculated energies and coordinates of **3**

Electronic energy ... -2636.03279375 Eh
Total Enthalpy ... -2634.69593588 Eh
Final Gibbs free energy ... -2634.85706742 Eh

CARTESIAN COORDINATES (ANGSTROEM)

B	7.668118	18.158016	13.509537
B	8.081752	16.173924	14.997580
C	8.866736	17.362343	14.228986
C	9.363464	18.445028	15.165855
C	8.364513	19.283599	15.484820
H	8.429226	20.084783	16.214827
C	7.051936	18.882535	14.819292
H	6.372686	19.724644	14.665784
C	6.357741	17.815120	15.653193
H	5.428091	18.125158	16.133095
C	6.841660	16.560498	15.834105
C	6.229407	15.558743	16.794913
H	6.083682	14.620383	16.236035
C	4.890015	15.974074	17.400432
H	4.490855	15.172144	18.031299
H	4.148454	16.202843	16.627488
H	5.004371	16.865457	18.029461
C	7.244599	15.251666	17.909492
H	7.445704	16.157628	18.494314
H	8.190918	14.902317	17.484969
H	6.863310	14.479602	18.588488
C	10.748651	18.394647	15.744468
H	11.455173	18.214882	14.919651
C	10.864862	17.195699	16.701139
H	11.890706	17.094617	17.075346
H	10.583386	16.261611	16.206462
H	10.192730	17.332216	17.556181
C	11.149858	19.692596	16.443292
H	10.523481	19.869400	17.324838
H	11.044003	20.553184	15.774226
H	12.190655	19.640781	16.780727
C	9.615943	15.242129	13.796636
C	11.289552	15.445225	12.287333
C	10.833422	16.703185	12.556529
C	12.370762	14.964210	11.391854
H	13.131952	14.399785	11.945186
H	12.861601	15.814286	10.912826
H	11.991705	14.300412	10.603259
C	11.190610	18.024404	11.981494
H	10.301360	18.495769	11.547535
H	11.940301	17.921011	11.194222
H	11.583662	18.709854	12.742269
C	10.758705	13.128896	13.149443
C	11.551494	12.646625	14.199465
C	11.773410	11.269086	14.265923
H	12.374771	10.862046	15.073866
C	11.235885	10.414143	13.315217
H	11.421913	9.345708	13.381701
C	10.457232	10.916304	12.278937
H	10.041002	10.233336	11.545004
C	10.201410	12.282886	12.177439
C	9.292582	12.844076	11.103066
H	9.605163	13.876784	10.912077
C	7.848720	12.898595	11.627064
H	7.192120	13.372503	10.886841
H	7.789970	13.462659	12.563553
H	7.475449	11.885128	11.818200
C	9.368476	12.083783	9.777622
H	8.928051	11.083652	9.854682
H	10.402398	11.975173	9.432253
H	8.806392	12.623509	9.007683
C	12.118020	13.567062	15.262055
H	11.993322	14.600463	14.917530
C	11.324412	13.409310	16.568088
H	10.260554	13.601550	16.396086
H	11.690036	14.112072	17.325435
H	11.434222	12.392664	16.963831
C	13.616976	13.340525	15.492302
H	13.817187	12.355260	15.926983
H	14.004601	14.090895	16.189723
H	14.181050	13.417290	14.556629
C	6.643309	18.623269	11.242738
C	4.807873	19.005357	9.923791
C	5.900514	19.344937	9.200544
C	6.017036	19.870329	7.817968
H	6.606872	20.794310	7.793493
H	5.022730	20.083281	7.418439
H	6.508780	19.157943	7.143685
C	3.364813	19.024787	9.550471
H	2.739720	18.850461	10.430116
H	3.123205	18.248311	8.815216
H	3.080038	19.991302	9.122119
C	4.422430	18.431952	12.338527
C	3.868183	19.593602	12.901691
C	3.038412	19.439925	14.014293
H	2.600383	20.317470	14.481631
C	2.766261	18.182168	14.530269
H	2.119708	18.080864	15.397642
C	3.326122	17.049677	13.952531
H	3.112129	16.076219	14.378578
C	4.172173	17.147722	12.849209
C	4.788525	15.915635	12.207833
H	5.828331	16.164039	11.955973
C	4.827194	14.708804	13.144751
H	5.397036	13.899529	12.680157
H	3.822138	14.323041	13.352026

H	5.315564	14.959579	14.090456
C	4.055114	15.550804	10.906416
H	2.999306	15.336619	11.110023
H	4.503065	14.655774	10.460017
H	4.105409	16.356112	10.168297
C	4.133031	20.985762	12.348593
H	4.680211	20.885987	11.405000
C	2.823759	21.729353	12.042938
H	2.159476	21.133569	11.409623
H	3.037872	22.671724	11.527087
H	2.279070	21.973679	12.961089
C	5.008732	21.821192	13.293284
H	4.543949	21.919116	14.280810
H	5.149182	22.827949	12.882707
H	5.994750	21.368848	13.428690
C	8.372821	19.370767	9.626698
C	9.029735	18.448260	8.795617
C	10.332438	18.746411	8.397460
H	10.869747	18.056670	7.754107
C	10.957285	19.913734	8.820173
H	11.972471	20.129717	8.498655
C	10.295963	20.797647	9.659528
H	10.800596	21.699068	9.995946
C	8.988420	20.545056	10.081655
C	8.292495	21.519642	11.011474
H	7.287418	21.136742	11.218075
C	9.031314	21.619037	12.353527
H	10.033679	22.043275	12.223097
H	9.131919	20.636447	12.824775
H	8.480881	22.270010	13.041977
C	8.134151	22.900271	10.360849
H	7.579341	23.570213	11.026958
H	7.591773	22.833986	9.411526
H	9.107793	23.360424	10.159662
C	8.376617	17.132236	8.421175
H	7.288661	17.268541	8.448595
C	8.727945	16.070092	9.475745
H	9.811478	15.899751	9.496002
H	8.234289	15.121557	9.234988
H	8.422247	16.382934	10.479773
C	8.747917	16.645850	7.017418
H	8.584353	17.421985	6.261963
H	8.138122	15.775672	6.753222
H	9.795879	16.332439	6.960494
N	7.471230	18.238375	12.150522
N	7.021874	19.107018	10.003880
N	5.259228	18.566275	11.184671
N	8.651798	14.908311	14.641558
N	9.826107	16.562679	13.501530
N	10.521472	14.535023	13.055349

Calculated energies and coordinates of **TS[2-INT1]**

Electronic energy ... -2884.21878642 Eh
Total Enthalpy ... -2882.78801773 Eh
Final Gibbs free energy ... -2882.95748456 Eh

CARTESIAN COORDINATES (ANGSTROEM)

N	-3.469053	-1.498364	-1.267586
N	-1.133599	-1.103155	-1.492413
N	0.770962	1.045760	1.025402
N	2.818558	1.790953	0.149953
N	-2.113644	-3.028877	-0.451786
N	2.513584	2.194136	2.305487
N	-0.337975	2.407447	-1.206016
C	-2.792264	-0.779424	-5.455960
H	-2.232329	-1.578381	-5.952836
H	-2.112072	0.065364	-5.302697
H	-3.585449	-0.464281	-6.143582
C	-3.351832	-1.294821	-4.127680
H	-2.507954	-1.716362	-3.570036
C	-3.949353	-0.194896	-3.267139
C	-3.961163	-0.303877	-1.865829
C	-2.117480	-1.785544	-1.084797
B	0.088263	-0.471091	-1.445971
B	0.776353	0.655892	-0.378616
C	1.913201	1.648226	1.197790
C	2.349893	0.946206	-0.949145
C	2.853528	-0.496871	-0.904742
C	3.894486	-0.977022	0.081568
H	4.074902	-0.180421	0.816303
C	5.227641	-1.268922	-0.626769
H	5.112776	-2.121894	-1.305009
H	6.014162	-1.508282	0.099454
H	5.559970	-0.417822	-1.229338
C	-4.369399	-2.419667	-4.372170
H	-5.268126	-2.028077	-4.863220
H	-4.665834	-2.899759	-3.434942
H	-3.935267	-3.190789	-5.018535
C	-4.458326	0.715364	-1.037893
C	-5.028654	1.832347	-1.651818
H	-5.431262	2.634911	-1.041499
C	-5.076896	1.936254	-3.034571
H	-5.533500	2.808590	-3.495156
C	-4.522204	0.943868	-3.834646
H	-4.542421	1.056293	-4.914128
C	-4.295604	0.653057	0.469022
H	-4.206363	-0.400374	0.761032
C	-2.982789	1.350210	0.864770
H	-2.116335	0.883449	0.389165
H	-2.833589	1.306758	1.949307
H	-3.003370	2.404762	0.561611
C	-5.482225	1.248638	1.232797
H	-5.534139	2.336828	1.117787
H	-5.373303	1.044121	2.302873
H	-6.436041	0.826413	0.897442
C	-4.266904	-2.522179	-0.724247
C	-5.749327	-2.442517	-0.752966
H	-6.120823	-2.210946	-1.758125
H	-6.128042	-1.656997	-0.086281
H	-6.184685	-3.392246	-0.435097
C	-3.434075	-3.465358	-0.224834
C	-3.717763	-4.778978	0.407979
H	-4.783293	-5.007567	0.336352
H	-3.437454	-4.800460	1.468405
H	-3.158661	-5.583355	-0.084834
C	-0.938736	-3.742968	-0.076591
C	-0.596777	-3.799208	1.284305
C	-1.350363	-2.977131	2.313587
H	-2.393373	-2.884360	1.988463
C	-0.761392	-1.560073	2.348875
H	0.299919	-1.590039	2.613735
H	-1.277709	-0.937545	3.088564
H	-0.825555	-1.060149	1.380391
C	-1.358196	-3.604452	3.709877
H	-1.696154	-4.646674	3.687695
H	-2.030551	-3.039148	4.363563
H	-0.365510	-3.578949	4.172871
C	0.519573	-4.556111	1.643745
H	0.813688	-4.620775	2.686622
C	1.273014	-5.211950	0.680950
H	2.142187	-5.792373	0.978449
C	0.936642	-5.113938	-0.663635
H	1.551156	-5.619289	-1.401211
C	-0.177872	-4.381202	-1.072994
C	-0.574931	-4.287222	-2.537374
H	-0.859555	-3.243770	-2.722198
C	0.565671	-4.613786	-3.503408
H	0.250667	-4.389204	-4.527732
H	0.837296	-5.675416	-3.473341
H	1.461191	-4.020930	-3.291336
C	-1.790822	-5.176373	-2.846598

H	-2.033307	-5.125059	-3.914232
H	-2.677362	-4.860977	-2.289530
H	-1.574857	-6.222206	-2.597861
C	3.804835	2.652606	1.949054
C	4.005032	2.365621	0.634854
C	2.288064	1.459027	-2.369472
C	2.877384	2.788084	-2.788787
H	3.041342	3.381331	-1.878357
C	4.227393	2.575544	-3.497306
H	4.054954	2.103483	-4.470837
H	4.885499	1.912206	-2.930217
H	4.747705	3.527439	-3.663631
C	1.951158	3.602498	-3.699489
H	1.763058	3.075166	-4.640794
H	2.412928	4.565747	-3.947881
H	0.987060	3.795791	-3.223018
C	1.671766	0.612730	-3.206357
H	1.535299	0.836669	-4.261204
C	1.177550	-0.697807	-2.659040
H	0.826426	-1.378806	-3.441368
C	2.241641	-1.308716	-1.787004
H	2.443648	-2.375754	-1.832090
C	3.422816	-2.220107	0.845529
H	2.457756	-2.043143	1.330190
H	4.157354	-2.509056	1.607345
H	3.291652	-3.071438	0.169489
C	5.210376	2.620529	-0.193322
H	5.499530	1.722740	-0.748456
H	6.050102	2.899601	0.447150
H	5.060102	3.422646	-0.924516
C	4.632821	3.406820	2.924548
H	5.641140	3.557525	2.533271
H	4.710599	2.880530	3.883068
H	4.204044	4.395550	3.138415
C	2.003835	2.182709	3.641243
C	1.353120	3.325775	4.125632
C	0.952318	3.339335	5.462983
H	0.443013	4.211095	5.863196
C	1.187704	2.247039	6.283971
H	0.877747	2.274317	7.324999
C	1.796587	1.106993	5.773849
H	1.943933	0.249210	6.421871
C	2.209033	1.045052	4.442531
C	2.847823	-0.211531	3.877576
H	2.517377	-0.291750	2.835084
C	4.382887	-0.127701	3.888122
H	4.814112	-1.075328	3.546344
H	4.747880	0.064842	4.904183
H	4.751636	0.662221	3.228473
C	2.399621	-1.485657	4.601588
H	1.313171	-1.519264	4.727864
H	2.864843	-1.574905	5.590282
H	2.703907	-2.361799	4.018465
C	0.990026	4.473516	3.206033
H	1.615370	4.397772	2.307825
C	1.233872	5.852755	3.826541
H	2.258189	5.949016	4.201607
H	0.549629	6.050587	4.658393
H	1.067486	6.634931	3.077949
C	-0.478215	4.309138	2.777981
H	-0.767605	5.091318	2.066421
H	-1.136066	4.384905	3.651842
H	-0.639551	3.330976	2.311876
C	-0.105559	3.588475	-0.633155
H	0.606658	3.585363	0.188833
C	-0.728318	4.759978	-1.047891
H	-0.494649	5.702154	-0.561550
C	-1.645265	4.692378	-2.090458
H	-2.153991	5.586904	-2.438955
C	-1.897039	3.459326	-2.679809
H	-2.612173	3.351624	-3.488011
C	-1.216913	2.342097	-2.210035
H	-1.384550	1.357493	-2.643020

Calculated energies and coordinates of **INT1**

Electronic energy ... -2884.22598456 Eh
Total Enthalpy ... -2882.79415298 Eh
Final Gibbs free energy ... -2882.96290430 Eh

CARTESIAN COORDINATES (ANGSTROEM)

N	-3.369647	-1.571974	-1.324479
N	-1.000083	-1.254307	-1.364243
N	0.688010	1.160721	0.977449
N	2.663644	1.981443	0.004096
N	-2.113093	-3.128540	-0.400135
N	2.583320	2.090231	2.225466
N	-0.394774	2.344116	-0.987755
C	-2.276221	-0.865931	-5.463095
H	-1.643884	-1.651019	-5.890191
H	-1.649959	0.017860	-5.298470
H	-3.035005	-0.616862	-6.213912
C	-2.899487	-1.365008	-4.157525
H	-2.075657	-1.724176	-3.529827
C	-3.611813	-0.274528	-3.376096
C	-3.795348	-0.386380	-1.985721
C	-2.039083	-1.892000	-1.043425
B	-0.043920	-0.281475	-1.511790
B	0.470539	1.044088	-0.525520
C	1.847082	1.704104	1.114870
C	2.144484	1.199242	-1.111972
C	2.589885	-0.263068	-1.099742
C	3.542337	-0.830938	-0.071316
H	3.625099	-0.115072	0.754196
C	4.952908	-1.023250	-0.649342
H	4.933865	-1.771284	-1.450062
H	5.647569	-1.365203	0.127837
H	5.345130	-0.094247	-1.072237
C	-3.839199	-2.550316	-4.431567
H	-4.718036	-2.222699	-4.999445
H	-4.179244	-3.014969	-3.501306
H	-3.320234	-3.317388	-5.017379
C	-4.414728	0.622428	-1.229260
C	-4.937629	1.722837	-1.911291
H	-5.432662	2.513515	-1.356058
C	-4.815962	1.827545	-3.289068
H	-5.236696	2.686507	-3.805650
C	-4.135699	0.853092	-4.010214
H	-4.023914	0.967623	-5.083708
C	-4.421798	0.575385	0.287188
H	-4.345178	-0.471955	0.602272
C	-3.171327	1.301248	0.810749
H	-2.252140	0.831265	0.449203
H	-3.145909	1.289636	1.905942
H	-3.171710	2.346223	0.476901
C	-5.692395	1.160695	0.910741
H	-5.744281	2.247486	0.783184
H	-5.700272	0.963699	1.987694
H	-6.598789	0.725728	0.475424
C	-4.228566	-2.570096	-0.823319
C	-5.700127	-2.483380	-1.000382
H	-5.968139	-2.316904	-2.051043
H	-6.135721	-1.655907	-0.426398
H	-6.173789	-3.409537	-0.668360
C	-3.454691	-3.522873	-0.251577
C	-3.814085	-4.801392	0.413092
H	-4.883507	-4.994700	0.306292
H	-3.577294	-4.786334	1.484416
H	-3.264839	-5.645260	-0.020964
C	-0.991622	-3.810027	0.157505
C	-0.785624	-3.736435	1.544315
C	-1.622109	-2.809897	2.406545

H	-2.619370	-2.724051	1.957576
C	-0.989983	-1.409918	2.390930
H	0.009734	-1.437311	2.834309
H	-1.594425	-0.700025	2.966388
H	-0.870031	-1.016180	1.379455
C	-1.806735	-3.310015	3.842027
H	-2.183307	-4.338696	3.871576
H	-2.520528	-2.666984	4.367270
H	-0.868748	-3.274768	4.406879
C	0.280025	-4.459482	2.083651
H	0.467613	-4.425260	3.152330
C	1.116168	-5.205823	1.266045
H	1.943433	-5.760351	1.700737
C	0.917590	-5.230751	-0.109103
H	1.600810	-5.798718	-0.731534
C	-0.137781	-4.531020	-0.694755
C	-0.365428	-4.541170	-2.197743
H	-0.609155	-3.509983	-2.486191
C	0.873731	-4.950926	-2.996829
H	0.694727	-4.780215	-4.063330
H	1.105302	-6.015188	-2.872190
H	1.756308	-4.371773	-2.706701
C	-1.551911	-5.438813	-2.585615
H	-1.666993	-5.461809	-3.675332
H	-2.491942	-5.076442	-2.161117
H	-1.385124	-6.465881	-2.239818
C	3.863510	2.527715	1.800813
C	3.929349	2.405493	0.451385
C	2.100111	1.752931	-2.502343
C	2.693411	3.096894	-2.879346
H	3.071965	3.559669	-1.961326
C	3.865782	2.921391	-3.856927
H	3.497498	2.556839	-4.822334
H	4.591733	2.189268	-3.490917
H	4.384555	3.873064	-4.026622
C	1.663046	4.066611	-3.471382
H	1.202728	3.649311	-4.373848
H	2.142659	5.014491	-3.744554
H	0.865679	4.283069	-2.755545
C	1.448924	0.950569	-3.364082
H	1.317926	1.212889	-4.411787
C	0.934387	-0.361883	-2.859957
H	0.539844	-1.009891	-3.648817
C	1.975411	-1.030656	-2.016904
H	2.106465	-2.106608	-2.069278
C	3.021614	-2.153365	0.506438
H	1.994217	-2.049935	0.871206
H	3.657200	-2.493135	1.332973
H	3.023949	-2.946401	-0.248967
C	5.056291	2.748774	-0.456586
H	5.090604	2.068514	-1.311745
H	6.009191	2.666533	0.073051
H	4.982243	3.767983	-0.857777
C	4.830100	3.119752	2.760566
H	5.823569	3.174996	2.308651
H	4.902168	2.530050	3.681313
H	4.542326	4.137974	3.054844
C	2.155941	2.052951	3.587436
C	1.646854	3.222668	4.172275
C	1.320900	3.198272	5.530499
H	0.925550	4.092781	6.004108
C	1.489111	2.045394	6.279965
H	1.237212	2.042422	7.336912
C	1.962876	0.885847	5.677843
H	2.066243	-0.014932	6.273709
C	2.298960	0.861146	4.324536
C	2.807754	-0.409760	3.667549
H	2.370035	-0.440490	2.661867
C	4.339586	-0.402315	3.532834
H	4.685046	-1.356449	3.119687
H	4.807268	-0.269785	4.515946
H	4.689185	0.392523	2.869404
C	2.377194	-1.683982	4.401725
H	1.309670	-1.677318	4.642355
H	2.937506	-1.822084	5.334057
H	2.577018	-2.553703	3.766806
C	1.378162	4.476039	3.362994
H	1.808127	4.334371	2.364090
C	2.018300	5.727750	3.976842
H	3.091725	5.592416	4.140654
H	1.563095	5.977117	4.941381
H	1.878045	6.588720	3.313936
C	-0.139166	4.672702	3.209630
H	-0.353325	5.514363	2.539891
H	-0.598610	4.893579	4.179821
H	-0.617089	3.770754	2.814005
C	-0.442621	3.451839	-0.214274
H	0.106743	3.385864	0.716298
C	-1.168589	4.571068	-0.573347
H	-1.170158	5.431846	0.086908
C	-1.886067	4.571199	-1.766916
H	-2.472429	5.434712	-2.065830
C	-1.836840	3.432780	-2.560552
H	-2.387422	3.363360	-3.491321
C	-1.089414	2.344741	-2.149348
H	-1.058447	1.430266	-2.729347

Calculated energies and coordinates of **TS[INT1-INT2]**

Electronic energy ... -2884.21155483 Eh
Total Enthalpy ... -2882.78140296 Eh
Final Gibbs free energy ... -2882.95227095 Eh

CARTESIAN COORDINATES (ANGSTROEM)

N	-3.739512	1.182637	0.659169
N	-1.910566	-0.088978	-0.226865
N	2.031579	0.216741	0.181515
N	3.017551	-1.153264	-1.545128
N	-3.909380	-1.009509	0.709102
N	4.198566	-0.808471	0.290585
N	0.816827	2.132567	-0.773511
C	-3.714323	1.990265	-2.761327
H	-3.104169	2.732167	-3.290496
H	-4.370141	1.502726	-3.492319
H	-3.040438	1.239551	-2.338472
C	-4.542640	2.667544	-1.658205
H	-5.141816	1.884180	-1.177421
C	-3.622381	3.259281	-0.606349
C	-3.212511	2.499171	0.502141
C	-3.057441	0.019893	0.316132
B	-0.698413	0.041030	-0.772592
B	0.824870	0.669859	-0.469434
C	2.975363	-0.497159	-0.299278
C	1.797152	-1.269435	-2.252300
C	0.849034	-2.215404	-1.777663
C	1.239858	-3.357798	-0.858201
H	2.331305	-3.356732	-0.746934
C	0.827776	-4.710148	-1.455544
H	-0.263402	-4.782663	-1.534173
H	1.171315	-5.532970	-0.817455
H	1.247032	-4.849021	-2.457763
C	-5.512347	3.688627	-2.259542
H	-4.993511	4.419544	-2.888980
H	-6.055479	4.236706	-1.482387
H	-6.243260	3.177855	-2.895195
C	-2.332948	2.996268	1.473584
C	-1.832769	4.287717	1.293677
H	-1.127192	4.693805	2.013032
C	-2.225823	5.058307	0.211047
H	-1.822403	6.058957	0.087070
C	-3.119472	4.552910	-0.725880
H	-3.414833	5.172187	-1.567446

C	-1.944537	2.194237	2.700947
H	-2.466166	1.230789	2.659148
C	-0.439576	1.906334	2.737636
H	0.141771	2.833345	2.798375
H	-0.119894	1.363875	1.841981
H	-0.193250	1.296672	3.613985
C	-2.394528	2.905796	3.985800
H	-2.178357	2.280237	4.858882
H	-3.468659	3.118966	3.973672
H	-1.866585	3.856601	4.117114
C	-4.952492	0.868467	1.289354
C	-5.862553	1.937549	1.770656
H	-6.208845	2.575711	0.948061
H	-5.358009	2.592240	2.490904
H	-6.736376	1.501197	2.258329
C	-5.057339	-0.481807	1.327396
C	-6.130198	-1.363689	1.854177
H	-6.451716	-2.093005	1.101572
H	-6.996982	-0.766597	2.144709
H	-5.801376	-1.934780	2.731120
C	-3.614373	-2.397587	0.580298
C	-3.209287	-3.102480	1.724756
C	-2.964257	-2.387621	3.041257
H	-3.582131	-1.482659	3.065087
C	-1.498943	-1.933107	3.114863
H	-1.231707	-1.295171	2.266051
H	-0.831300	-2.801449	3.099028
H	-1.316114	-1.374432	4.040329
C	-3.332647	-3.230448	4.265823
H	-4.349139	-3.632052	4.191814
H	-3.270467	-2.616851	5.170481
H	-2.645265	-4.072411	4.400254
C	-2.953974	-4.468573	1.590984
H	-2.634213	-5.044143	2.454019
C	-3.080778	-5.096282	0.360490
H	-2.867393	-6.157738	0.270682
C	-3.467251	-4.374440	-0.763572
H	-3.547432	-4.884607	-1.717468
C	-3.748688	-3.010555	-0.679295
C	-4.196252	-2.209854	-1.892519
H	-3.641465	-1.262762	-1.869683
C	-3.888525	-2.891019	-3.228149
H	-4.510195	-3.781349	-3.377781
H	-2.836872	-3.180540	-3.315204
H	-4.111259	-2.199420	-4.047166
C	-5.696238	-1.872914	-1.824686
H	-5.932189	-1.218618	-0.981581
H	-6.291282	-2.789128	-1.733683
H	-6.005173	-1.358333	-2.741496
C	4.933964	-1.679501	-0.540578
C	4.197952	-1.908305	-1.652720
C	1.503570	-0.378632	-3.320626
C	2.628357	0.362607	-4.023356
H	3.500022	-0.305490	-4.033360
C	2.290269	0.699444	-5.477614
H	3.170334	1.116465	-5.979132
H	1.496883	1.452796	-5.535503
H	1.961879	-0.186227	-6.030993
C	3.041485	1.635908	-3.269580
H	2.218032	2.358145	-3.259610
H	3.903107	2.104125	-3.760507
H	3.313302	1.420806	-2.233296
C	0.174096	-0.189682	-3.625587
H	-0.131790	0.484933	-4.418221
C	-0.812907	-0.782749	-2.774236
H	-1.858407	-0.624805	-3.028822
C	-0.469276	-1.991046	-2.098241
H	-1.251910	-2.626947	-1.694423
C	0.628001	-3.177010	0.537561
H	0.985403	-3.957179	1.222084
H	-0.464594	-3.245346	0.490794
H	0.886227	-2.197942	0.952985
C	4.429275	-2.812634	-2.807257
H	5.331368	-3.408195	-2.651321
H	4.547501	-2.258794	-3.746637
H	3.583156	-3.497648	-2.944351
C	6.236439	-2.233983	-0.097061
H	6.129135	-2.787391	0.845041
H	6.982093	-1.450199	0.077595
H	6.634025	-2.915650	-0.851808
C	4.720695	-0.118361	1.423101
C	5.626803	0.940783	1.226066
C	6.113364	1.603754	2.354100
H	6.811300	2.427412	2.230494
C	5.711380	1.236920	3.631035
H	6.096910	1.770517	4.495484
C	4.819840	0.187551	3.803523
H	4.513504	-0.096419	4.806723
C	4.314582	-0.512610	2.707426
C	3.358704	-1.666655	2.924971
H	3.138103	-2.107884	1.947045
C	3.987374	-2.759127	3.800668
H	4.176833	-2.398548	4.817933
H	4.940167	-3.104790	3.385706
H	3.312167	-3.619090	3.873936
C	2.034104	-1.176272	3.520856
H	2.189933	-0.715121	4.503581
H	1.345646	-2.018037	3.648619
H	1.568864	-0.441447	2.857453
C	6.065097	1.397167	-0.155040
H	5.677052	0.685512	-0.893119
C	7.594950	1.431492	-0.291889
H	8.054611	0.484938	0.008464
H	8.032729	2.223078	0.326210
H	7.875264	1.633711	-1.331310
C	5.480621	2.777730	-0.487443
H	4.388593	2.768112	-0.452669
H	5.783022	3.087948	-1.494153
H	5.838597	3.532404	0.222694
C	1.704147	3.009280	-0.164874
H	2.338338	2.556762	0.588467
C	1.744738	4.339082	-0.486481
H	2.462739	4.969097	0.030876
C	0.884620	4.875445	-1.459782
H	0.930334	5.921207	-1.740900
C	-0.013176	3.992337	-2.068163
H	-0.698338	4.334450	-2.837914
C	-0.026734	2.660264	-1.740014
H	-0.655211	1.942154	-2.252255

Calculated energies and coordinates of **TS[INT1-INT2]$_{\mathbf{Py\text{-}CF3}}$**

Electronic energy ... -3221.27771193 Eh
Total Enthalpy ... -3219.83949274 Eh
Final Gibbs free energy ... -3220.01832079 Eh

CARTESIAN COORDINATES (ANGSTROEM)

N	-3.731400	-1.212054	0.076564
N	-2.015695	0.453088	-0.090470
N	1.890574	0.263490	0.428063
N	2.823768	2.274155	-0.519356
N	-4.086261	0.716742	1.065540
N	3.952208	1.290900	1.108024
N	0.883432	-1.119429	-1.334564
C	-0.446994	-2.475221	1.825048
H	-0.204037	-1.547063	1.299417
H	0.249681	-3.249316	1.483980
H	-0.276164	-2.315443	2.895524
C	-1.901784	-2.888504	1.577065
H	-2.543457	-2.076015	1.938647
C	-2.186743	-3.095454	0.101171
C	-3.091490	-2.291093	-0.606127

C	-3.149101	0.027691	0.303150
B	-0.800831	0.714644	-0.597400
B	0.766306	0.073067	-0.452907
C	2.791012	1.167566	0.351498
C	1.617031	2.592520	-1.186809
C	1.421076	2.205384	-2.544580
C	2.620335	1.924190	-3.434343
H	3.419024	2.610977	-3.123489
C	2.328714	2.191146	-4.912402
H	3.248367	2.087268	-5.498027
H	1.928001	3.197545	-5.070873
H	1.608568	1.467811	-5.310697
C	-2.252787	-4.146966	2.384828
H	-2.133151	-3.952714	3.456320
H	-1.594159	-4.981751	2.122452
H	-3.284667	-4.467138	2.205924
C	-3.424631	-2.529019	-1.949647
C	-2.810525	-3.604855	-2.587732
H	-3.042792	-3.823061	-3.625588
C	-1.885279	-4.396780	-1.918067
H	-1.396712	-5.215024	-2.438385
C	-1.571741	-4.139798	-0.592386
H	-0.842879	-4.765482	-0.084809
C	-4.384484	-1.616412	-2.690792
H	-5.042660	-1.143670	-1.950728
C	-3.607442	-0.487006	-3.385235
H	-4.295829	0.212249	-3.874470
H	-2.936376	-0.896052	-4.150038
H	-2.999897	0.067905	-2.664190
C	-5.273308	-2.358681	-3.692714
H	-4.698525	-2.732097	-4.546774
H	-6.036244	-1.680204	-4.088733
H	-5.779111	-3.211329	-3.227590
C	-4.973219	-1.293008	0.723749
C	-5.789343	-2.530934	0.660802
H	-6.703976	-2.411971	1.244621
H	-5.236994	-3.390324	1.058668
H	-6.068174	-2.780533	-0.370384
C	-5.192725	-0.106043	1.339690
C	-6.334353	0.371108	2.161273
H	-7.159547	-0.341719	2.105561
H	-6.698781	1.344961	1.814345
H	-6.060698	0.491045	3.216847
C	-3.912458	2.050066	1.538289
C	-4.102002	3.120121	0.644890
C	-4.492173	2.869585	-0.803550
H	-3.898401	2.015290	-1.152771
C	-4.192686	4.047449	-1.733785
H	-3.156521	4.389280	-1.649920
H	-4.856489	4.897236	-1.536865
H	-4.360219	3.741378	-2.771599
C	-5.976048	2.483031	-0.924240
H	-6.248475	2.369758	-1.979558
H	-6.612510	3.263470	-0.491292
H	-6.194050	1.536636	-0.422542
C	-3.934533	4.412048	1.144379
H	-4.062939	5.265333	0.486919
C	-3.601801	4.625925	2.477612
H	-3.475919	5.640757	2.844454
C	-3.420113	3.553591	3.338496
H	-3.145587	3.738220	4.372453
C	-3.564790	2.241203	2.884168
C	-3.271411	1.064213	3.796500
H	-3.788363	0.183179	3.400043
C	-1.767554	0.753794	3.774769
H	-1.547117	-0.126963	4.389018
H	-1.200899	1.601628	4.175419
H	-1.408282	0.566495	2.757663
C	-3.759954	1.278534	5.232734
H	-3.172057	2.042741	5.752136
H	-3.654714	0.349197	5.801940
H	-4.811539	1.583536	5.262515

C	4.637280	2.474674	0.757603
C	3.931269	3.088372	-0.219277
C	0.588859	3.213839	-0.424018
C	0.874591	3.915264	0.891233
H	1.961828	3.981464	1.020308
C	0.311867	5.342269	0.894314
H	0.700080	5.925525	0.052629
H	0.578327	5.855899	1.825387
H	-0.781868	5.331098	0.822198
C	0.314027	3.106739	2.069732
H	0.614195	3.553143	3.026363
H	0.670335	2.072210	2.034775
H	-0.780942	3.085303	2.034201
C	-0.699516	3.050914	-0.870606
H	-1.539996	3.428870	-0.295726
C	-0.930173	2.158154	-1.976273
H	-1.952898	2.086665	-2.342840
C	0.120597	2.064376	-2.963640
H	-0.120087	1.784313	-3.983398
C	3.156888	0.494865	-3.254895
H	4.068768	0.358978	-3.848030
H	2.417075	-0.238384	-3.592760
H	3.392784	0.277842	-2.210132
C	4.130759	4.395346	-0.894536
H	4.956161	4.940791	-0.432196
H	3.228505	5.016230	-0.827348
H	4.359087	4.275841	-1.960743
C	5.850061	2.895925	1.501727
H	5.616549	3.090662	2.556987
H	6.265164	3.808667	1.069225
H	6.630514	2.127915	1.486896
C	4.531001	0.207323	1.835678
C	4.127664	-0.016409	3.160676
C	4.700375	-1.085302	3.851523
H	4.398128	-1.284028	4.876134
C	5.653911	-1.894902	3.250540
H	6.092180	-2.720633	3.804248
C	6.048609	-1.650864	1.942374
H	6.794514	-2.291693	1.480090
C	5.494776	-0.602356	1.205759
C	5.934807	-0.388442	-0.233103
H	5.414881	0.492577	-0.626273
C	7.445321	-0.127706	-0.335332
H	7.718267	0.096929	-1.372080
H	8.019598	-1.007320	-0.024106
H	7.760550	0.713281	0.289142
C	5.555216	-1.587509	-1.113290
H	6.067025	-2.497797	-0.780668
H	5.843519	-1.400747	-2.153615
H	4.478944	-1.773867	-1.092955
C	3.091308	0.856451	3.837304
H	2.870637	1.692644	3.164525
C	3.612221	1.443496	5.155403
H	4.553290	1.983501	5.006588
H	3.788893	0.663601	5.904316
H	2.877919	2.141445	5.572883
C	1.788860	0.076598	4.053792
H	1.400257	-0.285641	3.097404
H	1.034235	0.720133	4.518287
H	1.953540	-0.783743	4.713836
C	0.111482	-1.247347	-2.483171
H	-0.565990	-0.429875	-2.689950
C	0.233536	-2.314852	-3.329745
H	-0.394803	-2.351625	-4.212235
C	1.166663	-3.327752	-3.069990
C	1.955326	-3.201200	-1.903365
H	2.686968	-3.960934	-1.644966
C	1.810755	-2.124592	-1.082301
H	2.382958	-1.989805	-0.171995
C	1.336539	-4.497910	-3.951811
F	0.488784	-4.483882	-5.009258
F	2.600674	-4.608689	-4.460891

F	1.122456	-5.686579	-3.296023

Calculated energies and coordinates of **INT2**

Electronic energy ... -2884.23623690 Eh
Total Enthalpy ... -2882.80356814 Eh
Final Gibbs free energy ... -2882.97726621 Eh

CARTESIAN COORDINATES (ANGSTROEM)

N	-3.504823	-1.941818	-1.292354
N	-1.360333	-1.139323	-0.580308
N	0.864825	1.659208	1.099221
N	2.992677	1.481265	-0.105036
N	-2.095047	-3.417095	-0.472989
N	3.009348	2.080353	2.009186
N	-1.097653	2.605319	-0.095411
C	-1.389036	-0.385257	-4.174474
H	-0.650973	-0.987219	-4.715737
H	-0.943609	-0.089547	-3.219028
H	-1.579125	0.522436	-4.759574
C	-2.681717	-1.187127	-3.969305
H	-2.425390	-2.089989	-3.402931
C	-3.682261	-0.387702	-3.159142
C	-4.043554	-0.746561	-1.851841
C	-2.226881	-2.069302	-0.767094
B	-0.796468	0.014300	-0.332079
B	-0.243778	1.422443	0.192595
C	2.131325	1.715289	0.979038
C	2.635355	0.943110	-1.373974
C	2.923469	-0.408815	-1.623641
C	3.394718	-1.312580	-0.497623
H	4.067555	-0.732800	0.146943
C	4.167720	-2.543536	-0.974567
H	3.514615	-3.254814	-1.492840
H	4.590346	-3.069051	-0.111779
H	4.988158	-2.276546	-1.650002
C	-3.269822	-1.642359	-5.311828
H	-3.488663	-0.790663	-5.964819
H	-4.198285	-2.205758	-5.170766
H	-2.554468	-2.285439	-5.836080
C	-4.940802	0.011602	-1.080803
C	-5.512452	1.136970	-1.672746
H	-6.208304	1.748196	-1.106754
C	-5.186469	1.500094	-2.973837
H	-5.643245	2.379966	-3.418904
C	-4.268144	0.757431	-3.701982
H	-4.007549	1.066303	-4.710396
C	-5.214750	-0.336784	0.369643
H	-5.010357	-1.406414	0.503839
C	-4.245248	0.438369	1.278698
H	-3.198220	0.220845	1.039331
H	-4.420283	0.179048	2.329183
H	-4.390387	1.518504	1.160867
C	-6.667697	-0.086673	0.785406
H	-6.900981	0.982720	0.822168
H	-6.840510	-0.487802	1.789426
H	-7.374292	-0.564618	0.097989
C	-4.141098	-3.192396	-1.316261
C	-5.522387	-3.339747	-1.838058
H	-5.822118	-4.389275	-1.821244
H	-5.598011	-2.977031	-2.870170
H	-6.244895	-2.766842	-1.243588
C	-3.276662	-4.099737	-0.800260
C	-3.425636	-5.550981	-0.527590
H	-4.433523	-5.881636	-0.785636
H	-3.253266	-5.772891	0.532653
H	-2.710119	-6.153221	-1.100632
C	-0.942799	-4.006838	0.120887
C	-0.721306	-3.835357	1.493879
C	-1.668486	-3.043388	2.374069
H	-2.500218	-2.688306	1.754931
C	-0.975045	-1.812887	2.970494
H	-0.130989	-2.110872	3.600592
H	-1.675050	-1.247125	3.595277
H	-0.605760	-1.143361	2.188371
C	-2.262537	-3.930153	3.478497
H	-2.751907	-4.816245	3.060136
H	-3.004501	-3.367816	4.055715
H	-1.488682	-4.270948	4.174960
C	0.406886	-4.445543	2.046715
H	0.610441	-4.331107	3.107939
C	1.264927	-5.202876	1.262850
H	2.134791	-5.673355	1.712772
C	1.022905	-5.359393	-0.097073
H	1.709270	-5.949516	-0.696390
C	-0.082621	-4.758999	-0.697988
C	-0.311778	-4.846720	-2.196370
H	-1.379818	-4.690294	-2.388251
C	0.445960	-3.712591	-2.903439
H	0.222400	-3.708896	-3.976957
H	1.525960	-3.851114	-2.781712
H	0.187613	-2.731399	-2.491481
C	0.068478	-6.206464	-2.789416
H	-0.246268	-6.256991	-3.836970
H	-0.407195	-7.030903	-2.247484
H	1.151367	-6.369797	-2.769874
C	4.341495	2.098730	1.557535
C	4.333277	1.717402	0.260821
C	2.091144	1.793047	-2.348784
C	1.762836	3.237657	-2.018117
H	1.406771	3.258981	-0.981992
C	3.012262	4.129873	-2.093164
H	3.455834	4.093465	-3.095438
H	3.769703	3.821793	-1.365973
H	2.742931	5.170316	-1.877083
C	0.641540	3.813351	-2.884915
H	0.976007	4.002658	-3.912118
H	0.305353	4.766517	-2.463647
H	-0.223698	3.143856	-2.916344
C	1.876338	1.263938	-3.621044
H	1.454674	1.889907	-4.401035
C	2.191633	-0.059320	-3.903505
H	2.031369	-0.449797	-4.905030
C	2.699101	-0.890898	-2.913611
H	2.927721	-1.923852	-3.152891
C	2.202589	-1.737370	0.375276
H	1.664435	-0.870615	0.771062
H	2.548166	-2.344070	1.220172
H	1.491944	-2.335324	-0.204714
C	5.449408	1.538253	-0.704047
H	5.251213	2.066669	-1.643979
H	5.614376	0.484716	-0.963814
H	6.375797	1.928969	-0.277020
C	5.477477	2.409517	2.466600
H	6.379470	2.617361	1.885928
H	5.698112	1.575555	3.144648
H	5.267051	3.279095	3.097305
C	2.610789	2.660362	3.248716
C	2.543094	4.058985	3.345475
C	2.346050	4.616776	4.611021
H	2.304905	5.696610	4.722495
C	2.182029	3.806676	5.724009
H	2.034045	4.254409	6.702921
C	2.164433	2.422571	5.588913
H	1.979208	1.811712	6.465612
C	2.370206	1.818670	4.348329
C	2.293195	0.308847	4.165758
H	1.703070	0.144970	3.252510
C	3.669721	-0.344911	3.959720
H	3.559621	-1.434669	3.916819
H	4.342028	-0.103772	4.791906
H	4.135601	-0.023702	3.025724

C	1.571204	-0.388846	5.320398
H	0.578852	0.037907	5.498730
H	2.147335	-0.331993	6.251552
H	1.447487	-1.450689	5.086795
C	2.621521	4.953726	2.118255
H	2.431953	4.326239	1.238866
C	3.997500	5.611558	1.935958
H	4.771455	4.877780	1.700147
H	4.291988	6.149687	2.844918
H	3.961104	6.333345	1.112066
C	1.536205	6.041108	2.155520
H	1.435244	6.498777	1.165474
H	1.791994	6.838155	2.862657
H	0.565325	5.630854	2.448603
C	-0.810789	3.833557	0.498824
H	0.023265	3.813412	1.187362
C	-1.559683	4.961215	0.256409
H	-1.270806	5.877982	0.763160
C	-2.655219	4.937647	-0.606512
H	-3.241903	5.824079	-0.814740
C	-2.950287	3.695028	-1.203242
H	-3.782489	3.586982	-1.890386
C	-2.190321	2.589308	-0.953184
H	-2.392395	1.637834	-1.436636

Calculated energies and coordinates of **TS[INT2-INT3]**

Electronic energy ... -3132.45264124 Eh
Total Enthalpy ... -3130.92611810 Eh
Final Gibbs free energy ... -3131.10582961 Eh

CARTESIAN COORDINATES (ANGSTROEM)

N	-3.031929	-1.448347	0.019644
N	-0.832859	-1.295045	-0.995974
N	0.781820	1.906804	0.439068
N	3.083360	1.050270	0.605177
N	-1.752580	-3.238905	0.003577
N	2.127581	1.997989	2.373341
N	-0.885384	2.470469	-1.307406
N	0.905823	-0.269229	-3.251801
C	-3.171386	-1.160469	-3.906113
H	-3.100884	-2.042536	-4.553877
H	-2.158187	-0.886997	-3.598523
H	-3.580760	-0.338091	-4.503474
C	-4.058620	-1.455945	-2.690684
H	-3.594122	-2.274583	-2.132189
C	-4.164251	-0.249763	-1.776451
C	-3.650261	-0.240578	-0.464602
C	-1.790812	-1.918145	-0.371369
B	-0.350644	-0.059303	-1.067714
B	-0.027042	1.471239	-0.657761
C	1.869045	1.643693	1.037692
C	3.483190	0.983148	-0.773300
C	3.750928	-0.262314	-1.368456
C	3.641961	-1.580697	-0.626626
H	3.353433	-1.361357	0.407734
C	4.991555	-2.319382	-0.605009
H	5.249491	-2.686902	-1.604373
H	4.932988	-3.189067	0.060062
H	5.811584	-1.678547	-0.268878
C	-5.445638	-1.950539	-3.125355
H	-5.962460	-1.207056	-3.741193
H	-6.078890	-2.167233	-2.258416
H	-5.351514	-2.866491	-3.719093
C	-3.825467	0.861763	0.389694
C	-4.495929	1.975514	-0.116299
H	-4.622174	2.848379	0.516751
C	-5.001546	1.993249	-1.407514
H	-5.509461	2.877433	-1.779606
C	-4.833924	0.886748	-2.226199
H	-5.229249	0.904909	-3.238260
C	-3.357061	0.889192	1.831448
H	-2.852035	-0.061221	2.045582
C	-2.360846	2.028838	2.076342
H	-1.453710	1.906031	1.480388
H	-2.070095	2.063990	3.131660
H	-2.803250	2.997526	1.821027
C	-4.549267	1.027533	2.793700
H	-5.008404	2.017925	2.701442
H	-4.210746	0.915536	3.830165
H	-5.329922	0.285544	2.600224
C	-3.749786	-2.492873	0.628681
C	-5.132741	-2.309191	1.137049
H	-5.709918	-1.662219	0.468480
H	-5.142724	-1.847065	2.130896
H	-5.640660	-3.273658	1.209805
C	-2.950716	-3.587951	0.644279
C	-3.166556	-4.942380	1.211593
H	-4.222052	-5.084139	1.454044
H	-2.586862	-5.093167	2.130351
H	-2.862148	-5.722509	0.505223
C	-0.688044	-4.163474	-0.265021
C	0.183679	-4.510432	0.777477
C	0.211923	-3.764975	2.096813
H	-0.703582	-3.166867	2.178074
C	1.410175	-2.799743	2.097346
H	2.336274	-3.340687	1.872263
H	1.528052	-2.327731	3.077592
H	1.284861	-2.012764	1.346794
C	0.273973	-4.698141	3.312410
H	-0.508843	-5.462833	3.279618
H	0.149804	-4.118257	4.233709
H	1.239723	-5.210075	3.379896
C	1.120839	-5.518949	0.540514
H	1.807855	-5.805722	1.331294
C	1.203169	-6.139007	-0.694598
H	1.938989	-6.919991	-0.863663
C	0.361300	-5.745584	-1.728977
H	0.457366	-6.223217	-2.697757
C	-0.604019	-4.758102	-1.540515
C	-1.539796	-4.360676	-2.669663
H	-1.648321	-3.269199	-2.628308
C	-0.999155	-4.707075	-4.060305
H	-1.621854	-4.224844	-4.820737
H	-1.032176	-5.786180	-4.249743
H	0.031548	-4.367473	-4.200897
C	-2.928558	-4.999681	-2.498965
H	-3.570710	-4.736157	-3.346958
H	-3.429336	-4.664703	-1.586818
H	-2.841050	-6.092035	-2.467200
C	3.449199	1.681995	2.724584
C	4.041696	1.130277	1.643516
C	3.708359	2.186313	-1.471500
C	3.582711	3.549433	-0.819970
H	3.258683	3.410415	0.214255
C	4.948365	4.250955	-0.769413
H	5.334894	4.444132	-1.776185
H	5.685529	3.640054	-0.237177
H	4.863488	5.212747	-0.251275
C	2.538431	4.422546	-1.524655
H	2.843683	4.657025	-2.550953
H	2.412766	5.372601	-0.992175
H	1.565764	3.923321	-1.561118
C	4.126003	2.113657	-2.800000
H	4.292774	3.031947	-3.357001
C	4.350478	0.890910	-3.415550
H	4.668214	0.852711	-4.453752
C	4.178236	-0.280376	-2.697340
H	4.369895	-1.234671	-3.179565
C	2.576398	-2.493692	-1.243357
H	1.590360	-2.026407	-1.236726
H	2.517925	-3.438499	-0.692253

H	2.827460	-2.728291	-2.284148
C	5.435705	0.648516	1.468896
H	5.838324	0.945641	0.493832
H	5.505065	-0.444148	1.531916
H	6.078717	1.069252	2.245562
C	3.972719	1.935344	4.090944
H	5.056568	1.801870	4.111692
H	3.536763	1.253090	4.831393
H	3.740969	2.953828	4.423893
C	1.178527	2.560329	3.278497
C	0.856558	3.928192	3.186043
C	0.000554	4.466953	4.146176
H	-0.265716	5.517610	4.098654
C	-0.513970	3.683036	5.171360
H	-1.168444	4.126300	5.916795
C	-0.211235	2.332292	5.228553
H	-0.646934	1.718545	6.011913
C	0.624696	1.743329	4.276745
C	0.813067	0.238417	4.282530
H	1.662848	-0.004941	3.634055
C	1.097427	-0.333792	5.676263
H	1.373250	-1.391517	5.596476
H	0.216209	-0.278295	6.324683
H	1.914462	0.195784	6.176882
C	-0.442290	-0.417343	3.681813
H	-0.543892	-0.168739	2.619900
H	-1.340421	-0.065469	4.202262
H	-0.400658	-1.506109	3.787280
C	1.447599	4.798602	2.092705
H	1.486573	4.184888	1.185741
C	2.881274	5.228281	2.442908
H	3.551137	4.367971	2.534927
H	2.896981	5.783034	3.388756
H	3.282120	5.881576	1.659394
C	0.597709	6.031478	1.772361
H	0.963816	6.498985	0.852551
H	0.654961	6.784695	2.566822
H	-0.455448	5.770944	1.622814
C	-1.045317	3.759848	-0.787412
H	-0.488091	3.951928	0.119530
C	-1.876455	4.682054	-1.363543
H	-1.955697	5.656216	-0.887461
C	-2.600447	4.393789	-2.532779
H	-3.227586	5.134499	-3.013942
C	-2.404093	3.116633	-3.082935
H	-2.904956	2.819623	-4.000290
C	-1.571089	2.206071	-2.497673
H	-1.402810	1.226357	-2.927003
C	0.948403	0.918965	-3.875158
H	1.204079	1.771857	-3.246829
C	0.568210	-1.341266	-3.991139
H	0.542750	-2.290220	-3.456773
C	0.260249	-1.272112	-5.342078
H	-0.011426	-2.173368	-5.885385
C	0.316642	-0.034505	-5.981718
H	0.080199	0.058726	-7.037527
C	0.676034	1.082222	-5.226530
H	0.735964	2.070262	-5.673138

Calculated energies and coordinates of **INT3**

Electronic energy ... -3132.49208136 Eh
Total Enthalpy ... -3130.96383754 Eh
Final Gibbs free energy ... -3131.14140678 Eh

CARTESIAN COORDINATES (ANGSTROEM)

N	-2.928244	-1.346752	0.038705
N	-0.807499	-1.302213	-1.188613
N	0.671365	1.775006	0.340223
N	3.003275	1.038543	0.488620
N	-1.650704	-3.151192	0.022168
N	2.001834	1.827581	2.296418
N	-1.052445	2.481128	-1.239163
N	0.415555	-0.117780	-2.942050
C	-3.221838	-1.207310	-3.934415
H	-3.158496	-2.117810	-4.541516
H	-2.202806	-0.886899	-3.700387
H	-3.694451	-0.431712	-4.547609
C	-4.027198	-1.469841	-2.657057
H	-3.516660	-2.254407	-2.091217
C	-4.118262	-0.233454	-1.783612
C	-3.604006	-0.187190	-0.473749
C	-1.696152	-1.829370	-0.435769
B	-0.223810	-0.088485	-1.593642
B	-0.134799	1.410255	-0.753286
C	1.768655	1.529964	0.948334
C	3.389024	0.965124	-0.894123
C	3.661708	-0.282156	-1.482656
C	3.616927	-1.593366	-0.721070
H	3.361076	-1.372502	0.321494
C	4.993239	-2.282847	-0.743767
H	5.214428	-2.674488	-1.742637
H	5.000132	-3.130794	-0.049079
H	5.804663	-1.601596	-0.471618
C	-5.428996	-2.003189	-2.989719
H	-5.995858	-1.287459	-3.594797
H	-6.002988	-2.202461	-2.078509
H	-5.354447	-2.936553	-3.558603
C	-3.836974	0.916831	0.364893
C	-4.512691	2.015336	-0.169851
H	-4.675302	2.891744	0.450456
C	-4.981417	2.007881	-1.473470
H	-5.490196	2.880405	-1.872311
C	-4.796922	0.884927	-2.265462
H	-5.189076	0.874352	-3.278956
C	-3.447147	0.947337	1.830593
H	-2.999149	-0.020352	2.084723
C	-2.419872	2.043026	2.126590
H	-1.483855	1.875692	1.590049
H	-2.194761	2.077183	3.197678
H	-2.805294	3.026082	1.833621
C	-4.684270	1.151910	2.723067
H	-5.068866	2.172917	2.625717
H	-4.415615	1.001119	3.775075
H	-5.498910	0.467271	2.469470
C	-3.616821	-2.376782	0.714138
C	-4.974403	-2.181973	1.285212
H	-5.594214	-1.566228	0.624709
H	-4.944852	-1.685208	2.262222
H	-5.469680	-3.146972	1.417089
C	-2.822432	-3.469901	0.726018
C	-3.029852	-4.806260	1.340029
H	-4.074051	-4.922894	1.638669
H	-2.408914	-4.949080	2.232925
H	-2.775568	-5.610643	0.640422
C	-0.623855	-4.108349	-0.256622
C	0.252126	-4.479865	0.774917
C	0.289460	-3.749435	2.101564
H	-0.642372	-3.183565	2.207738
C	1.452108	-2.744171	2.087990
H	2.389276	-3.248181	1.825287
H	1.579967	-2.288547	3.074988
H	1.277332	-1.948469	1.356152
C	0.411497	-4.690626	3.306272
H	-0.334232	-5.491510	3.274628
H	0.271013	-4.126633	4.235317
H	1.401565	-5.155956	3.358512
C	1.171165	-5.502693	0.530236
H	1.857154	-5.804707	1.316218
C	1.234735	-6.120543	-0.707563
H	1.957741	-6.911718	-0.885473
C	0.383946	-5.716897	-1.728594

H	0.455425	-6.200067	-2.696989
C	-0.566290	-4.716029	-1.526969
C	-1.534260	-4.337698	-2.633585
H	-1.672685	-3.251705	-2.583785
C	-1.013103	-4.664387	-4.036392
H	-1.654905	-4.188817	-4.784706
H	-1.024966	-5.742476	-4.234277
H	0.008344	-4.300628	-4.188746
C	-2.901819	-5.008810	-2.422543
H	-3.574708	-4.760813	-3.251348
H	-3.379100	-4.679201	-1.494953
H	-2.791118	-6.099139	-2.389685
C	3.337135	1.558456	2.635983
C	3.957531	1.100586	1.526446
C	3.597861	2.167309	-1.597075
C	3.476191	3.529970	-0.943514
H	3.201215	3.385660	0.104761
C	4.831186	4.254125	-0.951686
H	5.161567	4.471912	-1.973051
H	5.605981	3.647978	-0.470088
H	4.757528	5.205615	-0.413451
C	2.390178	4.388405	-1.600976
H	2.613265	4.569219	-2.658467
H	2.322781	5.363484	-1.104751
H	1.409540	3.908585	-1.533689
C	4.003174	2.091261	-2.928720
H	4.157801	3.007724	-3.492046
C	4.215557	0.867471	-3.544616
H	4.506612	0.824891	-4.589948
C	4.053894	-0.303424	-2.822373
H	4.238034	-1.258156	-3.305983
C	2.561144	-2.549608	-1.282875
H	1.550461	-2.152801	-1.167035
H	2.608450	-3.515026	-0.767932
H	2.730811	-2.731373	-2.349970
C	5.371625	0.692011	1.328489
H	5.745143	1.023077	0.353337
H	5.495515	-0.396423	1.373435
H	6.002552	1.132397	2.104233
C	3.854746	1.765607	4.012484
H	4.946861	1.741176	4.009867
H	3.506372	0.989339	4.704933
H	3.529839	2.729512	4.419925
C	1.072036	2.425924	3.206040
C	0.799916	3.806072	3.115099
C	-0.026738	4.374418	4.084152
H	-0.256199	5.433530	4.037266
C	-0.551831	3.613689	5.120906
H	-1.181968	4.081833	5.871944
C	-0.284329	2.256566	5.185820
H	-0.718412	1.661577	5.984167
C	0.519076	1.636200	4.226280
C	0.693289	0.131933	4.268321
H	1.522073	-0.133217	3.603314
C	1.017514	-0.400637	5.669567
H	1.296901	-1.458700	5.610281
H	0.152788	-0.332873	6.338569
H	1.843290	0.147108	6.134920
C	-0.579186	-0.539072	3.725447
H	-0.719908	-0.316657	2.663053
H	-1.461237	-0.178623	4.267418
H	-0.527362	-1.625184	3.852167
C	1.419715	4.668408	2.030640
H	1.440361	4.069354	1.113548
C	2.865592	5.047810	2.390393
H	3.508740	4.167131	2.481925
H	2.892401	5.595973	3.339727
H	3.290867	5.693625	1.613631
C	0.614996	5.936268	1.727502
H	0.987306	6.394085	0.805620
H	0.712750	6.681155	2.525696
H	-0.449594	5.721176	1.590176
C	-1.145702	3.731786	-0.624463
H	-0.648405	3.798180	0.333952
C	-1.826328	4.771023	-1.182641
H	-1.863325	5.712321	-0.641251
C	-2.480074	4.628619	-2.429725
H	-2.997890	5.459139	-2.894601
C	-2.399068	3.377362	-3.044458
H	-2.884452	3.190915	-3.998101
C	-1.697423	2.344082	-2.474502
H	-1.650113	1.365049	-2.931184
C	0.784697	1.078305	-3.569923
H	0.777133	1.964542	-2.950003
C	0.471555	-1.272811	-3.725403
H	0.197091	-2.180616	-3.205428
C	0.861159	-1.253752	-5.030247
H	0.885891	-2.194563	-5.573096
C	1.234631	-0.043690	-5.661600
H	1.516960	-0.014744	-6.707271
C	1.194040	1.113003	-4.879880
H	1.478453	2.076506	-5.292888

Calculated energies and coordinates of **TS[INT3-6^{C}-H]**

Electronic energy ... -3132.48887787 Eh
Total Enthalpy ... -3130.96160689 Eh
Final Gibbs free energy ... -3131.13779196 Eh

CARTESIAN COORDINATES (ANGSTROEM)

N	-2.875030	-1.358451	0.063767
N	-0.747672	-1.346895	-1.150373
N	0.634201	1.770538	0.408795
N	2.963617	1.023065	0.524568
N	-1.622259	-3.182007	0.053335
N	1.989732	1.822212	2.344236
N	-0.968555	2.450178	-1.323074
N	0.326931	-0.042224	-2.930006
C	-3.279640	-1.355165	-3.949934
H	-3.245862	-2.302575	-4.500136
H	-2.250879	-1.014036	-3.803412
H	-3.794385	-0.625416	-4.584679
C	-4.002513	-1.542716	-2.612781
H	-3.465632	-2.307270	-2.045001
C	-4.031997	-0.267352	-1.792350
C	-3.526221	-0.196487	-0.479646
C	-1.645893	-1.861581	-0.400132
B	-0.213455	-0.109466	-1.548953
B	-0.126169	1.392007	-0.711733
C	1.737207	1.518992	1.004549
C	3.318410	0.969953	-0.869082
C	3.554749	-0.266346	-1.495527
C	3.563807	-1.591232	-0.756780
H	3.314824	-1.396946	0.292917
C	4.967610	-2.221959	-0.811255
H	5.192870	-2.572413	-1.824466
H	5.017813	-3.089368	-0.142738
H	5.752271	-1.515376	-0.526695
C	-5.430010	-2.068687	-2.827719
H	-6.029304	-1.360624	-3.410482
H	-5.939160	-2.234289	-1.872212
H	-5.406879	-3.019558	-3.371776
C	-3.729085	0.942302	0.319862
C	-4.345902	2.050918	-0.263887
H	-4.482035	2.953323	0.324550
C	-4.786607	2.023096	-1.576971
H	-5.241836	2.905505	-2.016511
C	-4.651293	0.862941	-2.323977
H	-5.031002	0.834401	-3.341715
C	-3.389499	0.996981	1.797188
H	-2.935548	0.039270	2.077357
C	-2.397999	2.115580	2.128486

H	-1.443981	1.976312	1.617311
H	-2.204006	2.146516	3.206128
H	-2.800613	3.092125	1.836236
C	-4.668368	1.187489	2.633632
H	-5.068406	2.198471	2.499875
H	-4.443332	1.059401	3.698854
H	-5.456859	0.482384	2.356091
C	-3.583178	-2.381303	0.731001
C	-4.937841	-2.165341	1.301003
H	-5.540741	-1.525326	0.648125
H	-4.899832	-1.685320	2.286271
H	-5.456339	-3.120416	1.415294
C	-2.804893	-3.486145	0.746229
C	-3.031173	-4.818792	1.361523
H	-4.076389	-4.919725	1.662227
H	-2.410348	-4.970101	2.253349
H	-2.790227	-5.627708	0.662354
C	-0.599540	-4.146261	-0.219764
C	0.269870	-4.519637	0.816150
C	0.307300	-3.779690	2.137055
H	-0.627533	-3.218357	2.241726
C	1.463375	-2.767101	2.105433
H	2.402289	-3.268831	1.844297
H	1.593376	-2.296601	3.085162
H	1.278474	-1.983317	1.363220
C	0.439549	-4.708147	3.349908
H	-0.304180	-5.511394	3.330417
H	0.302012	-4.135097	4.273824
H	1.431434	-5.169618	3.401727
C	1.185838	-5.546705	0.577793
H	1.866630	-5.850629	1.367486
C	1.252040	-6.166691	-0.658830
H	1.971536	-6.962147	-0.831890
C	0.410179	-5.758528	-1.685342
H	0.486230	-6.240691	-2.653954
C	-0.534924	-4.751476	-1.490471
C	-1.485104	-4.360051	-2.607256
H	-1.624505	-3.275224	-2.544658
C	-0.939137	-4.664436	-4.005626
H	-1.569765	-4.178461	-4.756856
H	-0.944330	-5.739115	-4.221393
H	0.083617	-4.294826	-4.134769
C	-2.853815	-5.036806	-2.426009
H	-3.515345	-4.780420	-3.261452
H	-3.345047	-4.719784	-1.501159
H	-2.740870	-6.127187	-2.404698
C	3.329805	1.552311	2.665805
C	3.932724	1.087622	1.549197
C	3.512447	2.185368	-1.554563
C	3.456733	3.531382	-0.859329
H	3.143864	3.372330	0.175526
C	4.857053	4.161823	-0.810430
H	5.236597	4.358984	-1.819089
H	5.570805	3.502226	-0.305179
H	4.828331	5.113403	-0.267669
C	2.448987	4.482432	-1.511127
H	2.738062	4.731170	-2.538192
H	2.395165	5.420988	-0.947658
H	1.446039	4.046745	-1.534830
C	3.844718	2.135929	-2.907464
H	3.982633	3.063034	-3.457396
C	3.999771	0.923634	-3.562441
H	4.226601	0.901412	-4.624112
C	3.873645	-0.260819	-2.855250
H	4.026151	-1.205880	-3.367917
C	2.541313	-2.582786	-1.318385
H	1.519112	-2.214921	-1.212596
H	2.613931	-3.541860	-0.794180
H	2.725583	-2.772204	-2.381891
C	5.339556	0.660812	1.337251
H	5.698471	0.961484	0.347068
H	5.453355	-0.427475	1.409713
H	5.989225	1.116077	2.088637
C	3.863494	1.762033	4.035692
H	4.954402	1.708004	4.025489
H	3.498212	1.004930	4.740811
H	3.567519	2.739444	4.433209
C	1.065495	2.413049	3.264985
C	0.779893	3.789835	3.178495
C	-0.038451	4.350047	4.159276
H	-0.277414	5.407274	4.116557
C	-0.546467	3.582392	5.199227
H	-1.171047	4.043856	5.959019
C	-0.272086	2.226026	5.255250
H	-0.696601	1.625447	6.054348
C	0.526234	1.615215	4.285575
C	0.698707	0.110667	4.303160
H	1.546849	-0.143503	3.658263
C	0.978633	-0.455551	5.699731
H	1.253692	-1.513596	5.623391
H	0.096001	-0.398787	6.345903
H	1.795028	0.075686	6.199935
C	-0.559671	-0.536947	3.702077
H	-0.659116	-0.288417	2.640261
H	-1.455976	-0.177947	4.221237
H	-0.523062	-1.625874	3.806267
C	1.369310	4.655829	2.080529
H	1.388465	4.049559	1.168531
C	2.812011	5.064450	2.419271
H	3.470685	4.195203	2.511072
H	2.840947	5.621471	3.363356
H	3.217401	5.709842	1.631601
C	0.534087	5.903391	1.775770
H	0.890017	6.362698	0.848028
H	0.619994	6.656373	2.567594
H	-0.526275	5.662105	1.646919
C	-1.032985	3.743988	-0.819801
H	-0.633563	3.855150	0.181325
C	-1.563814	4.779122	-1.515697
H	-1.596534	5.760741	-1.053106
C	-2.091355	4.565442	-2.828763
H	-2.497763	5.389916	-3.404539
C	-2.083024	3.281799	-3.322404
H	-2.518786	3.053969	-4.291832
C	-1.490508	2.225363	-2.624994
H	-1.759787	1.199019	-2.846834
C	0.553470	1.238645	-3.500648
H	0.893242	2.001716	-2.809451
C	0.348139	-1.139537	-3.782259
H	0.193009	-2.091507	-3.288276
C	0.561682	-1.031036	-5.116480
H	0.580719	-1.933253	-5.719963
C	0.782900	0.254795	-5.704789
H	0.931195	0.357403	-6.774356
C	0.823733	1.345471	-4.867968
H	1.043594	2.337259	-5.254670

Calculated energies and coordinates of **6^{C}-H**

Electronic energy ... -3132.52738284 Eh
Total Enthalpy ... -3130.99659787 Eh
Final Gibbs free energy ... -3131.17326297 Eh

CARTESIAN COORDINATES (ANGSTROEM)

N	-2.852377	-1.391002	0.064142
N	-0.686247	-1.377104	-1.061115
N	0.559840	1.670472	0.473574
N	2.918411	1.046211	0.535114
N	-1.601581	-3.216307	0.108126
N	1.953289	1.793824	2.383412
N	-0.666928	2.482564	-1.500413
N	0.011152	-0.012230	-2.986362

C	-3.368476	-1.297085	-4.076023
H	-3.292901	-2.265623	-4.583033
H	-2.378436	-0.833808	-4.081434
H	-4.043819	-0.674365	-4.672987
C	-3.893656	-1.494372	-2.650828
H	-3.255864	-2.232599	-2.159585
C	-3.875411	-0.222741	-1.825563
C	-3.442229	-0.200475	-0.483802
C	-1.606261	-1.898680	-0.350587
B	-0.290582	-0.118571	-1.557204
B	-0.065231	1.372579	-0.755292
C	1.681468	1.484641	1.049262
C	3.221707	0.995631	-0.869778
C	3.429995	-0.240581	-1.508705
C	3.510720	-1.561703	-0.766779
H	3.274190	-1.374081	0.287229
C	4.943627	-2.121189	-0.850441
H	5.164360	-2.455667	-1.870167
H	5.049654	-2.987196	-0.186769
H	5.697931	-1.378179	-0.577955
C	-5.318616	-2.071384	-2.677015
H	-6.013378	-1.361275	-3.140151
H	-5.680872	-2.290267	-1.667312
H	-5.342854	-3.001047	-3.256474
C	-3.620885	0.940234	0.320247
C	-4.130997	2.092869	-0.281726
H	-4.246202	2.994902	0.311456
C	-4.491951	2.110110	-1.618680
H	-4.863532	3.025340	-2.070286
C	-4.390745	0.951183	-2.373952
H	-4.715594	0.958045	-3.410219
C	-3.368819	0.958126	1.816009
H	-2.948629	-0.013404	2.102654
C	-2.380310	2.050588	2.233352
H	-1.407144	1.918330	1.756609
H	-2.234084	2.036451	3.318784
H	-2.759321	3.044614	1.968515
C	-4.695944	1.157473	2.571984
H	-5.069200	2.176846	2.424695
H	-4.543727	1.012179	3.647782
H	-5.475687	0.469773	2.233627
C	-3.584575	-2.407206	0.715428
C	-4.945532	-2.175554	1.264520
H	-5.522984	-1.510025	0.614205
H	-4.916746	-1.716506	2.260161
H	-5.486575	-3.121408	1.348523
C	-2.810393	-3.513972	0.760709
C	-3.054717	-4.840627	1.381306
H	-4.102298	-4.928431	1.677870
H	-2.438702	-4.993802	2.276547
H	-2.820064	-5.656636	0.687828
C	-0.584644	-4.189088	-0.157395
C	0.280707	-4.562372	0.881488
C	0.319711	-3.813036	2.196462
H	-0.617949	-3.256175	2.300522
C	1.470092	-2.794152	2.147358
H	2.408090	-3.291812	1.875215
H	1.610882	-2.320223	3.123763
H	1.269900	-2.014210	1.404816
C	0.462693	-4.730956	3.415684
H	-0.280114	-5.535408	3.407860
H	0.330405	-4.150828	4.335924
H	1.455871	-5.189948	3.464873
C	1.193453	-5.593912	0.649515
H	1.871625	-5.897067	1.441702
C	1.258291	-6.220816	-0.583614
H	1.973635	-7.021228	-0.751128
C	0.421633	-5.812345	-1.614188
H	0.498664	-6.297420	-2.581334
C	-0.516532	-4.797566	-1.426401
C	-1.446119	-4.390378	-2.554444
H	-1.567498	-3.303967	-2.489443

C	-0.883816	-4.692652	-3.946677
H	-1.492490	-4.185587	-4.702111
H	-0.905113	-5.764982	-4.173207
H	0.147229	-4.339802	-4.055479
C	-2.829249	-5.042309	-2.396021
H	-3.466706	-4.786855	-3.250117
H	-3.336419	-4.703144	-1.487512
H	-2.734934	-6.133966	-2.357943
C	3.313687	1.580497	2.669415
C	3.907846	1.143532	1.537949
C	3.369772	2.212245	-1.567803
C	3.343151	3.556421	-0.868086
H	2.925619	3.413992	0.131211
C	4.778226	4.078999	-0.690845
H	5.262318	4.215682	-1.664697
H	5.387486	3.384448	-0.103748
H	4.771755	5.045557	-0.174522
C	2.479438	4.592767	-1.593640
H	2.926690	4.892405	-2.547801
H	2.389075	5.496901	-0.981074
H	1.472570	4.215049	-1.790553
C	3.634536	2.162941	-2.936160
H	3.740267	3.089615	-3.492885
C	3.754490	0.949994	-3.598624
H	3.924346	0.927965	-4.671105
C	3.669573	-0.234465	-2.885225
H	3.799206	-1.178529	-3.405543
C	2.530359	-2.609274	-1.301642
H	1.493016	-2.286035	-1.196880
H	2.648852	-3.551326	-0.755084
H	2.720251	-2.818925	-2.360430
C	5.317587	0.738232	1.301970
H	5.645421	1.011156	0.293281
H	5.453943	-0.345013	1.407649
H	5.978876	1.230493	2.019377
C	3.869473	1.802211	4.028167
H	4.960910	1.774210	3.995354
H	3.536301	1.036347	4.739992
H	3.559086	2.772532	4.432583
C	1.035742	2.367985	3.321281
C	0.718205	3.737999	3.237508
C	-0.083668	4.286391	4.238347
H	-0.344414	5.338627	4.197688
C	-0.548858	3.512995	5.293830
H	-1.160772	3.965180	6.069392
C	-0.249680	2.161758	5.344509
H	-0.643854	1.555533	6.154889
C	0.534954	1.563247	4.356135
C	0.731995	0.062031	4.370331
H	1.563599	-0.178291	3.699082
C	1.064142	-0.496867	5.758302
H	1.351504	-1.551182	5.675761
H	0.201831	-0.450403	6.432210
H	1.888644	0.047203	6.230632
C	-0.535042	-0.605696	3.809387
H	-0.666771	-0.366998	2.748831
H	-1.421043	-0.253819	4.350555
H	-0.482288	-1.693164	3.920728
C	1.248935	4.609801	2.114873
H	1.257977	3.995345	1.208584
C	2.687046	5.066408	2.407513
H	3.376756	4.219382	2.476949
H	2.726929	5.624414	3.350623
H	3.044374	5.725541	1.608081
C	0.364803	5.825013	1.818303
H	0.679699	6.280700	0.874189
H	0.446314	6.591316	2.597811
H	-0.689712	5.545317	1.721256
C	-0.949549	3.696048	-0.912984
H	-0.783185	3.713556	0.159378
C	-1.370556	4.783192	-1.594089
H	-1.584130	5.707337	-1.070517

C	-1.474492	4.689345	-3.031846
H	-1.723991	5.578303	-3.606671
C	-1.268104	3.525427	-3.666668
H	-1.393576	3.459286	-4.742288
C	-1.001334	2.237292	-2.936668
H	-1.907516	1.604501	-3.005376
C	0.148787	1.368382	-3.541290
H	1.080371	1.823968	-3.156377
C	0.197950	-1.115949	-3.788503
H	0.167474	-2.057184	-3.248978
C	0.385994	-1.056338	-5.124036
H	0.535933	-1.964493	-5.695555
C	0.351188	0.238843	-5.762907
H	0.448233	0.301700	-6.844368
C	0.214680	1.362778	-5.043339
H	0.245001	2.331594	-5.531015

4. EPR spectroscopy

The EPR spectra were recorded on a Bruker EMX-10/12 X-band (v= 9.3 GHz) digital EPR spectrometer equipped with a Bruker N2-temperature controller. The spectra were recorded at a microwave power of 100-200 mW, 100 kHz magnetic field modulation of 1.0 G amplitude. Digital field resolution was 2048 points per spectrum. When measurements were carried out under UV irradiation, a high-pressure mercury lamp (1 kW) (ARC lamp power supply model 69920) was used, with the output being focused on the sample with a quartz lens and filtered through distilled water to remove infrared radiation.

We attempted to directly observe the $\mathbf{1}_{\mathbf{INT}}^{\mathbf{T}}$ using EPR spectroscopy. The sample was measured in toluene at temperatures 170 K and above under continuous photoirradiation. Without irradiation no signal is observed. Upon irradiation at 170 K a radical species is observed (**Figure S72**).

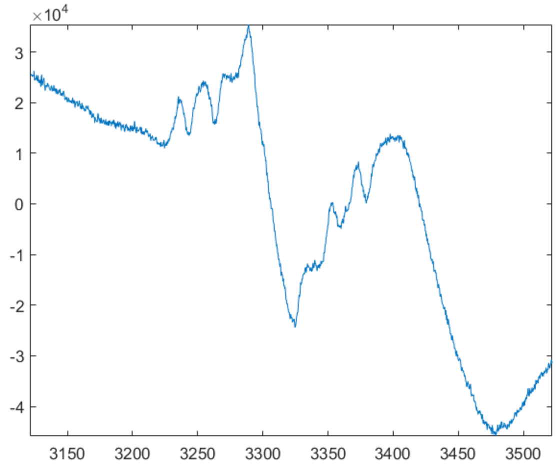

Figure S72. EPR spectrum of **2** in toluene at 173 K under UV irradiation.

The radical species is persistent when the irradiation is stopped. The signal becomes sharper when the solution is irradiated at 183 K and 200 K (**Figure S73**).

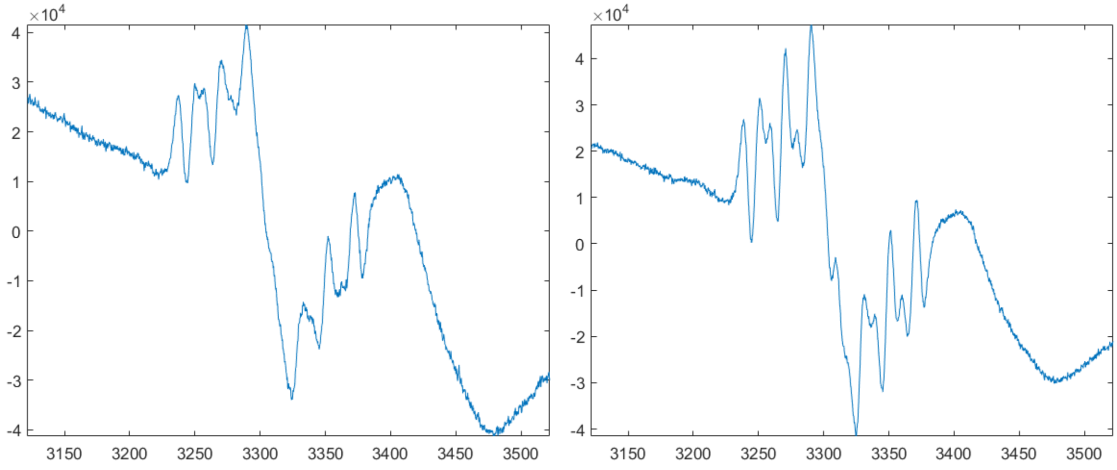

Figure S73. EPR spectra of **2** in toluene at 183 K and 200 K under UV irradiation.

The signal disappears when the temperature is raised to 230 K. However, DFT simulations indicate that these paramagnetic species cannot be assigned to $\mathbf{1}_{\mathbf{INT}}$ or to the other diradical intermediates shown in Figure 7. We therefore propose that the observed radical species most likely arises from a secondary radical or decomposition products of the transient triplet diradical intermediate $\mathbf{1}_{\mathbf{INT}}{}^{\mathbf{T}}$. It is possible that owing to its high reactivity and extremely short lifetime $\mathbf{1}_{\mathbf{INT}}{}^{\mathbf{T}}$ decays on a timescale beyond the detection limit of EPR spectroscopy.

5. References

(1) Fulmer, G. R.; Miller, A. J. M.; Sherden, N. H.; Gottlieb, H. E.; Nudelman, A.; Stoltz, B. M.; Bercaw, J. E.; Goldberg, K. I. NMR Chemical Shifts of Trace Impurities: Common Laboratory Solvents, Organics, and Gases in Deuterated Solvents Relevant to the Organometallic Chemist. *Organometallics*, **2010**, *29*, 2176–2179.

(2) Muhr, M.; Heiß, P.; Schütz, M.; Bühler, R.; Gemel, C.; Linden, M. H.; Linden, H. B.; Fischer, R. A. Enabling LIFDI-MS measurements of highly air sensitive organometallic compounds: a combined MS/glovebox technique. *Dalton Trans.* **2021**, *50*, 9031–9036.

(3) Tamm, M.; Randoll, S.; Herdtweck, E.; Kleigrewe, N.; Kehr, G.; Erker, G.; Rieger, B., Imidazolin-2-iminato titanium complexes: synthesis, structure and use in ethylene polymerization catalysis. *Dalton Trans.* **2006,** 3, 459-67.

(4) Brown, H. C.; Ravindran, N., Molecular addition compounds. 3. Redistribution of borane-methyl sulfide with boron trichloride-methyl sulfide and boron tribromide-methyl sulfide as convenient routes to the corresponding haloborane-methyl sulfides. *Inorg. Chem.* **1977**, *16*, 2938-2940.

(5) Arrowsmith, M.; Böhnke, J.; Braunschweig, H.; Deißenberger, A.; Dewhurst, R. D.; Ewing, W. C.; Hörl, C.; Miesa, J.; Muessig, J. H., Simple solution-phase syntheses of tetrahalodiboranes(4) and their labile dimethylsulfide adducts. *Chem. Commun.* **2017**, *53*, 8265-8267.

(6) Shuya Y.; Takeshi K.; Philip S.; Kei M.; Kenichiro I., Dehydrogenative Synthesis of 2,2”-Bipyridyls through RegioselectivePyridine Dimerization. *Angew. Chem., Int. Ed.* **2019**, *58,* 8341 – 8345.

(7) APEX suite of crystallographic software, *APEX 3 version 2015.5-2*; Bruker AXS Inc.: Madison, Wisconsin, USA, **2015**.

(8) SAINT, Version 7.56a and SADABS Version 2008/1; Bruker AXS Inc.: Madison, Wisconsin, USA, **2008**.

(9) Sheldrick, G. M., SHELXL-2014, University of Göttingen, Göttingen, Germany, **2014**.

(10) Hübschle, C. B., Sheldrick, G. M., Dittrich, B., *J. Appl. Cryst.* **2011**, *44*, 1281-1284.

(11) Sheldrick, G. M., SHELXL-97, University of Göttingen, Göttingen, Germany, **1998**.

(12) Wilson, A. J. C. International Tables for Crystallography, Vol. C, Tables 6.1.1.4 (pp. 500-502), 4.2.6.8 (pp. 219-222), and 4.2.4.2 (pp. 193-199); Kluwer Academic Publishers: Dordrecht, The Netherlands, **1992**.

(13) Macrae, C. F.; Bruno, I. J.; Chisholm, J. A.; Edgington, P. R.; McCabe, P.; Pidcock, E.; Rodriguez-Monge, L.; Taylor, R.; van de Streek, J.; Wood, P. A., *J. Appl. Cryst.* **2008**, *41*, 466-470.

(14) Neese, F.; Wennmohs, F.; Becker, U.; Riplinger, C., The ORCA quantum chemistry program package. *J. Chem. Phys.* **2020**, *152*, 224108.

(15) Grimme, S.; Hansen, A.; Ehlert, S.; Mewes, J.-M. r2SCAN-3c: A "Swiss army knife" composite electronic-structure method. *J. Chem. Phys.* **2021**, *154*, 64103.

(16) Furness, J. W.; Kaplan, A. D.; Ning, J.; Perdew, J. P.; Sun, J. Accurate and Numerically Efficient r2SCAN Meta-Generalized Gradient Approximation. *J. Phys. Chem. Lett.* **2020**, *11*, 8208–8215.

(17) Furness, J. W.; Kaplan, A. D.; Ning, J.; Perdew, J. P.; Sun, J. Correction to "Accurate and Numerically Efficient r2SCAN Meta-Generalized Gradient Approximation". *J. Phys. Chem. Lett.* **2020**, *11*, 9248.

(18) Kruse, H.; Grimme, S. A geometrical correction for the inter- and intra-molecular basis set superposition error in Hartree-Fock and density functional theory calculations for large systems. *J. Chem. Phys.* **2012**, *136*, 154101.

(19) Caldeweyher, E.; Bannwarth, C.; Grimme, S. Extension of the D3 dispersion coefficient model. *J. Phys. Chem.* **2017**, *147*, 34112.

(20) Caldeweyher, E.; Ehlert, S.; Hansen, A.; Neugebauer, H.; Spicher, S.; Bannwarth, C.; Grimme, S. A generally applicable atomic-charge dependent London dispersion correction. *J. Chem. Phys.* **2019**, *150*, 154122.

(21) Caldeweyher, E.; Mewes, J.-M.; Ehlert, S.; Grimme, S. Extension and evaluation of the D4 London-dispersion model for periodic systems. *Phys. Chem. Chem. Phys.* **2020**, *22*, 8499–8512.

(22) Marenich, A. V.; Cramer, C. J.; Truhlar, D. G. Universal solvation model based on solute electron density and on a continuum model of the solvent defined by the bulk dielectric constant and atomic surface tensions. *J. Phys. Chem. B*. **2009**, *113*, 6378–6396.

(23) Zhao, Y.; Truhlar, D. G. Design of density functionals that are broadly accurate for thermochemistry, thermochemical kinetics, and nonbonded interactions. *J. Phys. Chem. A*. **2005**, *109*, 5656–5667.
(24) Weigend, F.; Ahlrichs, R. Balanced basis sets of split valence, triple zeta valence and quadruple zeta valence quality for H to Rn: Design and assessment of accuracy. *Phys. Chem. Chem. Phys.* **2005**, *7*, 3297–3305.
(25) Weigend, F. Accurate Coulomb-fitting basis sets for H to Rn. *Phys. Chem. Chem. Phys.* **2006**, *8*, 1057–1065.
(26) Hellweg, A.; Hättig, C.; Höfener, S.; Klopper, W. Optimized accurate auxiliary basis sets for RI-MP2 and RI-CC2 calculations for the atoms Rb to Rn. *Theor. Chem. Acc.* **2007**, *117*, 587–597.
(27) Adamo, C.; Barone, V. Toward reliable density functional methods without adjustable parameters: The PBE0 model. *J. Chem. Phys.* **1999**, *110*, 6158–6170.
(28) Glendening, E. D.; Badenhoop, J, K.; Reed, A. E.; Carpenter, J. E.; Bohmann, J. A.; Morales, C. M.; Karafiloglou, P.; Landis, C. R.; Weinhold, F. NBO 7.0; Theoretical Chemistry Institute, University of Wisconsin, Madison, **2018**.
(29) Bçhnke, J.; Braunschweig, H.; Dellermann, T.; Ewing, W. C.; Kramer, T.; Krummenacher, I.; Vargas, A. From an Electron-Rich Bis(boraketenimine) to an Electron-Poor Diborene. *Angew. Chem., Int. Ed.* **2015**, *54*, 4469 –4473.
(30) Jouany, C.; Barthelat, J. C.; Daudey, J. P. Stabilization of a π double bond between two boron atoms. *Chem. Phys. Lett.* **1987**, *136*, 52-56.
(31) Lehmann, A.; Queen, J. D.; Roberts, C. J.; Rissanen, K.; Tuononen, H. M.; Power, P. P. The Dialuminene Ar(iPr8)AlAlAr(iPr8) (Ar(iPr8)=C(6)H-2,6-(C(6)H(2)-2,4,6-(i)Pr(3))(2)-3,5-(i)Pr(2)). *Angew. Chem., Int. Ed.* **2024**, *63*, e202412599.
(32) Hardman, N. J.; Wright, R. J.; Phillips, A. D.; Power, P. P. Synthesis and Characterization of the Neutral "Digallene" Ar′GaGaAr′ and Its Reduction to Na2Ar′GaGaAr′ (Ar′=2,6-Dipp2C6H3, Dipp=2,6-iPr2C6H3). *Angew. Chem., Int. Ed.* **2002**, *41*, 2842-2844.

(33) Wright, R. J.; Phillips, A. D.; Hardman, N. J.; Power, P. P. The "diindene" ArInInAr (Ar = C(6)H(3)-2,6-Dipp(2), Dipp = C(6)H(3)-2,6-Pr(i)(2)). Dimeric versus monomeric in(I) aryls: para-substituent effects in terphenyl ligands. *J. Am. Chem. Soc.* **2002**, *124*, 8538-8539.

(34) Wright, R. J.; Phillips, A. D.; Hino, S.; Power, P. P. Synthesis and reactivity of dimeric Ar'TlTlAr' and trimeric (Ar"T1)3 (Ar', Ar" = bulky terphenyl group) thallium(I) derivatives: Tl(I)-Tl(I) bonding in species ligated by monodentate ligands. *J. Am. Chem. Soc.* **2005**, *127*, 4794-4799.

(35) Arrowsmith, M.; Braunschweig, H.; Stennett, T. E. Formation and Reactivity of Electron-Precise B-B Single and Multiple Bonds. *Angew. Chem., Int. Ed.* **2017**, *56*, 96-115.

(36) Wang, Y.; Quillian, B.; Wei, P.; Wannere, C. S.; Xie, Y.; King, R. B.; Schaefer, H. F., 3rd; Schleyer, P. V.; Robinson, G. H. A stable, neutral diborene containing a B=B double bond. *J. Am. Chem. Soc.* **2007**, *129*, 12412-12413.

(37) Bissinger, P.; Braunschweig, H.; Celik, M. A.; Claes, C.; Dewhurst, R. D.; Endres, S.; Kelch, H.; Kramer, T.; Krummenacher, I.; Schneider, C. Synthesis of cyclic diborenes with unprecedented cis-configuration. *Chem. Commun (Camb)* **2015**, *51*, 15917-15920.

(38) Fan, J.; Mah, J. Q.; Yang, M. C.; Su, M. D.; So, C. W. A N-Phosphinoamidinato NHC-Diborene Catalyst for Hydroboration. *J. Am. Chem. Soc.* **2021**, *143*, 4993-5002.

(39) Xu, C.; Cao, F.; Chen, X.; Chen, M.; Mo, Z. Crystalline Silylene-Stabilized Diboryne and Siladiborirene. *J. Am. Chem. Soc.* **2025**, *147*, 1207-1213.

(40) Braunschweig, H.; Dewhurst, R. D. Single, double, triple bonds and chains: the formation of electron-precise B-B bonds. *Angew. Chem., Int. Ed.* **2013**, *52*, 3574-3583.

(41) Arrowsmith, M.; Bohnke, J.; Braunschweig, H.; Celik, M. A. Reactivity of a Dihydrodiborene with CO: Coordination, Insertion, Cleavage, and Spontaneous Formation of a Cyclic Alkyne. *Angew. Chem., Int. Ed.* **2017**, *56*, 14287-14292.

(42) Lu, W.; Li, Y.; Ganguly, R.; Kinjo, R. Alkene-Carbene Isomerization induced by Borane: Access to an Asymmetrical Diborene. *J. Am. Chem. Soc.* **2017**, *139*, 5047-5050.

(43) Stennett, T. E.; Mattock, J. D.; Vollert, I.; Vargas, A.; Braunschweig, H. Unsymmetrical, Cyclic Diborenes and Thermal Rearrangement to a Borylborylene. *Angew. Chem., Int. Ed.* **2018**, *57*, 4098-4102.

(44) Muessig, J. H.; Thaler, M.; Dewhurst, R. D.; Paprocki, V.; Seufert, J.; Mattock, J. D.; Vargas, A.; Braunschweig, H. Phosphine-Stabilized Diiododiborenes: Isolable Diborenes with Six Labile Bonds. *Angew. Chem., Int. Ed.* **2019**, *58*, 4405-4409.

(45) Fan, J.; Yang, M. C.; Su, M. D.; So, C. W. Amidinatoamidosilylene-Dibromodiborene. *Inorg. Chem.* **2021**, *60*, 16065-16069.

(46) Lu, W.; Jayaraman, A.; Fantuzzi, F.; Dewhurst, R. D.; Harterich, M.; Dietz, M.; Hagspiel, S.; Krummenacher, I.; Hammond, K.; Cui, J.; et al. An Unsymmetrical, Cyclic Diborene Based on a Chelating CAAC Ligand and its Small-Molecule Activation and Rearrangement Chemistry. *Angew. Chem., Int. Ed.* **2022**, *61*, e202113947.

(47) Pyykko, P.; Atsumi, M. Molecular double-bond covalent radii for elements Li-E112. *Chem. Eur. J.* **2009**, *15*, 12770-12779.

(48) Huang, F.; Wang, X. F.; Ma, B.; Tao, L.; Kong, L.; Liu, L. L.; Liang, Q. A Crystalline Monomeric Diboryl Diazene. *J. Am. Chem. Soc.* **2025**, *147*, 37919–37925.

(49) Frank H. Allen, O. K., and David G. Watson. Tables of Bond Lengths determined by X-Ray and Neutron Diffraction. *J. Chem. Soc. Perkin Trans.* **1987**, *2*, S1-S19.

(50) Dai, Y.; Cui, P.; Tung, C. H.; Kong, L. Anionic Iminoborane as Boryl Anion Synthon. *Angew. Chem., Int. Ed.* **2025**, e202518214.

(51) Knight, L. B.; Kerr, K.; Miller, P. K.; Arrington, C. A. ESR Investigation of the HBBH(X3.SIGMA.) Radical in Neon and Argon Matrixes at 4 K. Comparison with ab Initio SCF and CI Calculations. *J. Phy. Chem.* **1995**, *99*, 16842-16848.

(52) Braunschweig, H.; Damme, A.; Dewhurst, R. D.; Vargas, A. Bond-strengthening pi backdonation in a transition-metal pi-diborene complex. *Nat. Chem.* **2013**, *5*, 115-121.

(53) Dill, J. D.; Schleyer, P. v. R.; Pople, J. A. Molecular orbital theory of the electron structure of organic compounds. XXIV. Geometries and energies of small boron compounds. Comparisons with carbocations. *J. Am. Chem. Soc.* **1975**, *97*, 3402-3409.

(54) Treboux, G.; Barthelat, J. C. X-X direct bonds versus bridged structures in group 13 X2H2 potential energy surfaces. *J. Am. Chem. Soc.* **1993**, *115*, 4870-4878.

(55) Zhu, H.; Kostenko, A.; Franz, D.; Hanusch, F.; Inoue, S. Room Temperature Intermolecular Dearomatization of Arenes by an Acyclic Iminosilylene. *J. Am. Chem. Soc.* **2023**, *145*, 1011-1021.

(56) Zhu, H.; Fujimori, S.; Kostenko, A.; Inoue, S. Dearomatization of C6 Aromatic Hydrocarbons by Main Group Complexes. *Chem. Eur. J.* **2023**, e202301973.

(57) isner, T.; Kostenko, A.; Hanusch, F.; Inoue, S. Room-Temperature-Observable Interconversion Between Si(IV) and Si(II) via Reversible Intramolecular Insertion Into an Aromatic C-C Bond. *Chem. Eur. J.* **2022**, *28*, e202202330.

(58) Liu, L. L.; Zhou, J.; Cao, L. L.; Kim, Y.; Stephan, D. W. Reversible Intramolecular Cycloaddition of Phosphaalkene to an Arene Ring. *J. Am. Chem. Soc.* **2019**, *141*, 8083-8087.

(59) Bissinger, P.; Braunschweig, H.; Kraft, K.; Kupfer, T. Trapping the elusive parent borylene. *Angew. Chem., Int. Ed.* **2011**, *50*, 4704-4707.

(60) Wendel, D.; Porzelt, A.; Herz, F. A. D.; Sarkar, D.; Jandl, C.; Inoue, S.; Rieger, B. From Si(II) to Si(IV) and Back: Reversible Intramolecular Carbon-Carbon Bond Activation by an Acyclic Iminosilylene. *J. Am. Chem. Soc.* **2017**, *139*, 8134-8137.

(61) Hicks, J.; Vasko, P.; Goicoechea, J. M.; Aldridge, S. Reversible, Room-Temperature C-C Bond Activation of Benzene by an Isolable Metal Complex. *J. Am. Chem. Soc.* **2019**, *141*, 11000-11003.

(62) Guo, R.; Jiang, J.; Hu, C.; Liu, L. L.; Cui, P.; Zhao, M.; Ke, Z.; Tung, C. H.; Kong, L. BNN-1,3-dipoles: isolation and intramolecular cycloaddition with unactivated arenes. *Chem. Sci.* **2020**, *11*, 7053-7059.

(63) Kong, R. Y.; Crimmin, M. R. Chemoselective C-C sigma-Bond Activation of the Most Stable Ring in Biphenylene*. *Angew. Chem., Int. Ed.* **2021**, *60*, 2619-2623.

(64) Feng, G.; Chan, K. L.; Lin, Z.; Yamashita, M. Al-Sc Bonded Complexes: Synthesis, Structure, and Reaction with Benzene in the Presence of Alkyl Halide. *J. Am. Chem. Soc.* **2022**, *144*, 22662-22668.

(65) Qiu, S.; Zhang, X.; Hu, C.; Chu, H.; Li, Q.; Ruiz, D. A.; Liu, L. L.; Tung, C. H.; Kong, L. Unveiling Hetero-Enyne Reactivity of Aryliminoboranes: Dearomative Hetero-Diels-Alder-Like Reactions. *Angew. Chem., Int. Ed.* **2022**, *61*, e202205814.

(66) Zhang, X.; Liu, L. L. Modulating the Frontier Orbitals of an Aluminylene for Facile Dearomatization of Inert Arenes. *Angew. Chem., Int. Ed.* **2022**, *61*, e202116658.

(67) Kern, R. H.; Schneider, M.; Eichele, K.; Schubert, H.; Bettinger, H. F.; Wesemann, L. Boradigermaallyl: (4+3) Cycloaddition-Initiated Boron Insertion into Benzene. *Angew. Chem., Int. Ed.* **2023**, *62*, e202301593.
